# Alkynylated and triazole-linked aroyl-*S*,*N*-ketene acetals: one-pot synthesis of solid-state emissive dyes with aggregation-induced enhanced emission characteristics

**DOI:** 10.1038/s41598-023-41146-w

**Published:** 2023-09-01

**Authors:** Lukas Biesen, Yannic Hartmann, Thomas J. J. Müller

**Affiliations:** Institut für Organische Chemie und Makromolekulare Chemie, Heinrich-Heine-Universität Düsseldorf, Universitätsstraße 1, 40225 Düsseldorf, Germany

**Keywords:** Chemistry, Organic chemistry, Physical chemistry

## Abstract

Alkynylated aroyl-*S*,*N*-ketene acetals are readily synthesized in mostly excellent yields by a Sonogashira reaction resulting in a substance library of more than 20 examples. Upon expansion of the reaction sequence by deprotection and concatenating of the copper-click reaction in a one-pot fashion, a library of 11 triazole-ligated aroyl-*S*,*N*-ketene acetals is readily accessible. All derivatives show pronounced solid-state emission and aggregation-induced emission properties depending on the nature of the alkynyl or the triazole substituents.

## Introduction

The phenomenon of aggregation-induced emission (AIE) has gained a massive boom of interest in the last years^1,2^ since Tang coined the term in 2001^3^. Aggregation of dyes by enhancing the emission has been known since Scheibe and Jelley^4–6^, but the full potential of AIE is only starting to unfold^7^. In 2020, the IUPAC recognized its relevance by naming AIE one of the “Top Ten Emerging Technologies in Chemistry”^8^, thus recognizing the myriad of possibilities to apply AIEgens^9,10^. There are basically no boundaries for AIE chromophores getting used in countless fields of academia and industry, from obvious ones like OLEDs^11^ or photovoltaics^12^ to sensing technologies for biological^13^ and medicinal applications^14,15^, in bioimaging^16^, metal ion detection^17^ up to comparably surprising ones like radiation identification^18^, fingerprint analysis^19^ or as smart materials^20,21^.

Especially for a successful application in biological systems, modifications of the chromophore parent structure are required in order to ensure the full potential to unfold^22,23^. A very common method awarded with last year’s Nobel Prize for Chemistry^24,25^ is the copper-click reaction, i.e. the Cu-catalyzed alkyne-azide cycloaddition (CuAAC), to form 1,2,3-triazoles^26^. The CuAAC offers many highly advantageous traits, such as broad biocompatibility, readily accessible and cheap starting materials, and high tolerance of various functional groups^27–29^. A good, facile and direct approach to highly diversified and functionalized compounds has always been the holy grail for chemists, and dye and material chemistry is certainly no exception to this rule^30^. A concept that emphasizes ideal syntheses^31^ are one-pot procedures, which enable direct access to complex structures without isolating intermediates, thus saving time and resources^32^. For chromophores, this allows for easy and swift modifications and opens alleys for conceiving a plethora of properties and applications^33–35^.

Aroyl-*S*,*N*-ketene acetals have been proven since their recent renaissance as versatile and easily accessible AIEgens with a rich bouquet of properties^36,37^. In previous works, we already succeeded in expanding the reaction sequence in a one-pot fashion to synthesize bi- and multichromophores^38,39^. With this work, we aim to establish a novel CuAAC embedding one-pot reaction^40^ via intermediary generation of alkynylated aroyl-*S*,*N*-ketene acetals to directly access triazole aroyl-*S*,*N*-ketene acetals. Additionally, the effects of both alkynylation and 1,2,3-triazole ligation on the photophysical properties of aroyl-*S*,*N*-ketene acetals in general and the AIE properties in specific are elucidated.

## Results and discussion

### Alkynylated aroyl-*S*,*N*-ketene acetals

The importance, in particular, of the *para*-position of the benzyl moiety of the aroyl *S*,*N*-ketene acetals has been emphasized previously^36,37^. One possibility to introduce further functionalities and to modify the benzyl moiety is the Sonogashira reaction. Since a rigid alkynyl group is introduced on the benzyl substituent^41^, a new dimension of influencing the photophysical properties is opened up.

**Figure 1:**
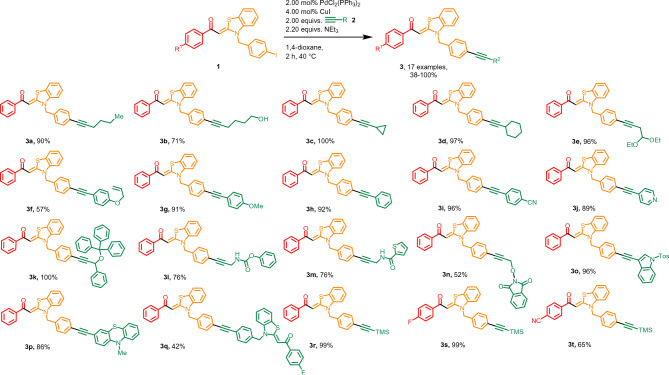
Sonogashira synthesis of alkynylated aroyl-*S*,*N*-ketene acetals **3**. All reactions were performed on a 0.500 mmol scale. The yields given in % present the yields obtained after flash chromatography on silica gel. For experimental details, see SI chpt 4.

An in-depth optimization study (see SI, Table S1) of the Sonogashira reaction step revealed that the use of 2.00 equivalents of (trimethylsilyl)acetylene (TMSA) (**2q**) is sufficient for almost complete conversion, but further reduction of the alkyne equivalents resulted in diminished conversion. Consequently, the use of 2.00 equivalents of TMSA (**2q**), 2.00 mol% PdCl_2_(PPh_3_)_2_, and 4.00 mol% CuI as a catalyst system at 40 °C and a reaction time of 2 h has been identified as ideal reaction conditions. Attempts were made to generate alkynylated aroyl-*S*,*N*-ketene acetals **3** in a one-pot fashion starting from benzothiazolium salts, but due to occurring side reactions isolation of pure compound was considerably hampered. Therefore, a Sonogashira single step approach with *N*-(*p*-iodo)benzyl aroyl-*S*,*N*-ketene acetals **1** and terminal alkynes **2** is employed to synthesize 20 alkynylated aroyl-*S*,*N*-ketene acetals **3** in yields ranging from 38 to 100% (Fig. 1). This synthesis allows the conversion of both aliphatic and aromatic alkynes in moderate to mostly excellent yields. In addition, it is possible to employ a trityl-propargyl ether derivative **2k**, which can be used for concatenating reactions^42^, as well as various amide alkynes **2l–m** or even heterocyclic alkynes such as phthaloylimide (**2n**), indolyl (**2o**), or phenothiazinyl derivatives (**2p**).

**Figure 2:**
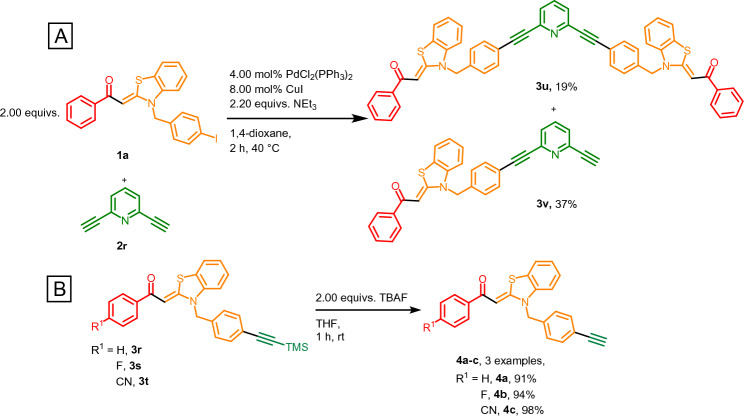
(**A**) Synthesis of pyridine bisalkyne-bridged aroyl-*S*,*N*-ketene acetals **3u** and **3v** and (**B**) deprotection of TMS-protected alkynylated aroyl-*S*,*N*-ketene acetals **4**.

A bis-alkynylated pyridine linker **2r** can be employed to generate a bridged aroyl-*S*,*N*-ketene acetal via a double Sonogashira reaction (Fig. 2A). Here, the alkyne component is used substoichiometrically, and the amount of catalyst has to be increased to allow for the highest conversion possible. However, complete conversion is not achieved; both the avowed pyridine bisalkyne-bridged aroyl-*S*,*N*-ketene acetal dimer **3u** and the monoalkynylation product **3v** are obtained in low yields of 19 and 37%, respectively, and can be separated via column chromatography and several ultrasonification steps. The TMS-protected alkynes **3r**–**3t** can be desilylated using tetra-*N*-butylammonium fluoride at room temperature within 1 h to give the corresponding free alkynes **4a**–**4c** in excellent yields of 91 to 98% (Fig. 2B).

All of the alkynylated aroyl-*S*,*N*-ketene acetals **3** and **4** were studied by absorption and emission spectroscopy. Since the factors influencing the substitution of the aroyl-*S*,*N*-ketene acetal core motif were discussed extensively in previous works, the focus is now on the influence of the variation of the alkynyl moiety on the photophysical properties. The longest wavelength absorption maximum *λ*_*abs,max*_ occurs at wavelengths from 380 to 383 nm, which can be attributed to the aroyl-*S*,*N*-ketene acetal motif^36,37^. Only for the cyano-substituted derivatives **3t** and **4c**, as expected, a bathochromic shift of the longest wavelength absorption maximum to 400 nm can be observed. In addition, for a large number of the alkynylated products, an additional absorption maximum occurring at higher wavenumbers, which is also due to the aroyl-*S*,*N*-ketene acetal motif, appears at 250–260 nm. Furthermore, depending on the corresponding alkyne substituent, more intense absorption maxima are observed in a range from 270 to 310 nm with ε between 30,000 and 60,000 L mol^−1^ cm^−1^ (see SI, chpts. 7 and 8). All alkynylated derivatives **3** and **4** luminesce very weakly blue in ethanol (*Φ*_*f*_ < 0.01) with emission bands between 447 to 454 nm. Only for systems with a pronounced secondary luminescent system as in compound **3p** a larger fluorescence quantum yield *Φ*_*f*_ can be detected.

Based on our previous experience, we assume that the alkynylated derivatives **3** and **4** can be expected to show pronounced solid-state emission. Only for the diethoxybutyne derivative **3e**, which was isolated exclusively as a resin, and for the pyridine bisalkynyl derivatives **3u** and **3v** no solid-state emission can be observed. The absence of solid-state emission of compounds **3u** and **3v** is apparently caused by the pyridine structural motif, since a significant decrease in *Φ*_*f*_ to 0.02 is already observed for the 4-pyridine-alkynylated system **3j**.

**Figure 3:**
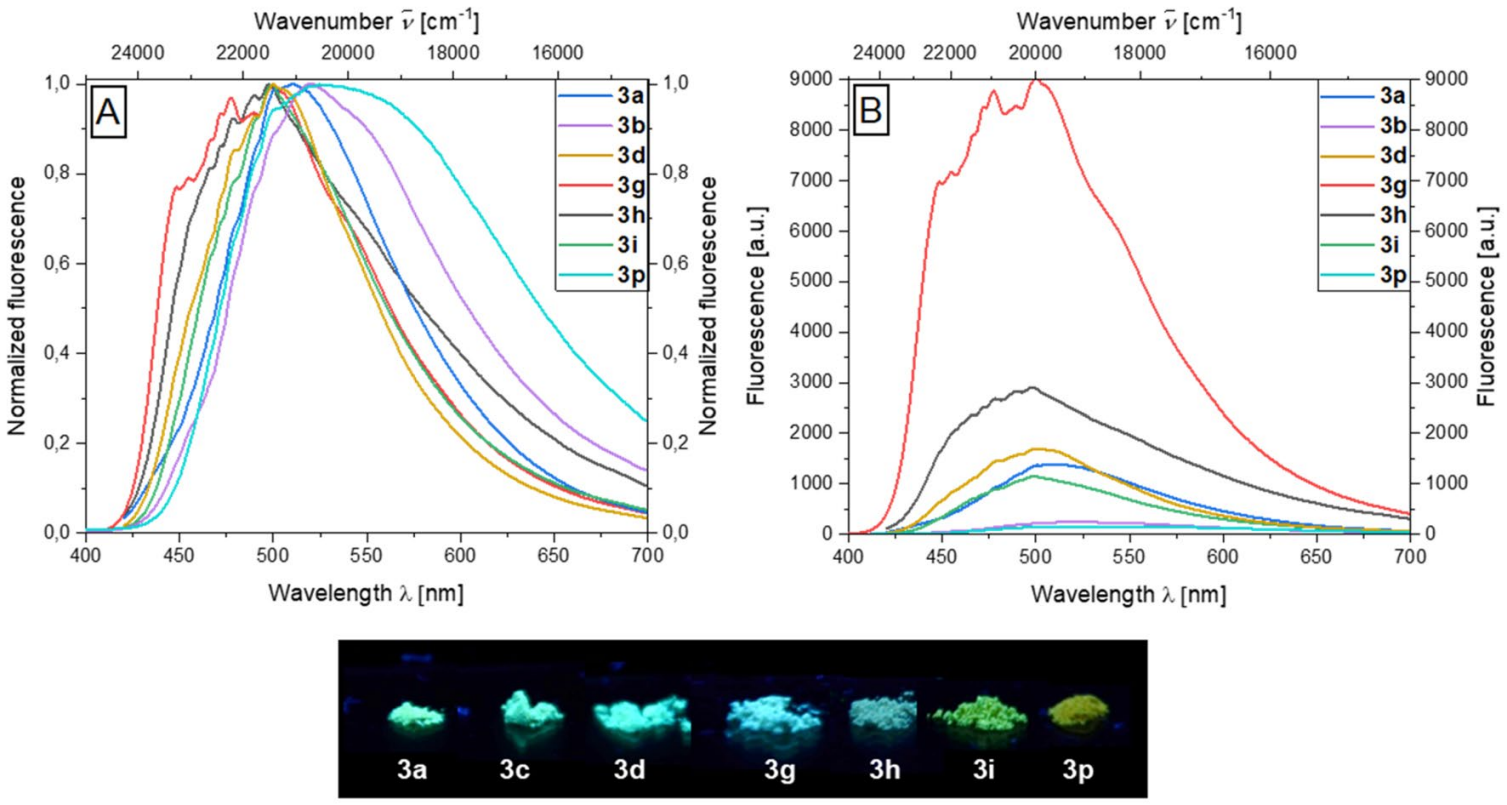
Normalized (**A**) and non-normalized (**B**) solid-state emission spectra and solid-state fluorescence colors of selected alkynylated aroyl-*S*,*N*-ketene acetals **3** (*λ*_*exc*_ = 365 nm). The fluorescence measurements were done at *T* = 298 K with a calibrated photometer and a calibrated fluorometer; the fluorescence was excited at the absorption maximum (*λ*_*exc*_ = *λ*_*abs,max*_).

As most of the compounds are void of a substituent on the aroyl moiety the covered bandwidth of the emission colors is smaller, nevertheless, the benzyl position exerts a smaller but still discernable influence on the emission color. Therefore, a weaker tuning of the emission colors by the benzyl alkynyl moiety is apparent. Thus, donor-substituted systems like **3g** or **3j** show a hypsochromic shift and acceptor-substituted systems like **3i** display a bathochromic shift of the emission maximum to 520 nm. The most pronounced hypsochromic shift is seen for the indole aroyl-*S*,*N*-ketene acetal system **3q** at 460 nm. The most pronounced bathochromic shift with 550 nm occurs in the case of the 1-(allyloxy)-4 ethynylbenzene system **3f**. The majority of the alkynylated systems fluoresce blue to greenish in the solid state (Fig. 3A). For substituents placed at the aroyl moiety, the emission wavelength is naturally affected, especially, cyano substituents cause a bathochromic shift.

With regards to the non-normalized emission, significant differences in emission intensity are noticeable. In particular, the phenyl and methoxyphenylacetylene systems **3g** and **3h** show much higher emission intensity than other systems. This can be attributed to the increased rigidization of the system by the tolane part (Fig. 3B). The deprotected alkyne systems **4** are significantly bathochromically shifted by over 2000 cm^−1^ compared to the TMS-protected systems and appear at an emission maximum of 547 nm for the cyano-substituted compound **4c**.

The fluorescence quantum yields *Φ*_*f*_ of compounds **3** rank in the range from 0.05 to 0.07 for most derivatives, with some selected alkynylated systems as exceptions. For instance, the hexynol system **3b** shows with *Φ*_*f*_ = 0.13 a significantly higher fluorescence quantum yield than the comparable hexynyl system **3a**. This may be due to the polar hydroxy head group of the alkyne. The quantum yields *Φ*_*f*_ of both the TMS-protected and deprotected systems are 0.09 to 0.10, which are also higher than the other alkyne systems. This can be explained by the correlation between solid-state fluorescence quantum yield and substituent size established for the core systems **1**^36,37^. Since the substituents of systems **4** are comparatively small substituents, a similar relationship can be assumed. The combination of phenothiazine and aroyl-*S*,*N*-ketene acetal again leads to a significant decrease in fluorescence quantum yields. The amide systems **3l** and **3m** show with 0.15 and 0.19, respectively, an increased fluorescence quantum yield. The highest fluorescence quantum yield can be observed for the dimeric system **3q** with *Φ*_*f*_ = 0.24.

**Figure 4:**
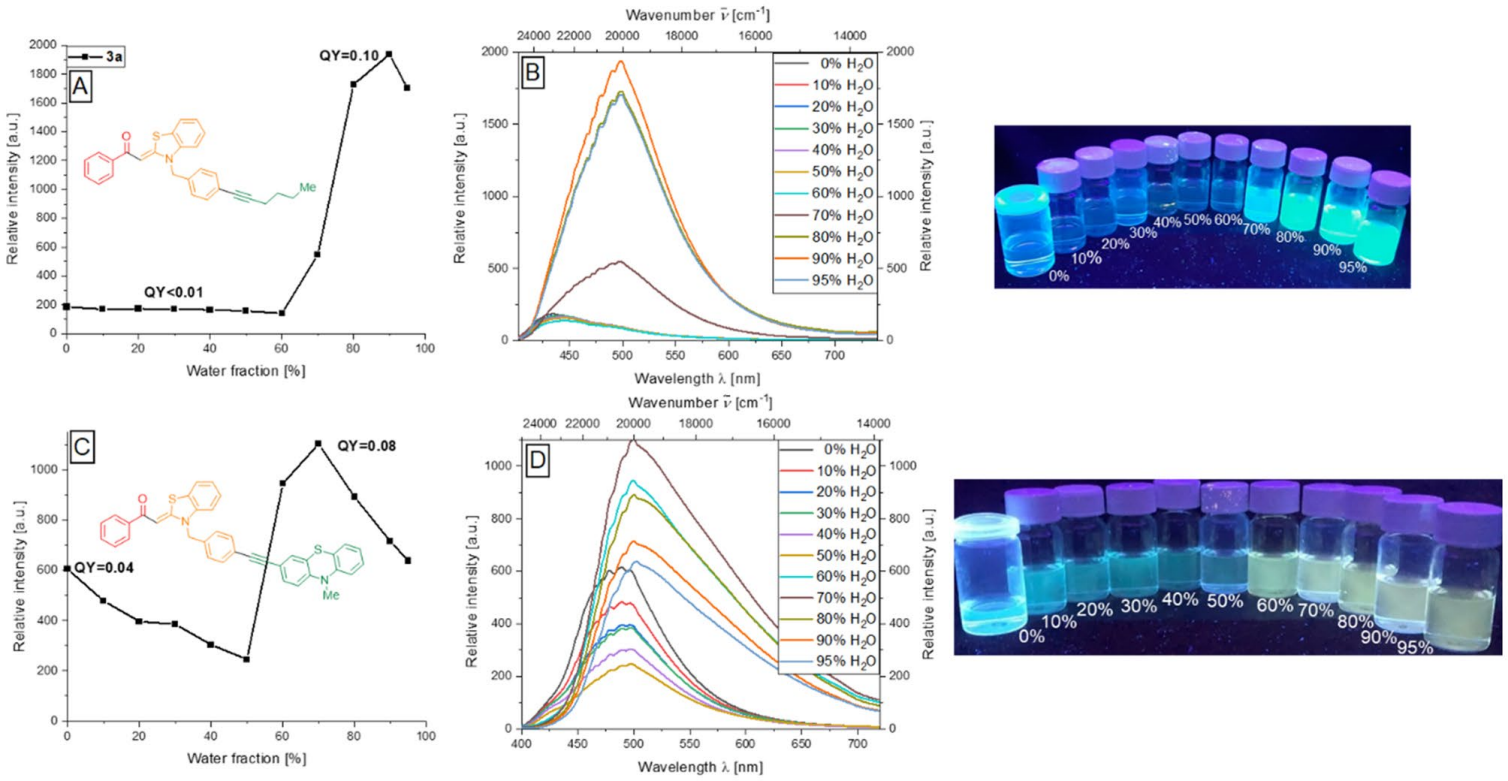
First column: Change in fluorescence intensity of compounds **3a** (**A**), and **3p** (**C**) upon aggregation. Second column: Emission spectra of **3a** (**B**), and **3p** (**D**) in ethanol/water mixtures upon increasing water content (recorded at *T* = 298 K, *c*(**3**) = 10^−7^ m, *λ*_*exc*_ = *λ*_*max,abs*_). Third column: Visualization (photographs) of the AIE features of **3a** (top), and **3p** (bottom) in ethanol/water mixtures of increasing water content upon excitation with a UV-lamp (*c*(**3**) = 10^−7^ m*, λ*_exc_ = 365 nm).

The standardized investigations to assess the aggregation behavior of the alkynylated products **3** and **4** in ethanol–water mixtures with defined concentrations were carried out, thus revealing a *J*-type of aggregation as seen by the considerable red shift of the emission bands in the obtained fluorescence spectra. Since the AIE or AIEE properties are largely defined by the aroyl-*S*,*N*-ketene acetal component, the aggregation behavior of compounds **3** is mostly relatively similar, especially with respect to the observed emission color. Again, the presence of the *N*-benzyl moiety sets the stage for occurrence of AIE as previously shown^36,37^. The aggregation-induced enhanced emission results as a consequence of the more extended alkynylated π-systems that also possess some inherent emission and solution, which enhances upon aggregation. Thus, for the majority of the alkynylated aroyl-*S*,*N*-ketene acetals **3**, only a very weak background fluorescence of the systems with an emission maximum at 447 nm can be observed in solution and at low water fractions. Above a water content of 60%, the formation of aggregates can be observed in both absorption and emission spectroscopy. There is a significant increase in emission intensity in combination with a bathochromic shift of the emission maximum to 500 nm, so that the aggregate solutions luminesce green. The maximum emission intensity is usually reached at a water content of 90%, which corresponds to about a tenfold increase of the initial intensity. A further increase of the water content causes the coagulation-related precipitation of the aggregates with a decrease of the emission intensity. This can be illustrated by the exemplary behavior of the hexynylated system **3a** (Fig. 4A,B). In the case of diethoxybutyne derivative **3e**, pyridine bisalkyne derivatives **3u** and **3v**, and 4-pyridine-alkynylated system **3j**, a much weaker aggregation behavior is observed, and it reflects the trend already seen in the case of solid-state emission. Furthermore, phenothiazine system **3p** represents an exception to the behavior described so far. Thus, by varying the solvent used, a different absorption and emission behavior could be obtained, where the solvatochromic behavior is attributed to the phenothiazine chromophore^43^ (see SI, Fig. S103).

The aggregation behavior of the phenothiazine derivative **3p** also deviates from the AIEE behavior of the alkynylated systems **3** described so far. Thus, the initial fluorescence of the compound with *Φ*_*f*_ = 0.04 is significantly larger than in the case of the previously discussed examples due to the phenylalkynyl phenothiazine typical fluorescence with an emission maximum at 500 nm. Below a water content of 50%, the fluorescence intensity of the aggregate solutions decreases continuously. The increasing polarity exerts an attenuation of the emission of the phenylethynyl phenothiazine system, which is predominantly responsible for the fluorescence in solution. Above a water content of 50%, i.e. much earlier than for other alkynylated systems **3**, the formation of aggregates and the accompanying enhancement of the emission intensity and a bathochromic shift of the emission maximum to 525 nm can be observed, resulting in a green-yellow fluorescence which may also be attributed to an occurring energy transfer from the phenothiazine to the aroyl-*S*,*N*-ketene acetal moiety. Thus, the fluorescence quantum yield *Φ*_*f*_ increases to 0.08 at a water content of 70% (Fig. 4C,D). Fluorescence lifetimes *τ* for all of the investigated compounds were in the magnitude of 1 ns and below in solution, solid-state and in the aggregated state.

### Triazole aroyl-*S*,*N*-ketene acetals

The selective generation of the TMS-protected alkynylated aroyl-*S*,*N*-ketene acetals and the subsequent deprotection to the free alkynes, which was also possible in a one-pot procedure, allows for further exploration of the sequence. The most relevant possibility for expanding the reaction sequence is the concatenation of the CuAAC, which allows access to the structural motifs of triazoles that are also interesting from a photophysical point of view. Furthermore, the application of CuAAC to the substance class of aroyl-*S*,*N*-ketene acetals allows for its use in biological systems, for example, for application as a molecular ruler in FRET processes. Indeed, CuAAC has become the most common synthetic tool for dye functionalization of biological probes and structures^44,45^.

**Figure 5:**
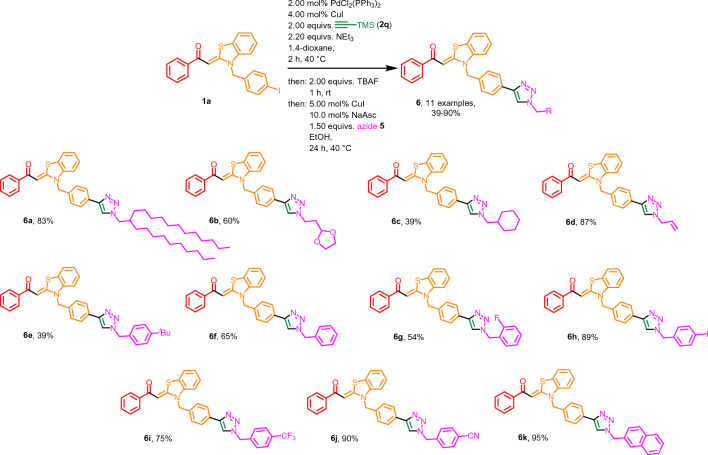
Sonogashira-desilylation-CuAAC one-pot reaction for the generation of triazole aroyl-*S*,*N*-ketene acetals **6**. All reactions were performed on a 0.200 mmol scale. The yields given in % present the yields obtained after flash chromatography on silica gel. For experimental details, see SI chpt 5.

Therefore, a novel Sonogashira-deprotection-CuAAC one-pot sequence of with *N*-(*p*-iodo)benzyl aroyl-*S*,*N*-ketene acetals **1** and TMSA (**2q**), followed by TBAF-mediated desilylation and addition of azides **5** to access the corresponding triazole aroyl-*S*,*N*-ketene acetal systems **6** has been established by a second optimization study for the CuAAC step (see SI, Table S4). The ideal reaction conditions for the CuAAC step are as follows: 5.00 mol% copper(I) iodide, 10.0 mol% sodium ascorbate, 1.50 equivalents of azide **5** at 40 °C for a period of 24 h. In the novel one-pot Sonogashira-desilylation-CuAAC sequence, an additional amount of 5.00 mol% copper(I) iodide has to be added for the CuAAC step. Starting from the iodinated aroyl-*S*,*N*-ketene acetal **1a**, TMSA (**2q**), and azide **5** these optimized reaction conditions are employed to give a substance library of 11 examples of triazole-aroyl-*S*,*N*-ketene acetals **6** in yields ranging between 39 and 90% (Fig. 5). Here, aliphatic azides as well as acceptor and donor substituted benzyl azides are used as substrates **5**.

**Figure 6:**
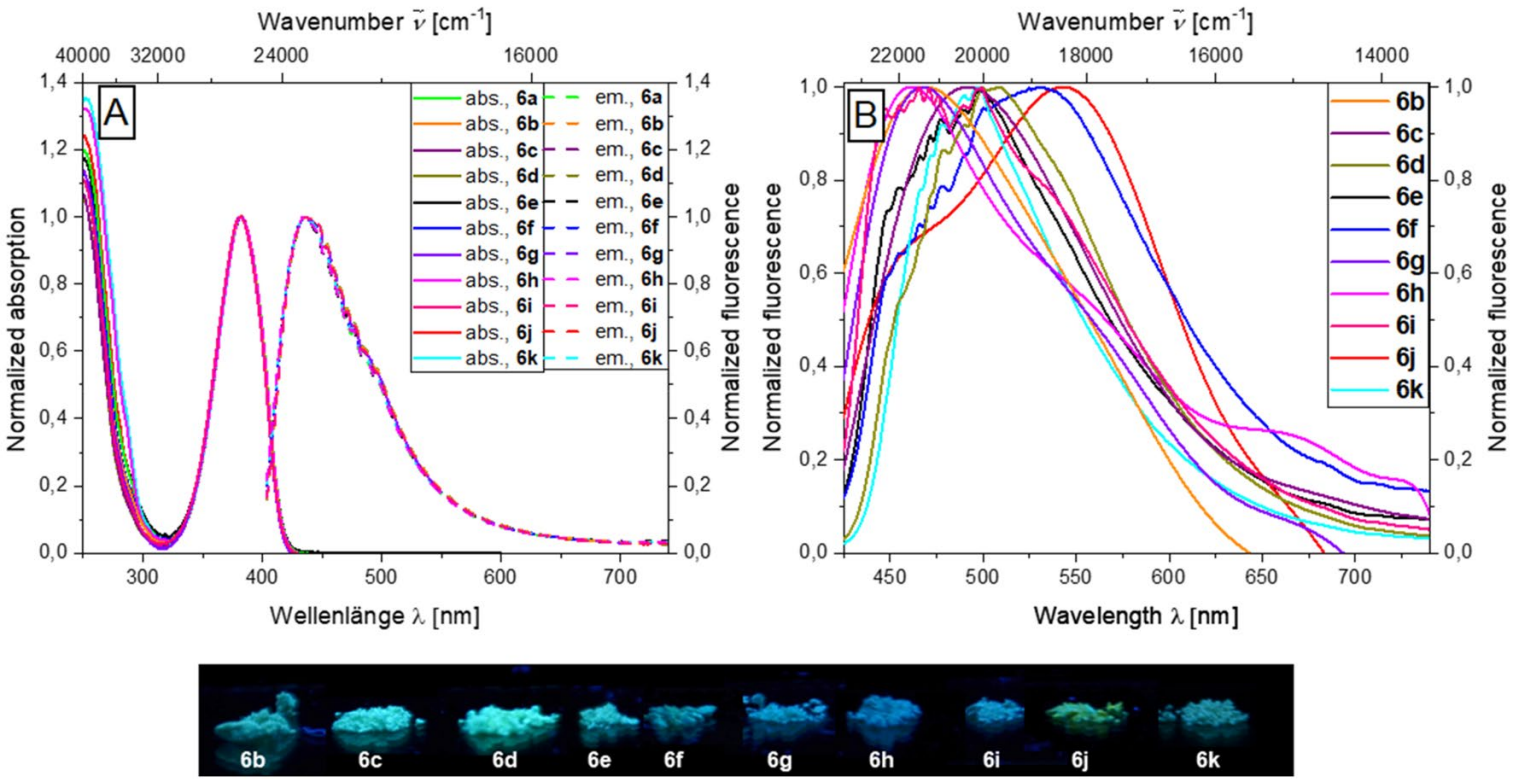
Selected normalized absorption and emission spectra in ethanol (*T* = 298 K, *c*(abs.) = 10^−5^ m, *c*(em.) = 10^−7^ m, *λ*_*exc*_ = *λ*_*max(abs.)*_) (**A**) and normalized (**B**) solid-state emission spectra and solid-state fluorescence colors of selected triazole aroyl-*S*,*N*-ketene acetals **6** (*λ*_*exc*_ = 365 nm). (*λ*_*exc*_ = *λ*_*abs,max*_).

Examination of the photophysical properties of the triazole aroyl-*S*,*N*-ketene acetals **6** reveals a trend comparable to the alkynylated systems **3**. Since the aroyl-*S*,*N*-ketene acetal moiety was kept identical for compounds **6**, an almost identical behavior for all examples of this class of compounds can be observed. The different triazole moieties have almost no influence on the photophysical properties of the chromophores in solution. In contrast to the previously discussed alkynylated aroyl-*S*,*N*-ketene acetals **3**, for the triazole systems **6** only a single absorption maximum between 381 and 383 nm can be detected, which clearly corresponds to the aroyl-*S*,*N*-ketene acetal parent chromophore^36,37^. All triazole aroyl-*S*,*N*-ketene acetals **6** luminesce blue in ethanol. However, the fluorescence quantum yields *Φ*_*f*_ are below the detection limit of 0.01. All compounds also exhibit a very similar fluorescence behavior, the emission maxima are found between 434 and 436 nm with Stokes shifts $\Delta \tilde{v}$*~* around 3150 cm^−1^ (Fig. 6A). The influence on the solid-state fluorescence of the triazole moiety on the consanguine line of chromophores **6** is significantly higher than in solution. The reasons for this can be traced back to the previously postulated importance of the 4-benzyl position^36,37^, which is influenced by the wide range of triazoles. The present triazole systems fluoresce blue to greenish in the solid state with emission maxima between 456 and 530 nm. A second emission maximum can also be observed for selected derivatives. This applies to almost all benzylic triazole systems which is in line with comparable effects occurring for parent aroyl-*S*,*N*-ketene acetals^36,37^. The most significant hypsochromic shift of the emission maximum is found for 4-iodobenzyltriazole derivative **6h** with 465 nm, and the most bathochromically shifted maximum is observed for the cyano-substituted derivative **6j** with 530 nm due to the high acceptor strength. Only for the swallowtail triazole derivative **6a**, which is obtained as a yellow resin, it is not possible to detect solid-state emission (Fig. 6B).

With regard to the solid-state fluorescence quantum yields, *Φ*_*f*_ of the triazoles **6** tends to be significantly higher in comparison to both the aroyl-*S*,*N*-ketene acetal parent systems and to the alkynylated precursors **3**. Indeed, *Φ*_*f*_ ranges from at least 0.14 to 0.16 for all investigated systems, for cyclohexylmethyl triazole **6c**, *o*-fluorobenzyl triazole **6g**, 4-iodobenzyl triazole **6h**, and 4-cyanobenzyl triazole **6j** a slightly increased *Φ*_*f*_ from 0.20 to 0.24 is measured. On the one hand, this suggests that the acceptor substitution on the triazole benzyl residue has a constructive effect with regard to the quantum yield. On the other hand, a distance dependence of *Φ*_*f*_ can be assumed, since a large iodine substituent has an amplifying effect on the quantum yield, although this is not directly involved in the chromophore like it was observed for the aroyl-*S*,*N*-ketene acetal parent structures^36,37^. Naphthylmethyl triazole aroyl-*S*,*N*-ketene acetal **6k** has a remarkably high *Φ*_*f*_ of 0.46 for aroyl-*S*,*N*-ketene acetal. This is due to the fact that the naphthyl moiety is an independent chromophore that contributes to the emission of the system. By far the highest solid-state fluorescence quantum yield is observed for the ethyldioxalane-substituted triazole system **6b** with *Φ*_*f*_ = 0.73 (for details, see SI chpts. 9 and 10).

**Figure 7:**
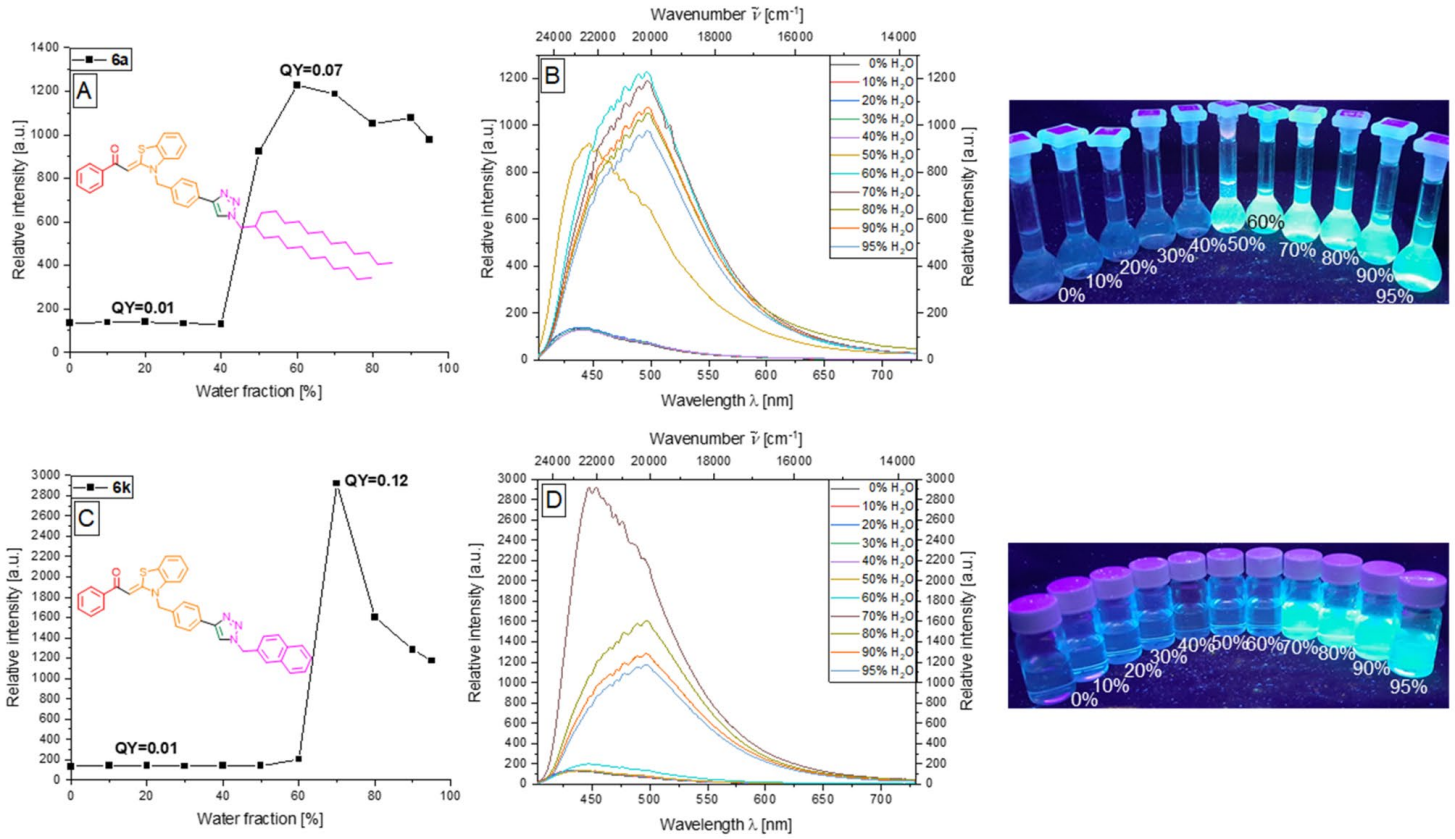
First column: Change in fluorescence intensity of compounds **6a** (**A**), and **6k** (**C**) upon aggregation. Second column: Emission spectra of **6a** (**B**), and **6k** (**D**) in ethanol/water mixtures upon increasing water content (recorded at *T* = 298 K, *c*(**6**) = 10^−7^ m, *λ*_*exc*_ = *λ*_*max,abs*_). Third column: Visualization (photographs) of the AIE features of **6a** (top), and **6 k** (bottom) in ethanol/water mixtures of increasing water content upon excitation with a UV-lamp (*c*(**6**) = 10^−7^ m*, λ*_exc_ = 365 nm).

The investigation of the AIEE properties of the triazole aroyl-*S*,*N*-ketene acetal systems **6** reveals the typical behavior already observed for the alkynylated systems **3** and **4**, which will be explained as examples for the swallowtail triazole derivative **6a** and the naphthylmethyl derivative **6k** (Fig. 7). Prior to aggregate formation, only a very weak background fluorescence at 435 nm with *Φ*_*f*_ = 0.01 can be detected in both cases. As expected, based on previous knowledge, the start of aggregation in the case of the clearly non-polar system **6a** commences already at a water content of 40%. Again, this is due to an increased steric bulk, which diminishes π–π-interactions while enhancing the AIEE effect and simultaneously increasing hydrophobic interactions. The maximum of the emission of the aggregated solution is recorded at a water content of 60%, where *Φ*_*f*_ reaches the maximum value of 0.07. At the beginning of aggregation at a water content of 50%, the emission maximum appears again at 435 nm. Above a water content of 60%, however, the emission maximum occurs at 500 nm, which corresponds to a bathochromic shift of about 3000 cm^−1^ (Fig. 7A,B). Naphthylmethyl derivative **6k** begins to aggregate above a water content of 60%. The maximum emission intensity is reached at a water content of 70% with *λ*_*em,max*_ = 454 nm and *Φ*_*f*_ = 0.12. The higher intensity as well as the higher *Φ*_*f*_ can be attributed to the enlarged π-system, as in solid-state emission. The further increase of the water content leads to a bathochromic shift of the emission maximum to 500 nm and a decrease in emission intensity. This is attributed to the coagulation of aggregates and the enhanced, destructive π–π-interactions (Fig. 7C,D).

## Conclusion

In summary, two highly diversified substance libraries of alkynylated aroyl-*S*,*N*-ketene acetals **3** and triazolyl aroyl-*S*,*N*-ketene acetals **6** were readily synthesized in good to mostly excellent yields. Using an optimized Sonogashira protocol a broad span of alkynes with TMS, heterocyclic, and even amide substituents were reacted with the respective aroyl-*S*,*N*-ketene acetals. Additionally, a follow up deprotection step resulted in cleavage of the protection group to get access to the terminal alkyne derivatives. Combining both reaction steps with a CuAAC gave rise to a novel one-pot procedure that led to the formation of different aliphatic or benzylic 1,2,3-triazole aroyl-*S*,*N*-ketene acetals. The investigation of the photophysical properties of series **3**, **4** and **6** revealed a weak luminescence in ethanol, but pronounced solid-state emission properties. As the main variation occurred on the benzylic position, the emission colors are only influenced to a minor extent, but a very significant effect on the quantum yields was observed, which could be raised up to *Φ*_*f*_ = 0.73. Furthermore, all synthesized compounds exhibit pronounced AIEE properties with a relatively small background fluorescence in the absence of water, but a vastly increased fluorescence by several magnitudes in the presence of an excess of water. This is partly modulated by the nature of the implemented alkyne or triazole, respectively. This work lays the synthetic and methodological foundation for an application of aroyl-*S*,*N*-ketene acetals as markers in biological systems introduced via CuAAC starting from simple and easily accessible aroyl-*S*,*N*-ketene acetals. Future works will address the investigation of biological systems and an optimization of the dye structure for an optimal performance.

## Methods

The used chemicals which have not been synthesized were purchased at Acros Organics BVBA, Alfa Aeser GmbH & Co KG, Fluorochem Ltd., J&K Scientific Ltd., Merck KGaA, Macherey–Nagel GmbH & Co. KG, Sigma-Aldrich Chemie GmbH and VWR and have been used without further purification. The melting points have been measured with Melting Point B-540 of the company Büchi. ^1^H,^13^C and DEPT 135-spectra have been measured at 298 K on an Avance III—300 and an Avance III—600 of the company Bruker. All mass spectrometry experiments have been performed by the department for mass spectrometry of the University of Düsseldorf (HHUCeMSA). EI mass spectra have been measured with Triple-Quadrupol-spectrometer TSQ 7000 of the company Finnigan MAT. MALDI spectra have been measured with a MALDI/TOF UltrafleXtreme of the company Bruker Daltronik. IR spectra were recorded with neat compounds under attenuated total reflection (ATR) with IRAffinity-1 of the company Shimadzu. The elementary analyses have been measured with Perkin Elmer Series II Analyser 2400 or Vario Micro Cube of the company Analysensysteme GmbH. UV/Vis spectra of the dye solutions were measured with a Lambda 19 spectrometer from Perkin Elmer. The emission spectra of the dye solutions and the solid compounds were recorded with a Hitachi F-7000 spectrofluorometer using the emission correction curve provided by the instrument manufacturer. Emission spectra were not corrected for the wavelength-dependent spectral responsivity of the fluorometer. All solution spectra were recorded with dyes dissolved in spectroscopic grade solvents at 298 K using 1 cm-_quat_z cuvettes from Hellma GmbH. The molar extinction coefficients of dye solutions of known dye concentration were determined by five-point regression line.

For aggregation studies, samples of the aroyl-*S*,*N*-ketene acetals were dissolved in various mixtures of organic solvents and water with water contents ranging from 0 to 99%, with ethanol/water mixtures giving the clearest results, which are discussed in detail below. Furthermore, solvent mixtures of cyclohexane and dichloromethane were also tested, but no aggregate formation was observed. A stock solution of the respective chromophore was prepared in ethanol. A defined amount of this stock solution was taken and the corresponding amount of ethanol was first placed in a 10 mL volumetric flask before the corresponding amount of water was added. To exclude aging and coagulation processes of the formed aggregates as far as possible, all solutions were first treated in an ultrasonic bath for 5 min before the emission and absorption spectra were recorded starting with the solutions of the highest water content. Selected aggregate solutions were stored for investigation of aggregate stability over time for subsequent measurements at defined time points. This general measurement protocol was applied to all aggregate studies to ensure comparability of measurements. For the photographs of the aggregate solutions, the corresponding measurement solutions were used directly.

### Supplementary Information

Supplementary Information.

## Data Availability

All data generated or analyzed during this study are included in this published article [and its supplementary information files. Experimental details of all synthesized derivatives and the respective spectroscopic and analytic data, spectra (^1^H and ^13^C NMR spectra, absorption and emission spectra, AIE-titration experiments) and photographs, see the accompanying Supporting Information. The datasets used and/or analyzed during the current study are available from the corresponding author on reasonable request.
