# Supplementary material for: Alkynylated and triazole-linked aroyl-S,N-ketene acetals: one-pot synthesis of solid-state emissive dyes with aggregation-induced enhanced emission characteristics
Source: Sci Rep. 2023 Sep 1;13:14399. doi: 10.1038/s41598-023-41146-w (PMC10474010; doi:10.1038/s41598-023-41146-w)
Supplement: Supplementary file 1 — Supplementary Information. [file 41598_2023_41146_MOESM1_ESM.pdf]

Alkynylated And Triazole-linked Aroyl-*S,N*-ketene Acetals – One-pot Synthesis of Solid-State Emissive Dyes with Aggregation-Induced Enhanced Emission Characteristics

Lukas Biesen, Yannic Hartmann, and Thomas J. J. Müller*

Institut für Organische Chemie und Makromolekulare Chemie, Heinrich-Heine-Universität Düsseldorf, Universitätsstraße 1, D-40225 Düsseldorf, Germany.

E-Mail: ThomasJJ.Mueller@uni-duesseldorf.de

Fax: (+)49 (0)211-8114324

Table of contents

1 General considerations 5

2 Overview of synthesized derivatives 7

2.1 Optimization study for the generation of alkynylated aroyl-*S,N*-ketene acetals 3 7

2.2 Overview of synthesized alkynylated aroyl-*S,N*-ketene acetals 3 9

2.3 Optimization study for the generation of triazole aroyl-*S,N*-ketene acetals 6 13

2.4 Overview of synthesized triazole aroyl-*S,N*-ketene acetals 6 15

3 Starting material 17

3.1 Benzothiazolium salt 17

3-(4-Iodobenzyl)-2-methylbenzo[*d*]thiazol-3-iumbromide[3],[4] 17

3.2 Synthesis and analytical data of aroyl-*S,N*-ketene acetals 1 18

3.2.1 General procedure I (GPI) for the synthesis of aroyl-*S*,*N*-ketene acetals **1**[3],[4] 18

(*Z*)-2-(3-(4-Iodobenzyl)benzo[*d*]thiazol-2(3*H*)-ylidene)-1-phenylethan-1-one (**1a**)[4] 19

(*Z*)-2-(3-(4-Iodobenzyl)benzo[*d*]thiazol-2(3*H*)-ylidene)-1-(4-fluoro)phenylethan-1-one (**1b**)[4] . 20

(*Z*)-4-(2-(3-(4-Iodobenzyl)benzo[*d*]thiazol-2(3*H*)-ylidene)acetyl)benzonitrile (**1c**)[4] 21

4 Synthesis and analytical data of alkynylated aroyl-*S*,*N*-ketene acetals 3 22

4.1 General procedure II (GPII) for the synthesis of alkynylated aroyl-*S,N*-ketene acetals 3 22

4.1.1 Spectroscopic data 27

(Z)-2-(3-(4-(Hex-1-yn-1-yl)benzyl)benzo[*d*]thiazol-2(3*H*)-ylidene)-1-phenylethan-1-one (**3a**) . 27

(*Z*)-2-(3-(4-(6-Hydroxyhex-1-yn-1-yl)benzyl)benzo[*d*]thiazol-2(3*H*)-ylidene)-1-phenylethan-1-one (**3b**) 28

(*Z*)-2-(3-(4-(Cyclopropylethynyl)benzyl)benzo[*d*]thiazol-2(3*H*)-ylidene)-1-phenylethan-1-one (**3c**) 29

(*Z*)-2-(3-(4-(4,4-Diethoxybut-1-yn-1-yl)benzyl)benzo[*d*]thiazol-2(3*H*)-ylidene)-1-phenylethan-1-one (**3e**) 31
(*Z*)-2-(3-(4-((4-(Allyloxy)phenyl)ethynyl)benzyl)benzo[*d*]thiazol-2(3*H*)-ylidene)-1-phenylethan-1-one (**3f**) 32
(Z)-2-(3-(4-((4-Methoxyphenyl)ethynyl)benzyl)benzo[*d*]thiazol-2(3*H*)-ylidene)-1-phenylethan-1-one (**3g**) 33
(*Z*)-1-Phenyl-2-(3-(4-(phenylethynyl)benzyl)benzo[*d*]thiazol-2(3*H*)-ylidene)ethan-1-one (5h). 34
(*Z*)-4-((4-((2-(2-Oxo-2-phenylethylidene)benzo[*d*]thiazol-3(2*H*)-yl)methyl)phenyl)ethynyl)benzonitrile (**3i**) 35
(*Z*)-1-Phenyl-2-(3-(4-(pyridine-4-ylethynyl)benzyl)benzo[*d*]thiazol-2(3*H*)-ylidene)ethan-1-one (**3j**) 36
(*Z*)-1-Phenyl-2-(3-(4-(3-phenyl-3-(trityloxy)prop-1-yn-1-yl)benzyl)benzo[*d*]thiazol-2(3*H*)-ylidene)ethan-1-one (**3k**) 37
(*Z*)-*N*-(3-(4-((2-(2-Oxo-2-phenylethylidene)benzo[*d*]thiazol-3(2*H*)-yl)methyl)phenyl)prop-2-yn-1-yl)-2-phenoxyacetamide (**3l**) 38
(*Z*)-*N*-(3-(4-((2-(2-Oxo-2-phenylethylidene)benzo[*d*]thiazol-3(2*H*)-yl)methyl)phenyl)prop-2-yn-1-yl)thiophene-2-carboxamide (**3m**) 39
(Z)-2-((3-(4-((2-(2-Oxo-2-phenylethylidene)benzo[d]thiazol-3(2H)-yl)methyl)phenyl)prop-2-yn-1-yl)oxy)isoindolin-1,3-dione (**3n**) 40
(*Z*)-2-(3-(4-((5-Chloro-1-tosyl-1*H*-indol-3-yl)ethinyl)benzyl)benzo[*d*]thiazol-2(3*H*)-ylidene)-1-phenylethan-1-one (**3o**) 41
(*Z*)-2-(3-(4-((10-Methyl-10*H*-phenothiazin-3-yl)ethynyl)benzyl)benzo[*d*]thiazol-2(3*H*)-ylidene)-1-phenylethan-1-one (**3p**) 42
(*Z*)-1-(4-Fluorophenyl)-2-(3-(4-((4-(((*Z*)-2-(2-oxo-2-phenylethylidene)benzo[*d*]thiazol-3(2*H*)-yl)methyl)phenyl)ethynyl)benzyl)benzo[*d*]thiazol-2(3*H*)-ylidene)ethan-1-one (**3q**) 43
(*Z*)-1-Phenyl-2-(3-(4-((trimethylsilyl)ethynyl)benzyl)benzo[*d*]thiazol-2(3*H*)-ylidenr)ethan-1-one (**3r**) 44
(*Z*)-1-(4-Fluorophenyl)-2-(3-(4-((trimethylsilyl)ethynyl)benzyl)benzo[*d*]thiazol-2(3*H*)-ylidene)ethan-1-one (**3s**) 45
(*Z*)-4-(2-(3-(4-((Trimethylsilyl)ethynyl)benzyl)benzo[*d*]thiazol-2(3*H*)-ylidene)acetyl)benzonitrile (**3t**) 47
(2*Z*,2'*Z*)-2,2'-((((Pyridine-2,6-diylbis(ethyn-2,1-diyl))bis(4,1-phenylene))bis(methylene))bis(benzo[*d*]thiazol-3(3*H*)-yl-2(3*H*)-yliden))bis(1-phenylethan-1-one) (**3u**) 48
(*Z*)-2-(3-(4-((6-Ethynylpyridine-2-yl)ethinyl)benzyl)benzo[*d*]thiazol-2(3*H*)-ylidene)-1-phenylethan-1-one (**3v**) 49
4.2 General procedure III (GPIII) for the deprotection of alkynylated aroyl-*S,N*-ketene acetals 4 50
4.2.1 Spectroscopic data 51
(*Z*)-2-(3-(4-Ethynylbenzyl)benzo[*d*]thiazol-2(3*H*)-ylidene)-1-phenylethan-1-one (**4a**) 51

(*Z*)-2-(3-(4-Ethynylbenzyl)benzo[*d*]thiazol-2(3*H*)-ylidene)-1-(4-fluorophenyl)ethan-1-one (**4b**) 52
(*Z*)-4-(2-(3-(4-Ethynylbenzyl)benzo[*d*]thiazol-2(3*H*)-ylidene)acetyl)benzonitrile (**4c**) 53

5 Synthesis and analytical data of triazole aroyl-*S*,*N*-ketene acetals 6 54

5.1 General procedure IV (GPIV) for the synthesis of triazole aroyl-*S,N*-ketene acetals 6 54

5.1.1 Spectroscopic data 57

(*Z*)-2-(3-(4-(1-(2-Decyltetradecyl)-1*H*-1,2,3-triazol-4-yl)benzyl)benzo[*d*]thiazol-2(3*H*)-ylidene)-1-phenylethan-1-one (**6a**) 57

(*Z*)-2-(3-(4-(1-(2-(1,3-Dioxolan-2-yl)ethyl)-1*H*-1,2,3-triazol-4-yl)benzyl)benzo[*d*]thiazol-2(3*H*)-ylidene)-1-phenylethan-1-one (**6b**) 58

(*Z*)-2-(3-(4-(1-(Cyclohexylmethyl)-1*H*-1,2,3-triazol-4-yl)benzyl)benzo[*d*]thiazol-2(3*H*)-ylidene)-1-phenylethan-1-one (**6c**) 59

(*Z*)-2-(3-(4-(1-Allyl-1*H*-1,2,3-triazol-4-yl)benzyl)benzo[*d*]thiazol-2(3*H*)-ylidene)-1-phenylethan-1-one (**6d**) 60

(*Z*)-2-(3-(4-(1-(4-(*tert*-Butyl)benzyl)-1*H*-1,2,3-triazol-4-yl)benzyl)benzo[*d*]thiazol-2(3*H*)-ylidene)-1-phenylethan-1-one (**6e**) 61

(*Z*)-2-(3-(4-(1-Benzyl-1*H*-1,2,3-triazol-4-yl)benzyl)benzo[*d*]thiazol-2(3*H*)-ylidene)-1-phenylethan-1-one (**6f**) 62

(*Z*)-2-(3-(4-(1-(2-Fluorobenzyl)-1*H*-1,2,3-triazol-4-yl)benzyl)benzo[*d*]thiazol-2(3*H*)-ylidene)-1-phenylethan-1-one (**6g**) 63

(*Z*)-2-(3-(4-(1-(4-Iodobenzyl)-1*H*-1,2,3-triazol-4-yl)benzyl)benzo[*d*]thiazol-2(3*H*)-ylidene)-1-phenylethan-1-one (**6h**) 64

(*Z*)-1-Phenyl-2-(3-(4-(1-(4-(trifluoromethyl)benzyl)-1*H*-1,2,3-triazol-4-yl)benzyl)benzo[*d*]thiazol-2(3*H*)-ylidene)ethan-1-one (**6i**) 65

(*Z*)-4-((4-(4-((2-(2-Oxo-2-phenylethylidene)benzo[*d*]thiazol-3(2*H*)-yl)methyl)phenyl)-1*H*-1,2,3-triazol-1-yl)methyl)benzonitrile (**6j**) 66

(*Z*)-2-(3-(4-(1-(Naphthalene-2-ylmethyl)-1*H*-1,2,3-triazol-4-yl)benzyl)benzo[*d*]thiazol-2(3*H*)-ylidene)-1-phenylethan-1-one (**6k**) 67

6 NMR Spectra 68

6.1 NMR spectra of alkynylated aroyl-*S*,*N*-ketene acetals 3 68

6.2 NMR spectra of deprotected alkynylated aroyl-*S*,*N*-ketene acetals 4 90

6.3 NMR spectra of triazole aroyl-*S*,*N*-ketene acetals 6 93

7 Overview of photophysical properties of alkynylated aroyl-*S*,*N*-ketene acetals 3 and 4 104

8 Absorption and emission spectra 108

8.1 Absorption and emission spectra of alkynylated aroyl-*S,N*-ketene acetals 3 and 4...108

9 Overview of photophysical properties of triazole aroyl-*S*,*N*-ketene acetals 6......139

10 Absorption and emission spectra 141

10.1 Absorption and emission spectra of triazole aroyl-*S,N*-ketene acetals 6 141

11 Literature..155

1 General considerations

Reactions were carried out in dried and sintered Schlenk tubes or round bottom flasks under nitrogen atmosphere. Solvents were dried by a solvent purification system *MB-SPS-800* of the company *MBraun Inertgas-Systeme GmbH*.

The used chemicals which have not been synthesized were purchased at *Acros Organics BVBA*, *Alfa Aeser GmbH & Co KG*, *Fluorochem Ltd.*, *J&K Scientific Ltd.*, *Merck KGaA*, *Macherey-Nagel GmbH & Co. KG*, *Sigma-Aldrich Chemie GmbH* and *VWR* and have been used without further purification. The solvents ethanol and tetrahydrofuran (THF) (spectroscopic grade) were purchased from *Merck* and *Sigma-Aldrich* (Germany), respectively. Milli-Q-water was obtained from a *Millipore water purification system*. Carboxylated 8 µm-sized polystyrene particles (PSP) were obtained from *Kisker Biotech GmbH* (Germany).

Further purification of the compounds was performed by flash column chromatography (silica gel M60 pore size 0.040-0.063 nm) of the company *Macherey-Nagel*. The crude product was adsorbed on Celite®545 of the company *Carl Roth GmbH*, placed on the suspended silica gel and purified with a positive pressure of 2 bar. Distilled solvent mixtures of *n*-hexane, acetone and methanol have been used as eluents.

The control of reaction progress was done via thin layer chromatography (TLC) with silica coated aluminium plates F_{254}, of the company *Macherey-Nagel GmbH & Co. KG.*

The melting points have been measured with *Melting Point B-540* of the company *Büchi* according to the protocol of *Kofler*.[1]

^{1}H,^{13}C and DEPT 135-spectra have been measured at 298 K on an *Avance III - 300* and an *Avance III - 600* of the company *Bruker*. Chemical shifts in the ^{1}H and ^{13}C NMR are reported in ppm relative to deuterated solvents such as acetone-d_6 (δ_H 2.05, δ_C 29.84, δ_C 206.26) with CS_2 (δ_C 192.28) and DMSO-d_6 (δ_H 2.50, δ_C 39.51).[2] The multiplicity is abbreviated as followed: s = singlet; d = doublet; t = triplet; td = triplet of doublet; dd = doublet of doublet; dt = doublet of triplet, dq = doublet of quartet; pd = quintet of doublet m = multiplet. The assignment of primary carbon centers (CH), secondary carbon centers (CH_2), tertiary carbon centers (CH_3) and quaternary carbon centers (C_{quat}) were made by using DEPT-135 spectra.

All mass spectrometry experiments have been performed by the department for mass spectrometry of the University of Düsseldorf (HHUCeMSA). EI mass spectra have been measured with Triple-Quadrupol-spectrometer *TSQ 7000* of the company *Finnigan MAT*. MALDI spectra have been measured with a *MALDI/TOF UltrafleXtreme* of the company *Bruker Daltronik*.

IR spectra were recorded with neat compounds under attenuated total reflection (ATR) with *IRAffinity-1* of the company *Shimadzu* and the intensities were characterized as strong (s), middle (m) and weak (w).

The elementary analyses have been measured with *Perkin Elmer Series II Analyser 2400* or *Vario Micro Cube* of the company *Analysensysteme GmbH* at the microanalytical laboratory of the institute for Pharmaceutical and Medicinal Chemistry of the University Düsseldorf.

UV/Vis spectra of the dye solutions were measured with a *Lambda 19* spectrometer from *Perkin Elmer*. The emission spectra of the dye solutions and the solid compounds were recorded with a *Hitachi F-7000* spectrofluorometer using the emission correction curve provided by the instrument manufacturer. Emission spectra were not corrected for the wavelength-dependent spectral responsivity of the fluorometer. All solution spectra were recorded with dyes dissolved in spectroscopic grade solvents at 298 K using 1 cm-$_{quat}$z cuvettes from *Hellma GmbH*. The molar extinction coefficients of dye solutions of known dye concentration were determined by five-point regression line.

2 Overview of synthesized derivatives

2.1 Optimization study for the generation of alkynylated aroyl-*S,N*-ketene acetals 3

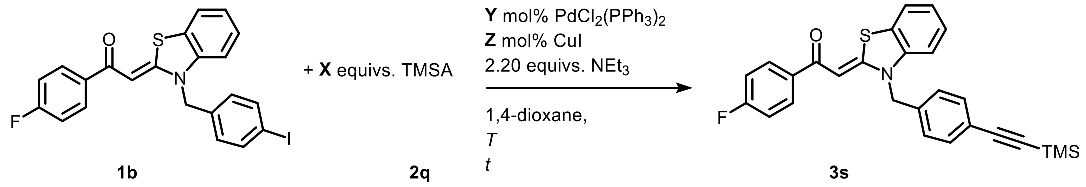

Figure S1. Test reaction for the optimization study for the alkynylation step of **3s**.

Table S1. Optimization study for the aroylation reaction step of **3s**.

entry	TMSA [equivs.]	$PdCl_2(PPh_3)_2$ [mol%]	CuI [mol%]	temperature *T* [°C]	time *t* [h]	yield of product [%]
1	2.50	2.00	4.00	100	16	44
2	2.50	2.00	4.00	80	16	88
3	2.50	2.00	4.00	60	16	89
4	2.50	2.00	4.00	40	16	98
5	2.50	1.00	2.00	40	16	53
6	2.50	2.00	4.00	40	8	98
7	2.50	2.00	4.00	40	6	99
8	2.50	2.00	4.00	40	4	99
9	2.50	2.00	4.00	40	2	99
10	2.00	2.00	4.00	40	2	99
11	1.50	2.00	4.00	40	2	78

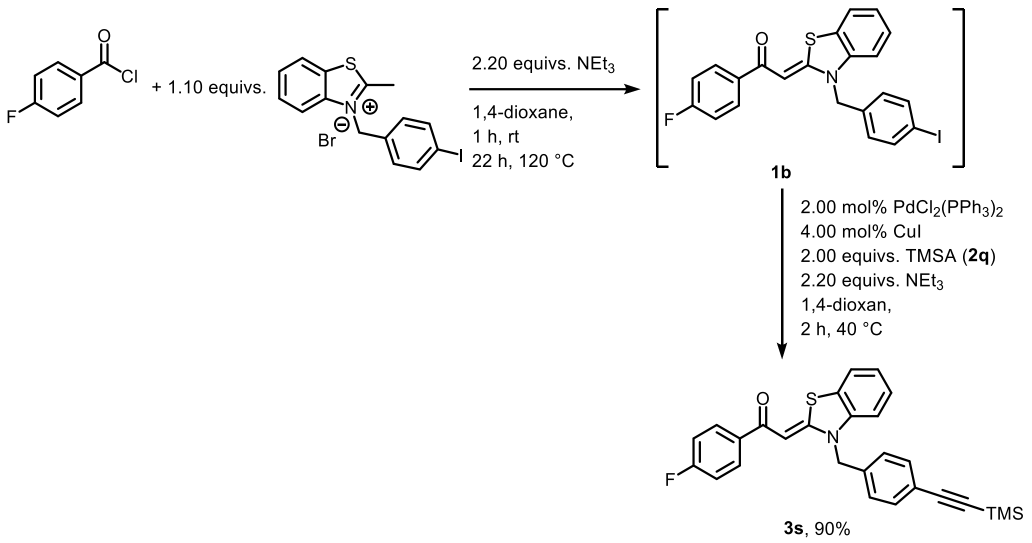

Figure S2. Synthesis of alkynylated aroyl-*S*,*N*-ketene acetal **3s** using the optimized reaction conditions in a one-pot process.

2.2 Overview of synthesized alkynylated aroyl-*S,N*-ketene acetals 3

Table S2. Synthesized alkynylated aroyl-*S,N*-ketene acetals **3**.

entry	R^1	alkyne **2**	product **3**
1	H	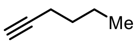 **2a**	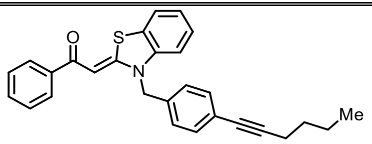 **3a**, 90%
2	H	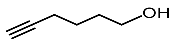 **2b**	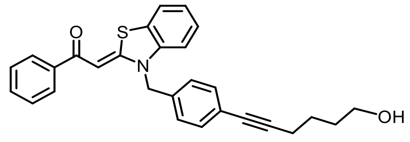 **3b**, 71%
3	H	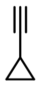 **2c**	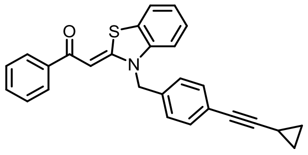 **3c**, 100%
4	H	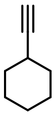 **2d**	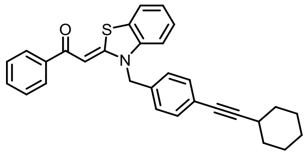 **3d**, 97%
5	H	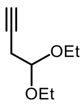 **2e**	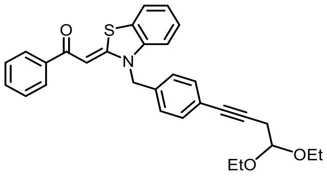 **3e**, 96%
6	H	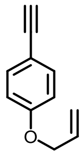 **2f**	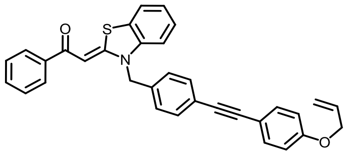 **3f**, 57%
7	H	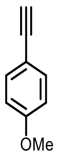 **2g**	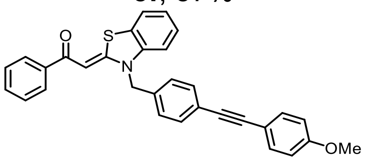 **3g**, 91%
8[b]	H	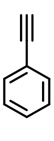 **2h**	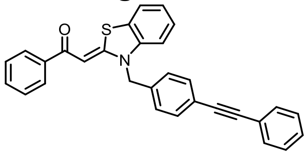 **3h**, 92%

Table S2 continued.

entry	R^1	alkyne **2**	product **3**
9	H	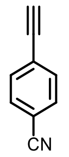 **2i**	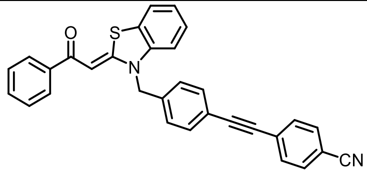 **3i**, 95%
10	H	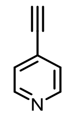 **2j**	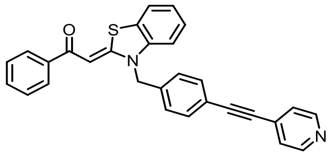 **3j**, 89%
11	H	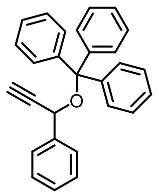 **2k**	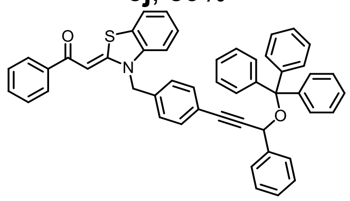 **3k**, 100%
12	H	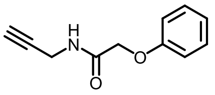 **2l**	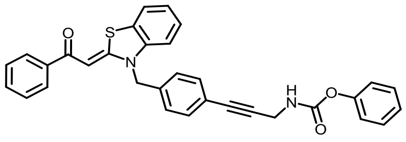 **3l**, 76%
13	H	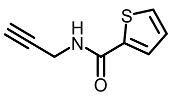 **2m**	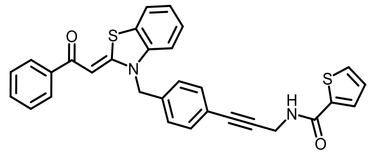 **3m**, 52%
14	H	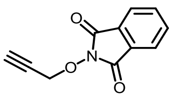 **2n**	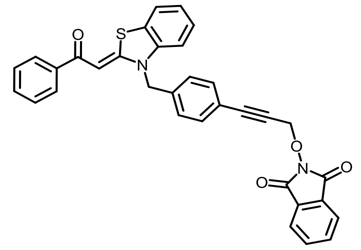 **3n**, 52%
15	H	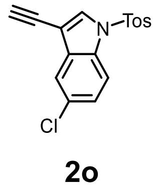 **2o**	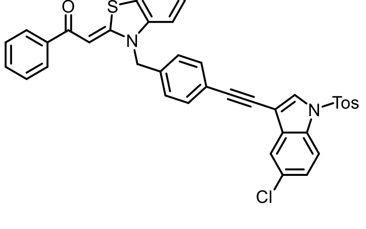 **3o**, 96%

Table S2 continued.

entry	R^1	alkyne **2**	product **3**
16	H	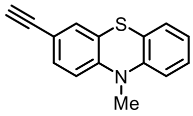<br>**2p**	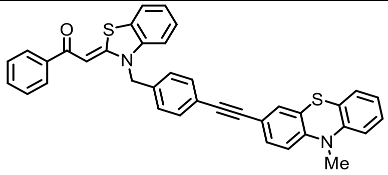<br>**3p**, 86%
17	H	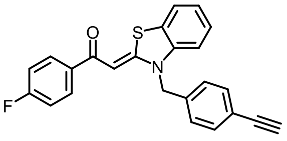<br>**2s**	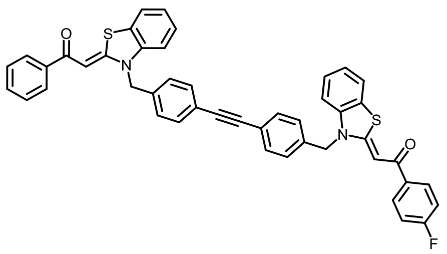<br>**3q**, 42%
18	H	 **2q**	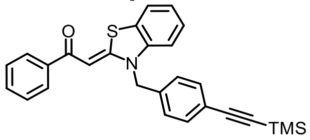<br>**3r**, 99%
19	F	 **2q**	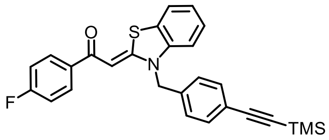<br>**3s**, 99%
20	CN	 **2q**	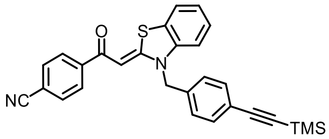<br>**3t**, 65%
21	H	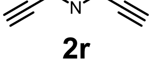<br>**2r**	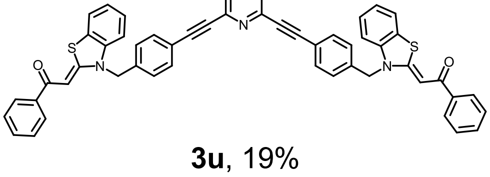<br>**3u**, 19%<br>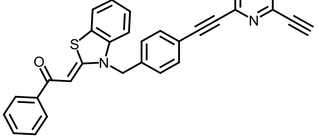<br>**3v**, 37%

Table S3. Synthesized deprotected alkynylated aroyl-*S,N*-ketene acetals **4**.

entry	alkyne **3**	product **4**
1	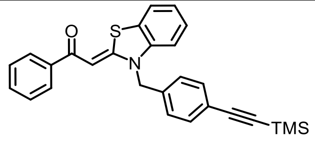<br>**3r**	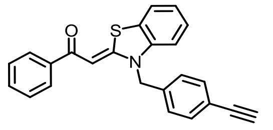<br>**4a**, 91%
2	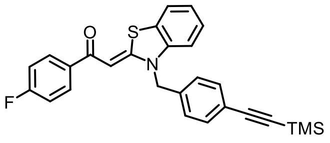<br>**3s**	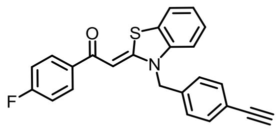<br>**4b**, 94%
3	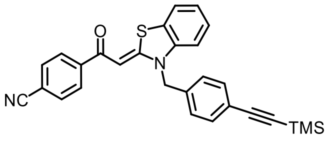<br>**3t**	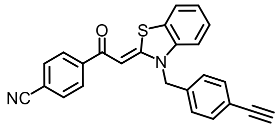<br>**4c**, 98%

2.3 Optimization study for the generation of triazole aroyl-*S,N*-ketene acetals 6

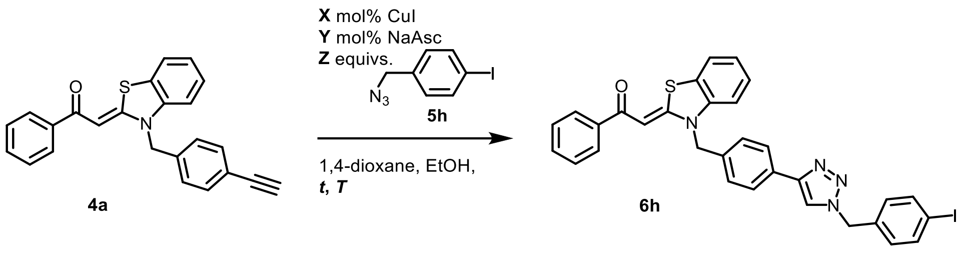

Figure S3. Test reaction for the optimization study for the Click reaction step of **6h**.

Table S4. Optimization study for the click reaction step of **6h**.

entry	CuI [mol%]	NaAsc [mol%]	azide **4h** [equivs.]	temperature *T* [°C]	time *t* [h]	yield of product [%]
1	4.00	8.00	1.00	RT	24	48
2	5.00	10.0	1.00	RT	24	62
3	7.00	14.0	1.00	RT	24	59
4	5.00	10.0	1.50	RT	24	68
5	5.00	10.0	2.00	RT	24	66
6	5.00	10.0	1.50	40	24	89
7	5.00	10.0	1.50	60	24	81
8	5.00	10.0	1.50	80	24	32
8	5.00	10.0	1.50	80	8	58
9	5.00	10.0	1.50	40	6	49

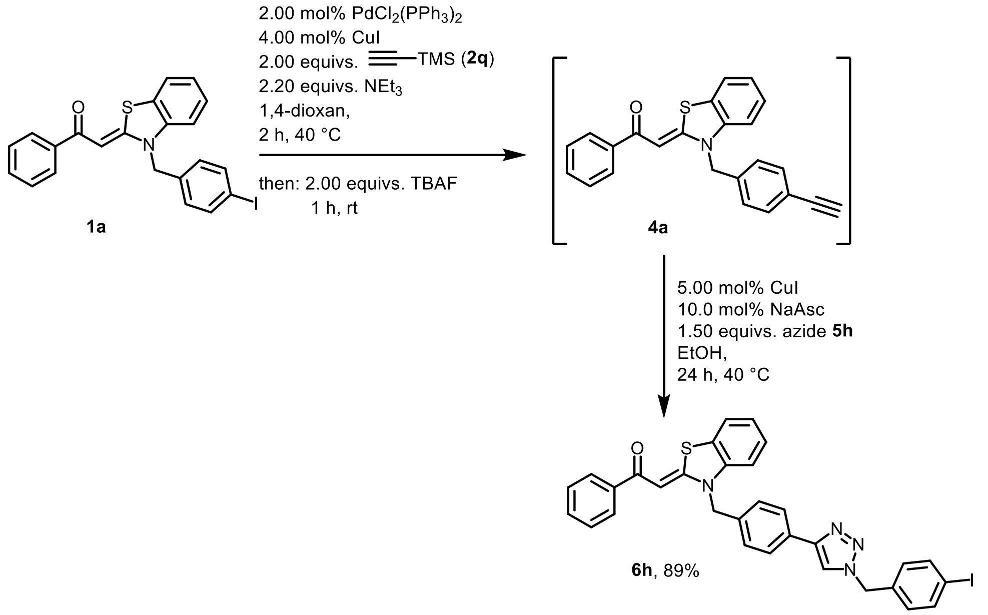

Figure S4. Synthesis of triazole aroyl-*S*,*N*-ketene acetal **6h** using the optimized reaction conditions in a one-pot process.

2.4 Overview of synthesized triazole aroyl-*S,N*-ketene acetals 6

Table S5. Synthesized triazole aroyl-*S,N*-ketene acetals **6**.

entry	azide **5**	product **6**
1	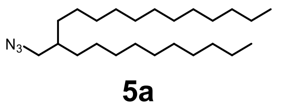 **5a**	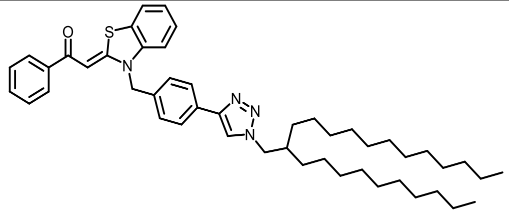 **6a**, 83%
2	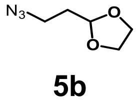 **5b**	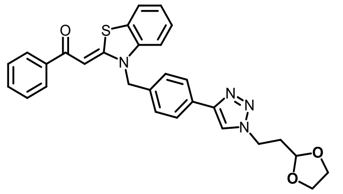 **6b**, 60%
3	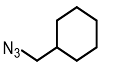 **5c**	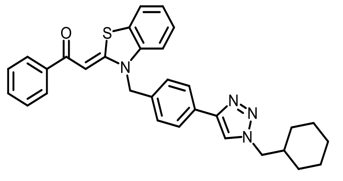 **6c**, 39%
4	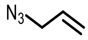 **5d**	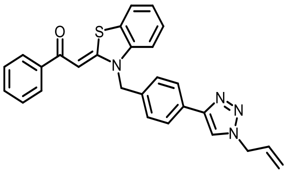 **6d**, 87%
5	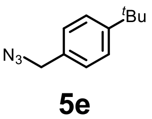 **5e**	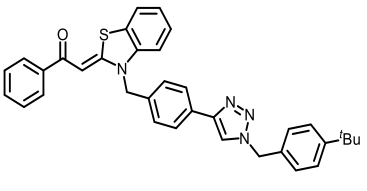 **6e**, 32%
6	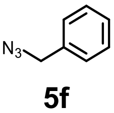 **5f**	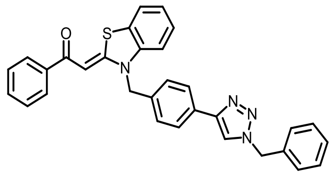 **6f**, 65%

Table S5 continued.

entry	azide **5**	product **6**
7	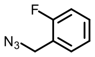 **5g**	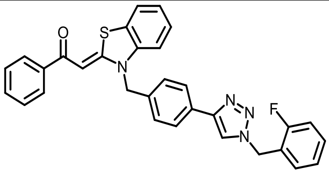 **6g**, 54%
8	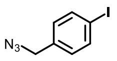 **5h**	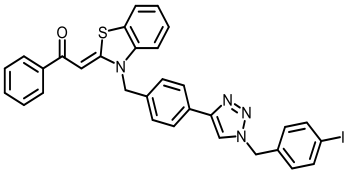 **6h**, 89%
9	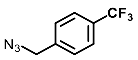 **5i**	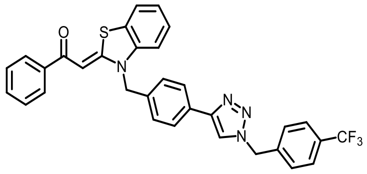 **6i**, 75%
10	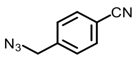 **5j**	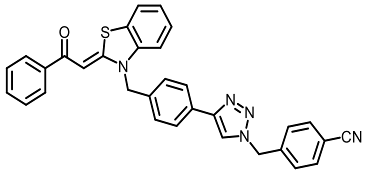 **6j**, 90%
11	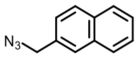 **5k**	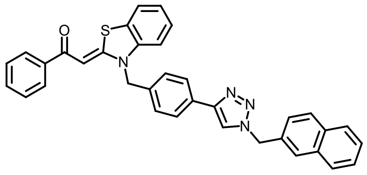 **6k**, 95%

3 Starting material

3.1 Benzothiazolium salt

3-(4-Iodobenzyl)-2-methylbenzo[*d*]thiazol-3-iumbromide[3],[4]

2-Methylbenzothiazole (1.00 g, 6.67 mmol) and 4-iodo benzylbromide (2.37 g, 8.00 mmol) were placed in a round-bottom flask. The reaction mixture was stirred at 75 °C for 20 h, until the solution was completely hardened. The formed solid was filtrated via a Buechner funnel, washed with diethyl ether and dried under vacuo. The synthesis yielded 3.07 g (5.89 mmol, 88%) of the desired product as a pink solid.

Mp: 211 °C.

R_f (*n*-hexane/acetone 1:1): 0.10.

^{1}H NMR (300 MHz, DMSO-d$_6$): δ 3.90 (s, 3 H), 6.07 (s, 2 H), 7.13-7.17 (m, 2 H), 7.73-7.86 (m, 4 H), 8.17-8.21 (m, 1 H), 8.51-8.54 (m, 1 H).

^{13}C NMR (75 MHz, DMSO-d$_6$): δ 17.4 (CH_3), 51.4 (CH_2), 95.1 (C_{quat}), 117.0 (CH), 124.9 (CH), 128.2 (CH), 129.25 (C_{quat}), 129.32 (CH), 129.5 (CH), 132.6 (C_{quat}), 137.8 (CH), 140.9 (C_{quat}), 178.7 (C_{quat}).

EI + MS (70 eV, *m/z* (%)): 366 ($[C_{15}H_{13}INS]^+$, 10), 365 (58), 364 (41), 238 ($[C_{15}H_{12}NS]^+$, 16), 236 (12), 223 ($[C_{14}H_9NS]^+$, 18), 217 ($[C_7H_6I]^+$, 100), 162 ($[C_9H_8NS]^+$, 11), 148 ($[C_8H_6NS]^+$, 36), 118 (11), 108 (11), 104 (13), 90 ($[C_7H_6]^+$, 39), 89 (41), 63 (13).

3.2 Synthesis and analytical data of aroyl-*S,N*-ketene acetals 1

3.2.1 General procedure I (GPI) for the synthesis of aroyl-*S,N*-ketene acetals 1[3],[4]

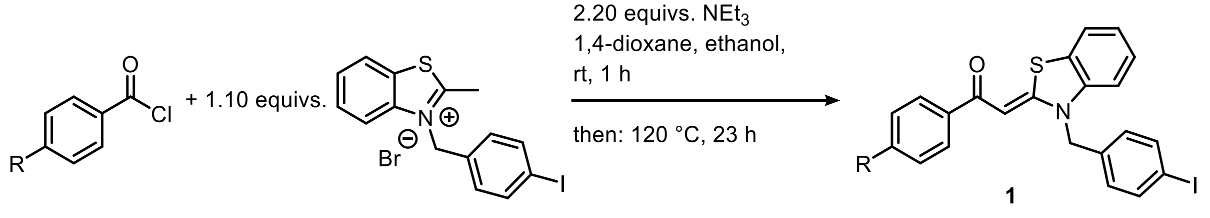

Figure S5. Synthesis of aroyl-S,N-ketene acetals **1**.

Acid chloride (1.00 equiv) and 1.10 equivs of benzothiazolium salt were placed in a sintered, dry screw-cap Schlenk-tube under nitrogen atmosphere and dissolved in 5 mL dry 1,4-dioxane and 2 mL ethanol. 2.20 equivs amine base was added to the reaction mixture and the solution was stirred for 1 h at room temperature. Thereafter, the reaction mixture was stirred at 120 °C (oil bath) for 23 h. The crude product was absorbed onto Celite® and purified by flash chromatography on silica gel (*n*-hexane/acetone 3:1). The product was suspended in *n*-hexane, the sediment was filtrated and dried under vacuo.

Table S6. Experimental details for the synthesis of aroyl-*S,N*-ketene acetals **1**.

entry	acid chloride [g] ([mmol])	benzylbenzo-thiazoliumbromide [g] ([mmol])	yield of product **1** [g] (%)
1	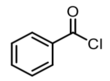 1.40 (10.0)	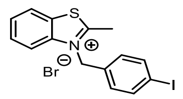 4.89 (11.0)	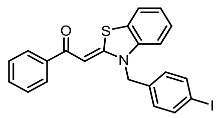 2.86 (61) of **1a**
2	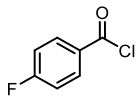 0.632 (4.00)	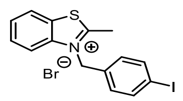 1.96 (4.40)	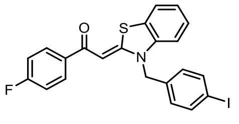 1.28 (66) of **1b**
3	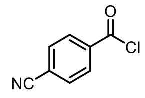 0.825 (5.00)	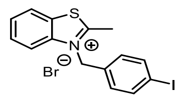 2.45 (5.50)	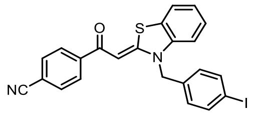 0.794 (32) of **1c**

(*Z*)-2-(3-(4-Iodobenzyl)benzo[*d*]thiazol-2(3*H*)-ylidene)-1-phenylethan-1-one (1a)[4]

The synthesis was performed according to **GPI** to give 2. 87 g (6.14 mmol, 61%) of the desired product **1a** as a yellow solid.

Mp: 172 °C.

R_f (*n*-hexane/acetone 4:1): 0.39.

^{1}H NMR (300 MHz, acetone-d$_6$): δ 5.61 (s, 2 H), 6.92 (s, 1 H), 7.14 (d, ^3J = 8.5 Hz, 2 H), 7.22-7.29 (m, 1 H), 7.35-7.49 (m, 5 H), 7.72-7.81 (m, 3 H), 7.96-8.01 (m, 2 H).

^{13}C NMR (75 MHz, acetone-d$_6$): δ 49.2 (CH_2), 88.2 (CH), 93.4 (CH), 111.5 (CH), 123.3 (CH), 123.9 (CH), 127.6 (CH), 127.8 (C_{quat}), 128.0 (CH), 129.1 (CH), 129.8 (CH), 131.6 (CH), 136.4 (C_{quat}), 138.8 (CH), 140.5 (C_{quat}), 140.8 (C_{quat}), 162.5 (C_{quat}), 184.6 (C_{quat}).

MALDI-TOF (*m/z*): 470.1 ($C_{22}H_{16}INOS+H^+$).

IR $\tilde{\nu}$ [cm^{-1}]: 608 (w), 683 (m), 702 (m), 714 (s), 737 (m), 787 (m), 806 (w), 829 (w), 876 (m), 891 (w), 918 (w), 937 (w), 968 (w), 982 (w), 1004 (m), 1022 (m), 1045 (m), 1057 (m), 1088 (m), 1117 (w), 1134 (w), 1153 (m), 1177 (m), 1196 (m), 1225 (m), 1261 (w), 1292 (w), 1038 (w), 1339 (m), 1395 (m), 1433 (s), 1449 (m), 1481 (s), 1497 (s), 1564 (m), 1601 (w), 2884 (w), 2941 (w), 2972 (w), 2970 (w).

Anal calcd for $C_{22}H_{16}INOS$ [469.0]: C 56.30, H 3.44, N 2.98, S 6.83; Found: C 56.56, H 3.46, N 3.00, S 6.84.

(*Z*)-2-(3-(4-Iodobenzyl)benzo[*d*]thiazol-2(3*H*)-ylidene)-1-(4-fluoro)phenylethan-1-one (1b)[4]

The synthesis was performed according to **GPI** to give 1.28 g (2.63 mmol, 66%) of the desired product **1b** as a yellow solid.

Mp: 181 °C.

R_f (*n*-hexane/acetone 3:1): 0.45.

^{1}H NMR (600 MHz, acetone-d$_6$/CS$_2$ 5:1): δ 5.57 (s, 2 H), 6.84 (s, 1 H), 7.08-7.16 (m, 4 H), 7.21-7.26 (m, 1 H), 7.31-7.39 (m, 2 H), 7.70-7.76 (m, 3 H), 7.97-8.04 (m, 2 H).

^{13}C NMR (150 MHz, acetone-d$_6$/CS$_2$ 5:1): δ 49.2 (CH$_2$), 87.9 (CH), 93.8 (C$_{quat}$), 111.4 (CH), 115.5 (CH), 115.8 (CH), 123.2 (CH), 123.9 (CH), 127.5 (CH), 127.9 (CH), 129.6 (CH), 130.4 (CF), 136.0 (C$_{quat}$), 136.8 (C$_{quat}$), 138.8 (CH), 140.7 (C$_{quat}$), 162.6 (C$_{quat}$), 163.4 (C$_{quat}$), 166.7 (C$_{quat}$), 182.9 (C$_{quat}$).

MALDI-TOF (*m/z*): 488.1 ($C_{22}H_{15}IFNOS+H^+$).

IR $\tilde{\nu}$ [cm^{-1}]: 613 (m), 660 (m), 681 (m), 702 (m); 716 (m), 764 (s), 781 (m), 795 (m), 810 (m), 843 (m), 881 (m), 916 (w), 939 (w), 951 (w), 993 (w), 1007 (m), 1043 (m), 1057 (m), 1067 (m), 1086 (m), 1111 (m), 1134 (w), 1152 (m), 1177 (m), 1188 (m), 1200 (m), 1223 (m), 1265 (w), 1292 (m), 1304 (m), 1339 (m), 1350 (m), 1398 (m), 1450 (m), 1466 (s), 1481 (s), 1578 (m), 1599 (m), 2533 (w), 2883 (w), 2953 (w), 3065 (w).

Anal calcd for $C_{22}H_{15}IFNOS$ [487.0]: C 54.22, H 3.10, N 2.87, S 6.58; Found: C 54.42, H 3.02, N 2.85, S 6.65.

(*Z*)-4-(2-(3-(4-Iodobenzyl)benzo[*d*]thiazol-2(3*H*)-ylidene)acetyl)benzonitrile (1c)[4]

The synthesis was performed according to **GPI** to give 0.794 g (1.61 mmol, 32%) of the desired product **1c** as a yellow solid.

Mp: 235 °C.

R_f (*n*-hexane/acetone 3:1): 0.39.

^{1}H NMR (300 MHz, acetone-d_6/CS_2 5:1): δ 5.63 (s, 2 H), 6.94 (s, 1 H), 7.06-7.15 (m, 2 H), 7.24-7.35 (m, 1 H), 7.38-7.46 (m, 2 H), 7.64-7.82 (m, 5 H), 8.09-8.14 (m, 2 H).

^{13}C NMR (75 MHz, acetone-d_6/CS_2 5:1): δ 49.3 (CH_2), 88.4 (CH), 111.7 (CH), 114.7 (C_{quat}), 118.9 (C_{quat}), 123.4 (CH), 124.3 (CH), 127.7 (CH), 127.9 (CH), 128.6 (CH), 129.6 (CH), 132.3 (C_{quat}), 132.9 (CH), 135.9 (C_{quat}), 138.2 (C_{quat}), 138.8 (CH), 140.6 (C_{quat}), 143.9 (C_{quat}), 163.5 (C_{quat}), 182.1 (C_{quat}).

MALDI-TOF (*m/z*): 495.1 ($C_{23}H_{15}IN_2OS+H^+$).

IR $\tilde{\nu}$ [cm^{-1}]: 660 (m), 681 (w), 698 (w), 716 (m), 750 (s), 766 (s), 785 (m), 799 (m), 820 (m), 841 (m), 856 (m), 881 (s), 932 (w), 978 (w), 1005 (m), 1045 (m), 1057 (m), 1069 (m), 1092 (m), 1111 (m), 1159 (w), 1175 (m), 1198 (m), 1229 (m), 1265 (w), 1292 (m), 1038 (w), 1327 (m), 1393 (m), 1447 (s), 1474 (s), 1501 (m), 1555 (m), 1593 (m), 1711 (w), 1900 (w), 2224 (w), 3042 (w).

Anal calcd for $C_{23}H_{15}IN_2OS$ [494.0]: C 55.88, H 3.06, N 5.67, S 6.49; Found: C 55.83, H 3.29, N 5.26, S 6.79.

4 Synthesis and analytical data of alkynylated aroyl-*S*,*N*-ketene acetals 3

4.1 General procedure II (GPII) for the synthesis of alkynylated aroyl-*S,N*-ketene acetals 3

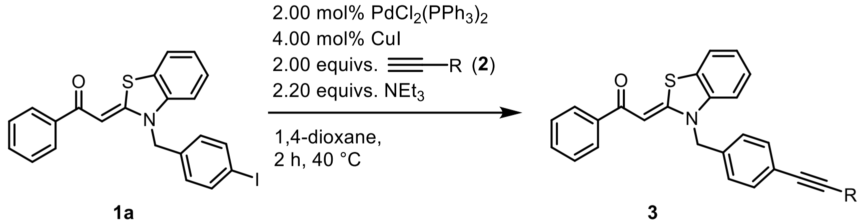

1a **3**

Figure S6. Synthesis of alkynylated aroyl-S,N-ketene acetals **3**.

0.235 g (1.00 equiv, 0.500 mmol) (*Z*)-2-(3-(4-iodobenzyl)benzo[d]thiazol-2(3*H*)-ylidene)-1-phenylethan-1-one (**1a**), 0.007 g (2.00 mol%, 0.010 mmol) bis(triphenylphosphane) palladium(II) dichloride, 0.004 g (4.00 mol%, 0.020 mmol) copper iodide and 2.00 equivs. alkyne **2** were placed in a sintered, dry screw-cap *Schlenk*-tube and dissolved in 3 mL dry 1,4-dioxane. Subsequently, 0.111 g (2.20 equivs., 0.150 mL, 1.10 mmol) of triethylamine was added, the reaction mixture was degassed with nitrogen and then stirred at 40 °C for 2 h. The product was then adsorbed on Celite® and the solvent was removed under reduced pressure. The crude product was purified by column chromatography on silica gel (*n*-hexane/acetone) and then suspended several times with *n*-hexane.

Table S7. Experimental details for the synthesis of alkynylated aroyl-*S*,*N*-ketene acetals **3**.

entry	alkyne **2**<br>[g] ([mmol])	yield of product **3**<br>[g] (%)
1	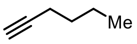<br>0.082 (1.00) of **2a**	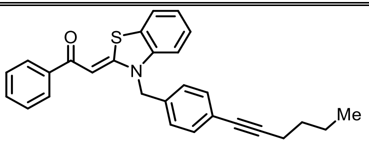<br>0.190 (90) of **3a**
2	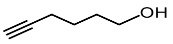<br>0.098 (1.00) of **2b**	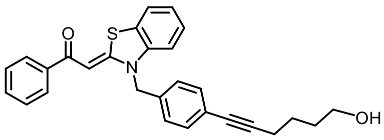<br>0.157 (71) of **3b**
3	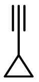<br>0.066 (1.00) of **2c**	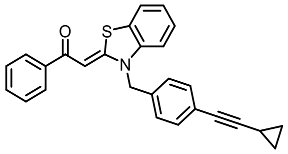<br>0.203 (100) of **3c**
4	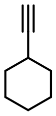<br>0.108 (1.00) of **2d**	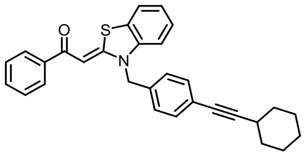<br>0.218 (97) of **3d**
5	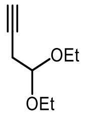<br>0.142 (1.00) of **2e**	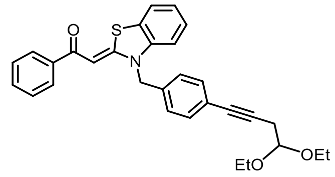<br>0.232 (96) of **3e**
6	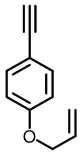<br>0.235 (1.00) of **2f**	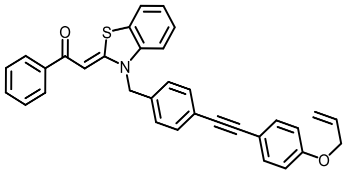<br>0.142 (57) of **3f**
7	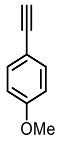<br>0.158 (1.00) of **2g**	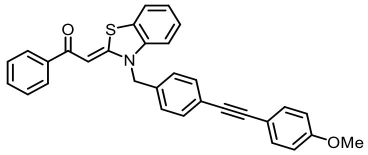<br>0.129 (91) of **3g**

Table S7 continued.

entry	alkyne **2**<br>[g] ([mmol])	yield of product **3**<br>[g] (%)
8	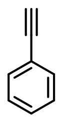<br>0.102 (1.00) of **2h**	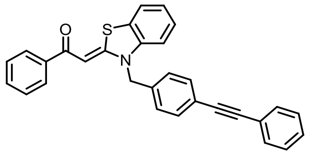<br>0.459 (92) of **3h**
9	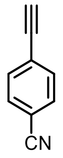<br>0.127 (1.00) of **2i**	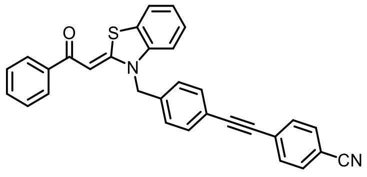<br>0.223 (95) of **3i**
10[(a)]	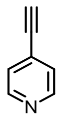<br>0.063 (0.614) of **2j**	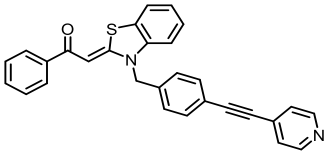<br>0.121 (89) of **3j**
11	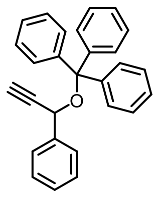<br>0.375 (1.00) of **2k**	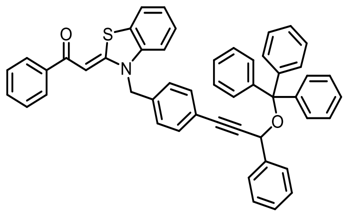<br>0.357 (100) of **3k**
12	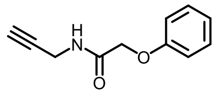<br>0.189 (1.00) of **2l**	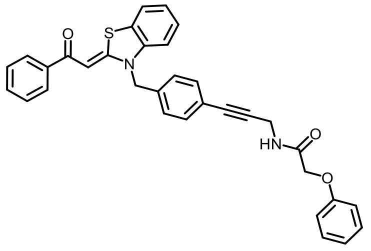<br>0.203 (76) of **3l**
13	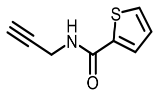<br>0.165 (1.00) of **2m**	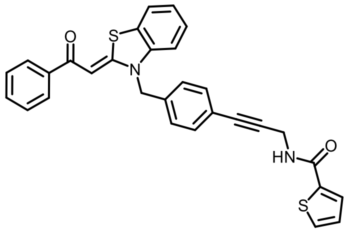<br>0.132 (52) of **3m**

Table S7 continued.

entry	alkyne **2** [g] ([mmol])	yield of product **3** [g] (%)
14	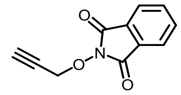 0.201 (1.00) of **2n**	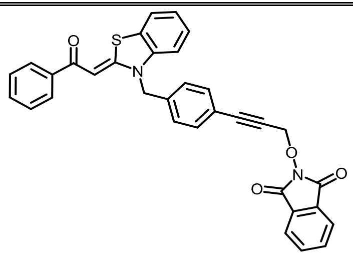 0.140 (52) of **3n**
15[b]	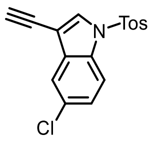 0.252 (0.760) of **2o**	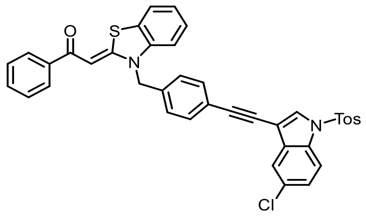 0.247 (96) of **3o**
16[b]	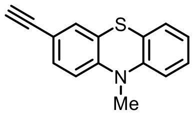 0.181 (0.760) of **2p**	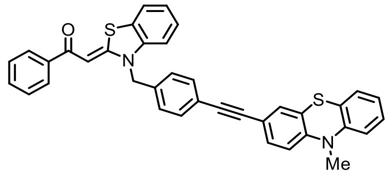 0.190 (86) of **3p**
17[c]	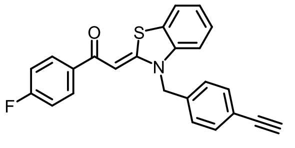 0.231 (0.600) of **4b**	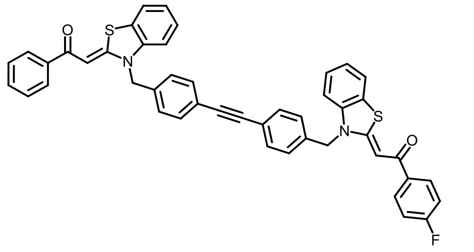 0.091 (42) of **3q**
18[d]	0.590 (6.00) of **2q**	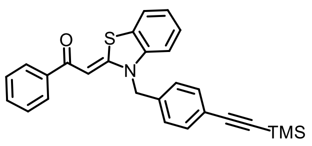 1.30 (99) of **3r**
19[e]	0.098 (1.00) of **2q**	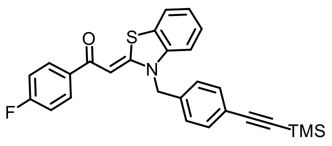 0.230 (99) of **3s**

Table S7 continued.

Entry	Alkyne **2** [g] ([mmol])	Yield of product **3** [g] (%)
20[(f)]	0.246 (2.50) of **2q**	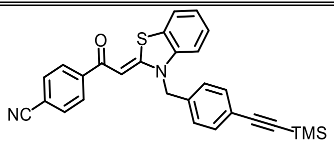 0.378 (65) of **3t**

(a): Aroyl-*S,N*-ketene acetal **1a**: 0.144 g (0.307 mmol), $Pd(PPh_3)_2Cl_2$: 0.004 g (0.00614 mmol), CuI: 0.002 g (0.0123 mmol), NEt_3: 0.068 g (0.092 mL, 0.675 mmol), 2 mL 1,4-dioxane.

(b): Aroyl-*S,N*-ketene acetal **1a**: 0.180 g (0.382 mmol), $Pd(PPh_3)_2Cl_2$: 0.004 g (0.00614 mmol), CuI: 0.002 g (0.0123 mmol), NEt_3: 0.084 g (0.115 mL, 0.840 mmol), 2 mL 1,4-dioxane.

(c): Aroyl-*S,N*-ketene acetal **1a**: 0.141 g (0.300 mmol), $Pd(PPh_3)_2Cl_2$: 0.004 g (0.00614 mmol), CuI: 0.002 g (0.0123 mmol), NEt_3: 0.067 g (0.090 mL, 0.660 mmol), 2 mL 1,4-dioxane.

(d): Aroyl-*S,N*-ketene acetal **1a**: 1.41 g (3.00 mmol), $Pd(PPh_3)_2Cl_2$: 0.022 g (0.030 mmol), CuI: 0.012 g (0.060 mmol), NEt_3: 0.607 g (0.830 mL, 6.00 mmol), 10 mL 1,4-dioxane.

(e): Instead of aroyl-*S,N*-ketene acetal **1a**, 4-fluoro-aroyl-*S,N*-ketene acetal **1b** (0.244 g, 0.500 mmol), was used.

(f): Instead of aroyl-*S,N*-ketene acetal **1a,** 4-cyano-aroyl-*S,N*-ketene acetal **1c** was used. Aroyl-*S,N*-ketene acetal **1c**: 0.617 g (1.25 mmol), $Pd(PPh_3)_2Cl_2$: 0.009 g (0.0125 mmol), CuI: 0.005 g (0.025 mmol), NEt_3: 0.253 g (0.345 mL, 2.50 mmol), 4 mL 1,4-Dioxan.

4.1.1 Spectroscopic data

(Z)-2-(3-(4-(Hex-1-yn-1-yl)benzyl)benzo[*d*]thiazol-2(3*H*)-ylidene)-1-phenylethan-1-one (3a)

The synthesis was performed according to **GPII** using *n*-hexane/acetone 7:1 as an eluent to give 0.190 g (0.449 mmol, 90%) of the desired product **3a** as a yellow solid.

Mp: 138 °C.

R_f (*n*-hexane/acetone 7:1): 0.38.

^{1}H NMR (300 MHz, acetone-d$_6$): δ 0.90 (t, ^3J = 6.9 Hz, 3 H), 1.37 – 1.59 (m, 4 H), 2.37 (t, ^3J = 6.9 Hz, 2 H), 5.63 (s, 2 H), 6.91 (s, 1 H), 7.20 – 7.33 (m, 3 H), 7.33 – 7.51 (m, 7 H), 7.79 (dt, ^3J = 7.7 Hz, ^4J = 1.0 Hz, 1 H), 7.95 – 8.01 (m, 2 H).

^{13}C NMR (75 MHz, acetone-d$_6$): δ 13.8 (CH_3), 19.4 (CH_2), 22.6 (CH_2), 31.5 (CH_2), 49.4 (CH_2), 80.8 (C_{quat}), 88.2 (CH), 91.4 (C_{quat}), 111.5 (CH), 123.2 (CH), 123.9 (CH), 124.5 (C_{quat}), 127.6 (CH), 127.6 (CH), 127.8 (C_{quat}), 127.9 (CH), 129.1 (CH), 131.6 (CH), 132.7 (CH), 135.9 (CH), 140.6 (C_{quat}), 140.9 (C_{quat}), 162.6 (C_{quat}), 184.6 (C_{quat}).

MALDI-TOF (*m/z*): 424.3 ($C_{28}H_{25}NOS+H^+$).

IR $\tilde{\nu}$ [cm^{-1}]: 619 (w), 646 (w), 667 (w), 691 (w), 702 (m), 719 (s), 743 (m), 797 (w), 820 (w), 878 (s), 926 (w), 945 (w), 972 (w), 972 (w), 991 (w), 1001 (w), 1024 (w), 1043 (w), 1061 (m), 1092 (w), 1111 (w), 1138 (w), 1157 (w), 1179 (w), 1192 (m), 1227 (m), 1265 (w), 1294 (w), 1300 (w), 1331 (w), 1377 (w), 1412 (w), 1435 (s), 1464 (s), 1495 (m), 1558 (w), 1568 (w), 1595 (w), 1711 (w), 2559 (w), 2803 (w), 2860 (w), 2870 (w), 2930 (w), 2957 (w), 3057 (w).

UV/Vis (C_3H_6O): $\lambda_{max}(\varepsilon)$ = 256 (50700), 382 (37000).

Anal calcd for $C_{28}H_{25}NOS$ [423.6]: C 79.40, H 9.95, N 3.31, S 7.57; Found: C 79.18, H 9.89, N 3.22, S 7.47.

(*Z*)-2-(3-(4-(6-Hydroxyhex-1-yn-1-yl)benzyl)benzo[*d*]thiazol-2(3*H*)-ylidene)-1-phenylethan-1-one (3b)

The synthesis was performed according to **GPII** using *n*-hexane/acetone 3:1 to 1:1 as an eluent to give 0.157 g (0.358 mmol, 71%) of the desired product **3b** as a yellow solid.

Mp: 92 °C.

R_f (*n*-hexane/acetone 3:1): 0.13.

^{1}H NMR (300 MHz, acetone-d$_6$): δ 1.59 – 1.66 (m, 3 H), 2.38 – 2.43 (m, 2 H), 2.85 (s, 2 H), 3.48 – 3.58 (m, 2 H), 5.63 (s, 2 H), 6.91 (s, 1 H), 7.23 – 7.31 (m, 3 H), 7.35 – 7.49 (m, 7 H), 7.79 (dt, ^3J = 7.7 Hz, ^4J = 0.9 Hz, 1 H), 7.95 – 8.00 (m, 2 H).

^{13}C NMR (75 MHz, acetone-d$_6$): δ 19.5 (CH_2OH), 26.1 (CH_2), 32.9 (CH_2), 49.4 (CH_2), 61.9 (CH_2), 80.9 (C_{quat}), 88.2 (CH), 91.5 (C_{quat}), 111.5 (CH), 123.3 (CH), 123.9 (CH), 124.5 (C_{quat}), 127.6 (CH), 127.6 (CH), 127.8 (C_{quat}), 127.9 (CH), 129.1 (CH), 131.6 (CH), 132.7 (CH), 135.9 (CH), 140.6 (C_{quat}), 140.9 (C_{quat}), 162.6 (C_{quat}), 184.6 (C_{quat}).

MALDI-TOF (*m/z*): 440.3 ($C_{28}H_{25}NO_2S$+H^+).

IR $\tilde{\nu}$ [cm^{-1}]: 608 (m), 669 (m), 691 (m), 702 (m), 721 (s), 741 (s), 797 (m), 820 (m), 837 (w), 878 (s), 928 (w), 970 (w), 988 (w), 1022 (m), 1061 (m), 1138 (w), 1157 (m), 1179 (m), 1192 (m), 1227 (m), 1265 (w), 1294 (m), 1302 (m), 1329 (m), 1389 (w), 1412 (m), 1437 (s), 1450 (s), 1497 (m), 1560 (m), 1593 (w), 1973 (w), 2154 (w), 2189 (w), 2224 (w), 2829 (w).

UV/Vis (C_3H_6O): $\lambda_{max}(\varepsilon)$ = 256 (37500), 382 (35300).

Anal calcd for $C_{28}H_{25}NO_2S$ [439.2]: C 76.51, H 5.73, N 3.19, S 7.29; Found: C 76.60, H 5.73, N 3.21, S 7.64.

(*Z*)-2-(3-(4-(Cyclopropylethynyl)benzyl)benzo[*d*]thiazol-2(3*H*)-ylidene)-1-phenylethan-1-one (3c)

The synthesis was performed according to **GPII** using *n*-hexane/acetone 4:1 as an eluent to give 0.203 g (0.499 mmol, 100%) of the desired product **3c** as a yellow solid.

Mp: 175 °C.

R_f (*n*-hexane/acetone 4:1): 0.39.

^{1}H NMR (300 MHz, acetone-d_6/CS_2 5:1): δ 0.66 – 0.78 (m, 2 H), 0.82 – 0.95 (m, 2 H), 1.44 (tt, ^3J = 8.2 Hz, ^3J = 5.0 Hz, 1 H), 5.56 (s, 2 H), 6.81 (s, 1 H), 7.19 – 7.28 (m, 3 H), 7.29 – 7.48 (m, 7 H), 7.74 (d, ^3J = 7.6 Hz, 1 H), 7.90 – 7.96 (m, 2 H).

^{13}C NMR (75 MHz, acetone-d_6/CS_2 5:1): δ 0.8 (CH), 9.1 (CH_2), 49.5 (CH_2), 76.1 (C_{quat}), 88.2 (CH), 94.8 (C_{quat}), 111.3 (CH), 123.2 (CH), 123.8 (CH), 124.4 (C_{quat}), 127.43 (CH), 127.45 (CH), 127.9 (CH), 128.0 (C_{quat}), 128.9 (CH), 131.4 (CH), 132.7 (CH), 135.4 (C_{quat}), 140.4 (C_{quat}), 140.8 (C_{quat}), 162.3 (C_{quat}), 184.3 (C_{quat}).

MALDI-TOF (*m/z*): 408.3 ($C_{27}H_{21}NOS+H^+$).

IR $\tilde{\nu}$ [cm^{-1}]: 689 (m), 704 (w), 718 (s), 741 (m), 758 (m), 779 (w), 816 (w), 878 (m), 924 (w), 955 (w), 1061 (w), 1092 (w), 1153 (w), 1177 (w), 1190 (m), 1225 (w), 1254 (w), 1300 (w), 1331 (w), 1410 (w), 1437 (m), 1468 (s), 1566 (m), 1595 (w), 2170 (w), 2247 (w), 2342 (w), 2401 (w), 2413 (w), 2448 (w), 2837 (w).

UV/Vis (C_3H_6O): $\lambda_{max}(\varepsilon)$ = 257, 382.2 (37400).

Anal calcd for $C_{27}H_{21}NOS$ [407.5]: C 79.58, H 5.19, N 3.44, S 7.87; Found: C 79.38, H 5.25, N 3.39, S 7.70.

(Z)-2-(3-(4-(Cyclohexylethynyl)benzyl)benzo[*d*]thiazol-2(3*H*)-ylidene)-1-phenylethan-1-one (3d)

The synthesis was performed according to **GPII** using *n*-hexane/acetone 4:1 as an eluent to give 0.218 g (0.485 mmol, 97%) of the desired product **3d** as a yellow solid.

Mp: 192 °C.

R_f (*n*-hexane/acetone 4:1): 0.42.

^{1}H NMR (300 MHz, acetone-d$_6$): δ 1.22 – 1.94 (m, 10 H), 2.57 (tt, ^3J = 8.9 Hz, ^3J = 3.7 Hz, 1 H), 5.63 (s, 2 H), 6.91 (s, 1 H), 7.20 – 7.32 (m, 3 H), 7.34 – 7.50 (m, 7 H), 7.80 (d, ^3J = 7.7 Hz, 1 H), 7.98 – 8.02 (m, 2 H).

^{13}C NMR (75 MHz, acetone-d$_6$): 25.4 (CH_2), 26.5 (CH_2), 30.2 (CH), 33.4 (CH_2), 49.4 (CH_2), 80.9 (C_{quat}), 88.2 (CH), 95.3 (C_{quat}), 111.5 (CH), 123.3 (CH), 123.9 (CH), 124.5 (C_{quat}), 127.6 (CH), 127.6 (CH), 127.8 (C_{quat}), 127.9 (CH), 129.1 (CH), 131.6 (CH), 132.7 (CH), 135.8 (C_{quat}), 140.6 (C_{quat}), 140.9 (C_{quat}), 162.6 (C_{quat}), 184.6 (C_{quat}).

MALDI-TOF (*m/z*): 450.3 ($C_{30}H_{27}NOS+H^+$).

IR $\tilde{\nu}$ [cm^{-1}]: 629 (w), 673 (w),691 (w), 719 (m), 733 (s), 746 (m), 818 (w), 843 (w), 881 (m), 922 (w), 1022 (w), 1047 (w), 1061 (w), 1096 (w), 1155 (w), 1180 (w), 1192 (w), 1204 (w), 1233 (w), 1269 (w), 1298 (w), 1325 (w), 1346 (w), 1393 (w), 1412 (w), 1437 (m), 1458 (m), 1477 (s), 1568 (w), 1595 (w), 2853 (w), 2928 (w), 3024 (w), 3046 (w).

UV/Vis (C_3H_6O): $\lambda_{max}(\varepsilon)$ = 258, 382 (35500).

Anal calcd for $C_{30}H_{27}NOS$ [423.6]: C 80.14, H 6.05, N 3.12, S 7.13; Found: C 79.92, H 6.03, N 2.99, S 6.86.

(*Z*)-2-(3-(4-(4,4-Diethoxybut-1-yn-1-yl)benzyl)benzo[*d*]thiazol-2(3*H*)-ylidene)-1-phenylethan-1-one (3e)

The synthesis was performed according to **GPII** using *n*-hexane/acetone 3:1 as an eluent to give 0.232 g (0.480 mmol, 96%) of the desired product **3e** as a yellow solid.

R_f (*n*-hexane/acetone 3:1): 0.40.

^{1}H NMR (300 MHz, acetone-d$_6$): δ 1.14 (t, ^3J = 7.8 Hz, 6 H), 2.65 (d, ^3J = 5.8 Hz, 2 H), 3.52 – 3.70 (m, 4 H), 4.17 (t, ^3J = 5.8 Hz, 1 H), 5.61 (s, 2 H), 6.92 (s, 1 H), 7.12 – 7.16 (m, 1 H), 7.22 – 7.31 (m, 2 H), 7.33 – 7.49 (m, 6 H), 7.72 – 7.81 (m, 2 H), 7.96 – 8.01 (m, 2 H).

^{13}C NMR (75 MHz, acetone-d$_6$): 15.6 (CH_3), 26.3 (CH_2), 49.2 (CH_2), 62.4 (CH_2), 64.6 (CH), 81.8 (C_{quat}), 88.2 (CH), 93.4 (C_{quat}), 111.5 (CH), 123.3 (CH), 123.9 (CH), 127.3 (C_{quat}), 127.6 (CH), 127.8 (C_{quat}), 128.0 (CH), 128.9 (C_{quat}), 129.1 (CH), 129.7 (CH), 131.6 (CH), 132.8 (CH), 136.3 (CH), 138.8 (CH), 140.5 (C_{quat}), 140.8 (C_{quat}), 162.6 (C_{quat}), 184.6 (C_{quat}).

MALDI-TOF (*m/z*): 483.3 ($C_{30}H_{29}NO_3S+H^+$).

IR $\tilde{\nu}$ [cm^{-1}]: 625 (w), 648 (w), 667 (m), 687 (m), 696 (m), 719 (s), 791 (m), 814 (m), 841 (w), 878 (s), 926 (w), 1005 (m), 1022 (m), 1045 (m), 1059 (m), 1090 (m), 1115 (m), 1155 (w), 1177 (m), 1196 (m), 1227 (m), 1261 (w), 1292 (m), 1300 (m), 1331 (m), 1373 (w), 1402 (m), 1435 (s), 1464 (s), 1495 (m), 1566 (m), 1595 (m), 1732 (w), 1884 (w), 2174 (w), 2536 (w), 2594 (w), 2733 (w), 2799 (w), 2870 (w), 2884 (w), 2901 (w), 2972 (w), 3019 (w), 3057 (w), 3296 (w), 3522 (w), 3649 (w).

UV/Vis (C_3H_6O): $\lambda_{max}(\varepsilon)$ = 382 (38000).

Anal calcd for $C_{30}H_{29}NO_3S$ [483.2]: C 74.51, H 6.04, N 2.90, S 6.63; Found: C 74.82, H 6.23, N 2.97, S 6.96.

(*Z*)-2-(3-(4-((4-(Allyloxy)phenyl)ethynyl)benzyl)benzo[*d*]thiazol-2(3*H*)-ylidene)-1-phenylethan-1-one (3f)

The synthesis was performed according to **GPII** using *n*-hexane/acetone 3:1 as an eluent to give 0.142 g (0.285 mmol, 57%) of the desired product **3f** as a yellow solid.

Mp: 120 °C.

R_f (*n*-hexane/acetone 3:1): 0.29.

^1H NMR (300 MHz, acetone-d$_6$): δ 4.61 (dt, ^3J = 5.1 Hz, ^4J = 1.6 Hz, 2 H), 5.25 (dq, ^3J = 10.7 Hz, ^4J = 1.6 Hz, 1 H), 5.41 (dq, ^3J = 15.6 Hz, ^4J = 1.8 Hz, 1 H), 5.67 (s, 2 H), 6.00 – 6.22 (m, 1 H), 6.92 – 7.01 (m, 3 H), 7.13 – 7.16 (m, 1 H), 7.24 – 7.32 (m, 1 H), 7.36 – 7.53 (m, 9 H), 7.73 – 7.83 (m, 2 H), 7.97 – 8.00 (m, 2 H).

^{13}C NMR (75 MHz, acetone-d$_6$): δ 49.2 (CH_2), 49.5 (CH_2), 69.4 (CH_2), 88.17 (C_{quat}), 88.23 (CH), 90.5 (C_{quat}), 111.5 (CH),1 115.8 (CH), 117.7 (CH), 123.3 (CH), 123.8 (C_{quat}), 123.9 (CH), 127.6 (CH), 127.8 (CH), 128.0 (CH), 129.1 (CH), 129.8 (C_{quat}), 130.4 (C_{quat}), 131.6 (CH), 132.6 (CH), 133.8 (CH), 134.3 (C_{quat}), 136.5 (C_{quat}), 138.9 (C_{quat}), 140.6 (C_{quat}), 140.9 (C_{quat}), 159.9 (C_{quat}), 162.6 (C_{quat}), 184.6 (C_{quat}).

MALDI-TOF (*m/z*): 500.3 ($C_{33}H_{25}NO_2S+H^+$).

IR $\tilde{\nu}$ [cm^{-1}]: 642 (w), 667 (m), 692 (m), 719 (s), 797 (w), 829 (m), 878 (s), 926 (w), 999 (m), 1022 (m), 1043 (m), 1061 (m), 1092 (m), 1109 (w), 1136 (m), 1157 (m), 1175 (m), 1192 (m), 1227 (s), 1285 (m), 1302 (m), 1331 (m), 1412 (m), 1433 (s), 1452 (s), 1495 (m), 1566 (m), 1599 (m), 1672 (w), 1730 (w), 2122 (w), 2868 (w), 2901 (w), 2911 (w), 2972 (w), 3551 (w).

UV/Vis (C_3H_6O): $\lambda_{max}(\varepsilon)$ = 293 (31000), 312 (25300), 382 (38900).

Anal calcd for $C_{33}H_{25}NO_2S$ [499.2]: C 79.33, H 5.04, N 2.80, S 6.42; Found: C 79.43, H 4.87, N 3.05, S 6.48.

(Z)-2-(3-(4-((4-Methoxyphenyl)ethynyl)benzyl)benzo[*d*]thiazol-2(3*H*)-ylidene)-1-phenylethan-1-one (3g)

The synthesis was performed according to **GPII** using *n*-hexane/acetone 4:1 as an eluent to give 0.129 g (0.273 mmol, 91%) of the desired product **3g** as a yellow solid.

Mp: 188 °C.

R_f (*n*-hexane/acetone 4:1): 0.21.

^{1}H NMR (300 MHz, acetone-d$_6$/CS$_2$ 5:1): δ 3.82 (s, 3 H), 5.62 (s, 2 H), 6.85 – 6.97 (m, 3 H), 7.21 – 7.29 (m, 1 H), 7.30 – 7.54 (m, 11 H), 7.76 (dt, ^3J = 7.7 Hz, ^4J = 0.9 Hz, 1 H), 7.91 – 7.99 (m, 2 H).

^{13}C NMR (75 MHz, acetone-d$_6$/CS$_2$ 5:1): δ 49.5 (CH_2), 55.7 (CH_3), 88.3 (CH), 88.4 (C_{quat}), 90.8 (C_{quat}), 111.4 (CH), 115.0 (CH), 115.8 (C_{quat}), 123.2 (CH), 123.8 (CH), 123.9 (C_{quat}), 127.5 (CH), 127.7 (CH), 128.0 (CH), 129.0 (CH), 131.5 (CH), 132.6 (CH), 133.8 (CH), 136.2 (C_{quat}), 140.5 (C_{quat}), 140.8 (C_{quat}), 160.8 (C_{quat}), 162.4 (C_{quat}), 184.4 (C_{quat}).

MALDI-TOF (*m/z*): 474.3 ($C_{31}H_{23}NO_2S+H^+$).

IR $\tilde{\nu}$ [cm^{-1}]: 613 (w), 669 (w), 706 (w), 719 (s), 743 (s), 818 (m), 831 (m), 883 (m), 1001 (w), 1024 (w), 1061 (w), 1092 (w), 1105 (w), 1138 (w), 1173 (w), 1200 (w), 1230 (m), 1248 (m), 1269 (w), 1287 (w), 1339 (w), 1350 (w), 1385 (w), 1410 (w), 1435 (m), 1477 (s), 1495 (m), 1568 (m), 1601 (w), 2220 (w), 2263 (w), 2511 (w), 2833 (w), 2932 (w).

UV/Vis (C_3H_6O): $\lambda_{max}(\varepsilon)$ = 382 (49800).

Anal calcd for $C_{31}H_{23}NO_2S$ [423.6]: C 78.62, H 4.90, N 2.96, S 6.77; Found: C 78.42, H 4.95, N 2.87, S 6.89.

(Z)-1-Phenyl-2-(3-(4-(phenylethynyl)benzyl)benzo[*d*]thiazol-2(3*H*)-ylidene)ethan-1-one (3h)

The synthesis was performed according to **GPII** using *n*-hexane/acetone 4:1 to 2:1 as an eluent to give 0.203 g (0.459 mmol, 92%) of the desired product **3h** as a yellow solid.

Mp: 230 °C.

R_f (*n*-hexane/acetone 4:1): 0.35.

^{1}H NMR (300 MHz, acetone-d$_6$/CS$_2$ 5:1): δ 5.60 (s, 2 H), 6.82 (s, 1 H), 7.23 (ddd, ^3J = 8.0 Hz, ^3J = 6.2 Hz, ^4J = 1.9 Hz, 1 H), 7.30 – 7.57 (m, 14 H), 7.74 (d, ^3J = 7.8 Hz, 1 H), 7.89 – 7.98 (m, 2 H).

^{13}C NMR (75 MHz, acetone-d$_6$/CS$_2$ 5:1): δ 49.5 (CH$_2$), 88.2 (CH), 89.9 (C$_{quat}$), 90.7 (C$_{quat}$), 111.2 (CH), 123.2 (CH), 123.5 (C$_{quat}$), 123.8 (CH), 123.9 (C$_{quat}$), 127.4 (CH), 127.6 (CH), 127.9 (CH), 128.0 (C$_{quat}$), 128.9 (CH), 129.2 (CH), 129.3 (CH), 131.4 (CH), 132.2 (CH), 132.8 (CH), 136.4 (C$_{quat}$), 140.4 (C$_{quat}$), 140.7 (C$_{quat}$), 162.3 (C$_{quat}$), 184.2 (C$_{quat}$).

MALDI-TOF (*m/z*): 444.3 ($C_{30}H_{21}NOS+H^+$).

IR $\tilde{\nu}$ [cm^{-1}]: 669 (w), 689 (m), 706 (m), 733 (s), 748 (m), 816 (w), 851 (w), 883 (m), 1001 (w), 1042 (w), 1063 (w), 1098 (w), 1138 (w), 1157 (w), 1177 (w), 1202 (m), 1234 (w), 1275 (w), 1302 (w), 1335 (w), 1362 (w), 1387 (w), 1412 (w), 1441 (m), 1458 (m), 1474 (s), 1495 (m), 1570 (w), 1593 (w), 2592 (w), 2918 (w), 3061 (w), 3092 (w), 3343 (w), 3528 (w), 3657 (w), 3771 (w), 3906 (w).

UV/Vis (C_3H_6O): $\lambda_{max}(\varepsilon)$ = 381.9 (45700).

Anal calcd for $C_{30}H_{21}NOS$ [443.6]: C 81.24, H 4.77, N 3.16, S 7.23; Found: C 80.99, H 4.80, N 3.08, S 7.12.

(*Z*)-4-((4-((2-(2-Oxo-2-phenylethylidene)benzo[*d*]thiazol-3(2*H*)-yl)methyl)phenyl)ethynyl)benzonitrile (3i)

The synthesis was performed according to **GPII** using *n*-hexane/acetone 4:1 to 3:1 to 2:1 as an eluent to give 0.223 g (0.476 mmol, 95%) of the desired product **3i** as a yellow solid.

Mp: 194 °C.

R_f (*n*-hexane/acetone 4:1): 0.20.

^{1}H NMR (300 MHz, acetone-d_6/CS_2 5:1): δ 5.65 (s, 2 H), 6.85 (s, 1 H), 7.25 (ddd, ^3J = 8.1 Hz, ^3J = 5.3 Hz, ^4J = 3.1 Hz, 1 H), 7.33 – 7.49 (m, 7 H), 7.53 – 7.62 (m, 2 H), 7.64 – 7.72 (m, 2 H), 7.73 – 7.82 (m, 3 H), 7.91 – 7.99 (m, 2 H).

^{13}C NMR (75 MHz, acetone-d_6/CS_2 5:1): δ 49.5 (CH_2), 88.3 (CH), 89.0 (C_{quat}), 93.8 (C_{quat}), 111.3 (CH), 112.6 (C_{quat}), 118.8 (C_{quat}), 122.5 (C_{quat}), 123.3 (CH), 123.9 (CH), 127.5 (CH), 127.8 (CH), 127.9 (CH), 128.6 (C_{quat}), 129.0 (CH), 131.5 (CH), 133.0 (CH), 133.1 (CH), 133.5 (C_{quat}), 137.5 (C_{quat}), 140.4 (C_{quat}), 140.8 (C_{quat}), 162.4 (C_{quat}), 184.4 (C_{quat}).

MALDI-TOF (*m/z*): 469.2 ($C_{31}H_{20}N_2OS+H^+$).

IR $\tilde{\nu}$ [cm^{-1}]: 631 (w), 667 (w), 689 (w), 706 (w), 719 (s), 741 (m), 799 (w), 822 (w), 841 (m), 878 (m), 928 (w), 972 (w), 1016 (w), 1038 (w), 1061 (w), 1092 (w), 1107 (w), 1134 (w), 1155 (w), 1177 (w), 1190 (w), 1227 (w), 1263 (w), 1292 (w), 1304 (w), 1327 (w), 1354 (w), 1408 (w), 1437 (m), 1450 (w), 1470 (s), 1497 (m), 1557 (w), 1568 (w), 1603 (w), 2220 (w), 3019 (w), 3057 (w), 3088 (w), 3252 (w).

UV/Vis (C_3H_6O): $\lambda_{max}(\varepsilon)$ = 316, 382 (34100).

Anal calcd for $C_{31}H_{20}N_2OS$ [423.6]: C 79.46, H 4.30, N 5.98, S 6.84; Found: C 79.59, H 4.19, N 6.26, S 6.60.

(*Z*)-1-Phenyl-2-(3-(4-(pyridine-4-ylethynyl)benzyl)benzo[*d*]thiazol-2(3*H*)-ylidene)ethan-1-one (3j)

The synthesis was performed according to **GPII** using *n*-hexane/acetone 4:1 to 3:1 to 2:1 as an eluent to give 0.121 g (0.273 mmol, 89%) of the desired product **3j** as a yellow solid.

Mp: 237 °C.

R_f (*n*-hexane/acetone 4:1): 0.10.

^{1}H NMR (300 MHz, acetone-d$_6$/CS$_2$ 5:1): δ 5.63 (s, 2 H), 6.81 (s, 1 H), 7.24 (ddd, ^3J = 7.9 Hz, ^3J = 6.2 Hz, ^4J = 1.7 Hz, 1 H), 7.29 – 7.51 (m, 9 H), 7.53 – 7.62 (m, 2 H), 7.74 (d, ^3J = 7.5 Hz, 1 H), 7.88 – 7.96 (m, 2 H), 8.53 – 8.66 (m, 2 H).

^{13}C NMR (75 MHz, acetone-d$_6$/CS$_2$ 5:1): δ 49.5 (CH_2), 88.0 (C_{quat}), 88.3 (CH), 94.0 (C_{quat}), 111.2 (CH), 122.4 (C_{quat}), 123.2 (CH), 123.8 (CH), 126.0 (CH), 126.6 (CH), 127.4 (CH), 127.8 (CH), 127.9 (CH), 128.0 (CH), 128.9 (CH), 131.4 (CH), 133.1 (CH), 137.5 (C_{quat}), 140.3 (C_{quat}), 140.7 (C_{quat}), 150.6 (CH), 162.3 (C_{quat}), 184.2 (C_{quat}).

MALDI-TOF (*m/z*): 445.3 ($C_{29}H_{20}N_2OS+H^+$).

IR $\tilde{\nu}$ [cm^{-1}]: 691 (w), 702 (m), 719 (m), 731 (s), 789 (w), 814 (w), 831 (m), 878 (m), 1022 (w), 1065 (w), 1098 (w), 1175 (w), 1194 (w), 1204 (w), 1229 (w), 1273 (w), 1292 (w), 1308 (w), 1327 (w), 1362 (w), 1396 (w), 1414 (w), 1441 (m), 1458 (m), 1477 (s), 1570 (w), 1587 (m), 1605 (w), 2216 (w), 3026 (w), 3246 (w), 3507 (w), 3566 (w), 3904 (w).

UV/Vis (C_3H_6O): $\lambda_{max}(\varepsilon)$ = 285.9 (41900), 382.5 (32800).

HPLC-HRMS (*m/z*): HRMS (ESI) ber. für $C_{29}H_{20}N_2OS+H^+$: 445.1369; Found: 445.1370. HPLC-rein.

(*Z*)-1-Phenyl-2-(3-(4-(3-phenyl-3-(trityloxy)prop-1-yn-1-yl)benzyl)benzo[*d*]thiazol-2(3*H*)-ylidene)ethan-1-one (3k)

The synthesis was performed according to **GPII** using *n*-hexane/acetone 4:1 as an eluent to give 0.357 g (0.499 mmol, 99%) of the desired product **3k** as a yellow solid.

Mp: 128 °C.

R_f (*n*-hexane/acetone 4:1): 0.28.

^{1}H NMR (300 MHz, acetone-d$_6$): δ 5.24 (s, 1 H), 5.59 (s, 2 H), 6.87 (s, 1 H), 7.08 – 7.50 (m, 24 H), 7.51 – 7.62 (m, 6 H), 7.78 (dt, ^3J = 7.8 Hz, ^4J = 1.0 Hz, 1 H), 7.93 – 8.02 (m, 2 H).

^{13}C NMR (75 MHz, acetone-d$_6$): δ 49.4 (CH_2), 67.9 (CH), 86.9 (C_{quat}), 88.2 (CH), 89.4 (C_{quat}), 90.7 (C_{quat}), 111.4 (CH), 123.1 (C_{quat}), 123.3 (CH), 123.9 (CH), 127.4 (CH), 127.6 (CH), 127.7 (CH), 127.8 (C_{quat}), 127.9 (CH), 128.1 (CH), 128.5 (CH), 129.1 (CH), 129.2 (CH), 129.9 (CH), 131.6 (CH), 132.7 (CH), 136.6 (C_{quat}), 140.5 (C_{quat}), 140.9 (C_{quat}), 141.7 (C_{quat}), 145.2 (C_{quat}), 162.5 (C_{quat}), 184.6 (C_{quat}).

MALDI-TOF (*m/z*): 716.3 ($C_{50}H_{37}NO_2S+H^+$).

IR $\tilde{\nu}$ [cm^{-1}]: 696 (s), 743 (m), 766 (w), 880 (m), 1001 (w), 1024 (m), 1038 (w), 1059 (w), 1076 (w), 1192 (w), 1227 (w), 1331 (w), 1412 (w), 1439 (m), 1468 (s), 1570 (w), 2886 (w), 2972 (w), 2990 (w).

UV/Vis (C_2H_6O): $\lambda_{max}(\varepsilon)$ = 383 (31100).

Anal calcd for $C_{50}H_{37}NO_2S$ [715.9]: C 83.89, H 5.21, N 1.96, S 4.48; Found: C 83.86, H 5.27, N 1.90, S 4.36.

(*Z*)-*N*-(3-(4-((2-(2-Oxo-2-phenylethylidene)benzo[*d*]thiazol-3(2*H*)-yl)methyl)phenyl)prop-2-yn-1-yl)-2-phenoxyacetamide (3l)

The synthesis was performed according to **GPII** using *n*-hexane/acetone 3:1 to 1:1 to 1:3 as an eluent to give 0.203 g (0.386 mmol, 76%) of the desired product **3l** as a yellow solid.

Mp: 101 °C.

R_f (*n*-hexane/acetone 4:1): 0.11.

^{1}H NMR (300 MHz, acetone-d_6): δ 3.58 (s, 2 H), 4.22 (dd, ^3J = 9.8 Hz, ^4J = 2.5 Hz, 1 H), 4.51 (d, ^4J = 2.5 Hz, 2 H), 5.65 (s, 2 H), 6.90 (s, 1 H), 6.94 – 7.01 (m, 4 H), 7.23 – 7.33 (m, 5 H), 7.38 – 7.46 (m, 6 H), 7.79 – 7.85 (m, 1 H), 7.95 – 7.99 (m, 2 H).

^{13}C NMR (75 MHz, acetone-d_6): δ 49.4 (CH_2), 67.6 (CH_2), 68.0 (CH_2), 82.0 (C_{quat}), 87.4 (C_{quat}), 88.2 (CH), 111.5 (CH), 115.6 (CH), 122.3 (CH), 122.4 (C_{quat}), 123.3 (CH), 123.9 (CH), 127.6 (CH), 127.7 (CH), 127.8 (C_{quat}), 127.9 (CH), 129.1 (CH), 130.37 (CH), 130.39 (CH), 131.6 (CH), 132.9 (CH), 135.2 (C_{quat}), 136.7 (C_{quat}), 140.5 (C_{quat}), 140.9 (C_{quat}), 158.7 (C_{quat}), 162.6 (C_{quat}), 168.4 (C_{quat}), 184.6 (C_{quat}).

MALDI-TOF (*m/z*): 531.3 ($C_{33}H_{26}N_2O_3S+H^+$).

IR $\tilde{\nu}$ [cm^{-1}]: 615 (m), 648 (m), 667 (m), 689 (s), 721 (m), 746 (s), 795 (m), 822 (m), 835 (m), 880 (s), 926 (w), 970 (w), 1001 (w), 1022 (m), 1045 (m), 1061 (m), 1082 (m), 1119 (w), 1138 (w), 1157 (w), 1175 (m), 1192 (m), 1229 (m), 1261 (m), 1292 (m), 1302 (m), 1331 (m), 1385 (w), 1414 (m), 1435 (s), 1456 (s), 1557 (w), 1568 (m), 1597 (m), 1645 (w), 1661 (m), 1728 (w), 1742 (w), 2116 (w), 2216 (w), 2251 (w), 2394 (w), 2853 (w), 2911 (w), 2961 (w), 3011 (w).

UV/Vis (C_2H_6O): $\lambda_{max}(\varepsilon)$ = 256 (34000), 382 (29900).

Anal calcd for $C_{33}H_{26}N_2O_3S$ [530.2]: C 74.69, H 4.94, N 5.28, S 6.04; Found: C 74.27, H 5.22, N 5.01, S 6.28.

(*Z*)-*N*-(3-(4-((2-(2-Oxo-2-phenylethylidene)benzo[*d*]thiazol-3(2*H*)-yl)methyl)phenyl)prop-2-yn-1-yl)thiophene-2-carboxamide (3m)

The synthesis was performed according to **GPII** using *n*-hexane/acetone 2:1 to 1:3 as an eluent to give 0.132 g (0.261 mmol, 52%) of the desired product **3m** as a yellow solid.

Mp: 230 °C (decomposition).

R_f (*n*-hexane/acetone 3:1): 0.17.

^{1}H NMR (300 MHz, acetone-d$_6$/CS$_2$ 5:1): δ 3.58 (s, 1 H), 4.51 (d, ^3J = 5.6 Hz, 2 H), 5.60 (s, 2 H), 6.82 (s, 1 H), 7.07 – 7.11 (m, 1 H), 7.21 – 7.30 (m, 3 H), 7.33 – 7.47 (m, 6 H), 7.63 (dd, ^3J = 5.0 Hz, ^4J = 1.1 Hz, 1 H), 7.70 (dd, ^3J = 3.9 Hz, ^4J = 1.1 Hz, 1 H), 7.75 (dt, ^3J = 7.8 Hz, ^4J = 1.1 Hz, 1 H), 7.91 – 8.04 (m, 3 H).

^{13}C NMR (75 MHz, acetone-d$_6$/CS$_2$ 5:1): δ 49.4 (CH$_2$), 65.1 (CH$_2$), 82.2 (C$_{quat}$), 88.3 (CH), 91.0 (C$_{quat}$), 111.3 (CH), 118.26 (CH), 123.2 (CH), 123.3 (CH), 123.9 (CH), 127.1 (C$_{quat}$), 127.5 (CH), 127.6 (CH), 127.9 (CH), 128.4 (CH), 128.6 (C$_{quat}$), 129.0 (CH), 131.3 (CH), 131.5 (CH), 132.9 (CH), 136.4 (C$_{quat}$), 140.2 (C$_{quat}$), 140.8 (C$_{quat}$), 161.6 (C$_{quat}$), 162.4 (C$_{quat}$), 167.7 (C$_{quat}$), 184.3 (C$_{quat}$).

MALDI-TOF (*m/z*): 507.2 ($C_{30}H_{22}N_2O_2S_2$+H^+).

IR $\tilde{\nu}$ [cm^{-1}]: 669 (m), 683 (m), 704 (m), 714 (s), 791 (w), 820 (m), 845 (m), 856 (m), 881 (m), 941 (w), 972 (w), 1024 (m), 1042 (m), 1061 (m), 1078 (w), 1092 (w), 1128 (m), 1157 (w), 1175 (m), 1198 (m), 1231 (m), 1281 (m), 1335 (m), 1356 (m), 1381 (w), 1416 (m), 1435 (s), 1470 (s), 1477 (s), 1495 (m), 1510 (m), 1537 (m), 1568 (m), 1599 (m), 1641 (m), 3069 (w), 3329 (w).

UV/Vis (C_2H_6O): $\lambda_{max}(\varepsilon)$ = 382 (33400).

Anal calcd for $C_{30}H_{22}N_2O_2S_2$ [506.1]: C 71.12, H 4.38, N 5.53, S 12.66; Found: C 71.41, H 4.26, N 5.46, S 12.26.

(Z)-2-((3-(4-((2-(2-Oxo-2-phenylethylidene)benzo[d]thiazol-3(2H)-yl)methyl)phenyl)prop-2-yn-1-yl)oxy)isoindolin-1,3-dione (3n)

The synthesis was performed according to **GPII** using *n*-hexane/acetone 3:1 to 1:2 to 1:4 as an eluent to give 0.140 g (0.258 mmol, 52%) of the desired product **3n** as a yellow solid.

Mp: 70 °C.

R_f (*n*-hexane/acetone 3:1): 0.09.

^{1}H NMR (300 MHz, acetone-d$_6$): δ 3.58 (s, 2 H), 5.66 (s, 2 H), 6.90 (s, 1 H), 7.25 – 7.33 (m, 2 H), 7.37 – 7.46 (m, 4 H), 7.78 – 7.92 (m, 10 H), 7.95 – 7.98 (m, 1 H).

^{13}C NMR (75 MHz, acetone-d$_6$): δ 49.4 (CH_2), 66.2 (CH_2), 67.6 (C_{quat}), 72.7 (C_{quat}), 74.9 (C_{quat}), 83.5 (C_{quat}), 88.2 (CH), 89.2 (C_{quat}), 111.5 (CH), 122.1 (CH), 123.3 (CH), 123.7 (CH), 123.9 (CH), 124.1 (CH), 124.2 (CH), 127.6 (C_{quat}), 127.8 (CH), 127.9 (CH), 129.1 (CH), 129.9 (CH), 131.6 (C_{quat}), 133.0 (CH), 135.2 (CH), 135.6 (CH), 135.7 (CH), 137.5 (C_{quat}), 140.5 (C_{quat}), 140.9 (C_{quat}), 162.6 (C_{quat}), 163.6 (C_{quat}), 163.8 (C_{quat}), 184.6 (C_{quat}), 188.4 (C_{quat}).

MALDI-TOF (*m/z*): 543.2 ($C_{33}H_{22}N_2O_4S+H^+$).

IR $\tilde{\nu}$ [cm^{-1}]: 606 (m), 629 (w), 667 (m), 698 (s), 721 (m), 748 (m), 785 (m), 876 (m), 970 (m), 1015 (m), 1042 (w), 1063 (w), 1082 (m), 1121 (m), 1157 (w), 1186 (m), 1231 (m), 1292 (w), 1327 (m), 1358 (m), 1373 (m), 1418 (w), 1431 (m), 1464 (m), 1495 (m), 1549 (w), 1568 (w), 1593 (m), 1724 (s), 1790 (m), 2853 (w), 3038 (w).

UV/Vis (C_2H_6O): $\lambda_{max}(\varepsilon)$ = 382 (15600).

Anal calcd for $C_{33}H_{22}N_2O_4S$ [542.1]: C 73.05, H 4.09, N 5.16, S 5.91; Found: C 72.87, H 3.80, N 5.48, S 5.61.

(*Z*)-2-(3-(4-((5-Chloro-1-tosyl-1*H*-indol-3-yl)ethinyl)benzyl)benzo[*d*]thiazol-2(3*H*)-ylidene)-1-phenylethan-1-one (3o)

The synthesis was performed according to **GPII** using *n*-hexane/acetone 4:1 to 3:1 as an eluent to give 0.247 g (0.368 mmol, 96%) of the desired product **3o** as a yellow solid.

Mp: 143 °C.

R_f (*n*-hexane/acetone 4:1): 0.15.

^{1}H NMR (300 MHz, acetone-d$_6$/CS$_2$ 5:2): δ 5.59 (s, 2 H), 6.78 (s, 1 H), 7.21 – 7.26 (m, 1 H), 7.29 – 7.46 (m, 12 H), 7.55 – 7.58 (m, 1 H), 7.63 (d, ^4J = 2.1 Hz, 1 H), 7.73 (d, ^3J = 7.7 Hz, 1 H), 7.84 – 7.92 (m, 6 H), 7.95 – 8.00 (m, 2 H).

^{13}C NMR (75 MHz, acetone-d$_6$/CS$_2$ 5:2): δ 21.9 (C_{quat}), 49.5 (CH_2), 105.2 (C_{quat}), 111.1 (CH_3), 115.8 (CH), 120.7 (CH), 123.2 (CH), 123.8 (CH), 126.5 (CH), 127.4 (CH), 127.6 (CH), 127.88 (CH), 127.91 (CH), 128.9 (CH), 130.6 (C_{quat}), 131.0 (CH), 131.1 (CH), 131.4 (CH), 132.7 (C_{quat}), 132.8 (CH), 133.3 (C_{quat}), 135.3 (C_{quat}), 136.6 (C_{quat}), 140.3 (C_{quat}), 140.7 (C_{quat}), 146.7 (C_{quat}), 162.2 (C_{quat}), 168.5 (C_{quat}).

MALDI-TOF (*m/z*): 673.2 ($C_{39}H_{27}{}^{37}ClN_2O_3S_2$+H$^+$), 671.2 ($C_{39}H_{27}{}^{35}ClN_2O_3S_2$+H$^+$), 418.2 ($C_{24}H_{17}{}^{37}ClNO_2S^+$), 416.2 ($C_{24}H_{17}{}^{35}ClNO_2S^+$).

IR $\tilde{\nu}$ [cm^{-1}]: 671 (s), 706 (w), 716 (w), 743 (w), 772 (w), 791 (w), 799 (w), 810 (w), 852 (w), 878 (w), 972 (w), 1061 (w), 1099 (w), 1119 (w), 1150 (w), 1177 (s), 1227 (w), 1277 (w), 1294 (w), 1335 (w), 1368 (w), 1325 (w), 1412 (w), 1439 (w), 1452 (w), 1470 (w), 1481 (s), 1497 (w), 1595 (w), 1676 (w), 1811 (w), 2023 (w), 3098 (w).

UV/Vis (C_2H_6O): $\lambda_{max}(\varepsilon)$ = 308 (19800), 382 (15200).

Anal calcd for $C_{39}H_{27}ClN_2O_3S_2$ [671.2]: C 69.79, H 4.05, N 4.17, S 9.55; Found: C 69.89, H 4.43, N 4.13, S 9.17.

(*Z*)-2-(3-(4-((10-Methyl-10*H*-phenothiazin-3-yl)ethynyl)benzyl)benzo[*d*]thiazol-2(3*H*)-ylidene)-1-phenylethan-1-one (3p)

The synthesis was performed according to **GPII** using *n*-hexane/acetone 4:1 to 3:1 as an eluent to give 0.190 g (0.329 mmol, 86%) of the desired product **3p** as a yellow solid.

Mp: 143 °C.

R_f (*n*-hexane/acetone 4:1): 0.28.

^{1}H NMR (300 MHz, acetone-d$_6$): δ 3.39 (s, 3 H), 5.62 (s, 2 H), 6.83 – 7.00 (m, 4 H), 7.10 (dd, ^3J = 7.5 Hz, ^4J = 1.6 Hz, 1 H), 7.15 – 7.27 (m, 3 H), 7.28 – 7.53 (m, 10 H), 7.76 (dt, ^3J = 7.7 Hz, ^4J = 0.9 Hz, 1 H), 7.91 – 8.00 (m, 2 H).

^{13}C NMR (75 MHz, acetone-d$_6$): δ 35.8 (CH_3), 49.5 (CH_2), 88.3 (CH), 89.7 (C_{quat}), 90.2 (C_{quat}), 111.4 (CH), 115.1 (CH), 115.5 (CH), 117.7 (C_{quat}), 123.2 (CH), 123.7 (CH), 123.8 (CH), 124.2 (C_{quat}), 127.5 (CH), 127.7 (CH), 128.0 (CH), 128.6 (CH), 129.0 (CH), 130.2 (CH), 131.5 (CH), 131.9 (CH), 132.6 (CH), 136.3 (CH), 140.5 (C_{quat}), 140.8 (C_{quat}), 145.9 (C_{quat}), 146.8 (C_{quat}), 162.4 (C_{quat}), 184.4 (C_{quat}).

MALDI-TOF (*m/z*): 579.2 ($C_{37}H_{26}N_2OS_2+H^+$), 326.3 ($C_{22}H_{16}NS^+$).

IR $\tilde{\nu}$ [cm^{-1}]: 667 (w), 691 (w), 702 (w), 719 (m), 745 (m), 795 (w), 820 (w), 854 (w), 878 (m), 926 (w), 1022 (w), 1038 (w), 1061 (w), 1092 (w), 1105 (w), 1140 (w), 1155 (w), 1179 (w), 1190 (w), 1225 (w), 1260 (w), 1290 (w), 1300 (w), 1333 (m), 1396 (w), 1412 (w), 1439 (m), 1460 (s), 1477 (s), 1510 (w), 1568 (w), 1595 (w), 2818 (w), 2880 (w), 2961 (w), 3057 (w).

UV/Vis (C_3H_6O): $\lambda_{max}(\varepsilon)$ = 272, 379 (40400).

Anal calcd for $C_{37}H_{26}N_2OS_2$ [578.8]: C 76.79, H 4.53, N 4.84, S 11.08; Found: C 76.68, H 4.70, N 4.69, S 10.81.

(*Z*)-1-(4-Fluorophenyl)-2-(3-(4-((4-(((*Z*)-2-(2-oxo-2-phenylethylidene)benzo[*d*]thiazol-3(2*H*)-yl)methyl)phenyl)ethynyl)benzyl)benzo[*d*]thiazol-2(3*H*)-ylidene)ethan-1-one (3q)

The synthesis was performed according to **GPII** using *n*-hexane/acetone 4:1 to 2:1 as an eluent followed by a second column chromatography using *n*-hexane/acetone 5:1 as an eluent to give 0.091 g (0.125 mmol, 42%) of the desired product **3q** as a yellow solid.

Mp: 250 °C.

R_f (*n*-hexane/acetone 4:1): 0.06.

^{1}H NMR (300 MHz, acetone-d$_6$/CS$_2$ 5:2): δ 5.59 (s, 4 H), 6.79 (s, 2 H), 7.04 – 7.14 (m, 2 H), 7.19 – 7.54 (m, 17 H), 7.73 (d, ^3J = 7.7 Hz, 2 H), 7.87 – 8.02 (m, 4 H).

·^{13}C NMR (75 MHz, acetone-d$_6$/CS$_2$ 5:2): δ 49.5 (CH$_2$), 87.9 (CH), 88.2 (CH), 90.3 (C$_{quat}$), 111.2 (CH), 111.3 (CH), 115.5 (CH), 115.8 (d, $^2J_{CF}$ = 22.0 Hz, CH), 123.2 (CH), 123.4 (C$_{quat}$), 123.8 (CH), 123.9 (CH), 127.40 (d, $^3J_{CF}$ = 8.0 Hz, CH), 127.43 (CH), 127.9 (CH), 128.9 (CH), 130.3 (CH), 130.4 (CH), 131.4 (d, CH), 132.8 (CH), 140.4 ($^4J_{CF}$ = 3.1 Hz, C$_{quat}$), 140.6 (C$_{quat}$), 140.7 (C$_{quat}$), 162.2 (C$_{quat}$), 162.3 (d, $^1J_{CF}$ = 252.1 Hz, C$_{quat}$), 162.5 (C$_{quat}$), 182.8 (C$_{quat}$).

MALDI-TOF (*m/z*): 727.2 ($C_{46}H_{31}FN_2O_2S_2$+H$^+$), 474.2 ($C_{31}H_{21}FNOS^+$), 456.3 ($C_{31}H_{22}NOS^+$).

IR $\tilde{\nu}$ [cm^{-1}]: 615 (w), 702 (w), 719 (m), 741 (m), 762 (w), 816 (w), 849 (w), 881 (m), 1063 (w), 1086 (w), 1153 (w), 1196 (w), 1223 (m), 1267 (w), 1302 (w), 1341 (w), 1412 (w), 1437 (m), 1458 (s), 1570 (w), 1597 (w), 2488 (w), 2507 (w), 2847 (w), 2930 (w), 3202 (w), 3213 (w), 3277 (w), 3397 (w), 3485 (w), 3505 (w), 3566 (w), 3647 (w), 3688 (w), 3815 (w), 3836 (w), 3854 (m), 3902 (w), 3979 (w).

UV/Vis (C_2H_6O): $\lambda_{max}(\varepsilon)$ = 288 (39000), 307 (30500), 382 (61500).

HPLC-HRMS (*m/z*): HRMS (ESI) calcd. for $C_{46}H_{31}FN_2O_2S_2$+H$^+$: 727.1884; Found: 727.1874. HPLC-pure.

(*Z*)-1-Phenyl-2-(3-(4-((trimethylsilyl)ethynyl)benzyl)benzo[*d*]thiazol-2(3*H*)-ylidenr)ethan-1-one (3r)

The synthesis was performed according to **GPII** using *n*-hexane/acetone 3:1 as an eluent to give 1.30 g (2.97 mmol, 99%) of the desired product **3r** as a yellow solid.

Mp: 202 °C (decomposition).

R_f (*n*-hexane/acetone 3:1): 0.32.

^{1}H NMR (300 MHz, acetone-d$_6$): δ 0.20 (s, 9 H), 5.66 (s, 2 H), 6.91 (s, 1 H), 7.23 – 7.28 (m, 1 H), 7.30 – 7.35 (m, 2 H), 7.38 – 7.49 (m, 7 H), 8.83 (dt, ^3J = 7.7 Hz, ^4J = 0.9 Hz, 1 H), 7.96 – 8.00 (m, 2 H).

^{13}C NMR (75 MHz, acetone-d$_6$): δ 0.1 (CH_3), 49.4 (CH_2), 88.9 (CH), 94.9 (C_{quat}), 105.5 (C_{quat}), 111.5 (CH), 123.27 (CH), 123.33 (CH), 123.9 (CH), 127.6 (CH), 127.7 (CH), 127.8 (C_{quat}), 127.9 (CH), 129.8 (CH), 131.6 (CH), 133.1 (CH), 137.2 (C_{quat}), 140.5 (C_{quat}), 140.9 (C_{quat}), 162.5 (C_{quat}), 184.6 (C_{quat}).

ESI-MS (m/z): 901 (($C_{27}H_{25}NOSSi)_2+Na^+$), 462 ($C_{27}H_{25}NOSSi+Na^+$), 440 ($C_{27}H_{25}NOSSi+H^+$).

IR $\tilde{\nu}$ [cm^{-1}]: 627 (m), 652 (m), 677 (m), 719 (s), 731 (s), 743 (m), 758 (m), 802 (m), 818 (m), 843 (m), 862 (m), 1022 (m), 1061 (m), 1094 (w), 1155 (w), 1182 (m), 1200 (m), 1233 (m), 1252 (m), 1300 (m), 1348 (m), 1410 (m), 1437 (m), 1458 (m), 1477 (s), 1497 (m), 1566 (m), 1595 (m), 1904 (w), 1979 (w), 2021 (w), 2029 (w), 2158 (m), 2220 (w), 2934 (w), 3046 (w).

UV/Vis (C_3H_6O): λ_{max} (ε) = 263 (37700), 381 (35500).

Anal calcd for $C_{27}H_{25}NOSSi$ [439.1]: C 73.76, H 5.73, N 3.19, S 7.29; Found: C 73.55, H 5.65, N 3.18, S 7.29.

(*Z*)-1-(4-Fluorophenyl)-2-(3-(4-((trimethylsilyl)ethynyl)benzyl)benzo[*d*]thiazol-2(3*H*)-ylidene)ethan-1-one (3s)

Synthetic procedure 1

The synthesis was performed according to **GPII** using *n*-hexane/acetone 5:1 as an eluent to give 0.230 g (0.500 mmol, 99%) of the desired product **3s** as a yellow solid.

Synthetic procedure 2

0.080 g (1.00 equiv., 0.06 mL, 0.500 mmol) of 4-fluorobenzoyl chloride and 0.245 g (1.10 equivs., 0.550 mmol) of benzylbenzothiazolium bromide were placed in a sintered, dry screw-cap *Schlenk*-tube under nitrogen atmosphere and stirred in 3 mL of dry 1,4-dioxane. To the reaction solution, 0.15 mL (2.20 equivs., 1.10 mmol) of triethylamine was added and stirred for 1 h at room temperature. The reaction solution was then heated to reflux for 22 h at 120 °C. Then, 7.00 mg (2.00 mol%, 0.010 mmol) bis(triphenylphosphane)palladium dichloride(II), 0.15 mL (2.20 equivs., 1.10 mmol) triethylamine, 4.00 mg (4.00 mol%, 0.020 mmol) copper(I) iodide, and 0.098 g (2.00 equivs., 0.14 mL, 1.00 mmol) TMSA **2q** were added and stirred for 2 h at 40 °C. Then, the crude product was adsorbed on Celite 545®. The crude product was purified by column chromatography, a mixture of *n*-hexane and acetone (5:1) was used as eluent. The product was then overcoated with *n*-hexane and suspended in an ultrasonic bath before the solid was filtered off and the product dried under high vacuum. Thus, 218 mg (0.477 mmol, 95%) of product **3s** was obtained as a yellow solid.

Mp: 102 °C.

R_f (*n*-hexane/acetone 4:1): 0.29.

^{1}H NMR (300 MHz, acetone-d$_6$): δ 0.20 (s, 9 H), 5.66 (s, 2 H), 6.90 (s, 1 H), 7.13 – 7.19 (m, 2 H), 7.23 – 7.32 (m, 3 H), 7.37 – 7.40 (m, 2 H), 7.44 – 7.48 (m, 2 H), 8.83 (dt, ^3J = 7.7 Hz, ^4J = 0.9 Hz, 1 H), 8.01 – 8.08 (m, 2 H).

^{13}C NMR (75 MHz, acetone-d$_6$): δ 0.1 (CH_3), 49.4 (CH_2), 87.9 (CH), 94.9 (C_{quat}), 105.4 (C_{quat}), 111.5 (CH), 115.7 (CH), 115.9 (d, $^2J_{CF}$ = 21.8 Hz, CH), 123.28 (CH), 123.34 (CH), 124.0 (CH), 127.6 (d, $^3J_{CF}$ = 7.9 Hz, CH), 127.7 (CH), 127.8 (C_{quat}), 130.4 (CH), 130. 5 (CH), 133.1 (CH), 136.98 (d, $^4J_{CF}$ = 3.5 Hz, C_{quat}), 137.02 (C_{quat}), 137.1 (C_{quat}), 140.8 (C_{quat}), 162.8 (d, $^1J_{CF}$ = 252.2 Hz, C_{quat}), 163.6 (C_{quat}), 166.9 (C_{quat}), 183.2 (C_{quat}).

MALDI-TOF (m/z): 458.2 ($C_{27}H_{24}FNOSSi+H^+$).

IR $\tilde{\nu}$ [cm^{-1}]: 610 (m), 627 (m), 660 (w), 700 (m), 743 (m), 762 (m), 841 (s), 862 (m), 881 (m), 1013 (w), 1043 (w), 1065 (m), 1086 (m), 1153 (m), 1192 (m), 1223 (m), 1248 (m), 1302 (w),

1331 (m), 1408 (m), 1454 (s), 1468 (s), 1506 (m), 1574 (m), 1599 (m), 2158 (w), 2849 (w), 2897 (w), 2926 (w), 2957 (w), 3069 (w).

UV/Vis (C_3H_6O): λ_{max} (ε) = 253 (61400), 264 (65500), 381 (35100).

Anal calcd for $C_{27}H_{24}FNOSSi$ [457.1]: C 70.86, H 5.29, N 3.06, S 7.01; Found: C 71.03, H 5.56, N 2.96, S 6.82.

(*Z*)-4-(2-(3-(4-((Trimethylsilyl)ethynyl)benzyl)benzo[*d*]thiazol-2(3*H*)-ylidene)acetyl)benzonitrile (3t)

The synthesis was performed according to **GPII** using *n*-hexane/acetone 3:1 to 1:1 as an eluent to give 0.378 g (0.814 mmol, 65%) of the desired product **3t** as a yellow solid.

Mp: 250 °C.

R_f (*n*-hexane/acetone 3:1): 0.17.

^{1}H NMR (300 MHz, acetone-d$_6$/CS$_2$ 5:1): δ 0.22 (s, 9 H), 5.68 (s, 2 H), 6.94 (s, 1 H), 7.26 – 7.34 (m, 3 H), 7.39 – 7.45 (m, 4 H), 7.77 – 7.83 (m, 3 H), 8.10 – 8.14 (m, 2 H).

^{13}C NMR (75 MHz, acetone-d$_6$/CS$_2$ 5:1): δ 0.1 (CH_3), 49.6 (CH_2), 88.4 (CH), 95.1 (C_{quat}), 105.5 (C_{quat}), 111.8 (CH), 114.7 (C_{quat}), 119.0 (C_{quat}), 123.4 (CH), 123.5 (CH), 124.3 (CH), 127.6 (CH), 127.8 (CH), 127.9 (C_{quat}), 128.6 (CH), 133.0 (CH), 133.1 (CH), 136.7 (C_{quat}), 140.7 (C_{quat}), 144.0 (C_{quat}), 163.6 (C_{quat}), 182.2 (C_{quat}).

ESI-MS (m/z): 464 ($C_{287}H_{24}N_2OSSi+H^+$).

IR $\tilde{\nu}$ [cm^{-1}]: 642 (m), 667 (m), 681 (m), 696 (m), 737 (s), 787 (m), 829 (m), 880 (w), 922 (m), 966 (w), 1003 (w), 1024 (m), 1042 (m), 1063 (m), 1082 (m), 1107 (w), 1140 (w), 1165 (m), 1188 (m), 1215 (w), 1283 (w), 1296 (m), 1304 (m), 1331 (m), 1360 (m), 1420 (m), 1449 (s), 1555 (w), 1566 (m), 1587 (w), 2887 (w), 2916 (w), 2972 (w), 3061 (w).

UV/Vis (C_3H_6O): λ_{max} (ε) = 262 (50300), 400 (38400).

Anal calcd for $C_{28}H_{24}N_2OSSi$ [464.1]: C 72.38, H 5.21, N 6.03, S 6.90; Found: C 72.36, H 5.22, N 5.92, S 6.86.

(2*Z*,2'*Z*)-2,2'-((((Pyridine-2,6-diylbis(ethyn-2,1-diyl))bis(4,1-phenylene))bis(methylene))bis(benzo[*d*]thiazol-3(3*H*)-yl-2(3*H*)-yliden))bis(1-phenylethan-1-one) (3u)

0.281 g (2.00 equivs, 0.600 mmol) (*Z*)-2-(3-(4-iodobenzyl)benzo[*d*]thiazol-2(3*H*)-ylidene)-1-phenylethan-1-one (**1a**), 0.008 g (4.00 mol%, 0.012 mmol) bis(triphenylphosphane) palladium(II) dichloride, 0.005 g (8.00 mol%, 0.024 mmol) copper(I) iodide, and 0.038 g (1.00 equiv., 0.300 mmol) of 2,6-diethynylpyridine (**2r**) were placed in a sintered, dry screw-cap *Schlenk*-tube under nitrogen atmosphere, and dissolved in 3 mL of dry 1,4-dioxane. After addition of 0.067 g (1.10 equivs., 0.092 mL, 0.660 mmol) of triethylamine, the mixture was stirred at 40 °C for 3 h. The product was then adsorbed on Celite 545® and purified by flash chromatography on silica gel using *n*-hexane/acetone 4:1 to 2:1 to 1:1 to 1:2 to 1:3 as an eluent to give 0.045 g (0.056 mmol, 19%) of the desired product **3u** as an orange solid and 0.052 g (0.111 mmol, 37%) of the product **3v** as an orange solid.

Mp: 139 °C (decomposition).

R_f (*n*-hexane/acetone 4:1): 0.06.

^{1}H NMR (300 MHz, acetone-d$_6$): δ 5.62 (s, 4 H), 6.80 (s, 2 H), 7.21 – 7.26 (m, 2 H), 7.31 – 7.43 (m, 14 H), 7.49 (d, ^3J = 7.9 Hz, 2 H), 7.57 – 7.61 (m, 4 H), 7.73 – 7.81 (m, 3 H), 7.89 – 7.92 (m, 4 H).

^{13}C NMR (75 MHz, acetone-d$_6$): δ 49.5 (CH_2), 88.3 (CH), 100.7 (C_{quat}), 111.2 (CH), 121.0 (CH), 122.4 (CH), 123.2 (CH), 123.8 (C_{quat}), 127.1 (CH), 127.4 (C_{quat}), 127.7 (CH), 127.9 (CH), 128.9 (CH), 131.4 (C_{quat}), 133.3 (C_{quat}), 137.4 (C_{quat}), 140.4 (C_{quat}), 140.7 (C_{quat}), 144.3 (C_{quat}), 162.3 (C_{quat}), 184.2 (C_{quat}).

MALDI-TOF (*m/z*): 810.3 ($C_{53}H_{35}N_3O_2S_2$+H^+), 557.2 ($C_{38}H_{25}N_2OS^+$).

IR $\tilde{\nu}$ [cm^{-1}]: 648 (w), 662 (w), 689 (w), 719 (m), 743 (m), 806 (w), 880 (m), 945 (w), 984 (w), 1022 (w), 1042 (w), 1061 (w), 1092 (w), 1119 (w), 1140 (w), 1155 (w), 1167 (w), 1177 (w), 1192 (w), 1227 (w), 1263 (w), 1292 (w), 1331 (w), 1398 (w), 1414 (w), 1435 (s), 1456 (s), 1553 (w), 1568 (w), 1595 (w), 2208 (w), 3051 (w).

UV/Vis (C_2H_6O): $\lambda_{max}(\varepsilon)$ = 271 (33200), 321 (21600), 381 (31500).

Anal calcd for $C_{53}H_{35}N_3O_2S_2$ [810.0]: C 78.59, H 4.36, N 5.19, S 7.92; Found: C 78.46, H 4.66, N 4.91, S 7.59.

(*Z*)-2-(3-(4-((6-Ethynylpyridine-2-yl)ethinyl)benzyl)benzo[*d*]thiazol-2(3*H*)-ylidene)-1-phenylethan-1-one (3v)

Mp: 93 °C.

R_f (*n*-hexane/acetone 4:1): 0.12.

^{1}H NMR (300 MHz, acetone-d$_6$): δ 3.82 (s, 1 H), 5.71 (s, 2 H), 6.94 (s, 1 H), 7.24 – 7.30 (m, 1 H), 7.39 – 7.47 (m, 7 H), 7.49 – 7.58 (m, 1 H), 7.60 – 7.67 (m, 3 H), 7.80 – 7.88 (m, 2 H), 7.97 – 8.06 (m, 2 H).

^{13}C NMR (75 MHz, acetone-d$_6$): δ 49.5 (CH_2), 79.1 (CH), 88.3 (CH), 89.1 (C_{quat}), 111.5 (CH), 122.2 (C_{quat}), 123.3 (CH), 124.0 (CH), 127.6 (CH), 127.8 (CH), 127.9 (CH), 128.0 (CH), 129.1 (CH), 131.6 (CH), 133.3 (CH), 137.9 (C_{quat}), 138.0 (CH), 140.5 (C_{quat}), 140.9 (C_{quat}), 143.7 (C_{quat}), 144.3 (C_{quat}), 162.6 (C_{quat}), 184.6 (C_{quat}).

MALDI-TOF (*m/z*): 469.2 ($C_{31}H_{20}N_2OS$+H^+).

IR $\tilde{\nu}$ [cm^{-1}]: 610 (w), 656 (w), 667 (m), 687 (w), 721 (m), 729 (m), 808 (m), 880 (m), 930 (w), 972 (w), 986 (w), 1022 (w), 1042 (w), 1061 (w), 1092 (w), 1109 (w), 1155 (w), 1167 (w), 1179 (w), 1192 (w), 1229 (w), 1265 (w), 1292 (w), 1302 (w), 1331 (w), 1412 (w), 1439 (s), 1468 (s), 1495 (w), 1555 (w), 1568 (w), 1595 (w), 1668 (w), 2104 (w), 2212 (w), 2922 (w), 3030 (w), 3053 (w).

UV/Vis (C_2H_6O): $\lambda_{max}(\varepsilon)$ = 284 (21600), 310 (17500), 382 (15700).

Anal calcd for $C_{31}H_{20}N_2OS$ [468.6]: C 79.46, H 4.30, N 5.98, S 6.84; Found: C 79.26, H 4.16, N 5.91, S 6.53.

4.2 General procedure III (GPIII) for the deprotection of alkynylated aroyl-*S,N*-ketene acetals 4

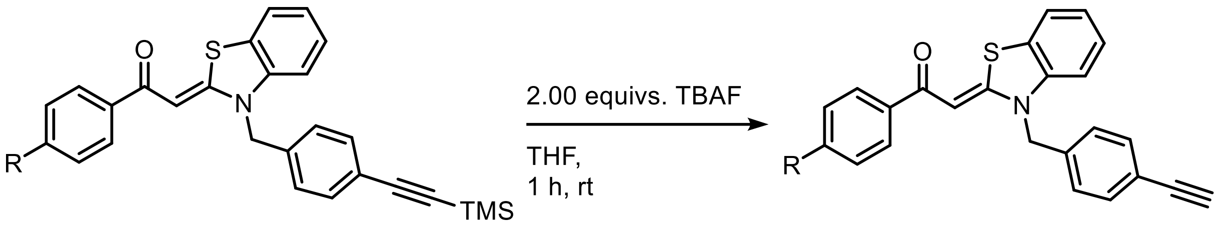

3r-t **4a-b**

Figure S7. Synthesis of alkynylated aroyl-*S*,*N*-ketene acetals **4**.

1.00 equiv. of TMS-protected aroyl-*S*,*N*-ketene acetal **3** and 2.00 equivs. of tetra-*N*-butylammonium fluoride were placed in a 50 mL round bottom flask, dissolved in 6 mL/mmol of THF, and stirred at room temperature for 1 h. Saturated ammonium chloride solution was added to the solution to stop the reaction and the mixture alongside with 30 mL of dichloromethane was transferred to a separatory funnel. The organic phase was separated, and the aqueous phase was extracted three times with 20 mL of dichloromethane. The combined organic phases were dried with magnesium sulfate, the desiccant was filtered off, and the crude product was adsorbed on Celite 545®. The crude product was purified by column chromatography using a mixture of n hexane and acetone (4:1) as eluent. The product was then overcoated with *n*-hexane and suspended in an ultrasonic bath before the solid was filtered off and the product dried under high vacuum.

Table S8. Experimental details for the synthesis of deprotected alkynylated aroyl-*S*,*N*-ketene acetals **4**.

entry	TMS-protected alkyne **3** [g] ([mmol])	TBAF [g] ([mmol])	yield of product **4** [g] (%)
1	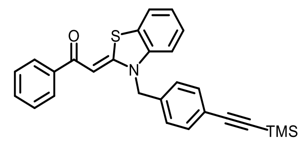 0.930 (2.12) of **3r**	0.989 (4.24)	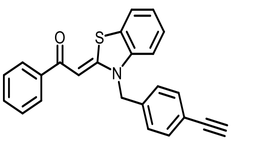 0.707 (91) of **4a**
2	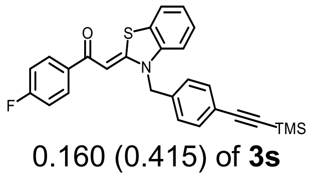 0.160 (0.415) of **3s**	0.217 (0.930)	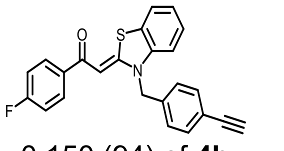 0.150 (94) of **4b**
3	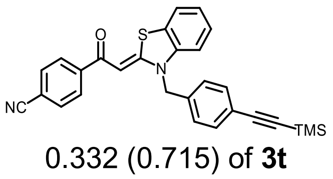 0.332 (0.715) of **3t**	0.334 (1.43)	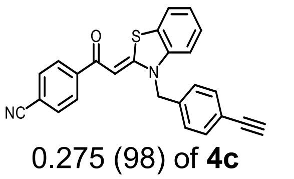 0.275 (98) of **4c**

4.2.1 Spectroscopic data

(*Z*)-2-(3-(4-Ethynylbenzyl)benzo[*d*]thiazol-2(3*H*)-ylidene)-1-phenylethan-1-one (4a)

The synthesis was performed according to **GPIII** to give 0.707 g (1.93 mmol, 91%) of the desired product **4a** as a yellow solid.

Mp: 198 °C.

R_f (*n*-hexane/acetone 3:1): 0.45.

^{1}H NMR (300 MHz, acetone-d$_6$): δ 3.64 (s, 1 H), 5.67 (s, 2 H), 6.92 (s, 1 H), 7.23 – 7.29 (m, 1 H), 7.32 – 7.36 (m, 2 H), 7.38 – 7.51 (m, 7 H), 7.80 (dt, ^3J = 7.7 Hz, ^4J = 1.0 Hz, 1 H), 7.96 – 8.00 (m, 2 H).

^{13}C NMR (75 MHz, acetone-d$_6$): δ 49.4 (CH$_2$), 79.5 (C_{quat}), 83.7 (C_{quat}), 88.2 (CH), 111.5 (CH), 122.6 (CH), 123.3 (CH), 123.9 (CH), 127.6 (CH), 127.7 (CH), 127.8 (C_{quat}), 129.1 (CH), 131.6 (CH), 133.3 (CH), 137.3 (C_{quat}), 140.5 (C_{quat}), 140.9 (C_{quat}), 162.6 (C_{quat}), 184.6 (C_{quat}).

MALDI-TOF (*m/z*): 368.1 ($C_{24}H_{17}NOS+H^+$).

IR $\tilde{\nu}$ [cm^{-1}]: 627 (m), 646 (m), 675 (s), 691 (s), 719 (s), 737 (s), 797 (m), 816 (m), 878 (s), 928 (w), 951 (w), 972 (w), 1001 (m), 1020 (m), 1043 (m), 1059 (m), 1090 (m), 1134 (m), 1153 (m), 1179 (m), 1190 (m), 1229 (m), 1261 (m), 1294 (m), 1304 (m), 1329 (m), 1339 (m), 1375 (w), 1414 (m), 1431 (s), 1456 (s), 1477 (s), 1497 (m), 1558 (m), 1570 (m), 1595 (w), 1892 (w), 2151 (w), 2160 (w), 2270 (w), 2855 (w), 2930 (w), 2961 (w), 3034 (w), 3256 (w).

UV/Vis (C_3H_6O): λ_{max} (ε) = 251 (40400), 382 (41300).

Anal calcd for $C_{24}H_{17}NOS$ [367.1]: C 78.45, H 4.66, N 3.81, S 8.72; Found: C 78.21, H 4.73, N 3.64, S 8.54.

(*Z*)-2-(3-(4-Ethynylbenzyl)benzo[*d*]thiazol-2(3*H*)-ylidene)-1-(4-fluorophenyl)ethan-1-one (4b)

The synthesis was performed according to **GPIII** to give 0.150 g (0.390 mmol, 94%) of the desired product **4b** as a yellow solid.

Mp: 145 °C.

R_f (*n*-hexane/acetone 4:1): 0.28.

^{1}H NMR (300 MHz, acetone-d$_6$): δ 3.51 (s, 1 H), 5.54 (s, 2 H), 6.78 (s, 1 H), 7.00 – 7.06 (m, 2 H), 7.10 – 7.15 (m, 1 H), 7.17 – 7.21 (m, 2 H), 7.25 – 7.27 (m, 2 H), 7.34 – 7.38 (m, 2 H), 7.67 (dt, ^3J = 7.8 Hz, ^4J = 0.9 Hz 1 H), 7.89 – 7.94 (m, 2 H).

^{13}C NMR (75 MHz, acetone-d$_6$): δ 49.4 (CH_2), 79.6 (C_{quat}), 83.7 (C_{quat}), 87.9 (CH), 111.6 (CH), 115.7 (CH), 116.0 (d, $^2J_{CF}$ = 20.9 Hz, CH), 122.6 (CH), 123.3 (CH), 124.0 (CH), 127.6 (d, $^3J_{CF}$ = 8.6 Hz, CH), 127.7 (CH), 127.8 (C_{quat}), 130.4 (CH), 130.5 (CH), 133.3 (CH), 137.00 (d, $^4J_{CF}$ = 3.4 Hz, C_{quat}), 137.04 (C_{quat}), 137.2 (C_{quat}), 140.9 (C_{quat}), 162.8 (d, $^1J_{CF}$ = 248.7 Hz, C_{quat}), 163.6 (C_{quat}), 166.9 (C_{quat}), 183.2 (C_{quat}).

MALDI-TOF (m/z): 386.3 ($C_{24}H_{16}FNOS+H^+$).

IR $\tilde{\nu}$ [cm^{-1}]: 606 (m), 631 (m), 654 (m), 679 (m), 719 (m), 745 (s), 766 (s), 791 (w), 808 (m), 826 (w), 835 (m), 851 (m), 881 (s), 928 (w), 948 (w), 970 (w), 999 (w), 1020 (m), 1045 (m), 1063 (m), 1084 (m), 1119 (w), 1136 (w), 1153 (s), 1188 (s), 1223 (s), 1261 (w), 1290 (m), 1304 (m), 1329 (m), 1344 (m), 1404 (m), 1449 (s), 1466 (s), 1477 (s), 1506 (m), 1537 (w), 1562 (m), 1575 (m), 1599 (m), 2392 (w), 3065 (w), 3250 (w).

UV/Vis (C_3H_6O): λ_{max} (ε) = 263 (74800), 386 (76200).

Anal calcd for $C_{24}H_{16}FNOS$ [385.1]: C 74.79, H 4.18, N 3.63, S 8.32; Found: C 74.69, H 4.42, N 3.49, S 8.25.

(*Z*)-4-(2-(3-(4-Ethynylbenzyl)benzo[*d*]thiazol-2(3*H*)-ylidene)acetyl)benzonitrile (4c)

The synthesis was performed according to **GPIII** to give 0.275 g (0.702 mmol, 98%) of the desired product **4c** as a yellow solid.

Mp: 216 °C.

R_f (*n*-hexane/acetone 3:1): 0.37.

^{1}H NMR (300 MHz, acetone-d$_6$/CS$_2$ 5:1): δ 3.46 (s, 1 H), 5.61 (s, 2 H), 6.86 (s, 1 H), 7.21 – 7.27 (m, 3 H), 7.31 – 7.36 (m, 2 H), 7.38 – 7.43 (m, 2 H), 7.69 – 7.76 (m, 3 H), 8.03 – 8.07 (m, 2 H).

^{13}C NMR (75 MHz, acetone-d$_6$/CS$_2$ 5:1): δ 49.5 (CH$_2$), 67.6 (CH), 79.7 (C_{quat}), 83.7 (C_{quat}), 88.3 (CH), 111.6 (CH), 114.7 (CH), 118.8 (CH), 122.7 (CH), 123.3 (CH), 124.2 (CH), 127.5 (CH), 127.7 (CH), 127.9 (C_{quat}), 128.6 (CH), 132.8 (CH), 133.3 (CH), 136.6 (C_{quat}), 140.5 (C_{quat}), 143.8 (C_{quat}), 163.4 (C_{quat}), 182.0 (C_{quat}).

ESI-MS (m/z): 393 ($C_{25}H_{16}N_2OS + H^+$).

IR $\tilde{\nu}$ [cm^{-1}]: 652 (m), 719 (m), 746 (s), 806 (s), 874 (m), 968 (w), 1022 (m), 1057 (w), 1105 (m), 1132 (m), 1152 (m), 1167 (m), 1242 (m), 1256 (m), 1283 (m), 1312 (m), 1333 (m), 1373 (m), 1410 (w), 1456 (s), 1506 (m), 1599 (w), 1667 (m), 1705 (m), 1744 (w), 2851 (m), 2920 (m), 2955 (w).

UV/Vis (C_3H_6O): λ_{max} (ε) = 401 (28300).

Anal calcd for $C_{25}H_{16}N_2OS$ [392.1]: C 76.51, H 4.11, N 7.14, S 8.14; Found: C 76.58, H 4.01, N 6.95, S 7.91.

5 Synthesis and analytical data of triazole aroyl-*S*,*N*-ketene acetals 6

5.1 General procedure IV (GPIV) for the synthesis of triazole aroyl-*S*,*N*-ketene acetals 6

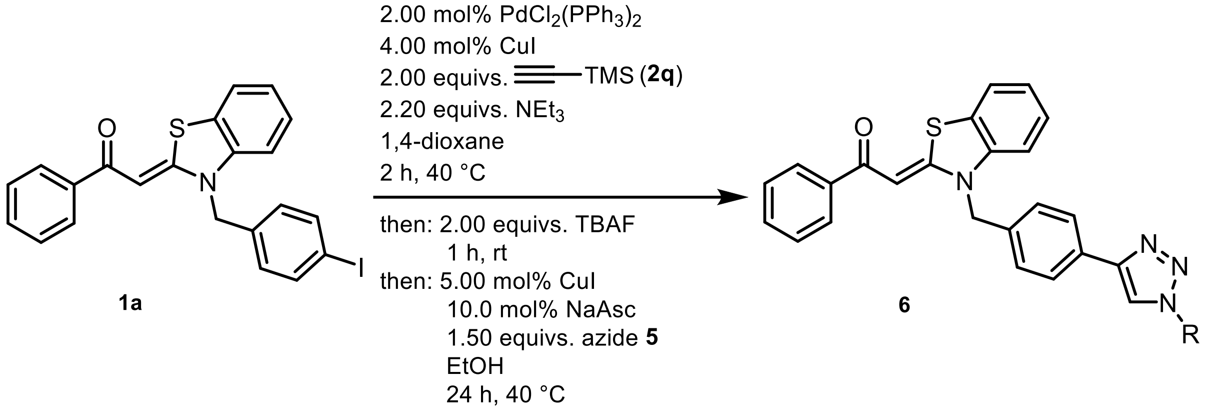

Figure S8. Synthesis of triazole aroyl-*S*,*N*-ketene acetals **4**.

0.094 g (1.00 equiv, 0.200 mmol) (*Z*)-2-(3-(4-iodobenzyl)benzo[*d*]thiazol-2(3*H*)-ylidene)-1-phenylethan-1-one (**1a**), 0.003 g (2.00 mol%, 0.004 mmol) bis(triphenylphosphane) palladium(II) dichloride, 0.001 g (4.00 mol%, 0.009 mmol) copper iodide and 0.039 g (2.00 equivs., 0.057 mL, 0.400 mmol) of TMSA (**2q**) were placed in a sintered, dry screw-cap *Schlenk*-tube under nitrogen atmosphere and dissolved in 2 mL of dry 1,4-dioxane. Subsequently, 0.040 g (2.20 equivs., 0.056 mL, 0.400 mmol) of triethylamine was added and the mixture was stirred at 40 °C for 2 h. Subsequently, 0.140 mL (2.00 equivs., 0.400 mmol) of tetra-*N*-butylammonium fluoride (75% in water) was added in nitrogen atmosphere and the reaction mixture was stirred at room temperature for 1 h. The reaction mixture was allowed to cool to room temperature and after thin layer chromatographic control, 0.002 g (5.00 mol%, 0.018 mmol) copper iodide, 0.004 g (10.0 mol%, 0.02 mmol) sodium ascorbate, 1.50 equivs. (0.300 mmol) azide **5**, and 1 mL ethanol were added and the reaction mixture was stirred at 40 °C for 24 h. The product was then adsorbed on Celite® and the solvent was removed under reduced pressure. The crude product was purified by column chromatography on silica gel (*n*-hexane/acetone) and then suspended several times with *n*-hexane.

Table S9. Experimental details for the synthesis of triazole aroyl-*S*,*N*-ketene acetals **6**.

entry	azide **5**<br>[g] ([mmol])	yield of product **6**<br>[g] (%)
1	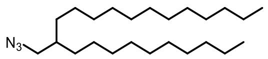<br>0.083 (0.300) of **5a**	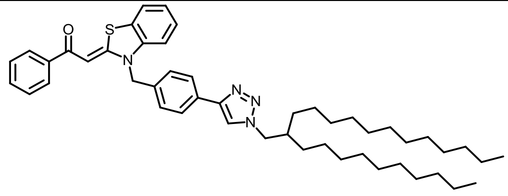<br>0.124 (83) of **6a**
2	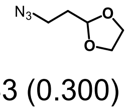<br>0.043 (0.300) of **5b**	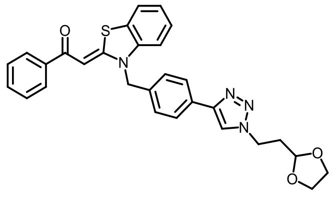<br>0.061 (60) of **6b**
3	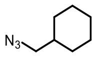<br>0.042 (0.300) of **5c**	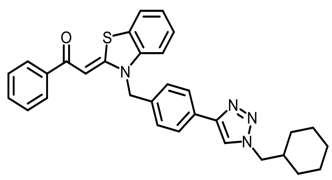<br>0.039 (39) of **6c**
4	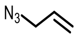<br>0.025 (0.300) of **5d**	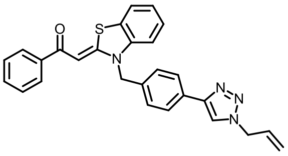<br>0.078 (87) of **6d**
5	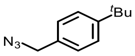<br>0.057 (0.300) of **5e**	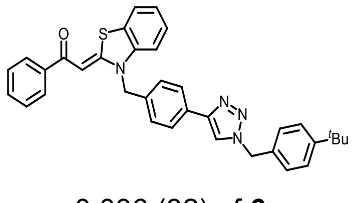<br>0.036 (32) of **6e**
6[(a)]	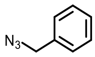<br>0.080 (0.675) of **5f**	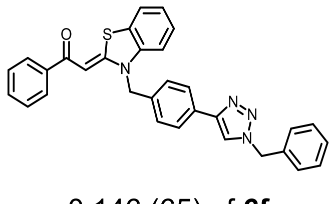<br>0.146 (65) of **6f**
7	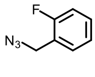<br>0.045 (0.300) of **5g**	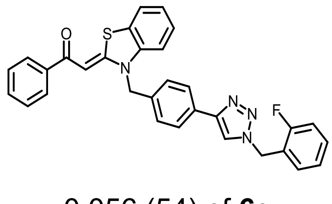<br>0.056 (54) of **6g**

Table S9 continued.

entry	azide **5** [g] ([mmol])	yield of product **6** [g] (%)
8	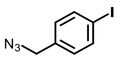 0.078 (0.300) of **5h**	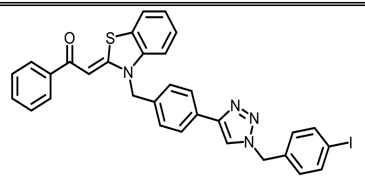 0.111 (89) of **6h**
9	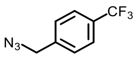 0.060 (0.300) of **5i**	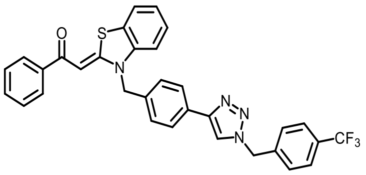 0.085 (75) of **6i**
10	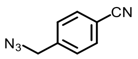 0.047 (0.300) of **5j**	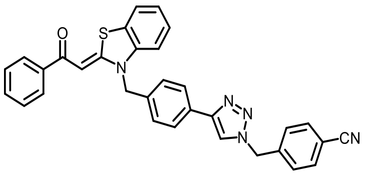 0.095 (90) of **6j**
11	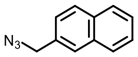 0.055 (0.300) of **5k**	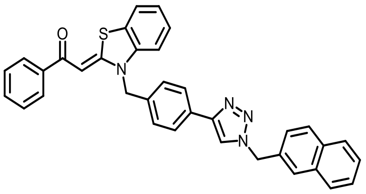 0.114 (95) of **6k**

(a): Aroyl-*S,N*-ketene acetal **1a**: 0.211 g (0.450 mmol), $Pd(PPh_3)_2Cl_2$: 0.007 g (0.009 mmol), CuI: 0.004 g (0.018 mmol), NEt_3: 0.091 g (0.125 mL, 0.900 mmol), 3 mL 1,4-dioxane, TBAF (75 % in H_2O): 0.314 mL (0.900 mmol), CuI: 0.005 g (0.0225 mmol), NaAsc: 0.009 g (0.045 mmol), 1 mL EtOH.

5.1.1 Spectroscopic data

(*Z*)-2-(3-(4-(1-(2-Decyltetradecyl)-1*H*-1,2,3-triazol-4-yl)benzyl)benzo[*d*]thiazol-2(3*H*)-ylidene)-1-phenylethan-1-one (6a)

The synthesis was performed according to **GPIV** using *n*-hexane/acetone 4:1 as an eluent to give 0.124 g (0.166 mmol, 83%) of the desired product **6a** as a yellow resin.

R_f (*n*-hexane/acetone 4:1): 0.43.

^{1}H NMR (300 MHz, acetone-d$_6$): δ 0.83 – 0.89 (m, 6 H), 1.25 – 1.36 (m, 41 H), 4.35 (d, ^3J = 6.9 Hz, 2 H), 5.66 (s, 2 H), 6.96 (s, 1 H), 7.23 – 7.29 (m, 1 H), 7.37 – 7.48 (m, 7 H), 7.80 (d, ^3J = 7.7 Hz, 1 H), 7.88 (d, ^3J = 8.4 Hz, 2 H), 7.99 (dd, ^3J = 7.9 Hz, ^4J = 2.2 Hz, 2 H), 8.30 (s, 1 H).

^{13}C NMR (75 MHz, acetone-d$_6$): δ 14.4 (CH_2), 23.32 (CH_2), 23.33 (CH_2), 26.9 (CH_2), 30.2 (CH_2), 30.5 (CH_2), 31.9 (CH_2), 32.61 (CH_2), 32.63 (CH_2), 39.8 (CH), 49.6 (CH_2), 54.3 (CH_2), 88.3 (CH), 111.6 (CH), 121.9 (CH), 123.2 (CH), 123.9 (CH), 126.7 (CH), 127.6 (CH), 127.9 (C_{quat}), 127.96 (CH), 128.02 (CH), 129.1 (CH), 131.6 (CH), 132.0 (C_{quat}), 135.7 (C_{quat}), 140.6 (C_{quat}), 141.0 (C_{quat}), 147.2 (C_{quat}), 162.6 (C_{quat}), 184.6 (C_{quat}).

MALDI-TOF (*m/z*): 747.5 ($C_{48}H_{66}N_4OS+H^+$).

IR $\tilde{\nu}$ [cm^{-1}]: 667 (w), 685 (m), 704 (m), 716 (s), 743 (s), 797 (m), 820 (m), 849 (w), 880 (s), 974 (m), 1022 (m), 1045 (m), 1061 (m), 1138 (w), 1155 (w), 1179 (m), 1196 (m), 1229 (m), 1269 (w), 1294 (w), 1312 (m), 1331 (m), 1342 (m), 1377 (w), 1416 (m), 1437 (s), 1470 (s), 1495 (s), 1568 (m), 1597 (m), 1674 (w), 2671 (w), 2851 (m), 2922 (m).

UV/Vis (C_3H_6O): λ_{max} (ε) = 383 (29100).

Anal calcd for $C_{48}H_{66}N_4OS$ [746.5]: C 77.27, H 8.78, N 7.51, S 4.30; Found: C 77.11, H 8.59, N 7.39, S 4.45.

(*Z*)-2-(3-(4-(1-(2-(1,3-Dioxolan-2-yl)ethyl)-1*H*-1,2,3-triazol-4-yl)benzyl)benzo[*d*]thiazol-2(3*H*)-ylidene)-1-phenylethan-1-one (6b)

The synthesis was performed according to **GPIV** using *n*-hexane/acetone 4:1 as an eluent to give 0.061 g (0.120 mmol, 60%) of the desired product **6b** as a yellow solid.

Mp: 200 °C.

R_f (*n*-hexane/acetone 4:1): 0.13.

^{1}H NMR (300 MHz, acetone-d$_6$/CS$_2$ 5:1): δ 1.84 – 1.93 (m, 2 H), 2.22 – 2.28 (m, 2 H), 3.42 (dt, ^3J = 7.3 Hz, ^4J = 1.8 Hz, 2 H), 4.50 (tt, ^3J = 7.3 Hz, ^4J = 1.0 Hz, 2 H), 4.90 (td, ^3J = 4.3 Hz, ^4J = 1.8 Hz, 1 H), 5.59 (s, 2 H), 6.85 (s, 1 H), 7.21 – 7.28 (m, 1 H), 7.33 – 7.46 (m, 7 H), 7.75 (dt, ^3J = 7.7 Hz, ^4J = 1.0 Hz, 1 H), 7.82 – 7.86 (m, 2 H), 7.91 – 7.96 (m, 2 H), 8.23 (s, 1 H).

^{13}C NMR (75 MHz, acetone-d$_6$/CS$_2$ 5:1): δ 35.0 (CH_2), 45.9 (CH), 49.6 (CH_2), 65.7 (CH_2), 88.3 (CH), 102.3 (CH), 111.4 (CH), 121.3 (CH), 123.2 (CH), 123.8 (CH), 126.7 (CH), 127.4 (CH), 127.8 (C_{quat}), 127.9 (CH), 128.0 (CH), 128.9 (CH), 131.4 (CH), 131.9 (CH), 135.3 (C_{quat}), 140.5 (C_{quat}), 140.9 (C_{quat}), 147.1 (C_{quat}), 162.4 (C_{quat}), 184.2 (C_{quat}).

MALDI-TOF (*m/z*): 511.2 ($C_{29}H_{26}N_4O_3S$+H^+).

IR $\tilde{\nu}$ [cm^{-1}]: 635 (w), 654 (m), 667 (m), 689 (m), 704 (m), 716 (s), 739 (s), 799 (m), 812 (m), 837 (w), 880 (s), 899 (m), 921 (w), 947 (m), 972 (m), 1013 (m), 1043 (m), 1061 (m), 1074 (m), 1092 (m), 1128 (m), 1179 (m), 1194 (m), 1227 (m), 1267 (m), 1288 (m), 1339 (m), 1346 (m), 1375 (w), 1393 (m), 1416 (m), 1429 (m), 1458 (s), 1470 (s), 1497 (m), 1568 (m), 1595 (m), 2097 (m), 2745 (w), 2862 (w), 2876 (w), 2930 (w), 2957 (w), 3069 (w), 3134 (w).

UV/Vis (C_3H_6O): λ_{max} (ε) = 381 (24400).

Anal calcd for $C_{29}H_{26}N_4O_3S$ [510.2]: C 68.22, H 5.13, N 10.97, S 6.28; Found: C 68.22, H 5.41, N 10.74, S 6.60.

(*Z*)-2-(3-(4-(1-(Cyclohexylmethyl)-1*H*-1,2,3-triazol-4-yl)benzyl)benzo[*d*]thiazol-2(3*H*)-ylidene)-1-phenylethan-1-one (6c)

The synthesis was performed according to **GPIV** using *n*-hexane/acetone 4:1 to 1:1 as an eluent to give 0.039 g (0.077 mmol, 39%) of the desired product **6c** as a yellow solid.

Mp: 211 °C.

R_f (*n*-hexane/acetone 4:1): 0.13.

^{1}H NMR (300 MHz, acetone-d$_6$/CS$_2$ 5:1): δ 0.98 – 1.32 (m, 5 H), 1.62 – 1.77 (m, 5 H), 1.87 – 1.97 (m, 1 H), 4.24 (d, ^3J = 7.5 Hz, 2 H), 5.60 (s, 2 H), 6.86 (s, 1 H), 7.21 – 7.27 (m, 1 H), 7.32 – 7.48 (m, 7 H), 7.75 (dt, ^3J = 7.5 Hz, ^4J = 1.0 Hz, 1 H), 7.84 (d, ^3J = 8.3 Hz, 2 H), 7.92 – 7.96 (m, 2 H), 8.18 (s, 1 H).

^{13}C NMR (75 MHz, acetone-d$_6$/CS$_2$ 5:1): δ 26.5 (CH), 27.1 (CH$_2$), 31.2 (CH$_2$), 39.6 (CH$_2$), 49.6 (CH$_2$), 56.7 (CH$_2$), 88.3 (CH), 111.4 (CH), 121.6 (CH), 123.2 (CH), 123.8 (CH), 126.7 (CH), 127.5 (CH), 127.88 (C$_{quat}$), 127.94 (CH), 128.0 (CH), 129.0 (CH), 131.4 (CH), 132.0 (C$_{quat}$), 135.4 (C$_{quat}$), 140.48 (C$_{quat}$), 140.50 (C$_{quat}$), 140.9 (C$_{quat}$), 147.0 (C$_{quat}$), 162.36 (C$_{quat}$), 162.41 (C$_{quat}$), 184.3 (C$_{quat}$).

MALDI-TOF (*m/z*): 507.2 ($C_{31}H_{30}N_4OS+H^+$).

IR $\tilde{\nu}$ [cm^{-1}]: 610 (m), 652 (m), 718 (s), 743 (s), 799 (m), 816 (m), 851 (w), 878 (s), 922 (w), 976 (m), 1024 (m), 1047 (m), 1061 (m), 1092 (w), 1138 (w), 1155 (m), 1179 (m), 1198 (m), 1229 (m), 1271 (m), 1294 (m), 1308 (m), 1333 (m), 1342 (m), 1416 (m), 1439 (s), 1460 (s), 1470 (s), 1495 (s), 1566 (m), 1599 (m2851 (w), 2926 (w).

UV/Vis (C_3H_6O): λ_{max} (ε) = 382 (37800).

Anal calcd for $C_{31}H_{30}N_4OS$ [506.2]: C 73.49, H 5.97, N 11.06, S 6.33; Found: C 73.26, H 5.83, N 10.98, S 6.49.

(*Z*)-2-(3-(4-(1-Allyl-1*H*-1,2,3-triazol-4-yl)benzyl)benzo[*d*]thiazol-2(3*H*)-ylidene)-1-phenylethan-1-one (6d)

The synthesis was performed according to **GPIV** using *n*-hexane/acetone 4:1 to 1:1 as an eluent to give 0.078 g (0.173 mmol, 87%) of the desired product **6d** as a yellow solid.

Mp: 194 °C.

R_f (*n*-hexane/acetone 4:1): 0.09.

^{1}H NMR (300 MHz, acetone-d$_6$): δ 5.06 (dt, ^3J = 6.2 Hz, ^4J = 1.5 Hz, 2 H), 5.25 (dq, ^3J = 6.8 Hz, ^4J = 1.5 Hz, 1 H), 5.30 (t, ^4J = 1.6 Hz, 2 H), 5.66 (s, 2 H), 6.96 (s, 1 H), 7.23 – 7.28 (m, 1 H), 7.37 – 7.47 (m, 7 H), 7.75 (dd, ^3J = 7.8 Hz, ^4J = 1.2 Hz, 1 H), 7.84 (d, ^3J = 8.5 Hz, 2 H), 7.99 (dd, ^3J = 7.5 Hz, ^4J = 2.1 Hz, 2 H), 8.27 (s, 1 H).

^{13}C NMR (75 MHz, acetone-d$_6$): δ 49.5 (CH_2), 53.0 (CH_2), 88.2 (CH), 111.6 (CH), 119.3 (CH), 121.4 (CH), 123.3 (CH), 123.9 (CH), 126.8 (CH), 127.6 (CH), 127.9 (C_{quat}), 128.0 (CH), 129.1 (CH), 131.6 (CH), 131.8 (C_{quat}), 133.4 (CH), 135.9 (C_{quat}), 140.6 (C_{quat}), 141. 0 (C_{quat}), 147.5 (C_{quat}), 162.6 (C_{quat}), 184.6 (C_{quat}).

MALDI-TOF (*m/z*): 451.2 ($C_{27}H_{22}N_4OS+H^+$).

IR $\tilde{\nu}$ [cm^{-1}]: 615 (m), 654 (m), 675 (m), 691 (m), 704 (m), 714 (s), 743 (s), 775 (m), 799 (m), 837 (w), 853 (w), 878 (s), 924 (w), 939 (m), 968 (w), 962 (m), 1001 (w), 1022 (m), 1047 (m), 1061 (m), 1076 (m), 1092 (m), 1136 (w), 1153 (m), 1179 (m), 1198 (m), 1227 (m), 1261 (m), 1292 (m), 1312 (m), 1333 (m), 1385 (w), 1414 (m), 1439 (s), 1460 (s), 1468 (s), 1495 (m), 1522 (w), 1564 (m), 1595 (m), 1722 (w), 2795 (w), 2857 (w), 2901 (w), 2926 (w), 2961 (w), 3011 (w), 3046 (w), 3073 (w), 3096 (w).

UV/Vis (C_3H_6O): λ_{max} (ε) = 382 (33900).

Anal calcd for $C_{27}H_{22}N_4OS$ [450.2]: C 71.98, H 4.92, N 12.44, S 7.12; Found: C 71.87, H 5.17, N 12.04, S 6.88.

(*Z*)-2-(3-(4-(1-(4-(*tert*-Butyl)benzyl)-1*H*-1,2,3-triazol-4-yl)benzyl)benzo[*d*]thiazol-2(3*H*)-ylidene)-1-phenylethan-1-one (6e)

The synthesis was performed according to **GPIV** using *n*-hexane/acetone 4:1 to 1:1 as an eluent to give 0.036 g (0.065 mmol, 32%) of the desired product **6e** as a yellow solid.

Mp: 230 °C.

R_f (*n*-hexane/acetone 4:1): 0.08.

^{1}H NMR (300 MHz, acetone-d$_6$/CS$_2$ 5:1): δ 1.30 (s, 9 H), 5.56 (s, 2 H), 5.59 (s, 2 H), 6.85 (s, 1 H), 7.20 – 7.29 (m, 3 H), 7.33 – 7.43 (m, 9 H), 7.75 (dd, ^3J = 7.7 Hz, ^4J = 0.9 Hz, 1 H), 7.82 – 7.85 (m, 2 H), 7.91 – 7.94 (m, 2 H), 8.20 (s, 1 H).

^{13}C NMR (75 MHz, acetone-d$_6$/CS$_2$ 5:1): δ 31.6 (CH_3), 35.0 (C_{quat}), 49.6 (CH_2), 54.0 (CH_2), 88.3 (CH), 111.4 (CH), 121.2 (CH), 123.2 (CH), 123.8 (CH), 126.5 (CH), 126.8 (CH), 127.4 (CH), 127.88 (CH), 127.94 (CH), 128.0 (C_{quat}), 128.6 (CH), 128.9 (CH), 131.4 (CH), 131.8 (C_{quat}), 133.8 (CH), 135.5 (C_{quat}), 140.5 (C_{quat}), 140.9 (C_{quat}), 147.6 (C_{quat}), 151.8 (C_{quat}), 162.3 (C_{quat}), 184.2 (C_{quat}).

MALDI-TOF (*m/z*): 557.2 ($C_{35}H_{32}N_4OS+H^+$).

IR $\tilde{\nu}$ [cm^{-1}]: 656 (w), 675 (m), 714 (s), 745 (s), 773 (m), 799 (m), 816 (m), 839 (w), 858 (w), 878 (m), 974 (m), 1024 (m), 1047 (m), 1063 (m), 1078 (m), 1094 (m), 1107 (m), 1136 (w), 1157 (m), 1179 (m), 1196 (m), 1227 (m), 1267 (w), 1296 (w), 1310 (m), 1331 (m), 1342 (m), 1412 (m), 1439 (m), 1460 (s), 1474 (s), 1495 (m), 1562 (m), 1593 (m), 1962 (w), 1977 (w), 2023 (w), 2158 (w), 2868 (w), 2941 (w), 2953 (w), 3021 (w), 3049 (w), 3063 (w), 3094 (w).

UV/Vis (C_3H_6O): λ_{max} (ε) = 383 (37400).

Anal calcd for $C_{35}H_{32}N_4OS$ [556.2]: C 75.51, H 5.79, N 10.06, S 5.76; Found: C 75.51, H 5.79, N 9.94, S 5.76.

(*Z*)-2-(3-(4-(1-Benzyl-1*H*-1,2,3-triazol-4-yl)benzyl)benzo[*d*]thiazol-2(3*H*)-ylidene)-1-phenylethan-1-one (6f)

The synthesis was performed according to **GPIV** using *n*-hexane/acetone 4:1 to 1:1 as an eluent to give 0.146 g (0.293 mmol, 65%) of the desired product **6f** as a yellow solid.

Mp: 134 °C.

R_f (*n*-hexane/acetone 4:1): 0.18.

^{1}H NMR (300 MHz, acetone-d$_6$): δ 3.58 (s, 2 H), 5.65 (s, 2 H), 6.95 (s, 1 H), 7.22 – 7.28 (m, 1 H), 7.32 – 7.45 (m, 12 H), 7.78 – 7.81 (m, 1 H), 7.86 – 7.89 (m, 2 H), 7.97 – 8.01 (m, 2 H), 8.33 (s, 1 H).

^{13}C NMR (75 MHz, acetone-d$_6$): δ 49.5 (CH_2), 54.3 (CH_2), 67.6 (C_{quat}), 88.2 (CH), 111.6 (CH), 121.6 (CH), 123.2 (CH), 123.9 (CH), 126.8 (CH), 127.6 (CH), 127.85 (CH), 127.94 (CH), 128.0 (C_{quat}), 128.8 (CH), 129.1 (CH), 129.7 (CH), 131.6 (CH), 131.8 (C_{quat}), 135.9 (C_{quat}), 137.0 (C_{quat}), 140.6 (C_{quat}), 141.0 (C_{quat}), 147.7 (C_{quat}), 162.6 (C_{quat}), 184.6 (C_{quat}).

MALDI-TOF (*m/z*): 501.2 ($C_{31}H_{24}N_4OS+H^+$).

IR $\tilde{\nu}$ [cm^{-1}]: 633 (w), 650 (m), 667 (m), 683 (m), 694 (s), 708 (s), 718 (s), 741 (s), 802 (m), 820 (m), 839 (m), 880 (s), 916 (w), 939 (w), 976 (m), 1001 (w), 1022 (m), 1045 (m), 1063 (m), 1076 (m), 1094 (w), 1115 (m), 1136 (w), 1161 (w), 1180 (m), 1194 (m), 1227 (m), 1271 (w), 1296 (w), 1308 (m), 1333 (m), 1344 (m), 1416 (m), 1439 (s), 1462 (s), 1472 (s), 1495 (m), 1568 (m), 1595 (m), 2309 (w), 2853 (w), 2922 (w), 3063 (w).

UV/Vis (C_3H_6O): λ_{max} (ε) = 383 (26700).

Anal calcd for $C_{31}H_{24}N_4OS$ [500.2]: C 74.38, H 4.83, N 11.19, S 6.40; Found: C 74.18, H 5.17, N 10.92, S 6.15.

(*Z*)-2-(3-(4-(1-(2-Fluorobenzyl)-1*H*-1,2,3-triazol-4-yl)benzyl)benzo[*d*]thiazol-2(3*H*)-ylidene)-1-phenylethan-1-one (6g)

The synthesis was performed according to **GPIV** using *n*-hexane/acetone 4:1 to 1:1 as an eluent to give 0.056 g (0.108 mmol, 54%) of the desired product **6g** as a yellow solid.

Mp: 191 °C.

R_f (*n*-hexane/acetone 4:1): 0.13.

^{1}H NMR (300 MHz, acetone-d$_6$): δ 5.67 (s, 2 H), 5.80 (s, 2 H), 6.95 (s, 1 H), 7.26 – 7.29 (m, 1 H), 7.37 – 7.47 (m, 7 H), 7.55 – 7.58 (m, 2 H), 7.72 (d, ^3J = 8.1 Hz, 2 H), 7.81 (dt, ^3J = 7.6 Hz, ^4J = 0.7 Hz, 1 H), 7.87 – 7.90 (m, 2 H), 7.97 – 8.01 (m, 2 H), 8.41 (s, 1 H).

^{13}C NMR (75 MHz, acetone-d$_6$): δ 49.5 (CH$_2$), 67.6 (CH$_2$), 88.3 (CH), 111.6 (CH), 121.9 (d, $^2J_{CF}$ = 22.0 Hz, CH), 123.3 (CH), 123.9 (CH), 126.6 (d, $^4J_{CF}$ =2.4 Hz, CH), 126.8 (CH), 127.6 (CH), 128.0 (C$_{quat}$), 128.1 (CH), 129.1 (CH), 129.4 (CH), 131.60 (d, $^3J_{CF}$ = 8.5 Hz, CH), 131.64 (C$_{quat}$), 136.0 (C$_{quat}$), 140.6 (C$_{quat}$), 141.0 (C$_{quat}$), 141.6 (C$_{quat}$), 147.9 (C$_{quat}$), 162.6 (d, $^1J_{CF}$ = 249.2 Hz, C$_{quat}$), 184.6 (C$_{quat}$).

MALDI-TOF (*m/z*): 519.2 ($C_{31}H_{23}FN_4OS+H^+$).

IR $\tilde{\nu}$ [cm^{-1}]: 613 (w), 652 (w), 689 (m), 700 (m), 721 (m), 743 (s), 775 (m), 802 (m), 820 (m), 843 (m), 874 (m), 880 (s), 928 (w), 978 (m), 999 (w), 1016 (m), 1047 (m), 1065 (s), 1080 (w), 1094 (m), 1117 (s), 1157 (m), 1196 (m), 1231 (m), 1263 (w), 1296 (w), 1325 (s), 1344 (m), 1416 (m), 1439 (s), 1462 (s), 1495 (m), 1564 (m), 1595 (m), 2390 (w), 2849 (w), 3057 (w), 3103 (w).

UV/Vis (C_3H_6O): λ_{max} (ε) = 382 (32300).

Anal calcd for $C_{31}H_{23}FN_4OS$ [518.2]: C 71.80, H 4.47, N 10.80, S 6.18; Found: C 71.62, H 4.45, N 10.72, S 6.03.

(*Z*)-2-(3-(4-(1-(4-Iodobenzyl)-1*H*-1,2,3-triazol-4-yl)benzyl)benzo[*d*]thiazol-2(3*H*)-ylidene)-1-phenylethan-1-one (6h)

The synthesis was performed according to **GPIV** using *n*-hexane/acetone 4:1 to 1:1 as an eluent to give 0.111 g (0.177 mmol, 89%) of the desired product **6h** as a yellow solid.

Mp: 206 °C.

R_f (*n*-hexane/acetone 4:1): 0.07.

^{1}H NMR (300 MHz, acetone-d$_6$/CS$_2$ 5:1): δ 5.60 (s, 4 H), 6.86 (s, 1 H), 7.15 – 7.19 (m, 2 H), 7.21 – 7.26 (m, 1 H), 7.34 – 7.46 (m, 8 H), 7.70 – 7.79 (m, 2 H), 7.82 – 7.85 (m, 2 H), 7.92 – 7.96 (m, 2 H), 8.26 (s, 1 H).

^{13}C NMR (75 MHz, acetone-d$_6$/CS$_2$ 5:1): δ 49.6 (CH$_2$), 53.7 (CH$_2$), 88.3 (CH), 111.4 (CH), 121.4 (CH), 123.2 (CH), 123.8 (CH), 126.8 (CH), 127.5 (CH), 127.92 (CH), 127.94 (CH), 128.0 (C_{quat}), 129.0 (CH), 130.9 (CH), 131.2 (C_{quat}), 131.5 (CH), 131.7 (C_{quat}), 135.6 (C_{quat}), 136.7 (C_{quat}), 138.7 (CH), 140.5 (C_{quat}), 140.9 (C_{quat}), 147.7 (C_{quat}), 162.4 (C_{quat}), 184.3 (C_{quat}).

MALDI-TOF (*m/z*): 627.1 ($C_{31}H_{23}IN_4OS+H^+$).

IR $\tilde{\nu}$ [cm^{-1}]: 650 (w), 669 (w), 687 (m), 719 (s), 741 (s), 770 (m), 800 (m), 818 (m), 845 (w), 880 (s), 978 (m), 1020 (m), 1049 (m), 1063 (m), 1080 (w), 1092 (w), 1113 (w), 1142 (w), 1157 (w), 1180 (m), 1194 (m), 1227 (m), 1265 (w), 1292 (w), 1312 (w), 1329 (m), 1346 (m), 1404 (m), 1437 (s), 1458 (s), 1564 (m), 1593 (m), 2097 (w), 2851 (w), 2901 (w), 2972 (w).

UV/Vis (C_3H_6O): λ_{max} (ε) = 382 (31200).

Anal calcd for $C_{31}H_{23}IN_4OS$ [626.1]: C 59.43, H 3.70, N 8.94, S 5.12; Found: C 59.09, H 3.75, N 9.06, S 4.87.

(*Z*)-1-Phenyl-2-(3-(4-(1-(4-(trifluoromethyl)benzyl)-1*H*-1,2,3-triazol-4-yl)benzyl)benzo[*d*]thiazol-2(3*H*)-ylidene)ethan-1-one (6i)

The synthesis was performed according to **GPIV** using *n*-hexane/acetone 4:1 to 1:1 as an eluent to give 0.085 g (0.150 mmol, 75%) of the desired product **6i** as a yellow solid.

Mp: 154 °C.

R_f (*n*-hexane/acetone 4:1): 0.07.

^{1}H NMR (300 MHz, acetone-d$_6$/CS$_2$ 5:1): δ 5.62 (s, 2 H), 5.69 (s, 2 H), 6.90 (s, 1 H), 7.15 – 7.26 (m, 3 H), 7.32 – 7.46 (m, 9 H), 7.76 (dt, ^3J = 7.7 Hz, ^4J = 0.8 Hz, 1 H), 7.84 – 7.88 (m, 2 H), 7.94 – 7.98 (m, 2 H), 8.29 (s, 1 H).

^{13}C NMR (75 MHz, acetone-d$_6$/CS$_2$ 5:1): δ 48.0 (CH_2), 49.6 (CH_2), 67.6 (C_{quat}), 88.3 (CH), 111.5 (CH), 116.2 (CH), 116.5 (CH), 121.6 (CH), 123.2 (CH), 123.7 (CH), 123.8 (CH), 123.9 (CH), 125.6 (C_{quat}), 126.8 (CH), 127.5 (CH), 128.0 (CH), 129.0 (CH), 131.4 (C_{quat}), 131.5 (q, $^3J_{CF}$ = 3.9 Hz, CF_3), 131.6 (CH), 131.7 (C_{quat}), 135.7 (C_{quat}), 140.5 (C_{quat}), 140.9 (C_{quat}), 147.6 (C_{quat}), 159.7 (C_{quat}), 162.5 (C_{quat}), 163.0 (C_{quat}),184.4 (C_{quat}).

MALDI-TOF (*m/z*): 569.2 ($C_{32}H_{23}F_3N_4OS$+H^+).

IR $\tilde{\nu}$ [cm^{-1}]: 606 (w), 650 (w), 669 (w), 679 (m), 698 (m), 719 (s), 791 (w), 802 (w), 820 (m), 845 (m), 880 (s), 932 (w), 976 (m), 1001 (w), 1024 (m), 1045 (m), 1061 (m), 1076 (m), 1094 (m), 1117 (m), 1156 (m), 1180 (m), 1192 (m), 1229 (m), 1263 (m), 1296 (m), 1319 (m), 1333 (m), 1344 (m), 1381 (w), 1416 (m), 1439 (s), 1464 (s), 1474 (s), 1495 (m), 1566 (m), 1595 (m), 2100 (w), 2848 (w), 2922 (w), 2961 (w), 3103 (w).

UV/Vis (C_3H_6O): λ_{max} (ε) = 382 (39400).

Anal calcd for $C_{32}H_{23}F_3N_4OS$ [568.2]: C 67.59, H 4.08, N 9.85, S 5.64; Found: C 67.49, H 4.12, N 10.03, S 5.57.

(*Z*)-4-((4-(4-((2-(2-Oxo-2-phenylethylidene)benzo[*d*]thiazol-3(2*H*)-yl)methyl)phenyl)-1*H*-1,2,3-triazol-1-yl)methyl)benzonitrile (6j)

The synthesis was performed according to **GPIV** using *n*-hexane/acetone 3:1 to 1:1 as an eluent to give 0.095 g (0.181 mmol, 90%) of the desired product **6j** as a yellow solid.

Mp: 105 °C.

R_f (*n*-hexane/acetone 4:1): 0.08.

^{1}H NMR (300 MHz, acetone-d$_6$): δ 5.66 (s, 2 H), 5.80 (s, 2 H), 6.95 (s, 1 H), 7.23 – 7.32 (m, 2 H), 7.36 – 7.48 (m, 6 H), 7.51 – 7.54 (m, 2 H), 7.76 – 7.82 (m, 3 H), 7.86 – 7.90 (m, 2 H), 7.97 – 8.00 (m, 2 H), 8.41 (s, 1 H).

^{13}C NMR (75 MHz, acetone-d$_6$): δ 49.5 (CH_2), 53.7 (CH_2), 67.6 (C_{quat}), 88.3 (CH), 111.6 (CH), 112.9 (CH), 119.0 (C_{quat}), 122.0 (CH), 123.3 (CH), 123.9 (CH), 126.4 (CH), 126.8 (CH), 127.6 (CH), 127.86 (C_{quat}), 127.94 (CH), 128.1 (CH), 129.1 (CH), 129.6 (CH), 131.6 (CH), 133.5 (CH), 136.0 (C_{quat}), 140.6 (C_{quat}), 141.0 (C_{quat}), 142.3 (C_{quat}), 147.9 (C_{quat}), 151.9 (C_{quat}), 162.6 (C_{quat}), 184.6 (C_{quat}).

MALDI-TOF (*m/z*): 526.2 ($C_{32}H_{23}N_5OS+H^+$).

IR $\tilde{\nu}$ [cm^{-1}]: 604 (w), 654 (m), 681 (m), 700 (m), 719 (s), 745 (s), 770 (m), 800 (m), 818 (m), 849 (m), 880 (m), 937 (w), 957 (w), 976 (m), 1001 (w), 1020 (m), 1045 (m), 1063 (m), 1082 (m), 1094 (w), 1117 (m), 1140 (w), 1159 (m), 1179 (m), 1192 (m), 1231 (m), 1254 (m), 1265 (m), 1290 (m), 1304 (m), 1331 (m), 1346 (m), 1412 (m), 1437 (s), 1466 (s), 1495 (m), 1564 (m), 1593 (m), 2097 (w), 2226 (w), 2887 (w), 2905 (w), 2914 (w), 2930 (w), 2955 (w).

UV/Vis (C_3H_6O): λ_{max} (ε) = 382 (39400).

Anal calcd for $C_{32}H_{23}N_5OS$ [525.2]: C 73.12, H 4.41, N 13.32, S 6.10; Found: C 72.84, H 4.18, N 13.70, S 5.90.

(*Z*)-2-(3-(4-(1-(Naphthalene-2-ylmethyl)-1*H*-1,2,3-triazol-4-yl)benzyl)benzo[*d*]thiazol-2(3*H*)-ylidene)-1-phenylethan-1-one (6k)

The synthesis was performed according to **GPIV** using *n*-hexane/acetone 3:1 to 1:1 as an eluent to give 0.114 g (0.189 mmol, 95%) of the desired product **6k** as a yellow solid.

Mp: 180 °C.

R_f (*n*-hexane/acetone 4:1): 0.08.

^{1}H NMR (600 MHz, acetone-d$_6$/CS$_2$ 5:1): δ 5.57 (s, 2 H), 5.78 (s, 2 H), 6.84 (s, 1 H), 7.21 – 7.23 (m, 1 H), 7.34 – 7.46 (m, 8 H), 7.48 – 7.53 (m, 4 H), 7.73 (dt, ^3J = 7.7 Hz, ^4J = 0.9 Hz, 1 H), 7.82 – 7.94 (m, 6 H), 8.26 (s, 1 H).

^{13}C NMR (150 MHz, acetone-d$_6$/CS$_2$ 5:1): δ 49.6 (CH$_2$), 55.4 (CH$_2$), 67.6 (C$_{quat}$), 88.3 (CH), 111.4 (CH), 121.4 (CH), 123.1 (CH), 123.8 (CH), 126.4 (CH), 126.8 (CH), 126.9 (CH), 127.1 (CH), 127.2 (CH), 127.3 (CH). 127.4 (CH), 127.85 (C$_{quat}$), 127.93 (CH), 128.0 (CH), 128.5 (CH), 128.7 (CH), 128.9 (CH), 129.4 (CH), 129.5 (CH), 131.4 (CH), 131.7 (C$_{quat}$), 133.9 (C$_{quat}$), 134.1 (C$_{quat}$), 134.2 (C$_{quat}$), 135.4 (C$_{quat}$), 140.5 (C$_{quat}$), 140.8 (C$_{quat}$), 147.6 (C$_{quat}$), 162.4 (C$_{quat}$), 184.2 (C$_{quat}$).

MALDI-TOF (*m/z*): 551.2 ($C_{35}H_{26}N_4OS+H^+$).

IR $\tilde{\nu}$ [cm^{-1}]: 606 (m), 631 (w), 652 (w), 667 (m), 694 (m), 718 (s), 741 (s), 777 (m), 799 (m), 824 (s), 854 (m), 878 (m), 974 (m), 1016 (m), 1049 (m), 1061 (m), 1092 (w), 1117 (w), 1144 (w), 1179 (m), 1190 (m), 1227 (m), 1260 (m), 1273 (m), 1290 (m), 1317 (m), 1327 (m), 1364 (w), 1422 (m), 1435 (s), 1454 (s), 1470 (s), 1495 (m), 1564 (m), 1595 (m), 1711 (w), 1800 (w), 1954 (w), 2099 (w), 2212 (w), 2520 (w), 2590 (w), 2625 (w), 2853 (w), 2884 (w), 2907 (w), 2938 (w), 2972 (w), 3021 (w), 3055 (w), 3130 (w), 3339 (w).

UV/Vis (C_3H_6O): λ_{max} (ε) = 382 (25300).

Anal calcd for $C_{35}H_{26}N_4OS$ [550.2]: C 76.34, H 4.76, N 10.17, S 5.82; Found: C 76.24, H 4.84, N 10.09, S 5.58.

6 NMR Spectra

6.1 NMR spectra of alkynylated aroyl-*S*,*N*-ketene acetals 3

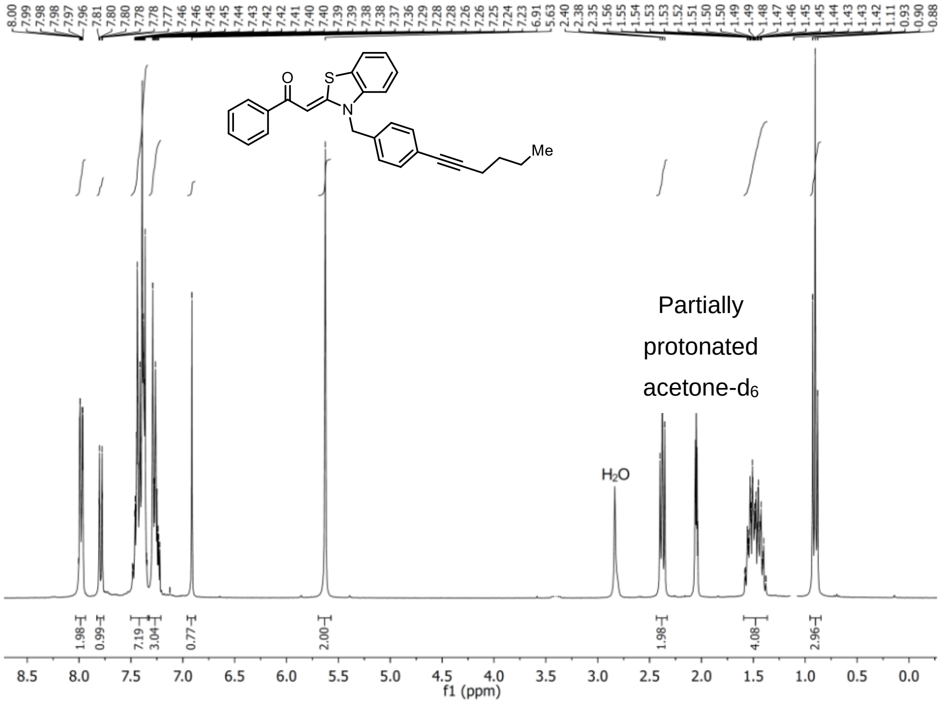

Figure S9. ^{1}H NMR spectrum (*Z*)-2-(3-(4-(hex-1-yn-1-yl)benzyl)benzo[*d*]thiazol-2(3*H*)-ylidene)-1-phenylethan-1-one (**3a**) (acetone-d_6, 300 MHz, 298 K).

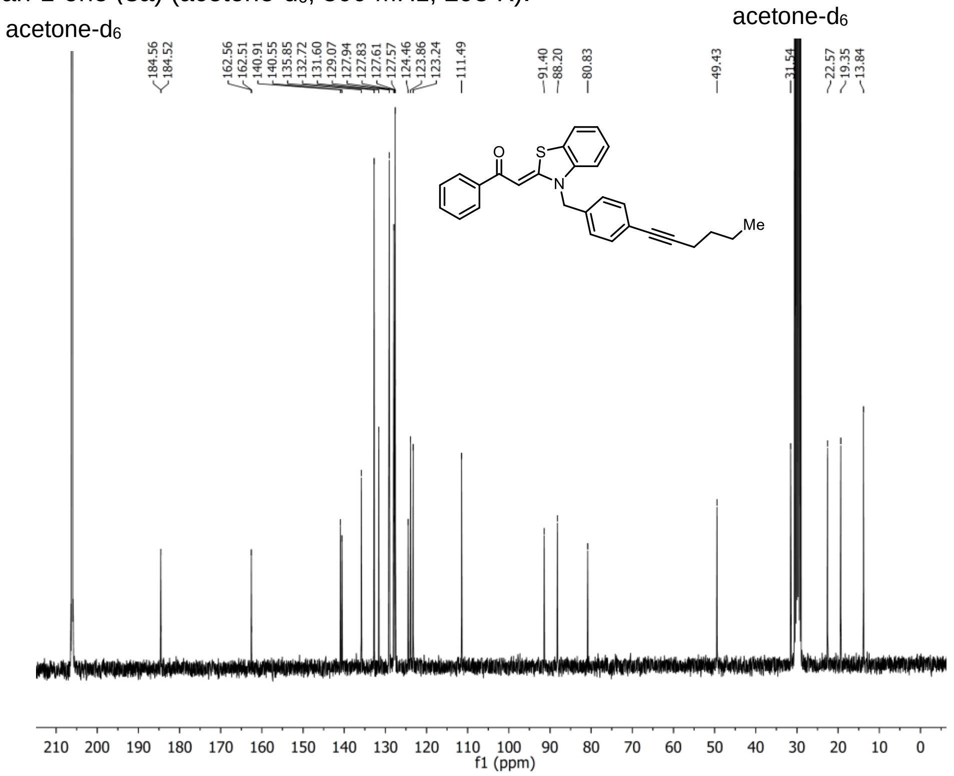

Figure S10. ^{13}C NMR spectrum (*Z*)-2-(3-(4-(hex-1-yn-1-yl)benzyl)benzo[*d*]thiazol-2(3*H*)-ylidene)-1-phenylethan-1-one (**3a**) (acetone-d_6, 75 MHz, 298 K).

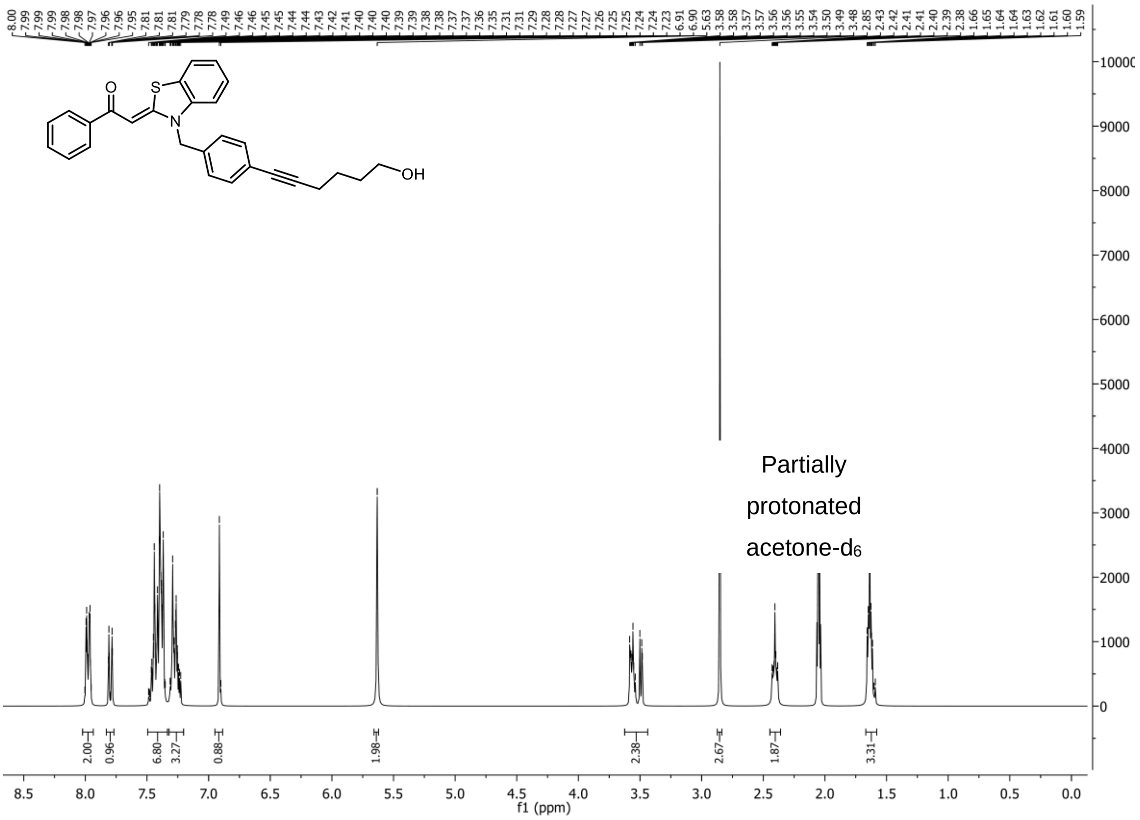

Figure S11. ^{1}H NMR spectrum (*Z*)-2-(3-(4-(6-hydroxyhex-1-yn-1-yl)benzyl)benzo[*d*]thiazol-2(3*H*)-ylidene)-1-phenylethan-1-one (**3b**) (acetone-d$_6$, 300 MHz, 298 K).

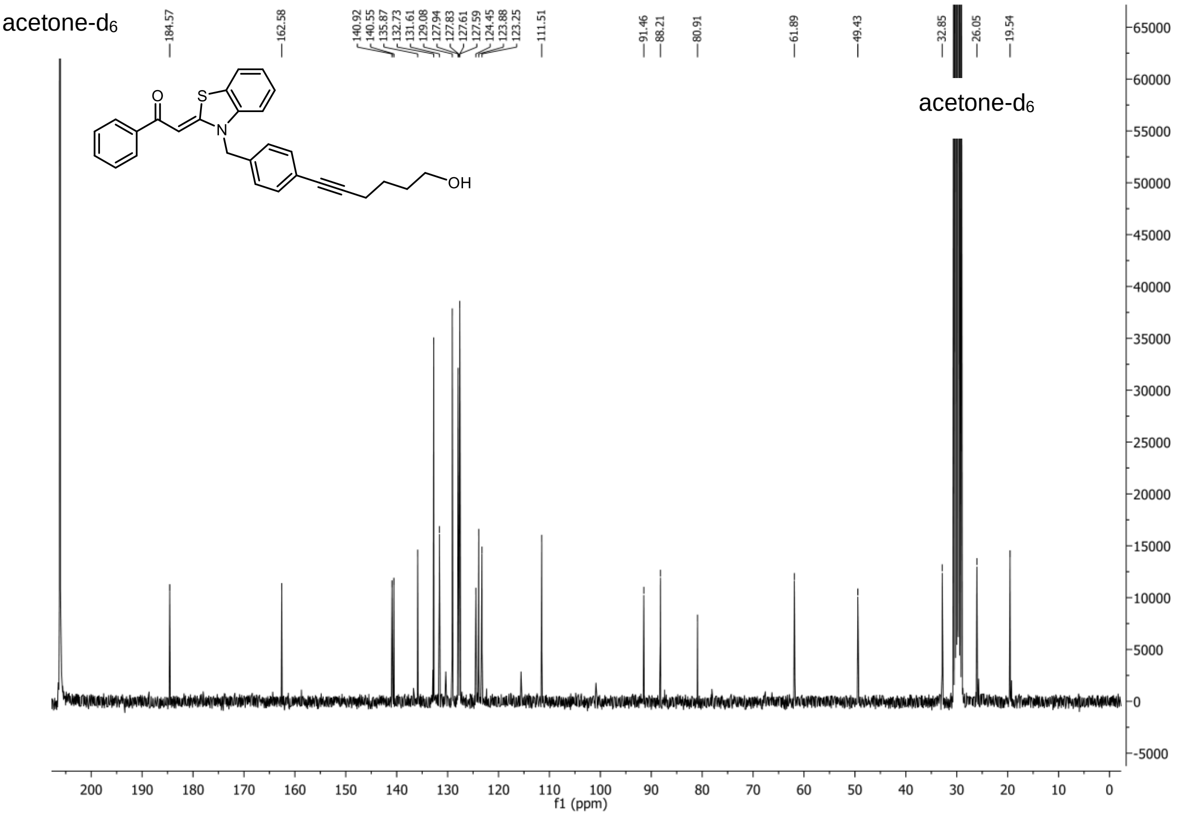

Figure S12. ^{13}C NMR spectrum (*Z*)-2-(3-(4-(6-hydroxyhex-1-yn-1-yl)benzyl)benzo[*d*]thiazol-2(3*H*)-ylidene)-1-phenylethan-1-one (**3b**) (acetone-d$_6$, 75 MHz, 298 K).

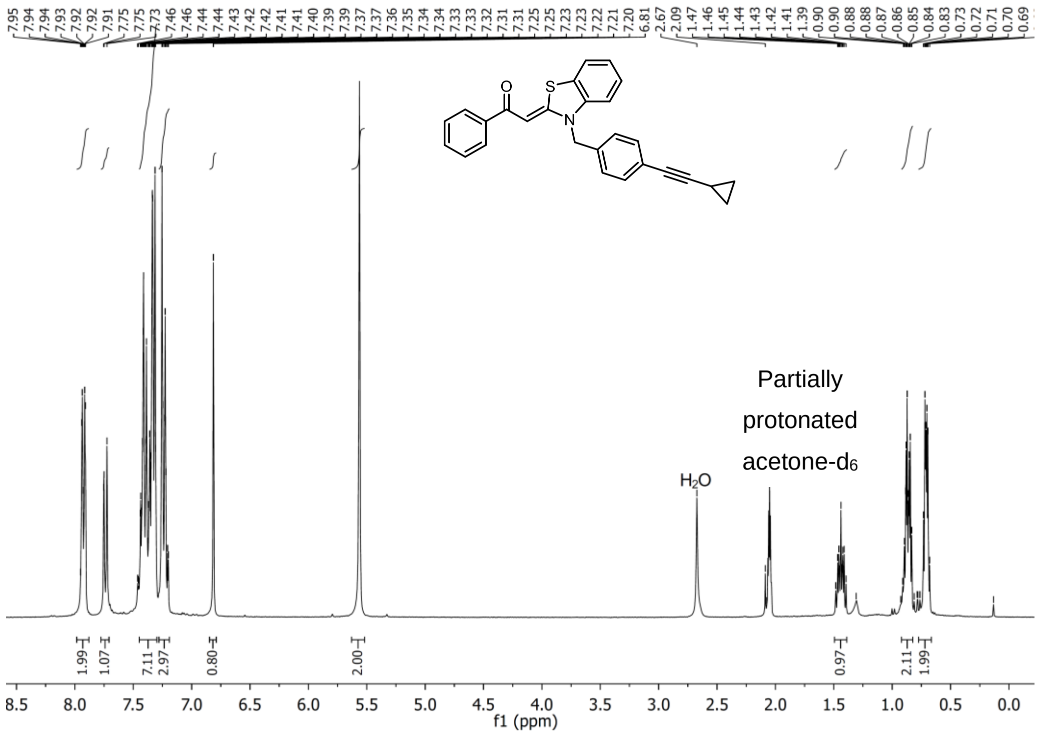

Figure S13. ^{1}H NMR spectrum (*Z*)-2-(3-(4-(cyclopropylethynyl)benzyl)benzo[*d*]thiazol-2(3*H*)-ylidene)-1-phenylethan-1-one (**3c**) (acetone-d_6/CS_2 5:1, 300 MHz, 298 K).

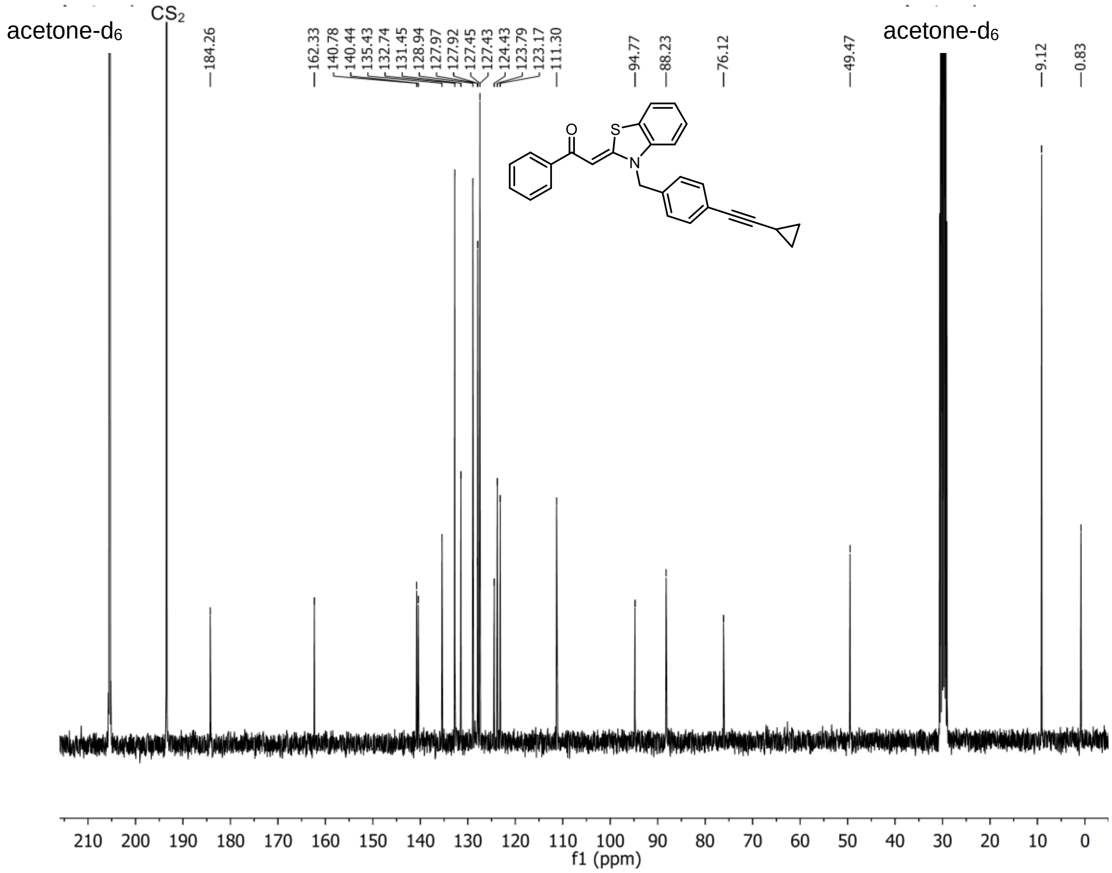

Figure S14. ^{13}C NMR spectrum (*Z*)-2-(3-(4-(cyclopropylethynyl)benzyl)benzo[*d*]thiazol-2(3*H*)-ylidene)-1-phenylethan-1-one (**3c**) (acetone-d_6/CS_2 5:1, 75 MHz, 298 K).

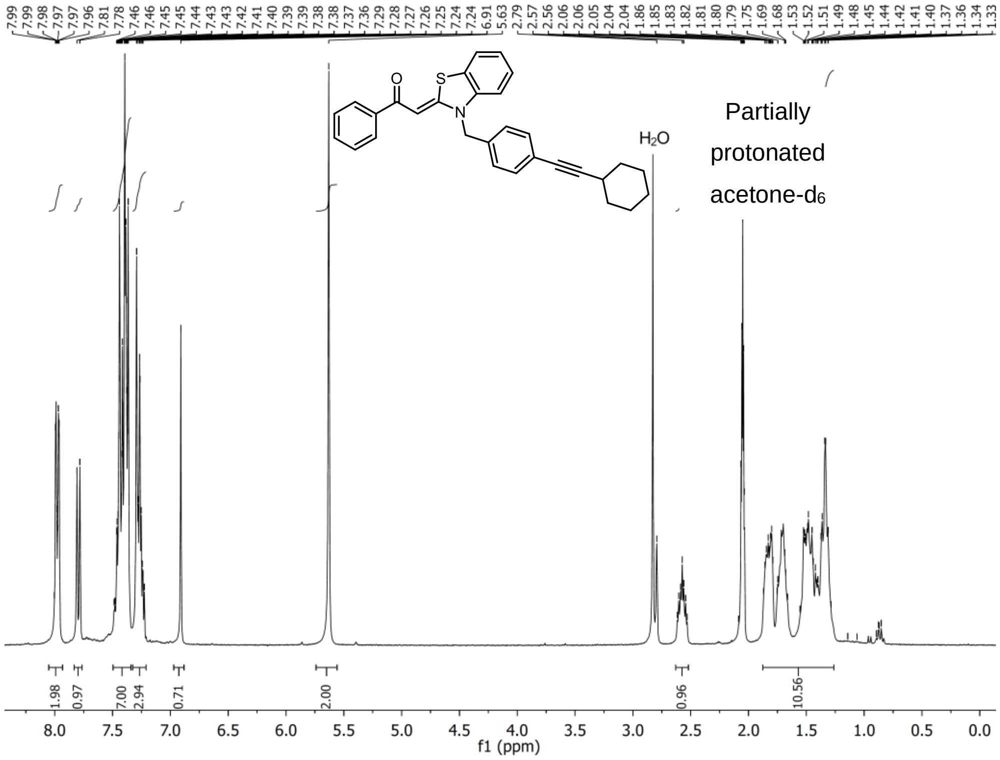

Figure S15. ^{1}H NMR spectrum (*Z*)-2-(3-(4-(cyclohexylethynyl)benzyl)benzo[*d*]thiazol-2(3*H*)-ylidene)-1-phenylethan-1-one (**3d**) (acetone-d$_6$, 300 MHz, 298 K).

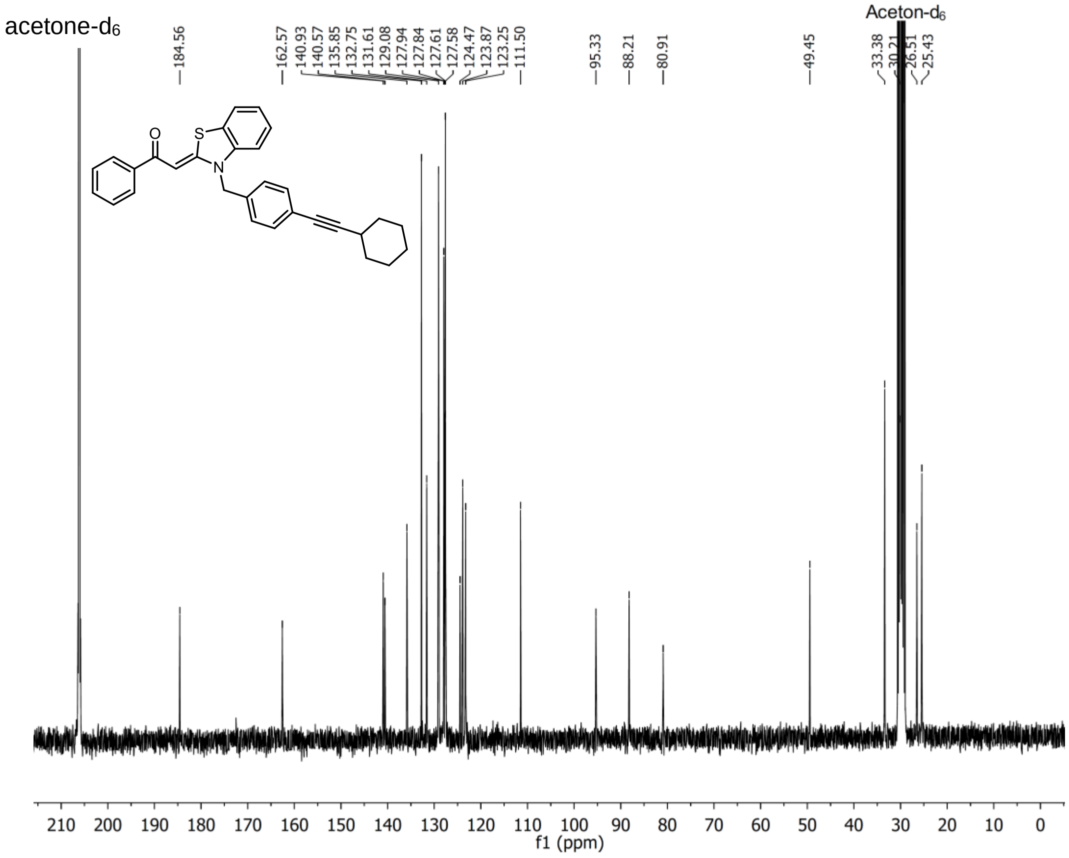

Figure S16. ^{13}C NMR spectrum (*Z*)-2-(3-(4-(cyclohexylethynyl)benzyl)benzo[*d*]thiazol-2(3*H*)-ylidene)-1-phenylethan-1-one (**3d**) (acetone-d$_6$, 75 MHz, 298 K).

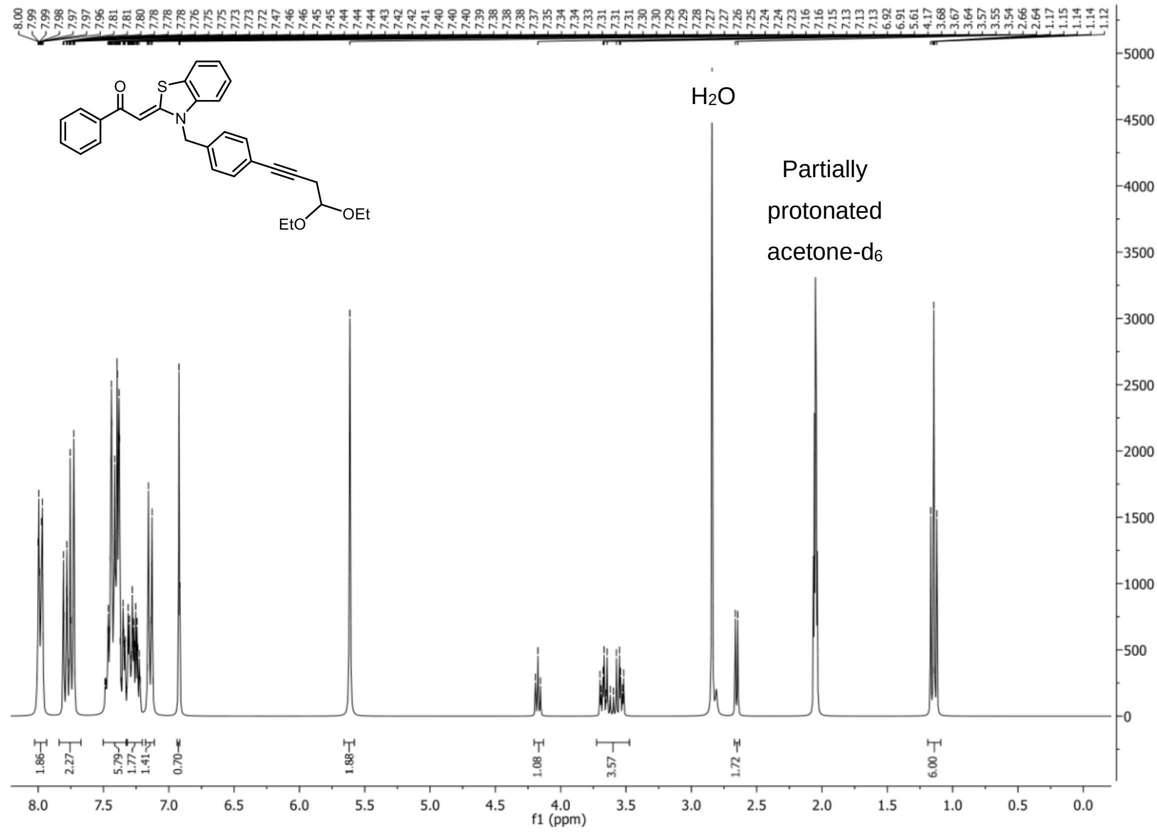

Figure S17. ^{1}H NMR spectrum (*Z*)-2-(3-(4-(4,4-diethoxybut-1-yn-1-yl)benzyl)benzo[*d*]thiazol-2(3*H*)-ylidene)-1-phenylethan-1-one (**3e**) (acetone-d$_6$, 300 MHz, 298 K).

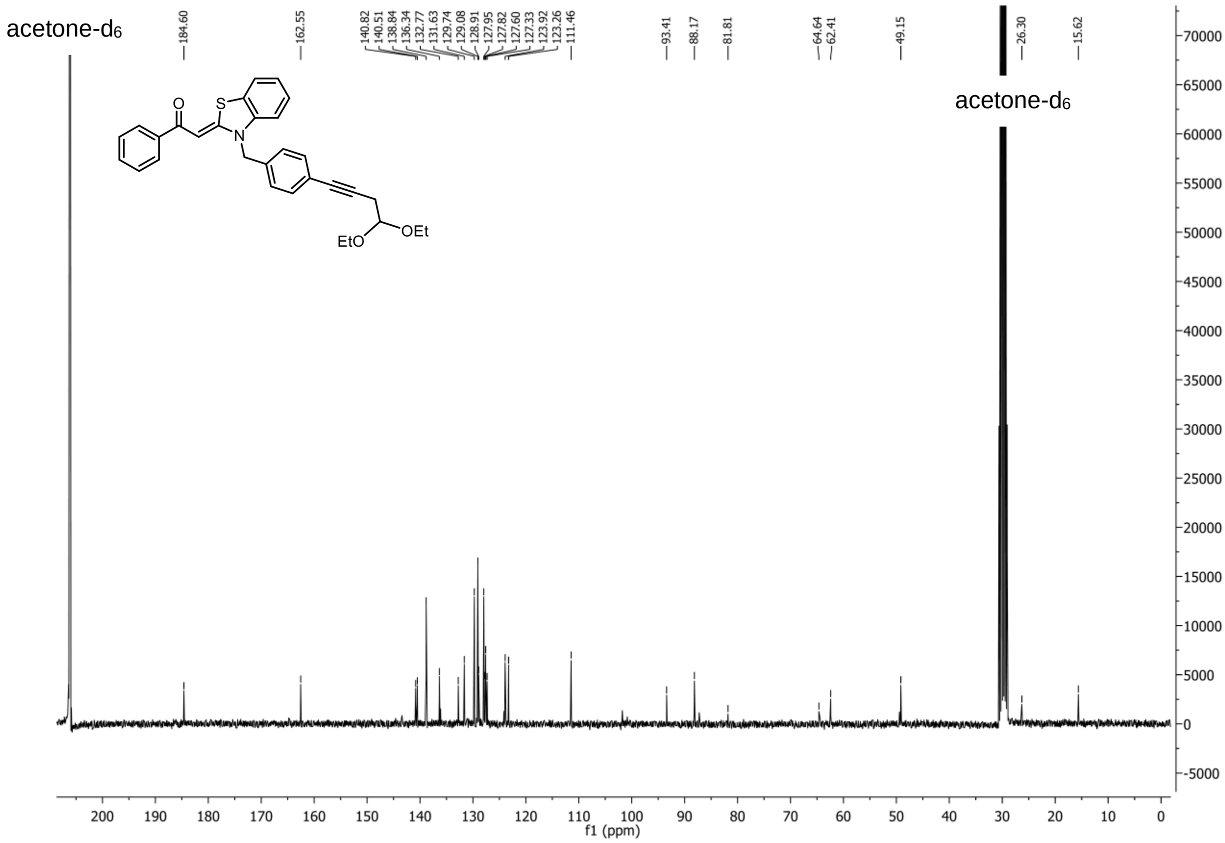

Figure S18. ^{13}C NMR spectrum (*Z*)-2-(3-(4-(4,4-diethoxybut-1-yn-1-yl)benzyl)benzo[*d*]thiazol-2(3*H*)-ylidene)-1-phenylethan-1-one (**3e**) (acetone-d$_6$, 75 MHz, 298 K).

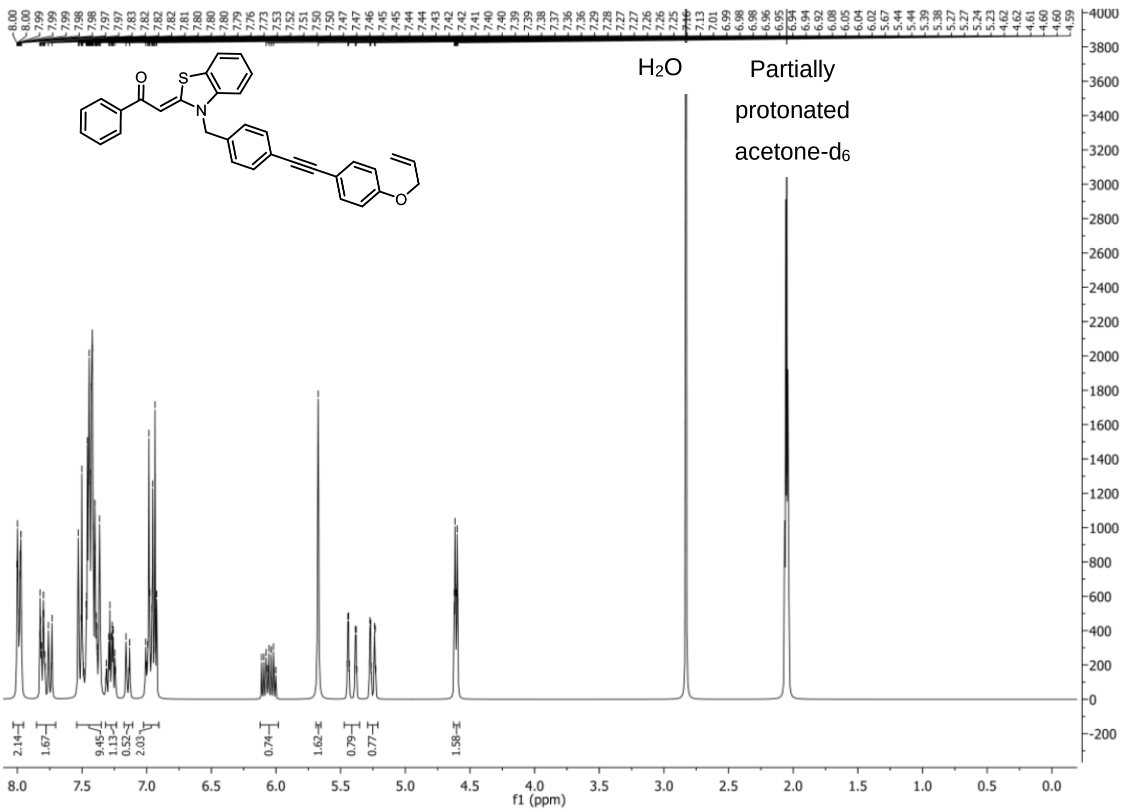

Figure S19. ^{1}H NMR spectrum (*Z*)-2-(3-(4-((4-(allyloxy)phenyl)ethynyl)benzyl)benzo[*d*]thiazol-2(3*H*)-ylidene)-1-phenylethan-1-one (**3f**) (acetone-d_6, 300 MHz, 298 K).

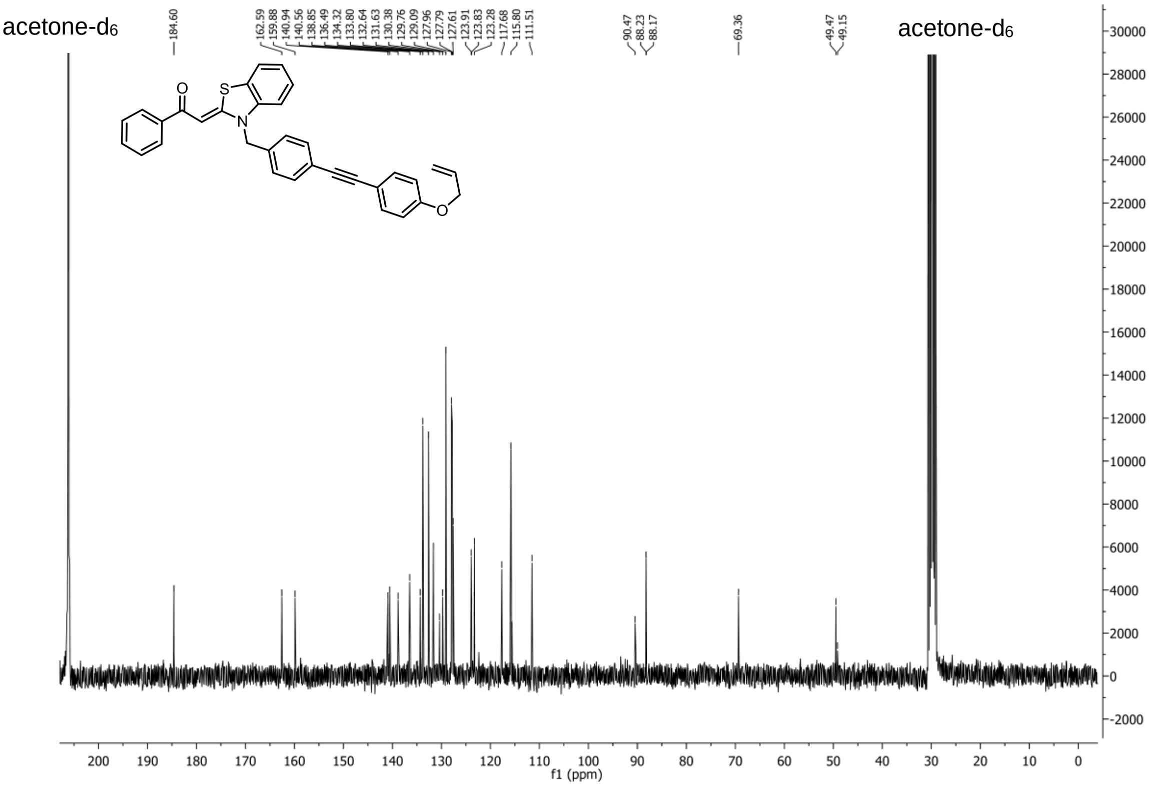

Figure S20. ^{13}C NMR spectrum (*Z*)-2-(3-(4-((4-(allyloxy)phenyl)ethynyl)benzyl)benzo[*d*]thiazol-2(3*H*)-ylidene)-1-phenylethan-1-one (**3f**) (acetone-d_6, 75 MHz, 298 K).

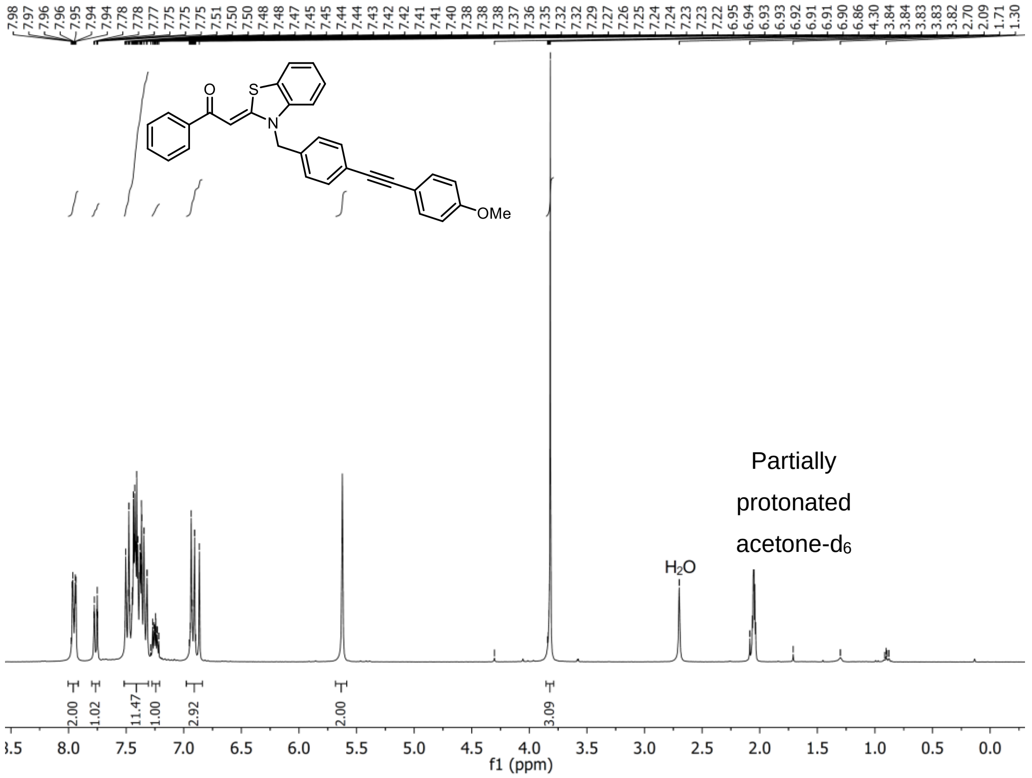

Figure S21. ^{1}H NMR spectrum (*Z*)-2-(3-(4-((4-methoxyphenyl)ethinyl)benzyl)benzo[*d*]thiazol-2(3*H*)-ylidene)-1-phenylethan-1-one (**3g**) (acetone-d_6/CS_2 5:1, 300 MHz, 298 K).

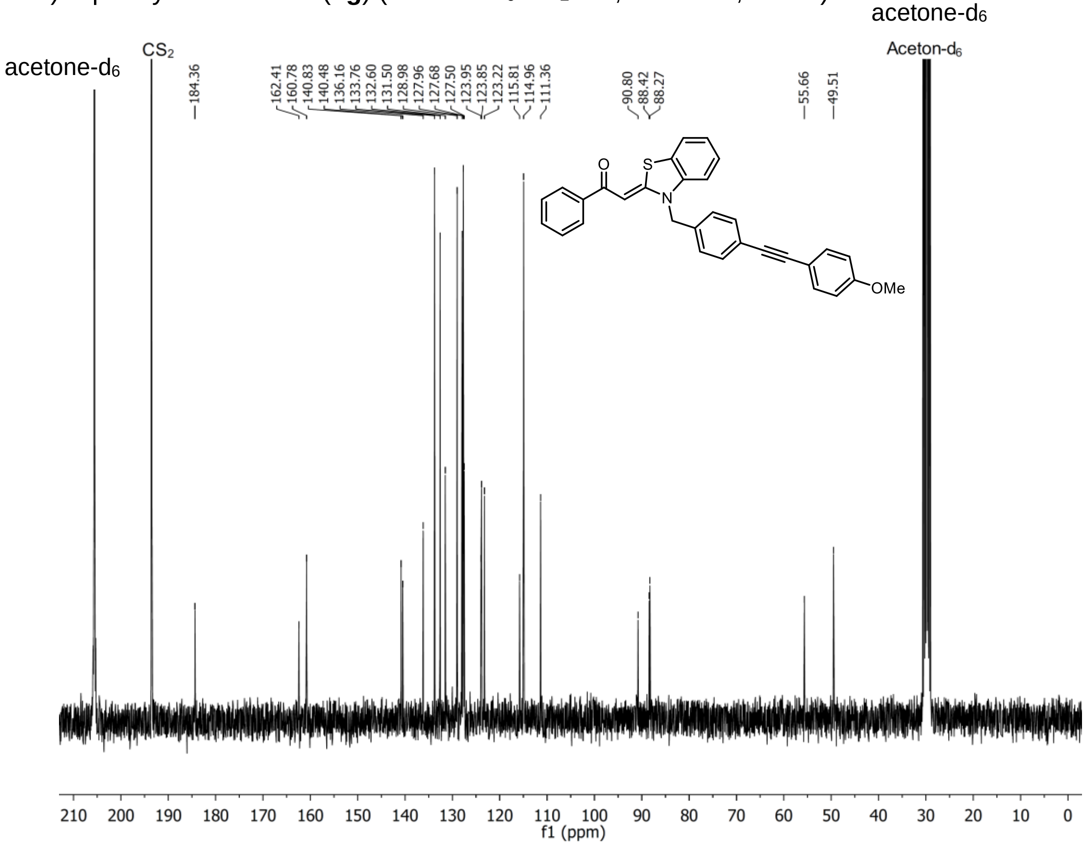

Figure S22. ^{13}C NMR spectrum (*Z*)-2-(3-(4-((4-methoxyphenyl)ethynyl)benzyl)benzo[*d*]thiazol-2(3*H*)-ylidene)-1-phenylethan-1-one (**3g**) (acetone-d_6/CS_2 5:1, 75 MHz, 298 K).

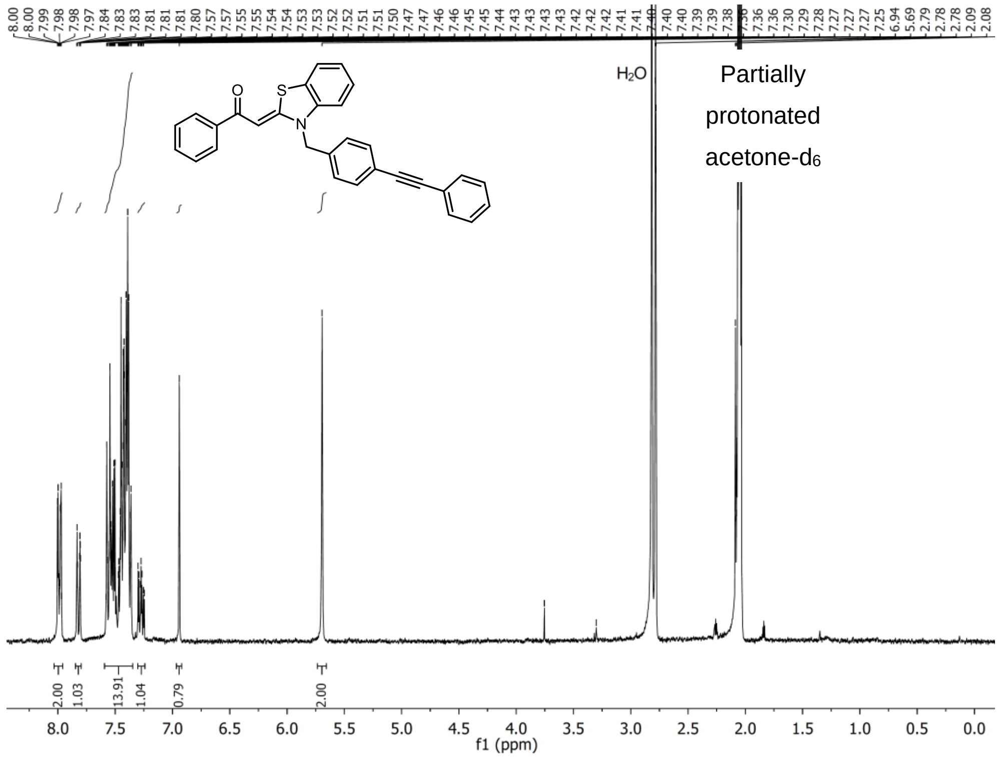

Figure S23. ^{1}H NMR spectrum (*Z*)-1-phenyl-2-(3-(4-(phenylethinyl)benzyl)benzo[*d*]thiazol-2(3*H*)-ylidene)ethan-1-one (**3h**) (acetone-d$_6$/CS_2 5:1, 300 MHz, 298 K).

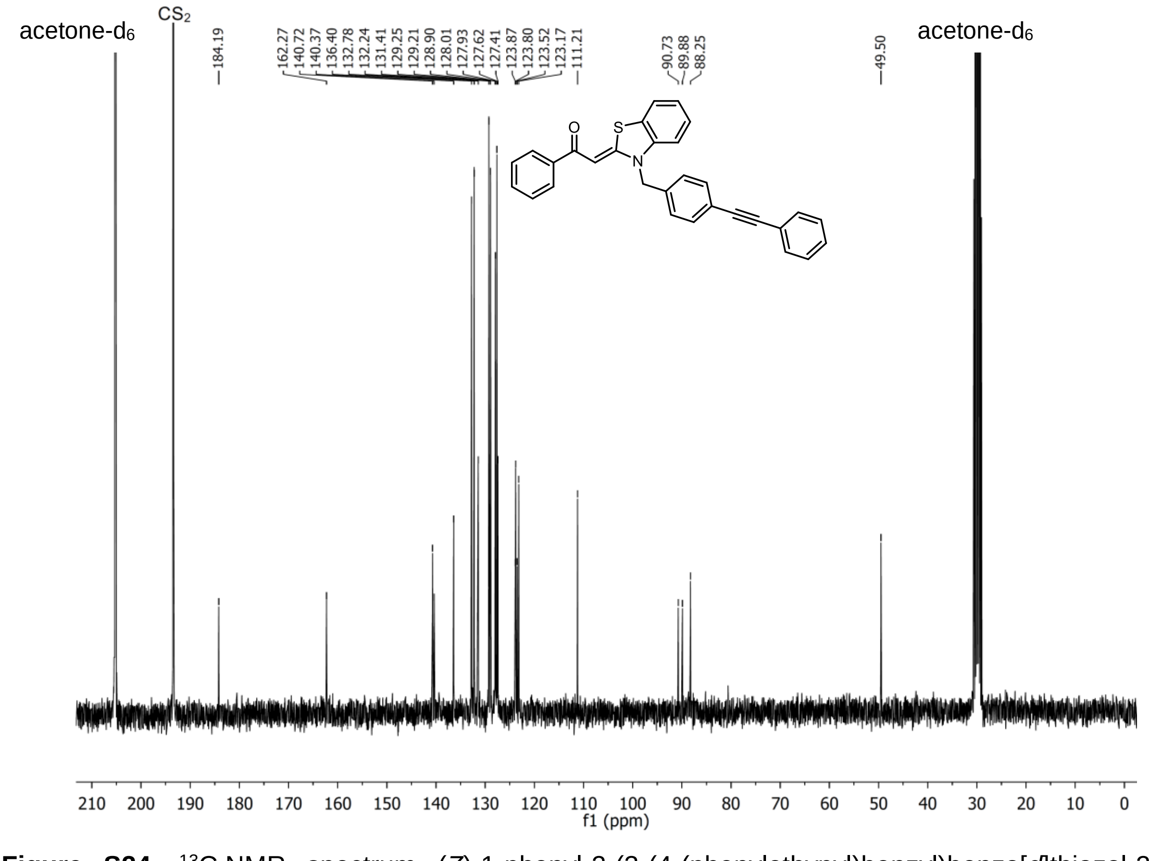

Figure S24. ^{13}C NMR spectrum (*Z*)-1-phenyl-2-(3-(4-(phenylethynyl)benzyl)benzo[*d*]thiazol-2(3*H*)-ylidene)ethan-1-one (**3h**) (acetone-d$_6$/CS_2 5:1, 75 MHz, 298 K).

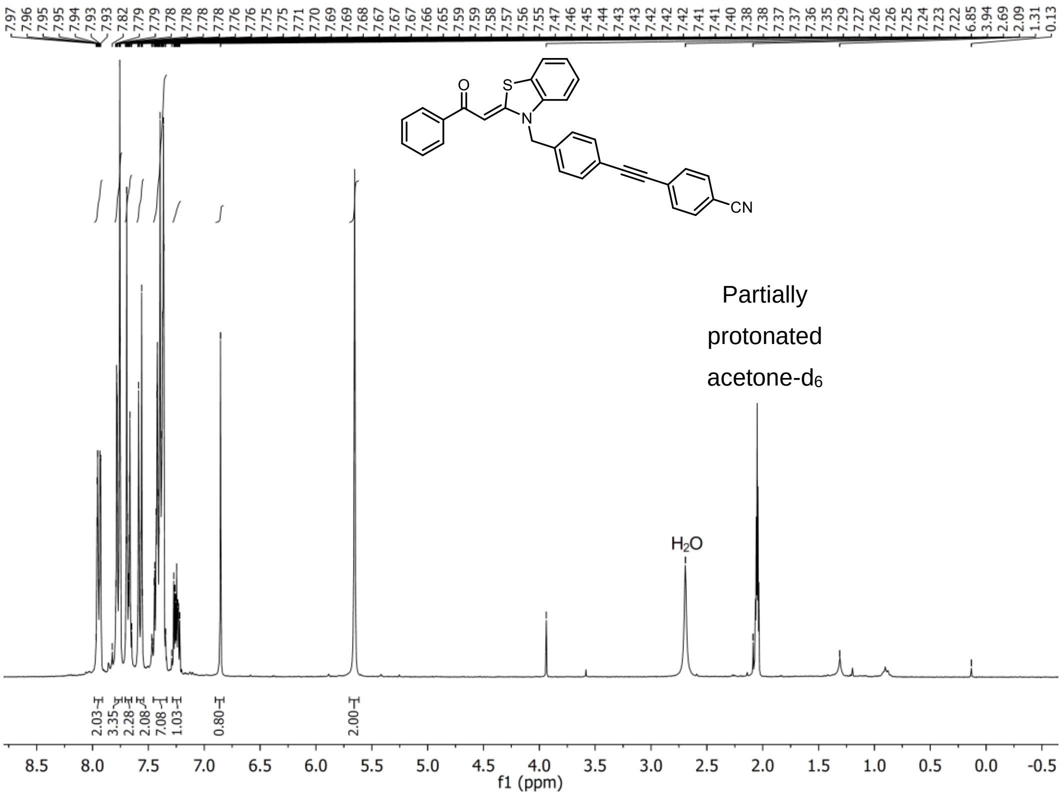

Figure S25. ^{1}H NMR spectrum (*Z*)-4-((4-((2-(2-oxo-2-phenylethylidene)benzo[*d*]thiazol-3(2*H*)-yl)methyl)phenyl)ethynyl)benzonitrile (**3i**) (acetone-d$_6$/CS_2 5:1, 300 MHz, 298 K).

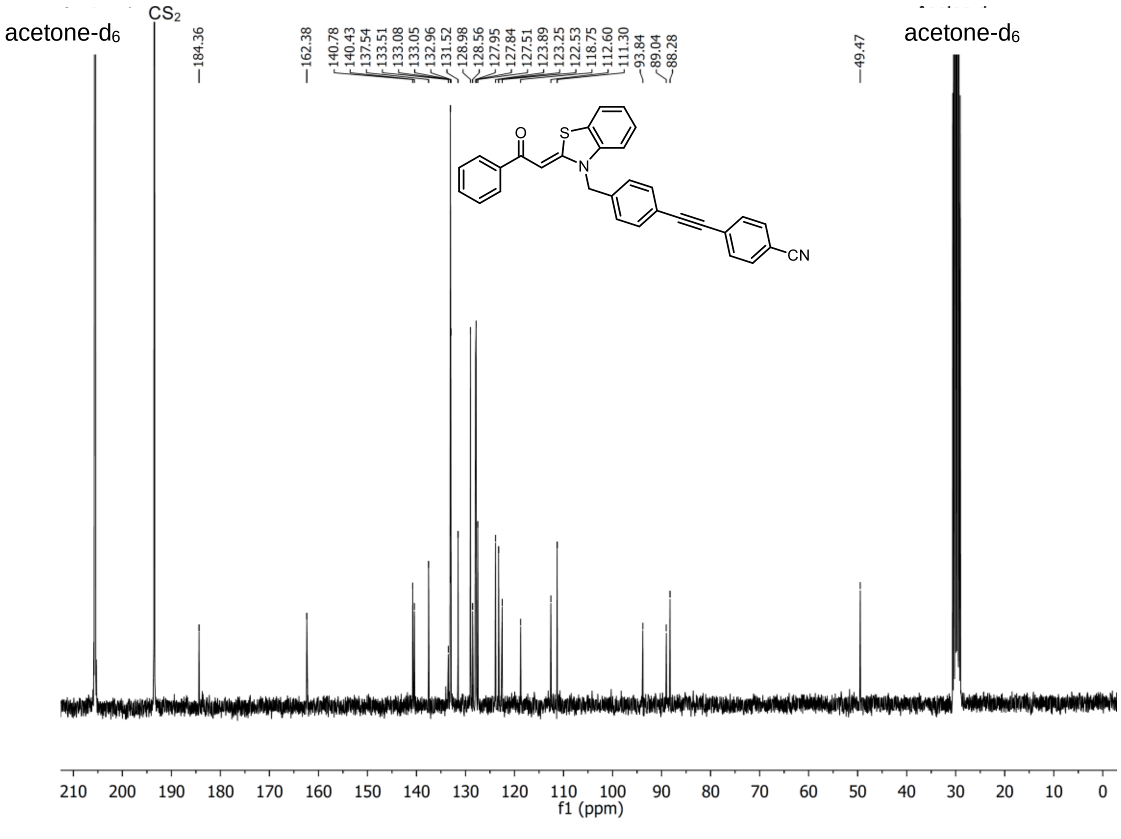

Figure S26. ^{13}C NMR spectrum (*Z*)-4-((4-((2-(2-oxo-2-phenylethylidene)benzo[*d*]thiazol-3(2*H*)-yl)methyl)phenyl)ethynyl)benzonitrile (**3i**) (acetone-d$_6$/CS_2 5:1, 75 MHz, 298 K).

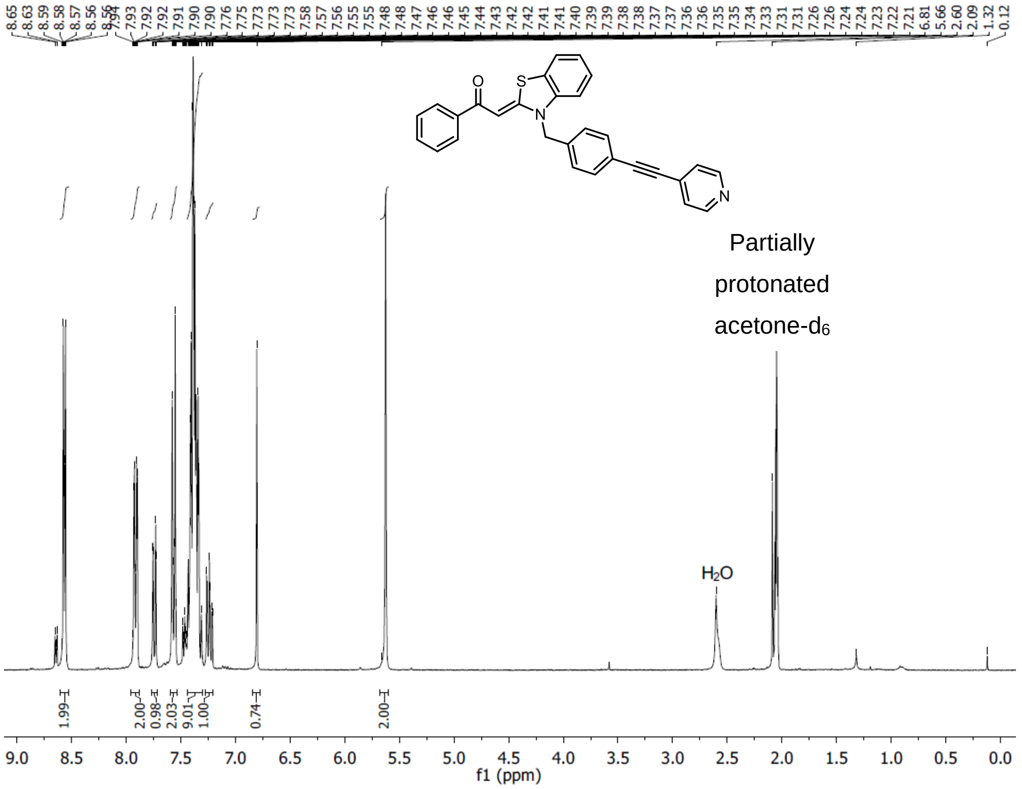

Figure S27. ^{1}H NMR spectrum (*Z*)-1-phenyl-2-(3-(4-(pyridine-4-ylethynyl)benzyl)benzo[*d*]thiazol-2(3*H*)-ylidene)ethan-1-one (**3j**) (acetone-d_6/CS_2 5:1, 300 MHz, 298 K).

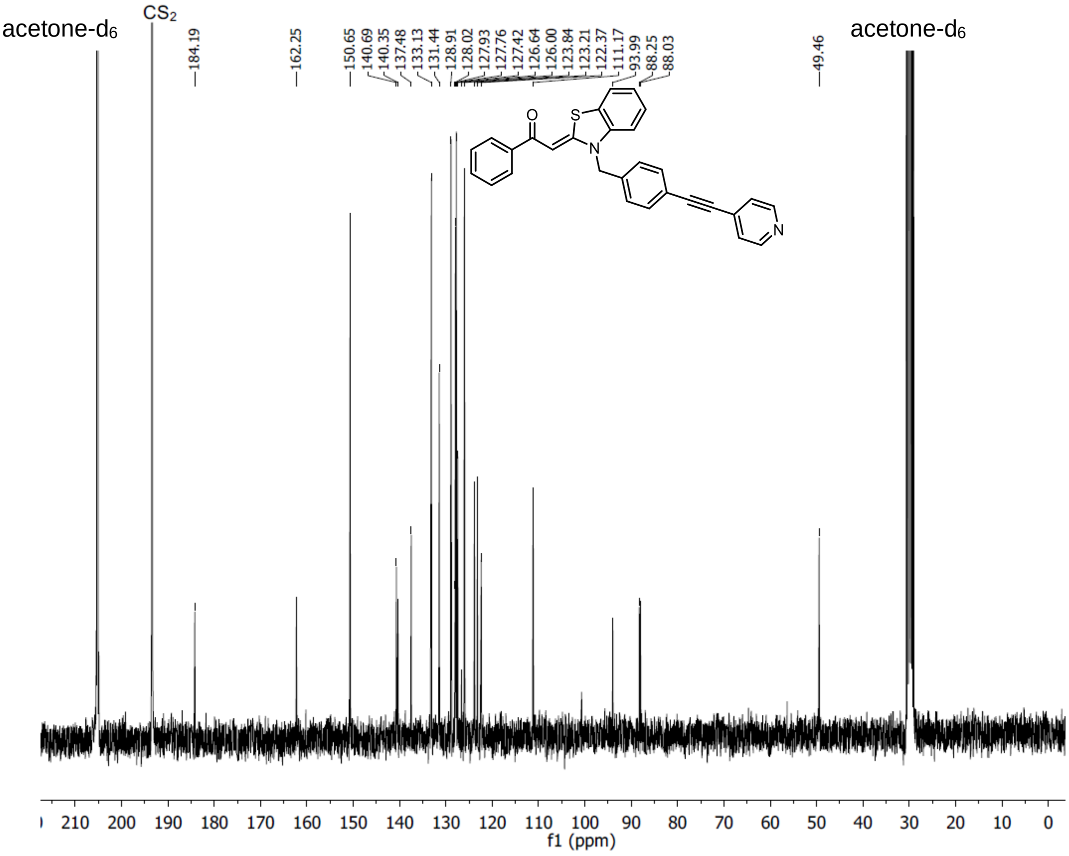

Figure S28. ^{13}C NMR spectrum (*Z*)-1-phenyl-2-(3-(4-(pyridine-4-ylethynyl)benzyl)benzo[*d*]thiazol-2(3*H*)-ylidene)ethan-1-one (**3j**) (acetone-d_6/CS_2 5:1, 75 MHz, 298 K).

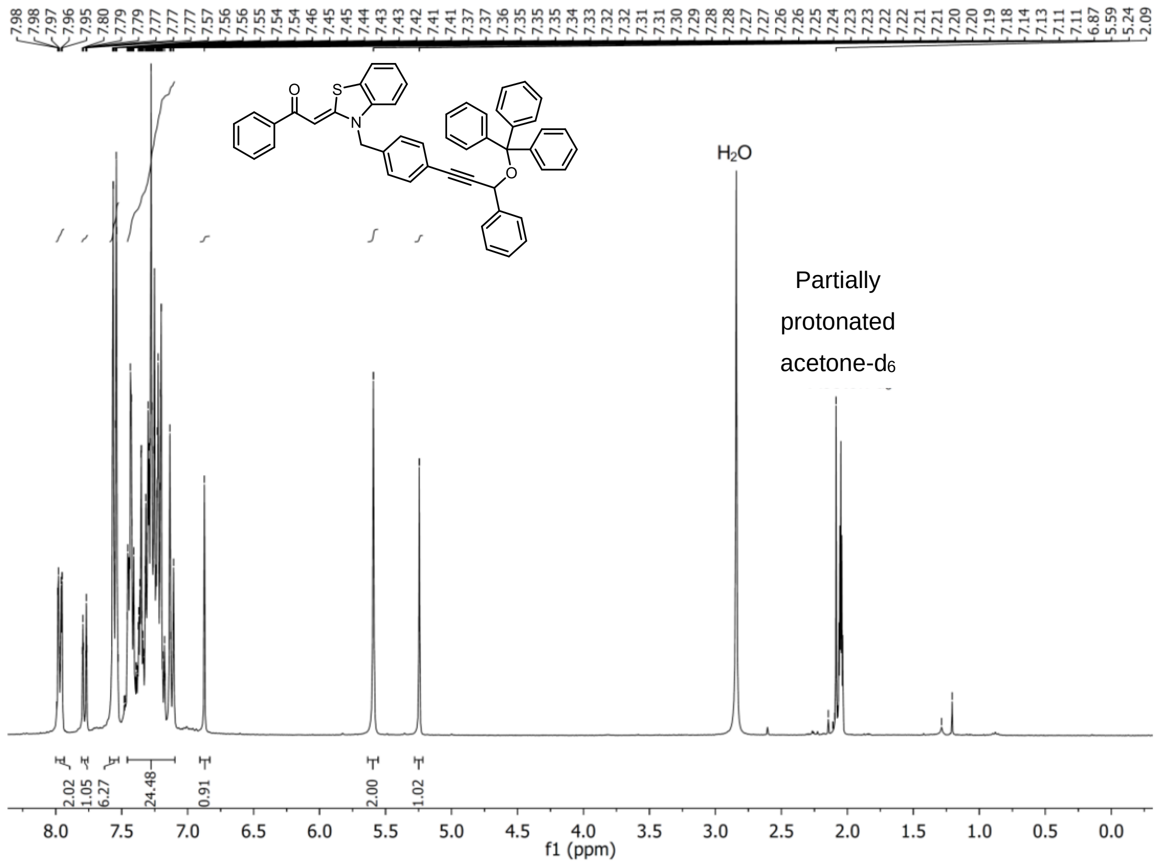

Figure S29. ^{1}H NMR spectrum (*Z*)-1-phenyl-2-(3-(4-(3-phenyl-3-(trityloxy)prop-1-yn-1-yl)benzyl)benzo[*d*]thiazol-2(3*H*)-ylidene)ethan-1-one (**3k**) (acetone-d$_6$, 300 MHz, 298 K).

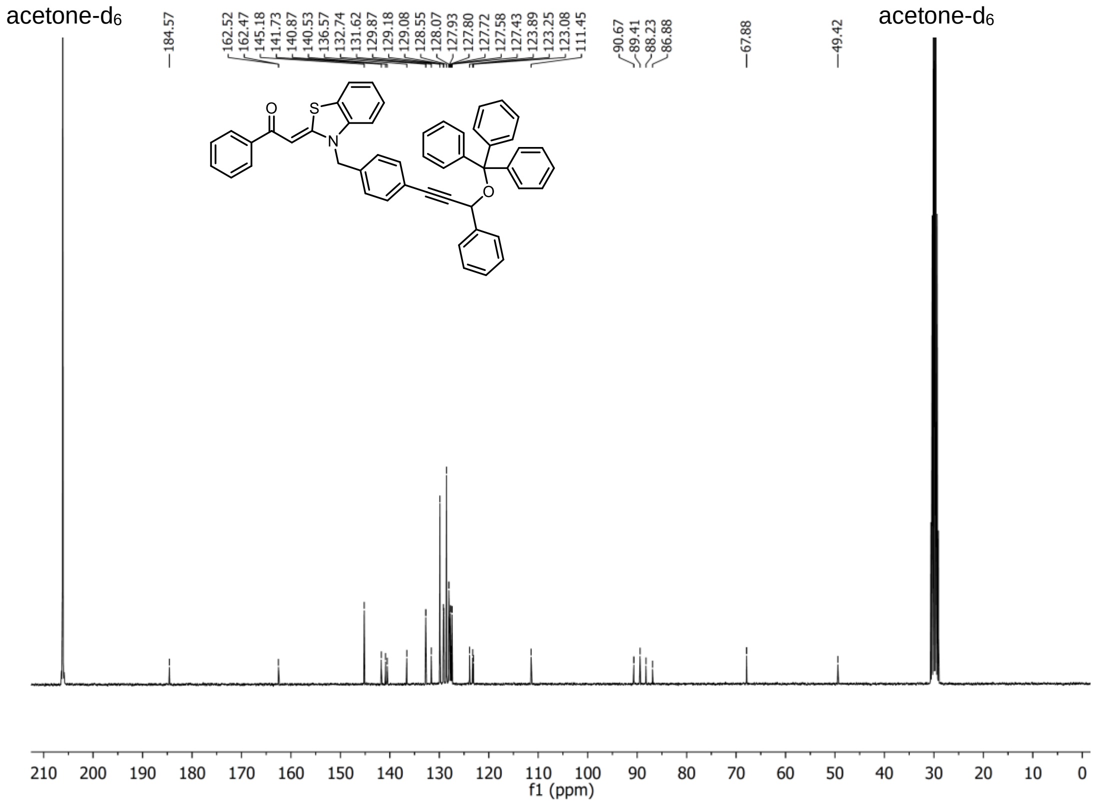

Figure S30. ^{13}C NMR spectrum (*Z*)-1-phenyl-2-(3-(4-(3-phenyl-3-(trityloxy)prop-1-yn-1-yl)benzyl)benzo[*d*]thiazol-2(3*H*)-ylidene)ethan-1-one (**3k**) (acetone-d$_6$, 75 MHz, 298 K).

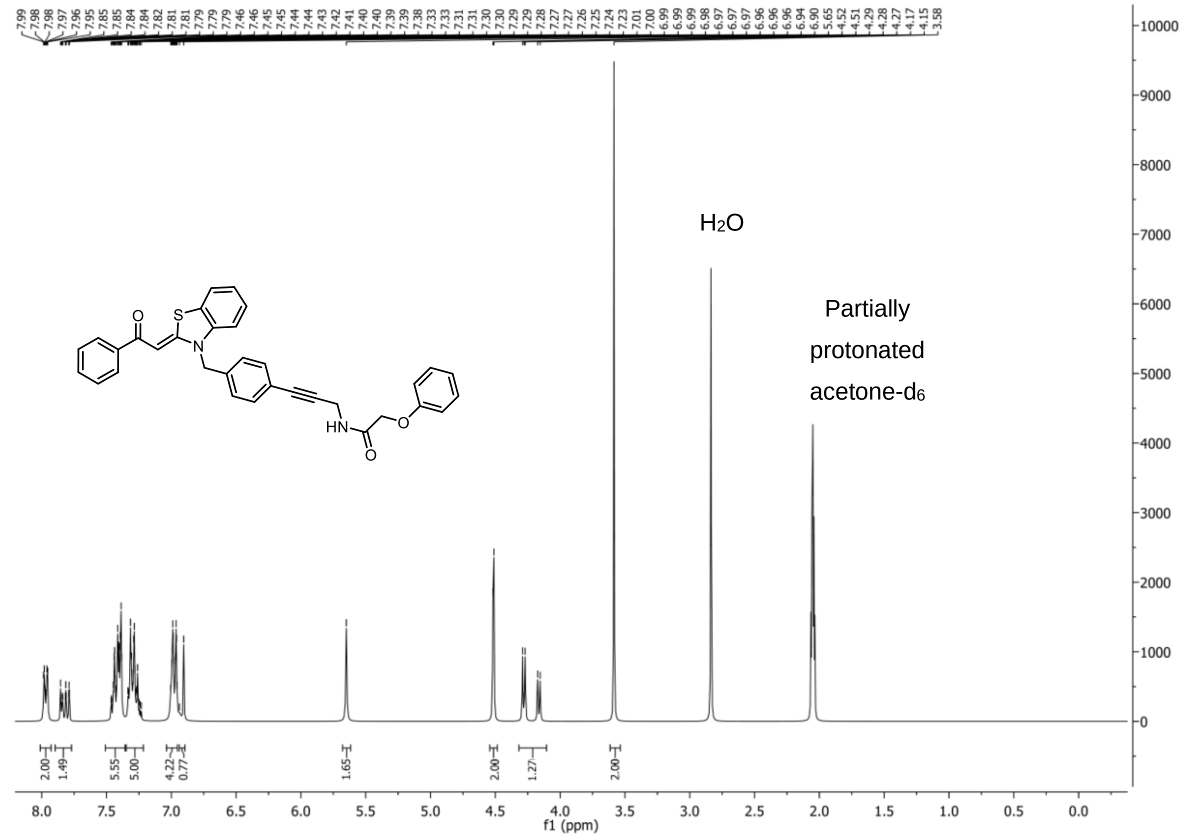

Figure S31. ^{1}H NMR spectrum (*Z*)-*N*-(3-(4-((2-(2-oxo-2-phenylethylidene)benzo[*d*]thiazol-3(2*H*)-yl)methyl)phenyl)prop-2-yn-1-yl)-2-phenoxyacetamide (**3l**) (acetone-d$_6$, 300 MHz, 298 K).

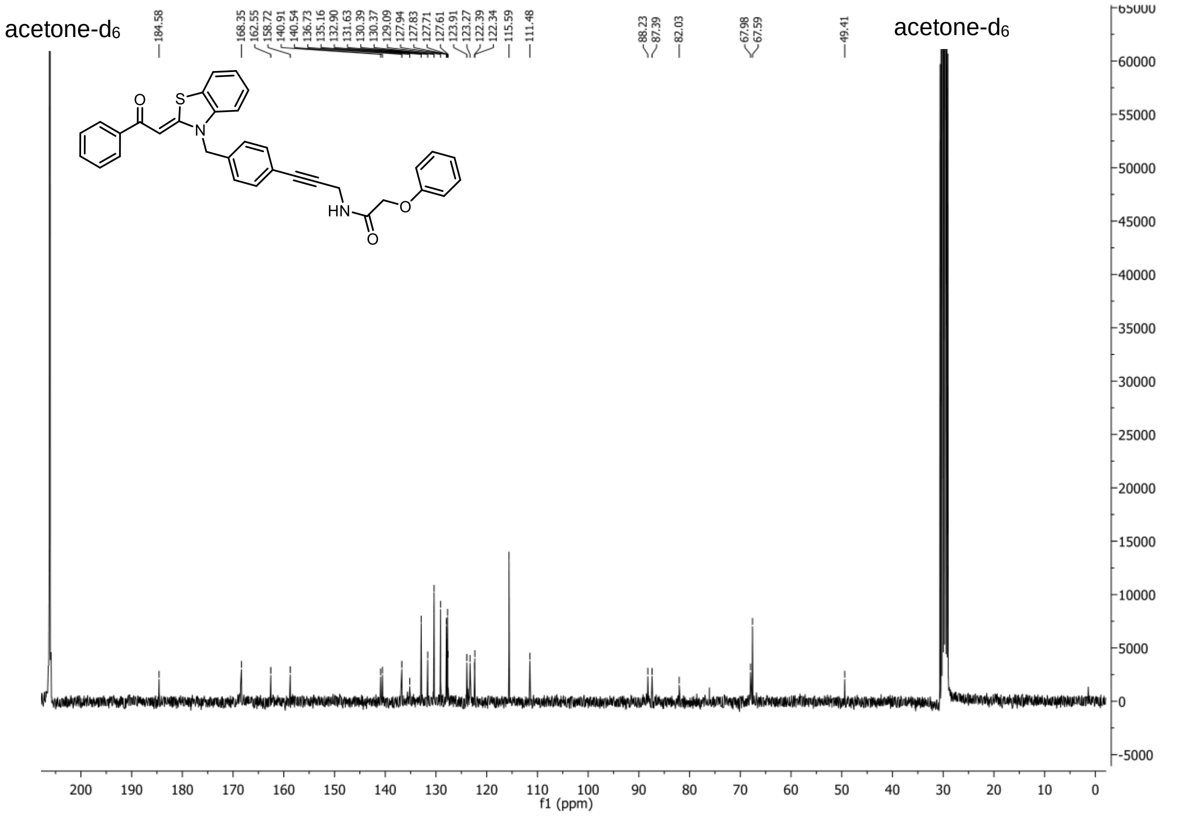

Figure S32. ^{13}C NMR spectrum (*Z*)-*N*-(3-(4-((2-(2-oxo-2-phenylethylidene)benzo[*d*]thiazol-3(2*H*)-yl)methyl)phenyl)prop-2-yn-1-yl)-2-phenoxyacetamide (**3l**) (acetone-d$_6$, 75 MHz, 298 K).

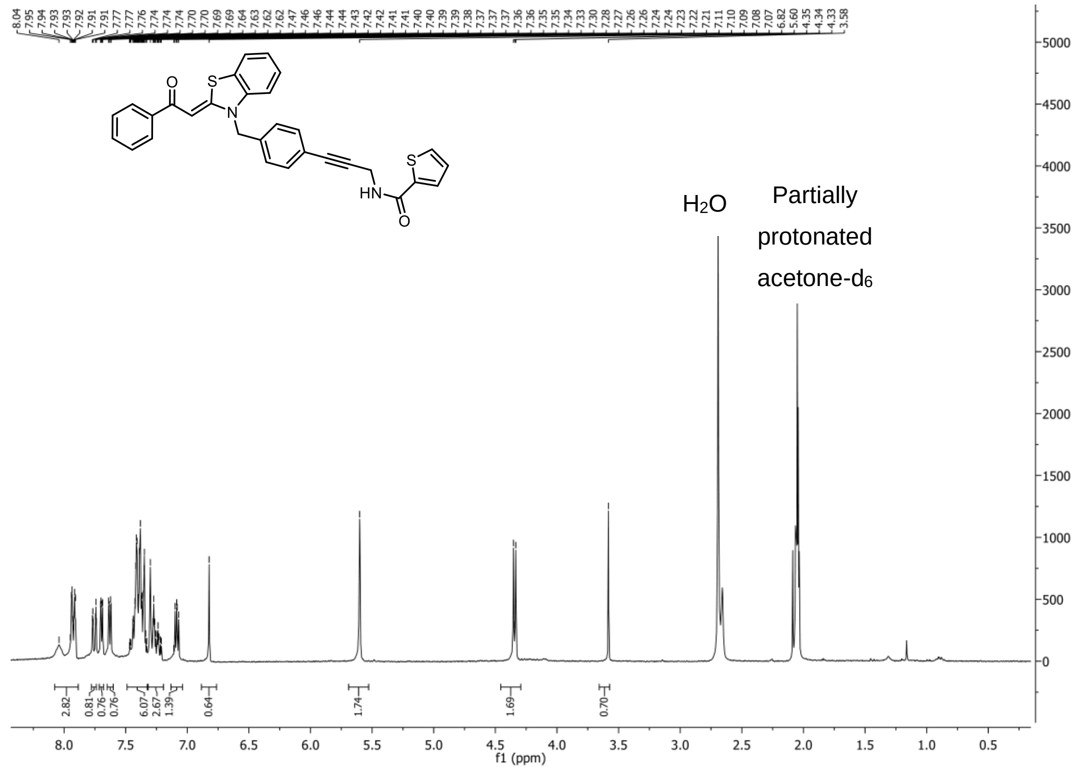

Figure S33. ^{1}H NMR spectrum (*Z*)-*N*-(3-(4-((2-(2-oxo-2-phenylethylidene)benzo[*d*]thiazol-3(2*H*)-yl)methyl)phenyl)prop-2-yn-1-yl)thiophen-2-carboxamide (**3m**) (acetone-d$_6$/CS_2 5:1, 300 MHz, 298 K).

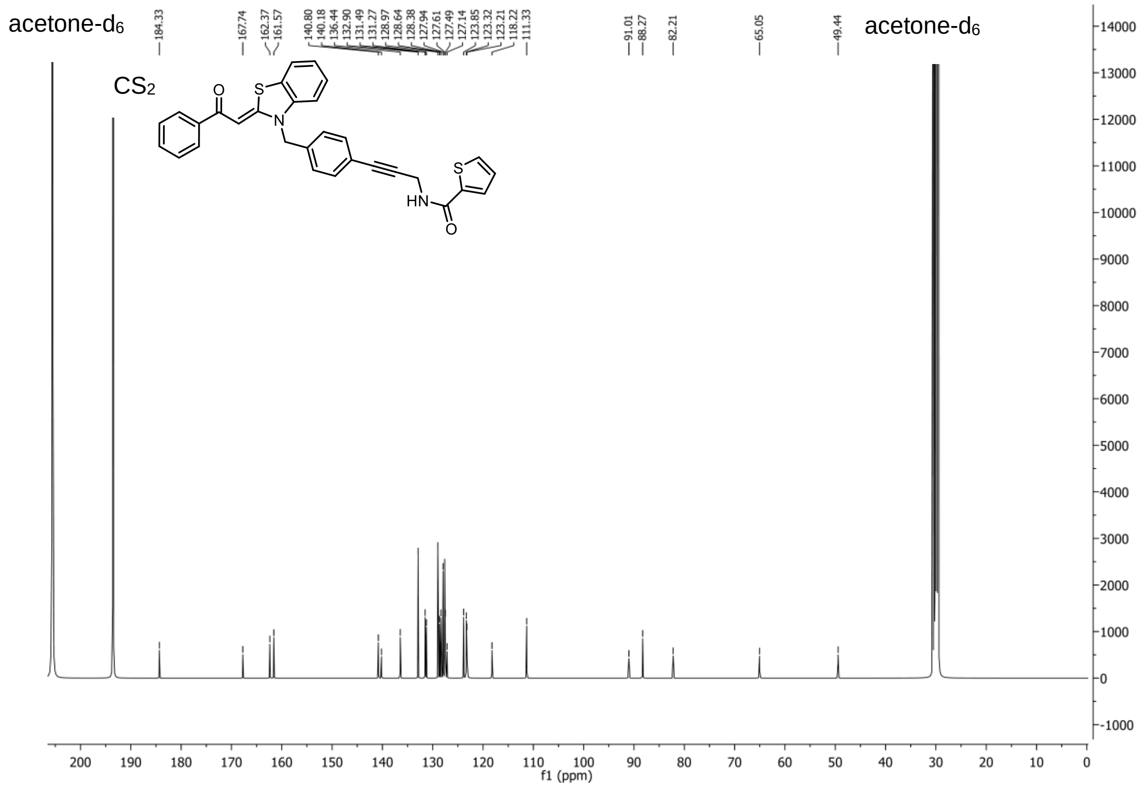

Figure S34. ^{13}C NMR spectrum (*Z*)-*N*-(3-(4-((2-(2-oxo-2-phenylethylidene)benzo[*d*]thiazol-3(2*H*)-yl)methyl)phenyl)prop-2-yn-1-yl)thiophen-2-carboxamide (**3m**) (acetone-d$_6$/CS_2 5:1, 75 MHz, 298 K).

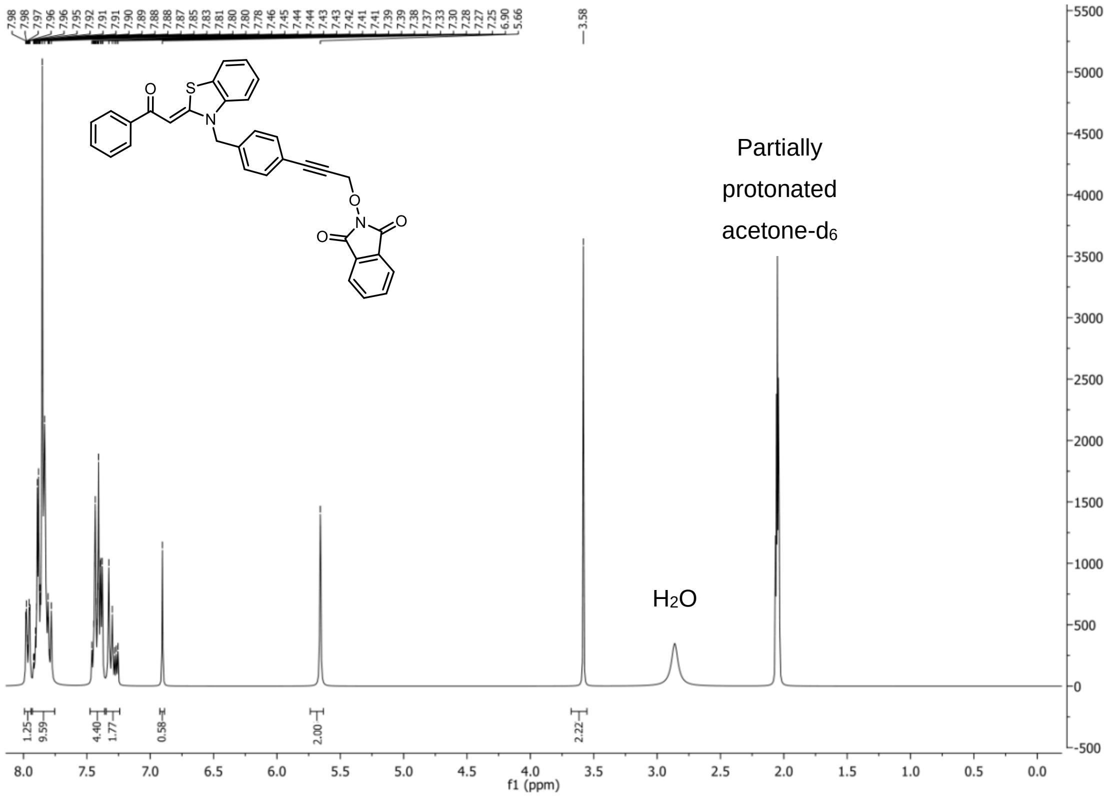

Figure S35. ^{1}H NMR spectrum (*Z*)-2-((3-(4-((2-(2-oxo-2-phenylethylidene)benzo[*d*]thiazol-3(2*H*)-yl)methyl)phenyl)prop-2-yn-1-yl)oxy)isoindolin-1,3-dione (**3n**) (acetone-d$_6$, 300 MHz, 298 K).

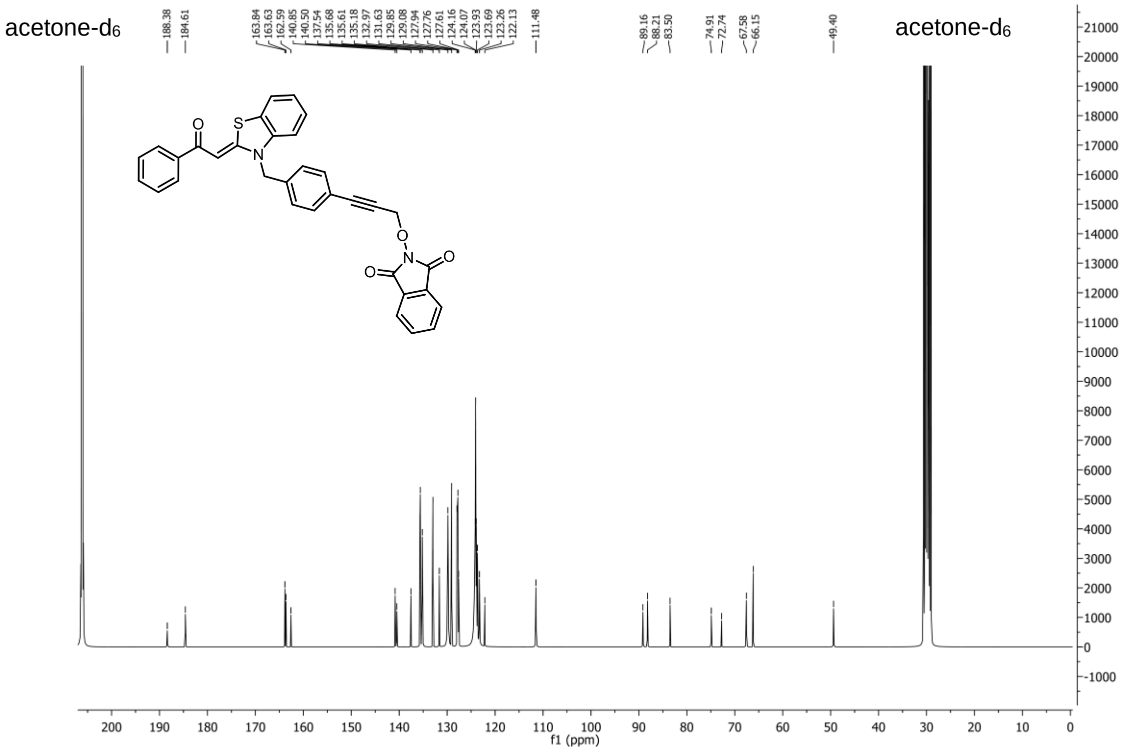

Figure S36. ^{13}C NMR spectrum (*Z*)-2-((3-(4-((2-(2-oxo-2-phenylethylidene)benzo[*d*]thiazol-3(2*H*)-yl)methyl)phenyl)prop-2-yn-1-yl)oxy)isoindolin-1,3-dione (**3n**) (acetone-d$_6$, 75 MHz, 298 K).

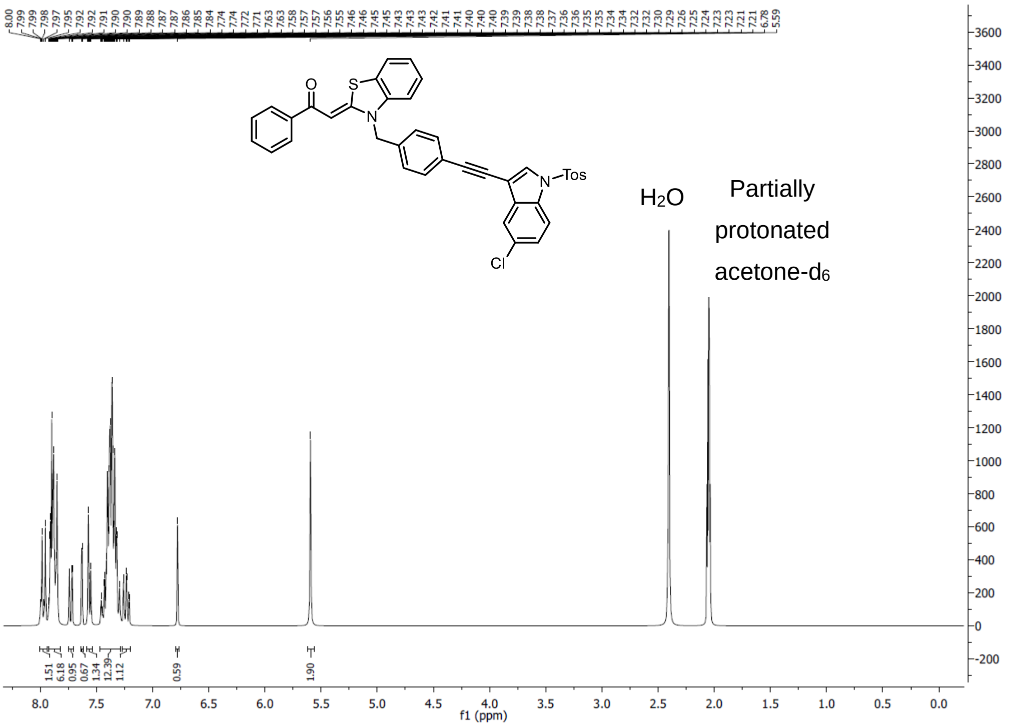

Figure S37. ^{1}H NMR spectrum (*Z*)-2-(3-(4-((5-chloro-1-tosyl-1*H*-indol-3-yl)ethynyl)benzyl)benzo[*d*]thiazol-2(3*H*)-ylidene)-1-phenylethan-1-one (**3o**) (acetone-d_6/CS_2 5:2, 300 MHz, 298 K).

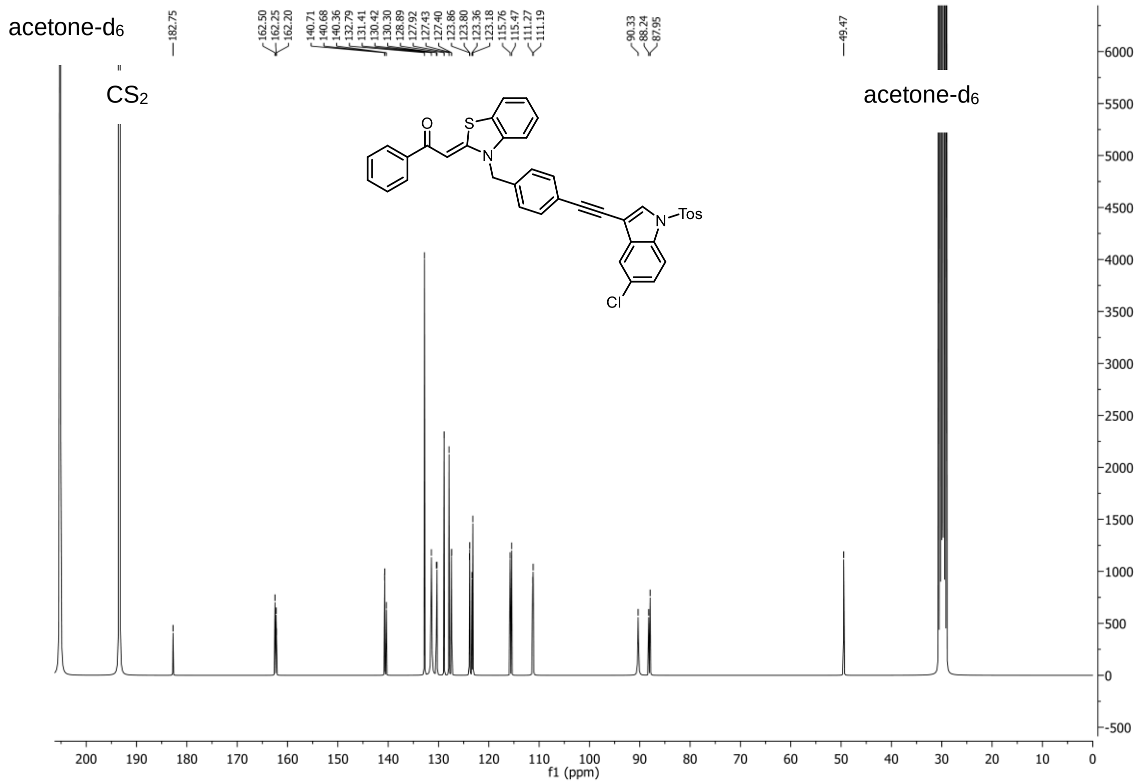

Figure S38. ^{13}C NMR spectrum (*Z*)-2-(3-(4-((5-chloro-1-tosyl-1*H*-indol-3-yl)ethynyl)benzyl)benzo[*d*]thiazol-2(3*H*)-ylidene)-1-phenylethan-1-one (**3o**) (acetone-d_6/CS_2 5:2, 75 MHz, 298 K).

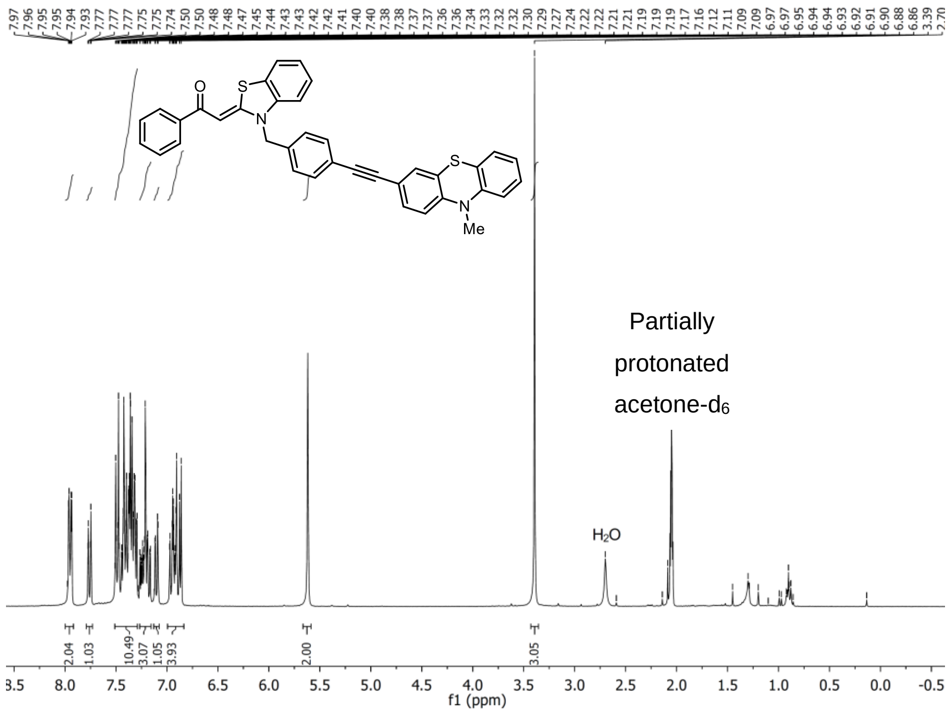

Figure S39. ^{1}H NMR spectrum (*Z*)-2-(3-(4-((10-methyl-10*H*-phenothiazin-3-yl)ethynyl)benzyl)benzo[*d*]thiazol-2(3*H*)-ylidene)-1-phenylethan-1-one (**3p**) (acetone-d_6/CS_2 5:1, 300 MHz, 298 K).

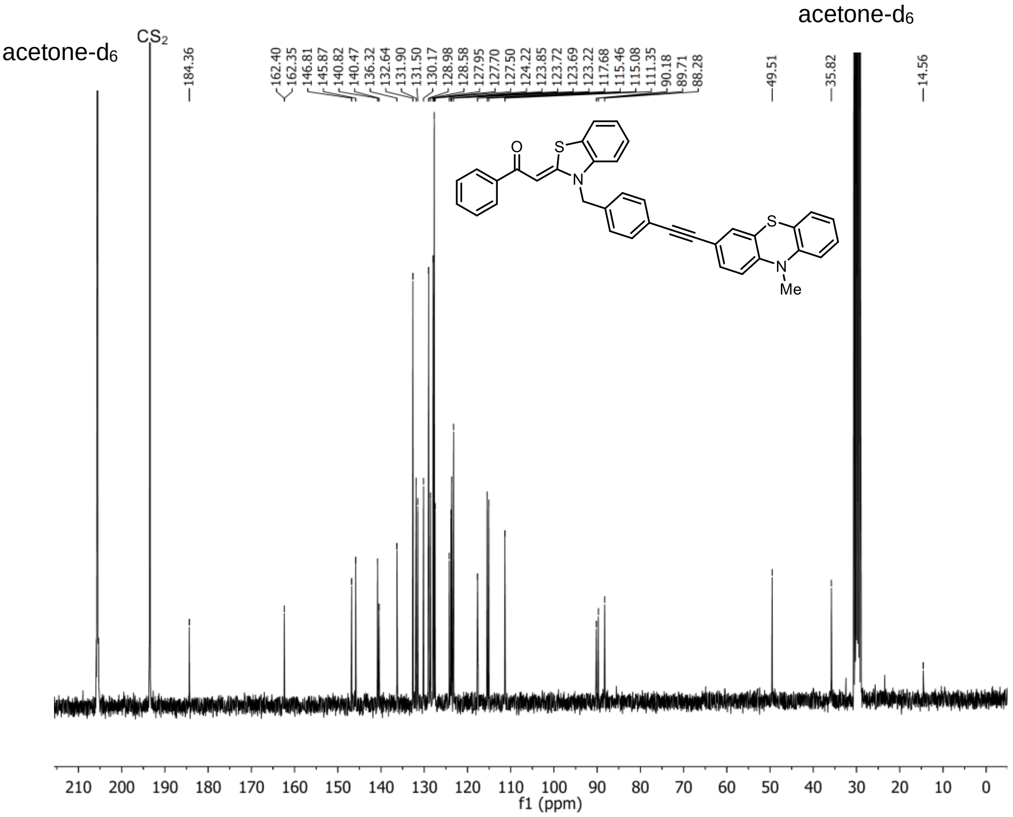

Figure S40. ^{13}C NMR spectrum (*Z*)-2-(3-(4-((10-methyl-10*H*-phenothiazin-3-yl)ethynyl)benzyl)benzo[*d*]thiazol-2(3*H*)-ylidene)-1-phenylethan-1-one (**3p**) (acetone-d_6/CS_2 5:1, 75 MHz, 298 K).

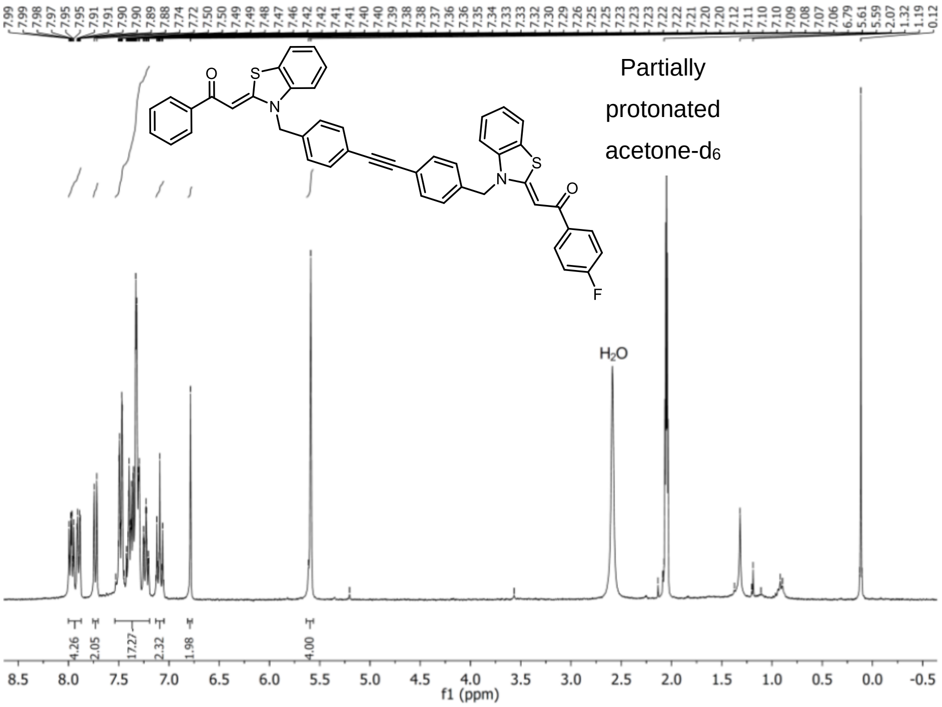

Figure S41. ^{1}H NMR spectrum (*Z*)-1-(4-fluorophenyl)-2-(3-(4-((4-(((Z)-2-(2-oxo-2-phenylethylidene)benzo[*d*]thiazol-3(2*H*)-yl)methyl)phenyl)ethynyl)benzyl)benzo[*d*]thiazol-2(3*H*)-ylidene)ethan-1-one (**3q**) (acetone-d$_6$/CS$_2$ 5:2, 300 MHz, 298 K).

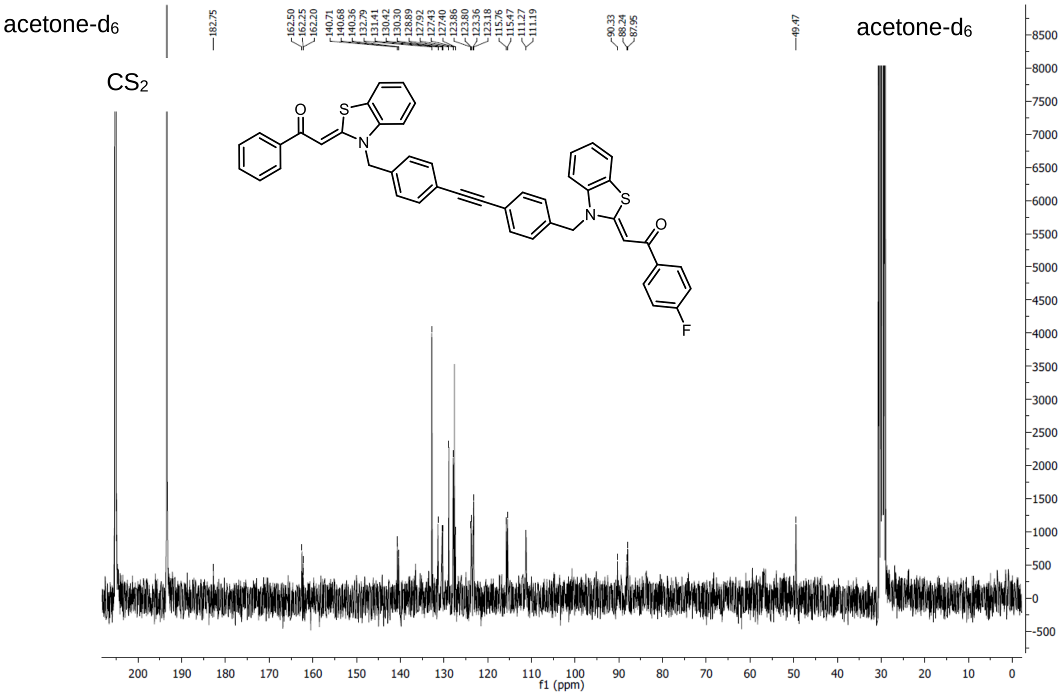

Figure S42. ^{13}C NMR spectrum (*Z*)-1-(4-fluorophenyl)-2-(3-(4-((4-(((Z)-2-(2-oxo-2-phenylethylidene)benzo[*d*]thiazol-3(2*H*)-yl)methyl)phenyl)ethynyl)benzyl)benzo[*d*]thiazol-2(3*H*)-ylidene)ethan-1-one (**3q**) (acetone-d$_6$/CS$_2$ 5:2, 75 MHz, 298 K).

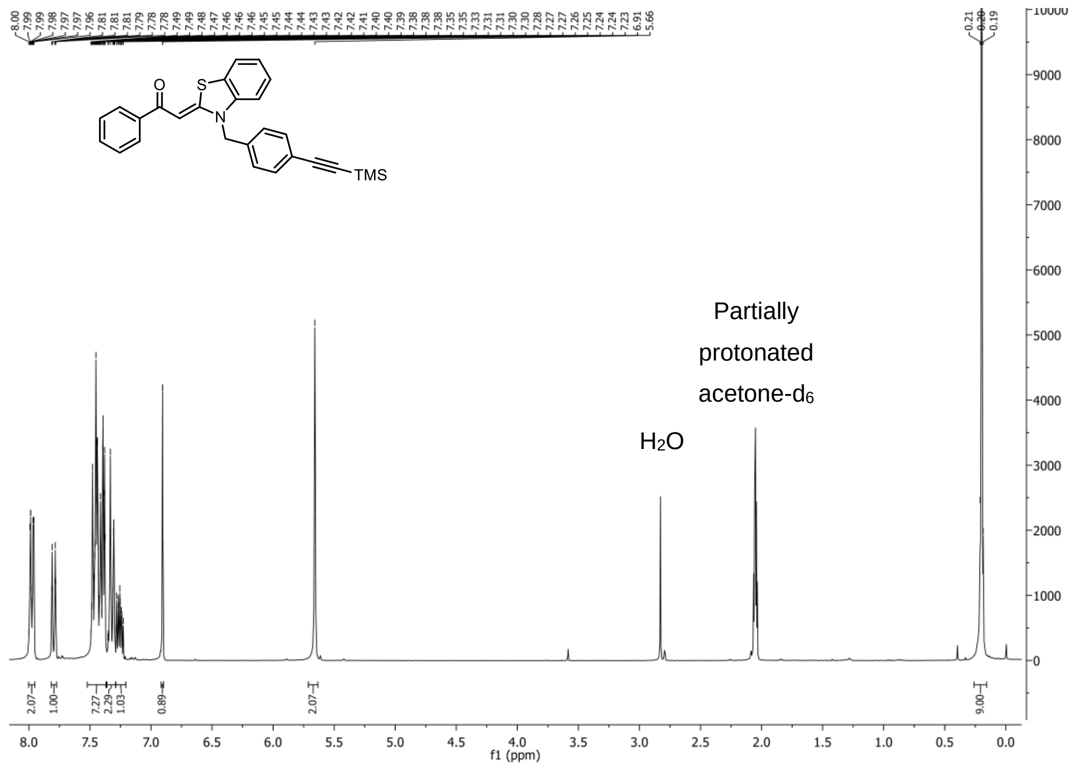

Figure S43. ^{1}H NMR spectrum (*Z*)-1-Phenyl-2-(3-(4-((trimethylsilyl)ethynyl)benzyl)benzo[*d*]thiazol-2(3*H*)-ylidene)ethan-1-one (**3r**) (300 MHz, acetone-d_6, 293 K).

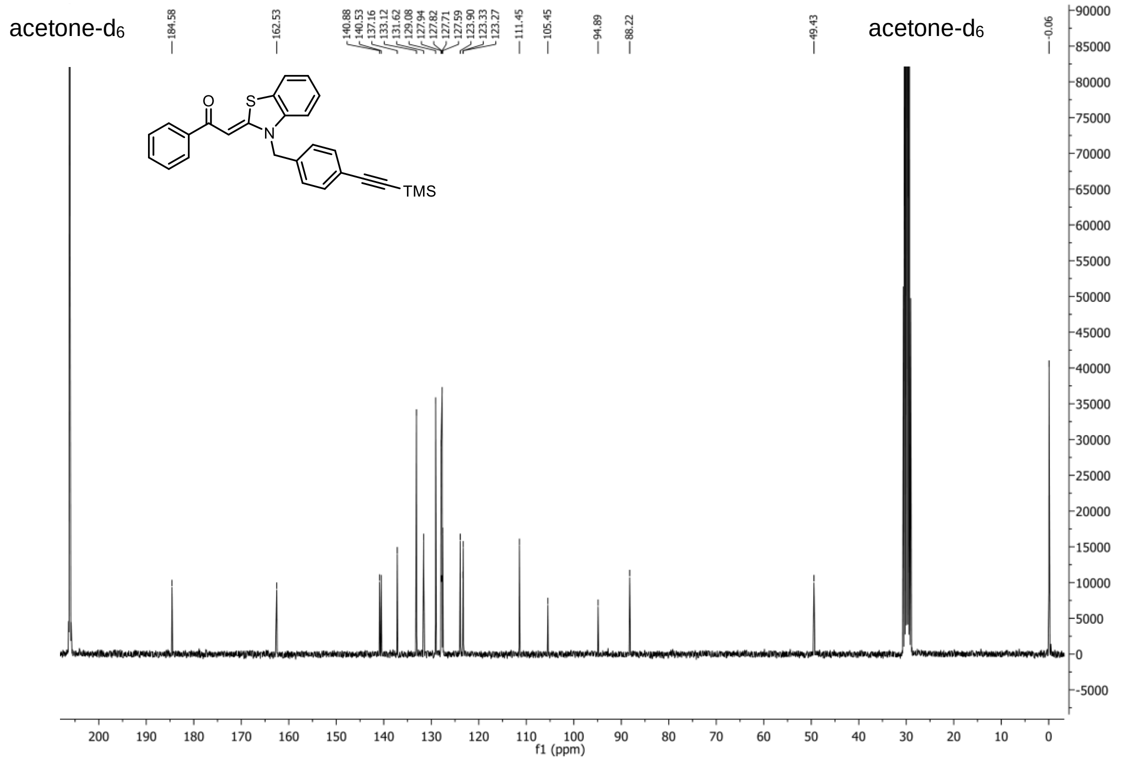

Figure S44. ^{13}C NMR spectrum (*Z*)-1-phenyl-2-(3-(4-((trimethylsilyl)ethynyl)benzyl)benzo[*d*]thiazol-2(3*H*)-ylidene)ethan-1-one (**3r**) (75 MHz, acetone-d_6, 293 K).

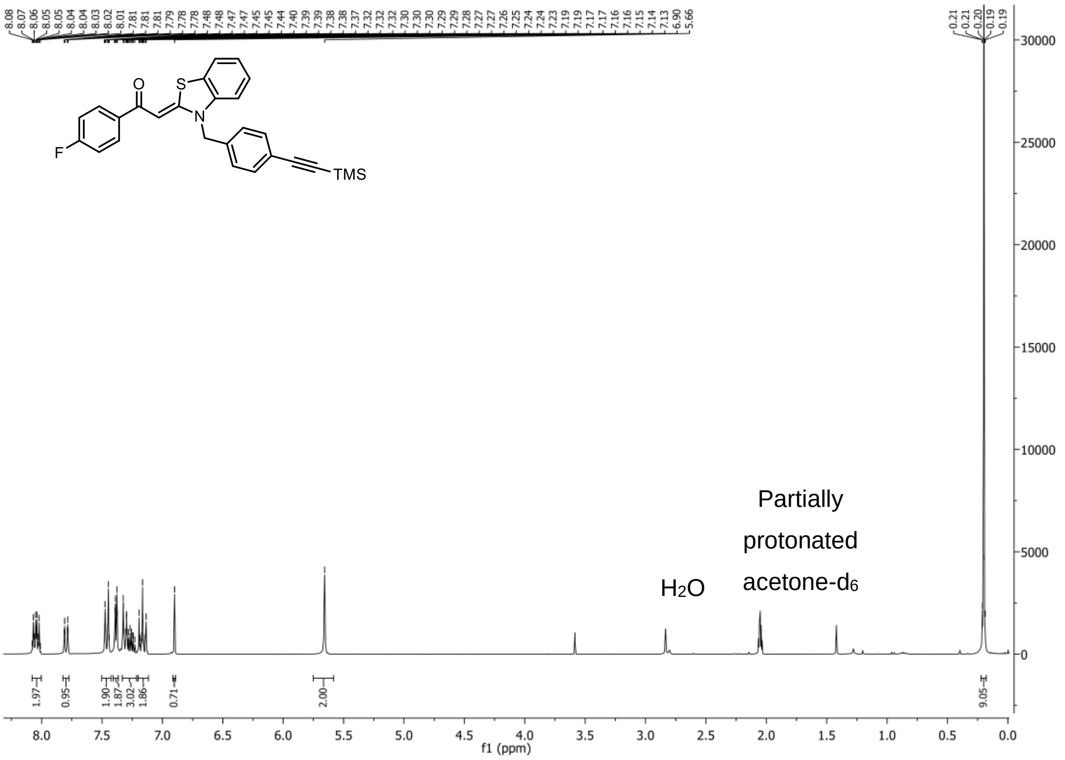

Figure S45. ^{1}H NMR spectrum (*Z*)-1-(4-fluorophenyl)-2-(3-(4-((trimethylsilyl)ethynyl)benzyl)benzo[*d*]thiazol-2(3*H*)-ylidene)ethan-1-one (**3s**) (300 MHz, acetone-d$_6$, 293 K).

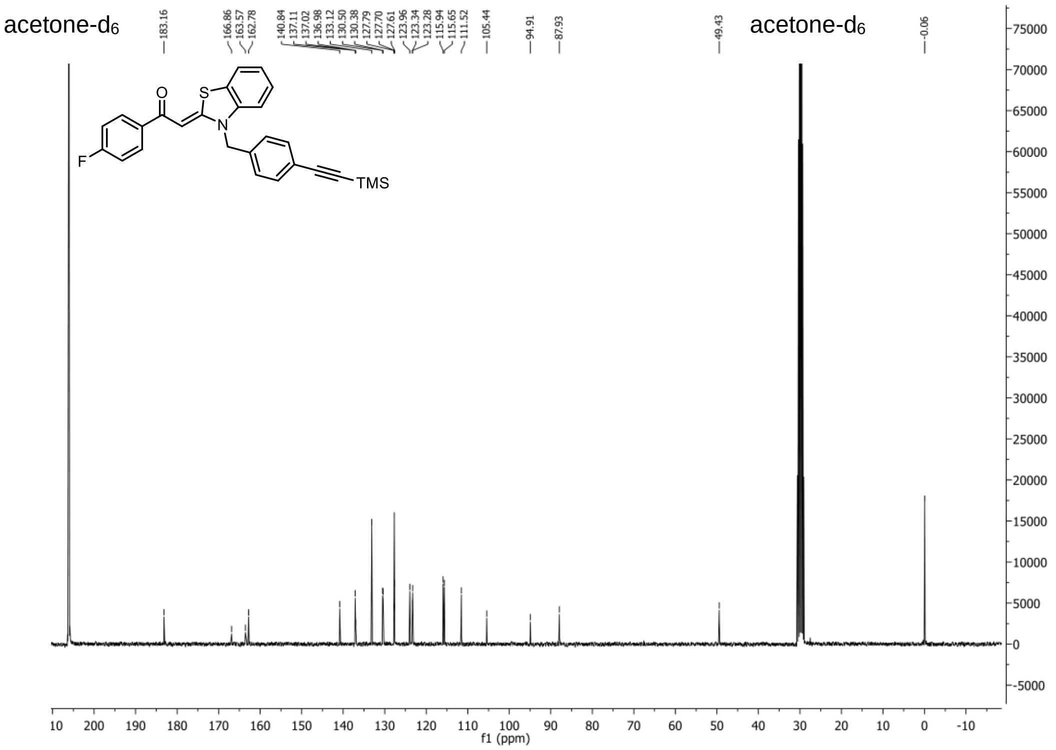

Figure S46. ^{13}C NMR spectrum (*Z*)-1-(4-fluorophenyl)-2-(3-(4-((trimethylsilyl)ethynyl)benzyl)benzo[*d*]thiazol-2(3*H*)-ylidene)ethan-1-one (**3s**) (75 MHz, acetone-d$_6$, 293 K).

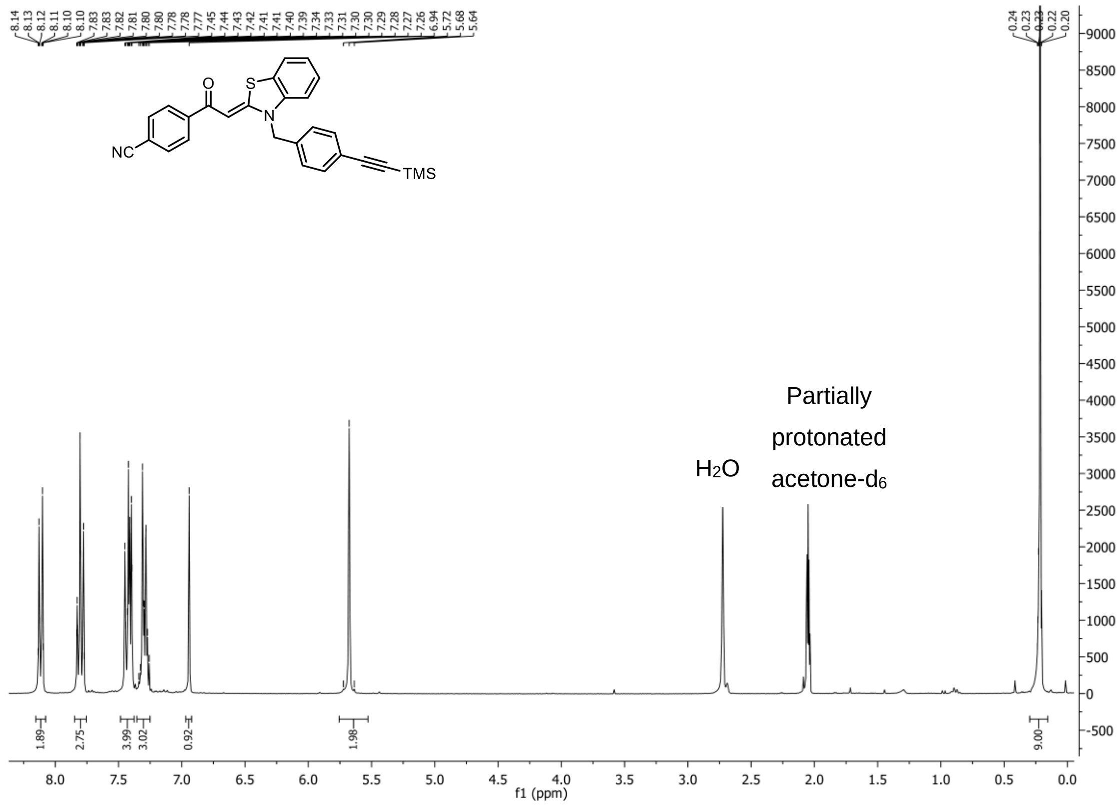

Figure S47. ^{1}H NMR spectrum (*Z*)-4-(2-(3-(4-((trimethylsilyl)ethynyl)benzyl)benzo[*d*]thiazol-2(3*H*)-ylidene)acetyl)benzonitrile (**3t**) (300 MHz, acetone-d$_6$/CS_2 5:1, 293 K).

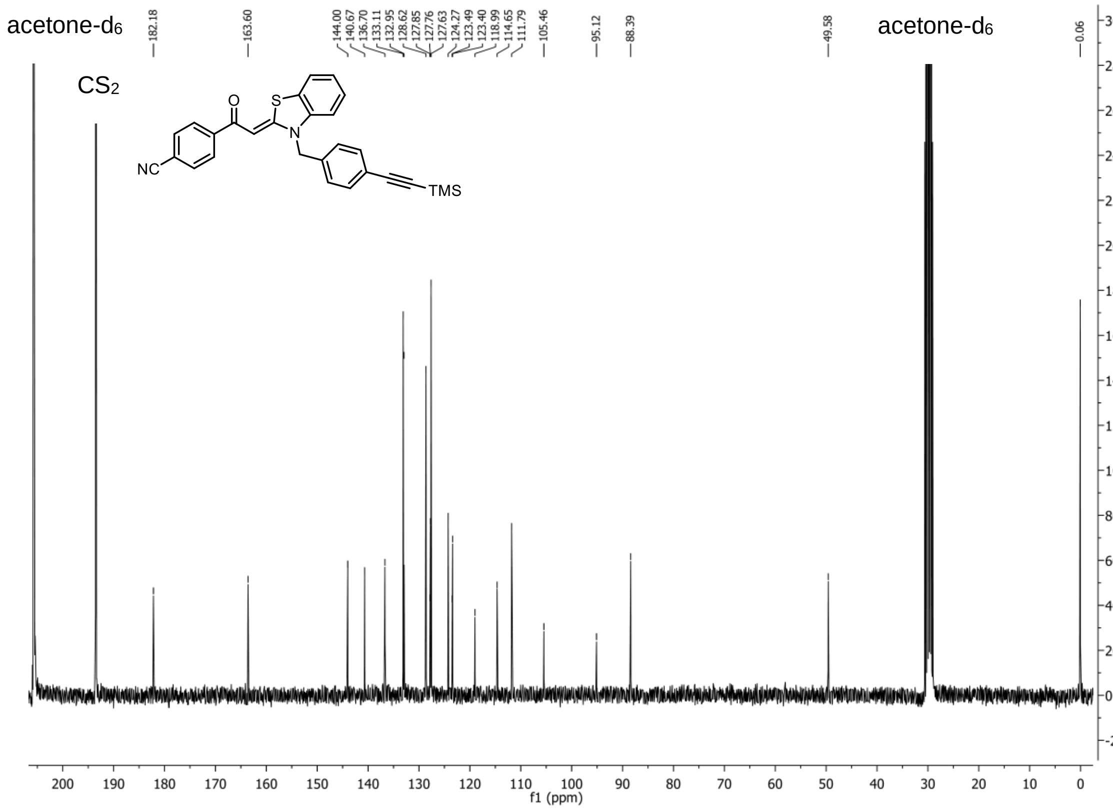

Figure S48. ^{13}C NMR spectrum (*Z*)-4-(2-(3-(4-((trimethylsilyl)ethynyl)benzyl)benzo[*d*]thiazol-2(3*H*)-ylidene)acetyl)benzonitrile (**3t**) (75 MHz, acetone-d$_6$/CS_2 5:1, 293 K).

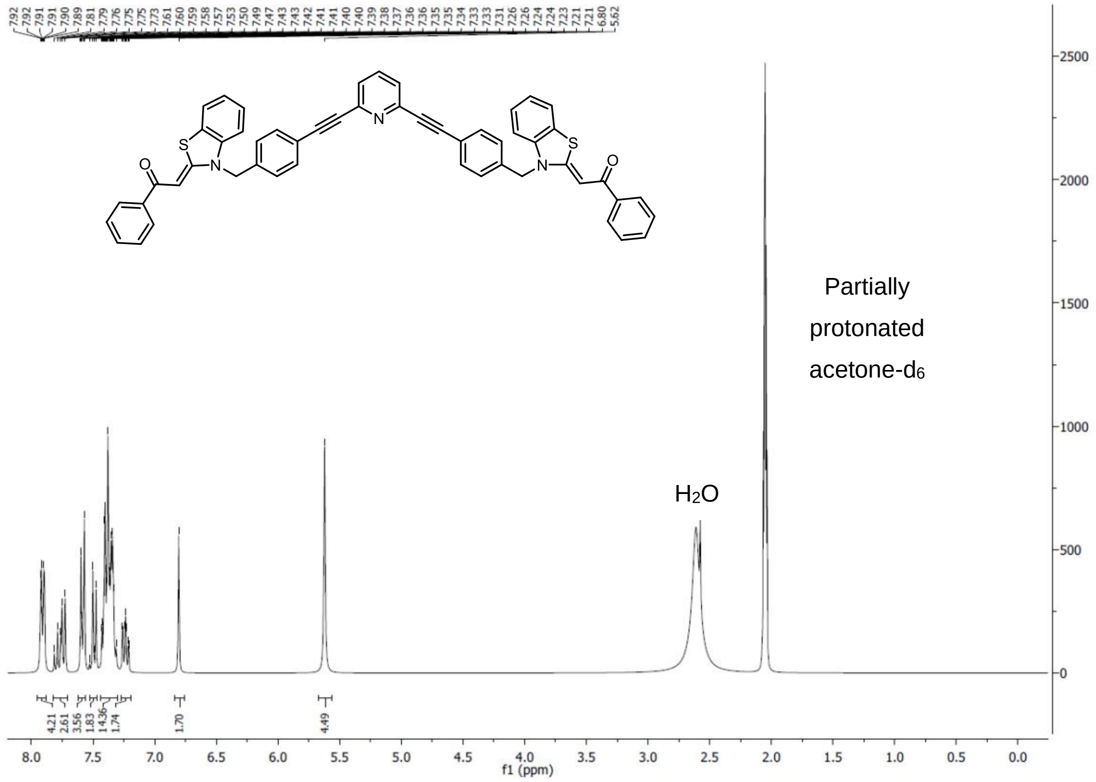

Figure S49. ^{1}H NMR spectrum (2*Z*,2'*Z*)-2,2'-((((pyridine-2,6-diylbis(ethyn-2,1-diyl))bis(4,1-phenylene))bis(methylene))bis(benzo[*d*]thiazol-3(3*H*)-yl-2(3*H*)-ylidene))bis(1-phenylethan-1-one) (**3u**) (acetone-d_6/CS_2 5:2, 300 MHz, 298 K).

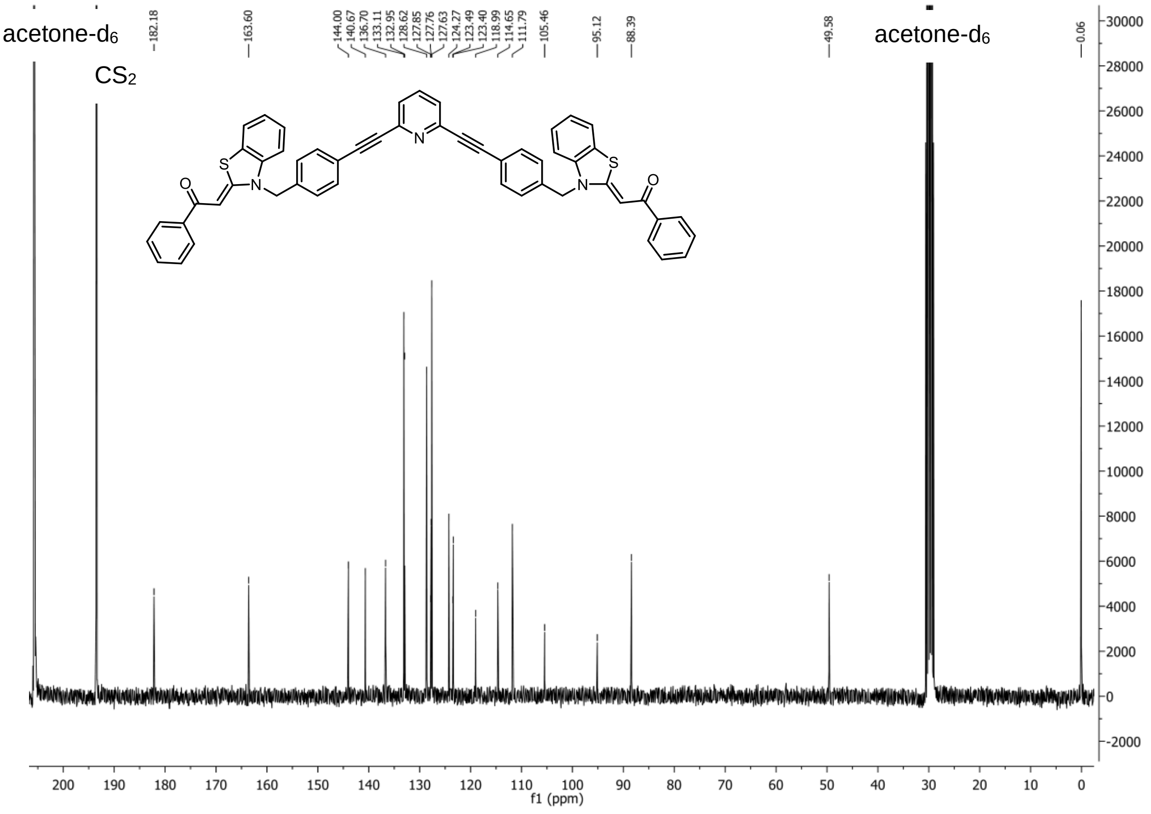

Figure S50. ^{13}C NMR spectrum (2*Z*,2'*Z*)-2,2'-((((pyridine-2,6-diylbis(ethyn-2,1-diyl))bis(4,1-phenylene))bis(methylene))bis(benzo[*d*]thiazol-3(3*H*)-yl-2(3*H*)-ylidene))bis(1-phenylethan-1-one) (**3u**) (acetone-d_6/CS_2 5:2, 75 MHz, 298 K).

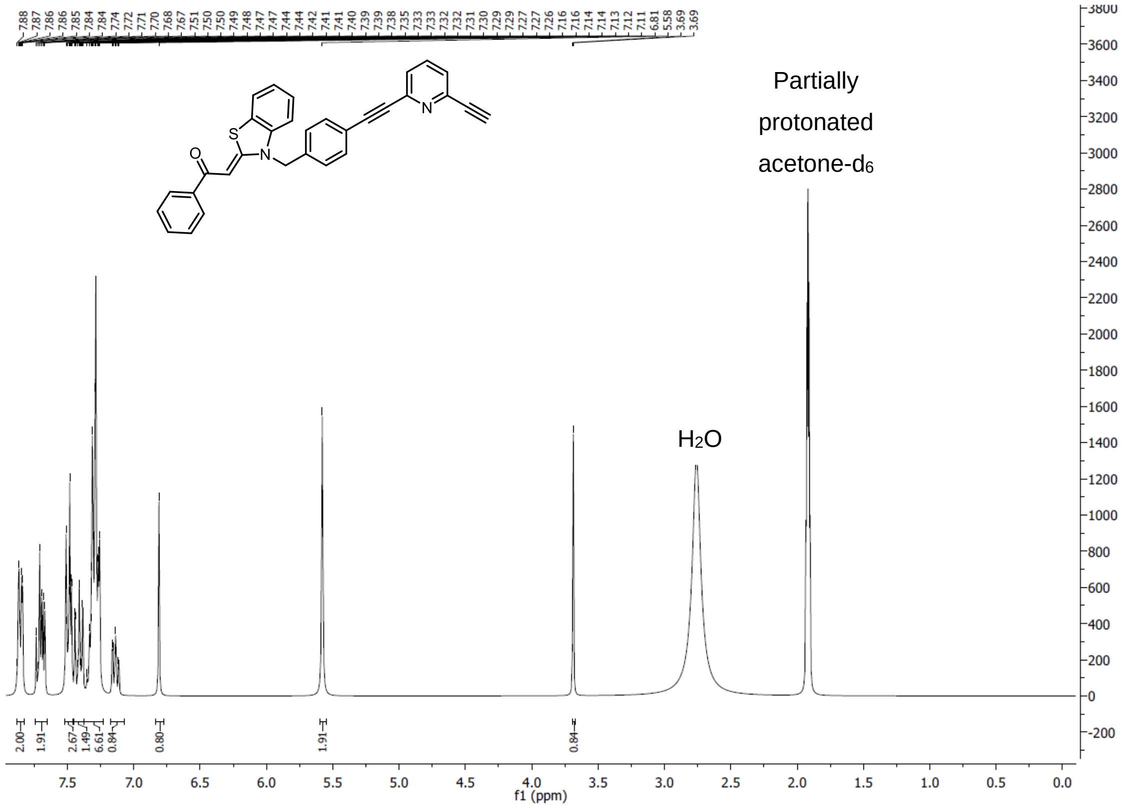

Figure S51. ^{1}H NMR spectrum (*Z*)-2-(3-(4-((6-ethynylpyridine-2-yl)ethynyl)benzyl)benzo[*d*]thiazol-2(3*H*)-ylidene)-1-phenylethan-1-one (**3v**) (acetone-d_6, 300 MHz, 298 K).

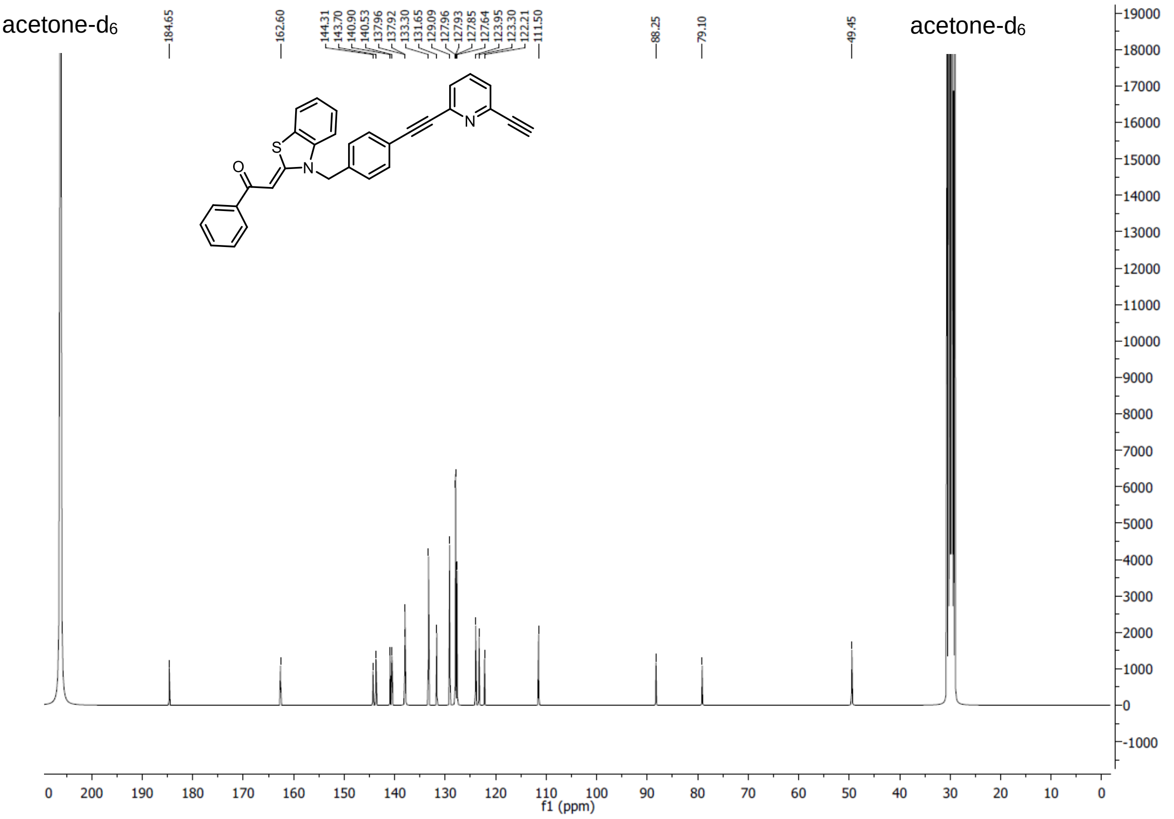

Figure S52. ^{13}C NMR spectrum (*Z*)-2-(3-(4-((6-ethynylpyridine-2-yl)ethynyl)benzyl)benzo[*d*]thiazol-2(3*H*)-ylidene)-1-phenylethan-1-one (**3v**) (acetone-d_6, 75 MHz, 298 K).

6.2 NMR spectra of deprotected alkynylated aroyl-*S*,*N*-ketene acetals 4

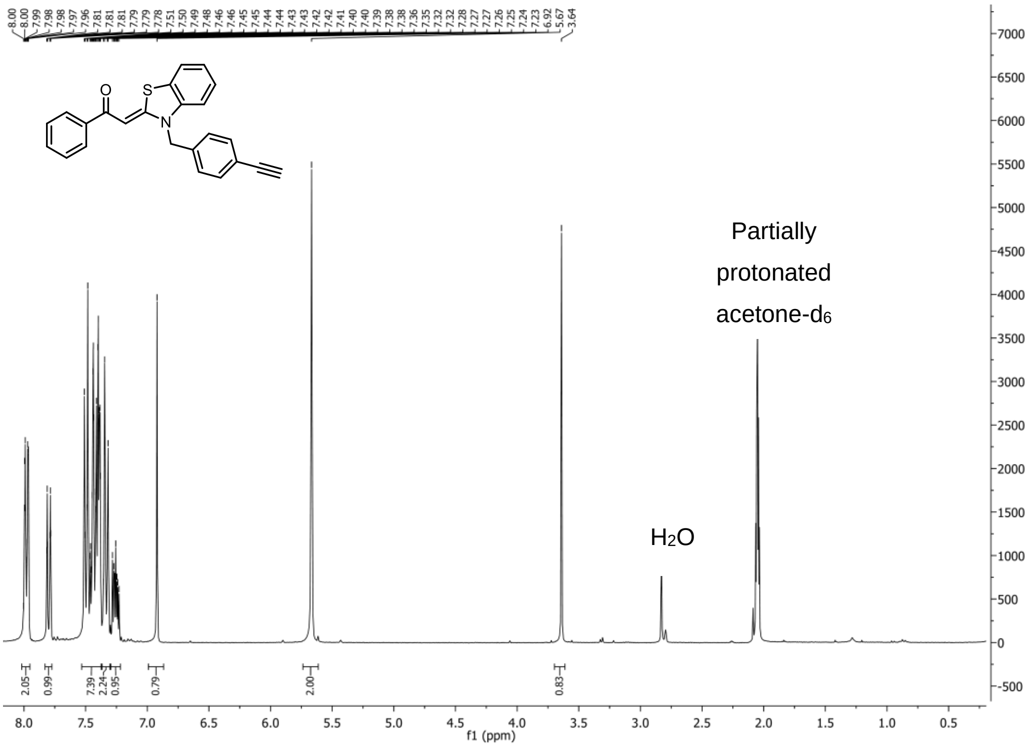

Figure S53. ^{1}H NMR spectrum (*Z*)-2-(3-(4-ethynylbenzyl)benzo[*d*]thiazol-2(3*H*)-ylidene)-1-phenylethan-1-one (**4a**) (300 MHz, acetone-d$_6$, 293 K).

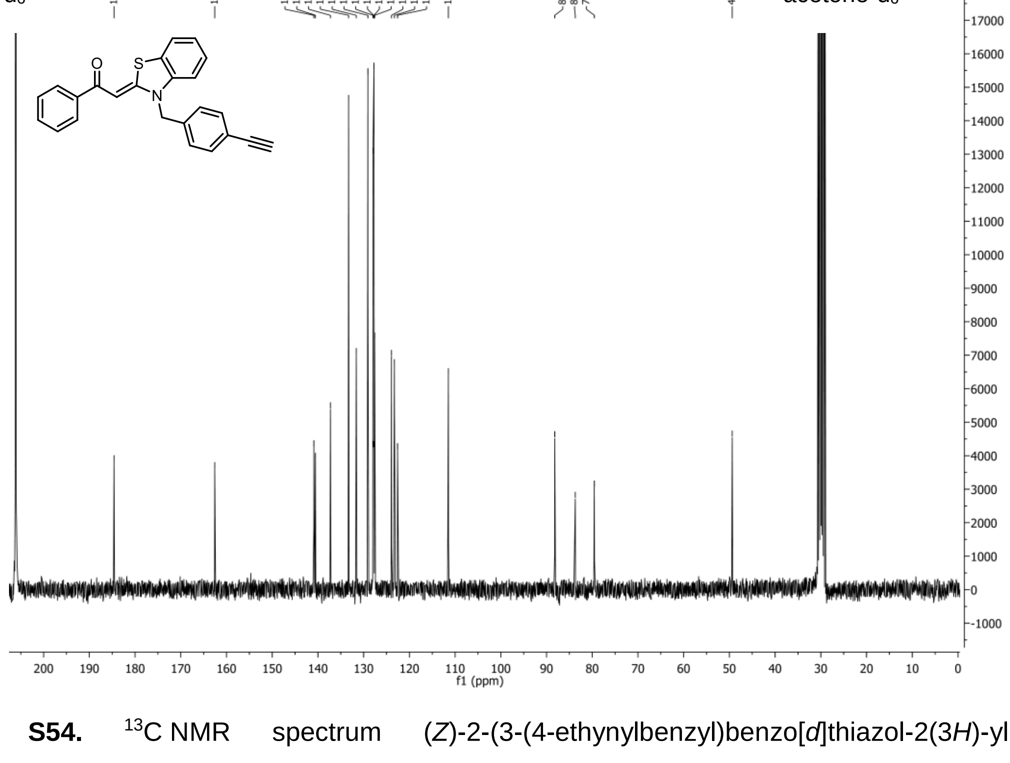

Figure S54. ^{13}C NMR spectrum (*Z*)-2-(3-(4-ethynylbenzyl)benzo[*d*]thiazol-2(3*H*)-ylidene)-1-phenylethan-1-one (**4a**) (75 MHz, acetone-d$_6$, 293 K).

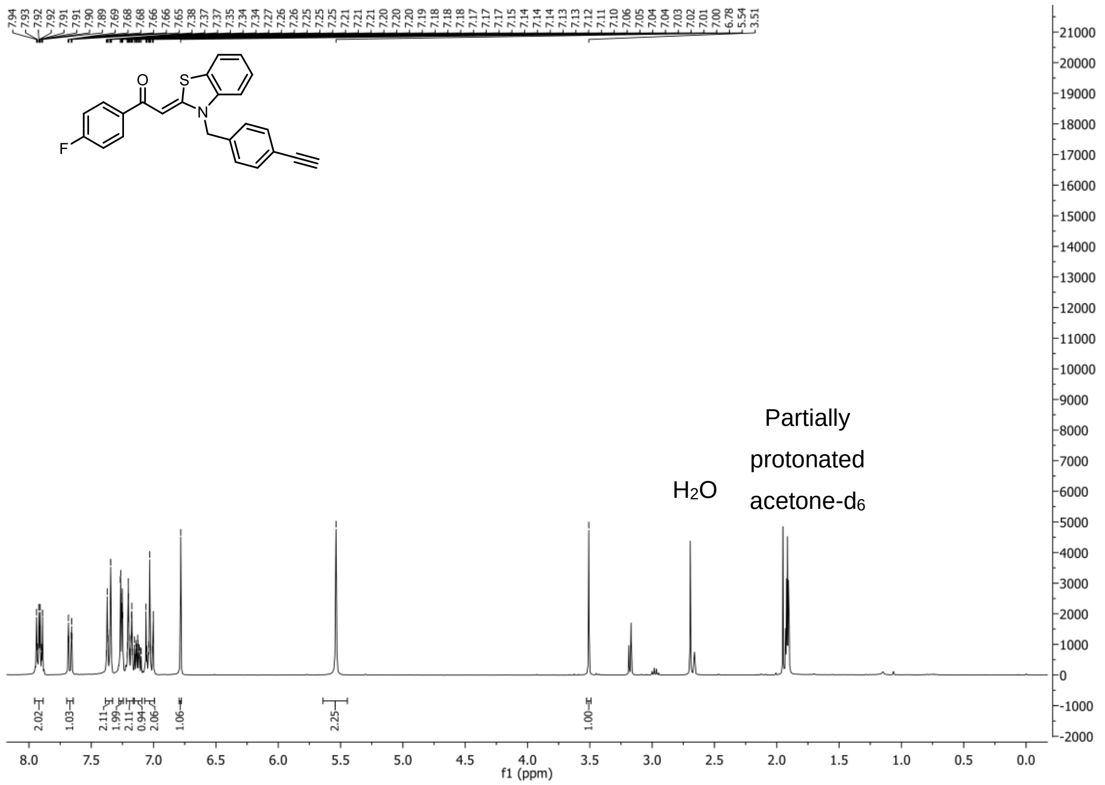

Figure S55. ^{1}H NMR spectrum (*Z*)-2-(3-(4-ethynylbenzyl)benzo[*d*]thiazol-2(3*H*)-ylidene)-1-(4-fluorophenyl)ethan-1-one (**4b**) (300 MHz, acetone-d$_6$, 293 K).

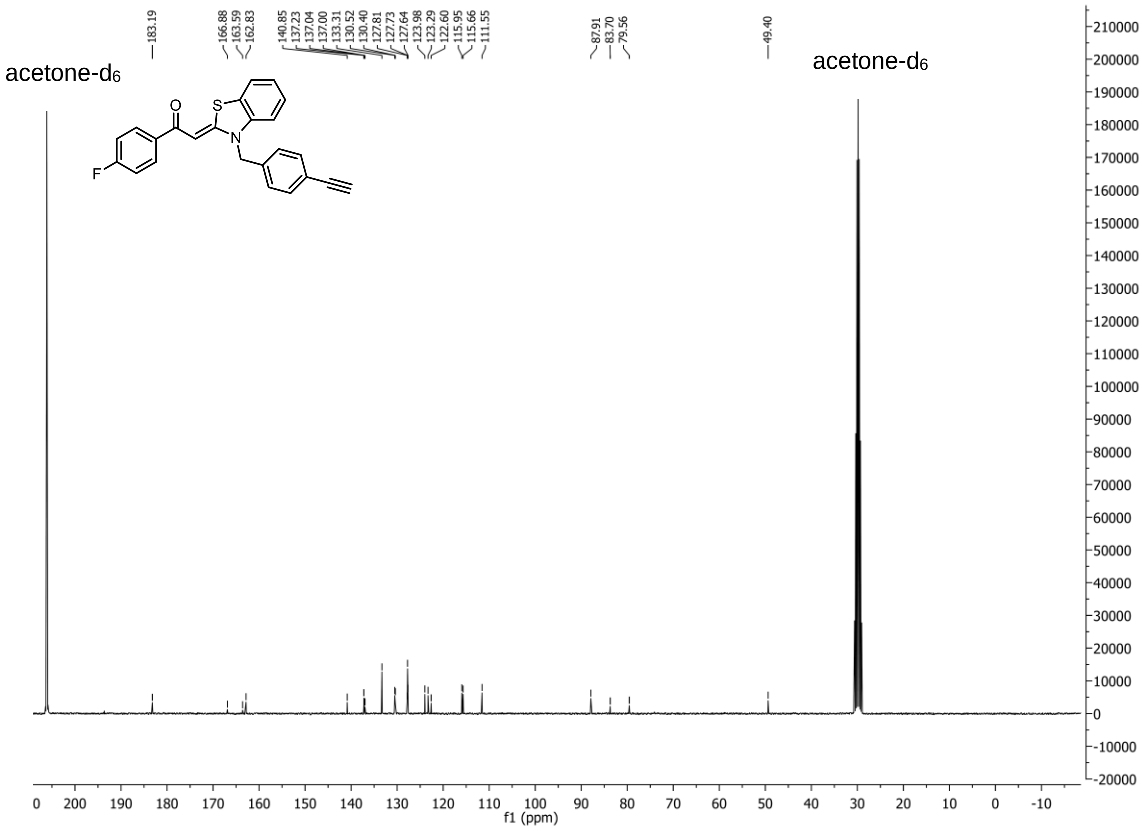

Figure S56. ^{13}C NMR spectrum (*Z*)-2-(3-(4-ethynylbenzyl)benzo[*d*]thiazol-2(3*H*)-ylidene)-1-(4-fluorophenyl)ethan-1-one (**4b**) (75 MHz, acetone-d$_6$, 293 K).

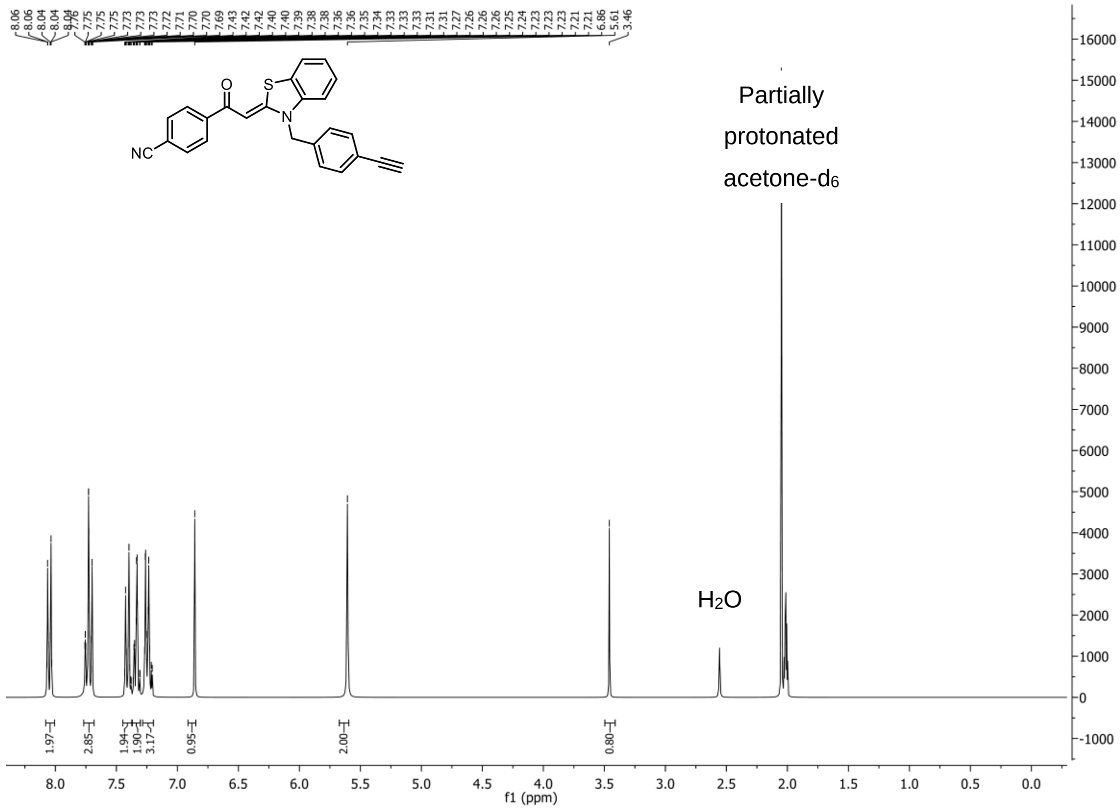

Figure S57. ^{1}H NMR spectrum (*Z*)-4-(2-(3-(4-ethynylbenzyl)benzo[*d*]thiazol-2(3*H*)-ylidene)acetyl)benzonitrile (**4c**) (300 MHz, acetone-d$_6$/CS$_2$ 5:1, 293 K).

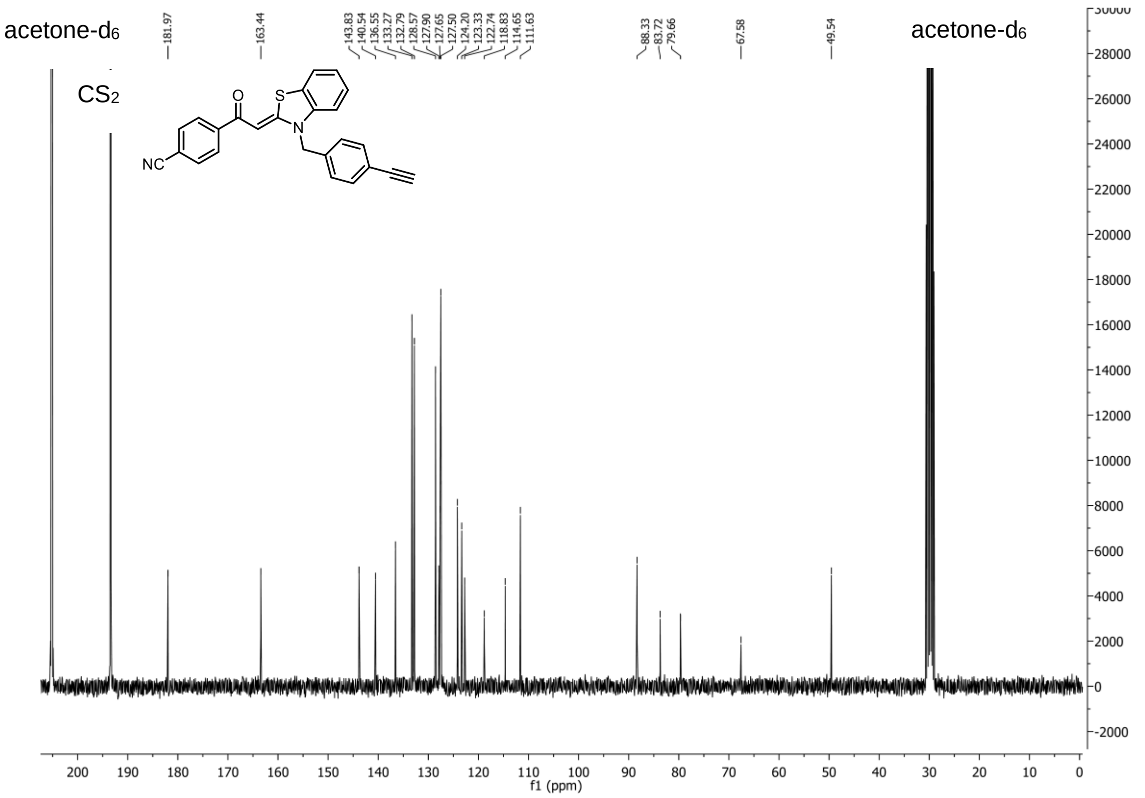

Figure S58. ^{13}C NMR spectrum ((*Z*)-4-(2-(3-(4-ethynylbenzyl)benzo[*d*]thiazol-2(3*H*)-ylidene)acetyl)benzonitrile (**4c**) (75 MHz, acetone-d$_6$/CS$_2$ 5:1, 293 K).

6.3 NMR spectra of triazole aroyl-*S*,*N*-ketene acetals 6

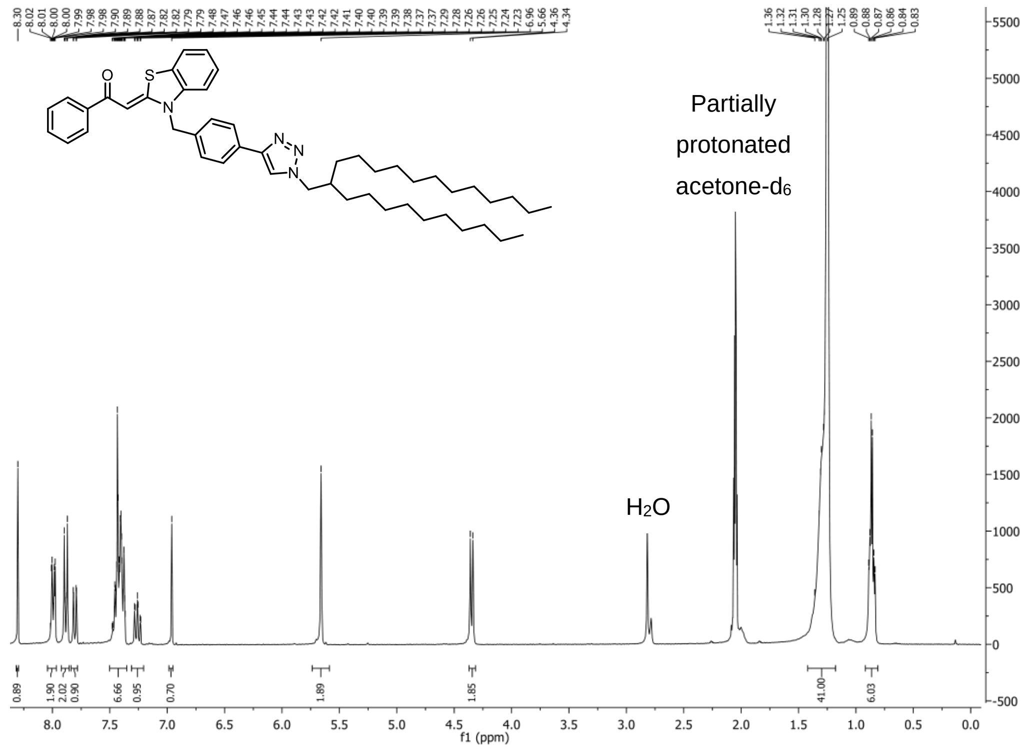

Figure S59. ^{1}H NMR-Spektrum-(*Z*)-2-(3-(4-(1-(2-decyltetradecyl)-1*H*-1,2,3-triazol-4-yl)benzyl)benzo[*d*]thiazol-2(3*H*)-ylidene)-1-phenylethan-1-one (**6a**) (acetone-d6, 300 MHz, 298 K).

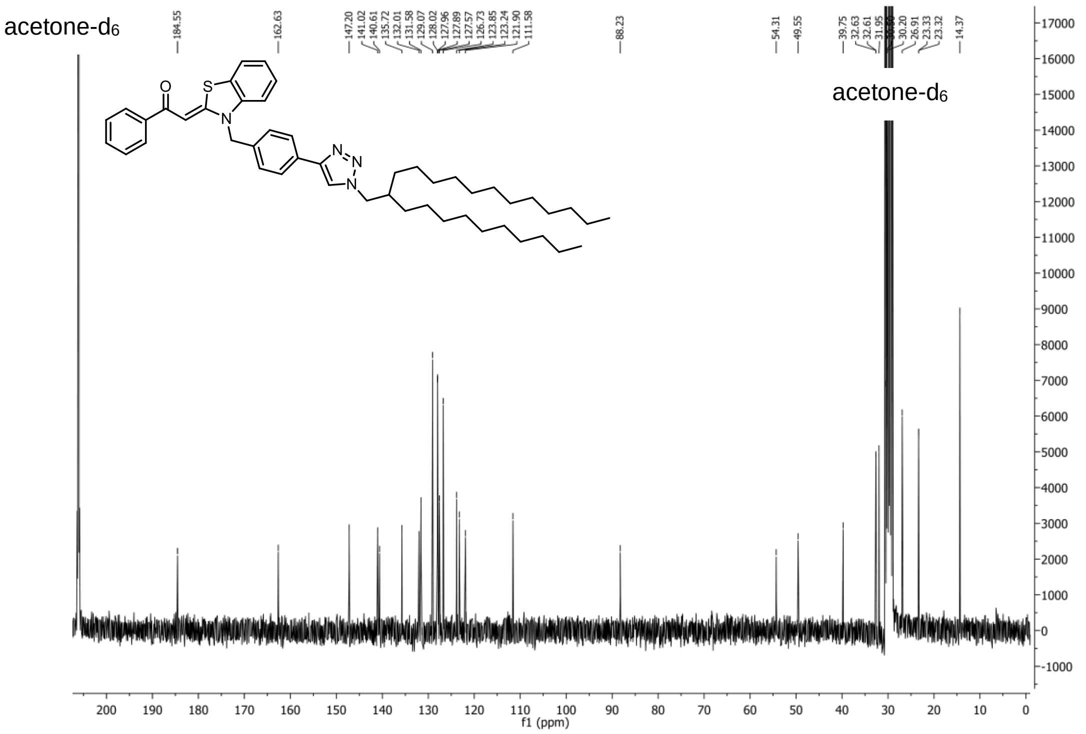

Figure S60. ^{13}C NMR spectrum (*Z*)-2-(3-(4-(1-(2-decyltetradecyl)-1*H*-1,2,3-triazol-4-yl)benzyl)benzo[*d*]thiazol-2(3*H*)-ylidene)-1-phenylethan-1-one (**6a**) (acetone-d6, 75 MHz, 298 K).

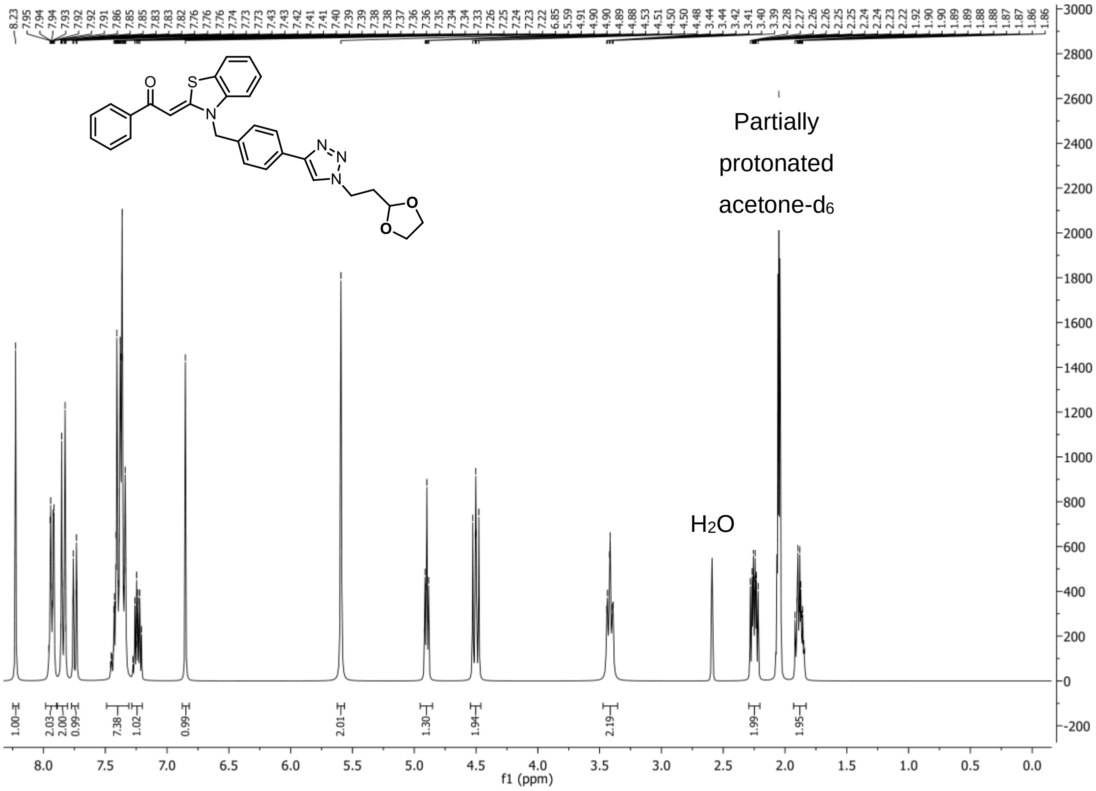

Figure S61. ^{1}H NMR spectrum (*Z*)-2-(3-(4-(1-(2-(1,3-dioxolan-2-yl)ethyl)-1*H*-1,2,3-triazol-4-yl)benzyl)benzo[*d*]thiazol-2(3*H*)-ylidene)-1-phenylethan-1-one (**6b**) (acetone-d_6/CS_2 5:1, 300 MHz, 298 K).

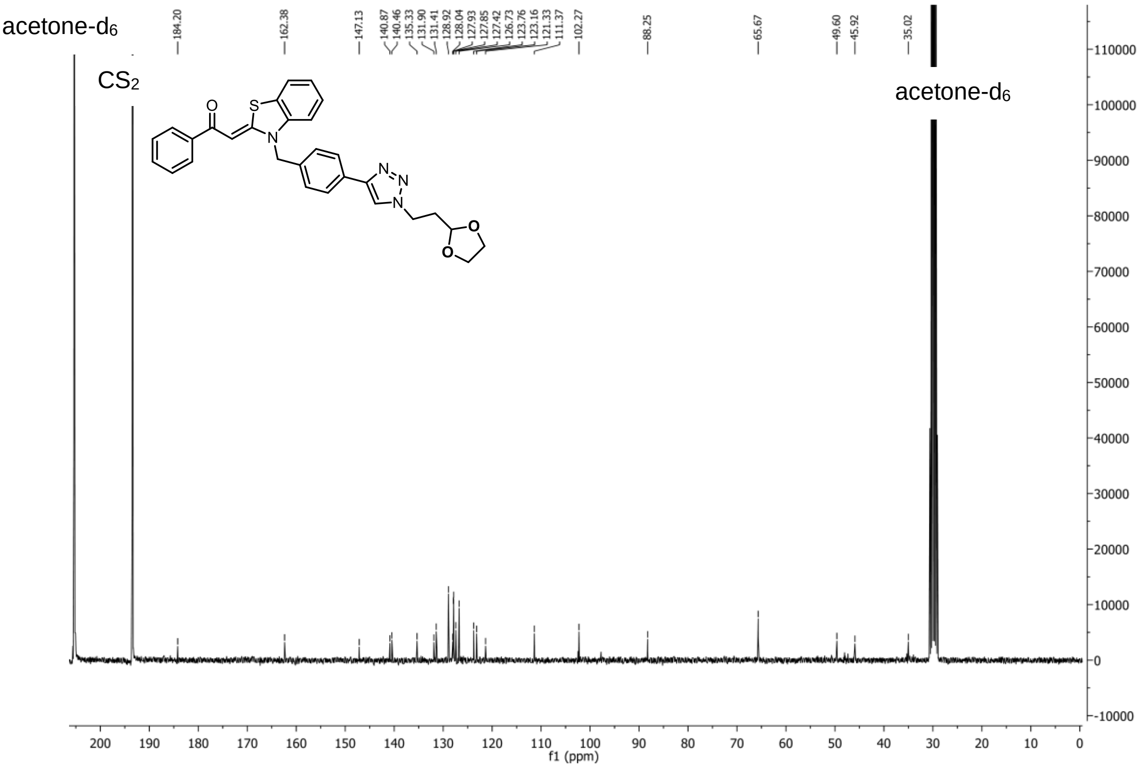

Figure S62. ^{13}C NMR spectrum (*Z*)-2-(3-(4-(1-(2-(1,3-dioxolan-2-yl)ethyl)-1*H*-1,2,3-triazol-4-yl)benzyl)benzo[*d*]thiazol-2(3*H*)-ylidene)-1-phenylethan-1-one (**6b**) (acetone-d_6/CS_2 5:1, 75 MHz, 298 K).

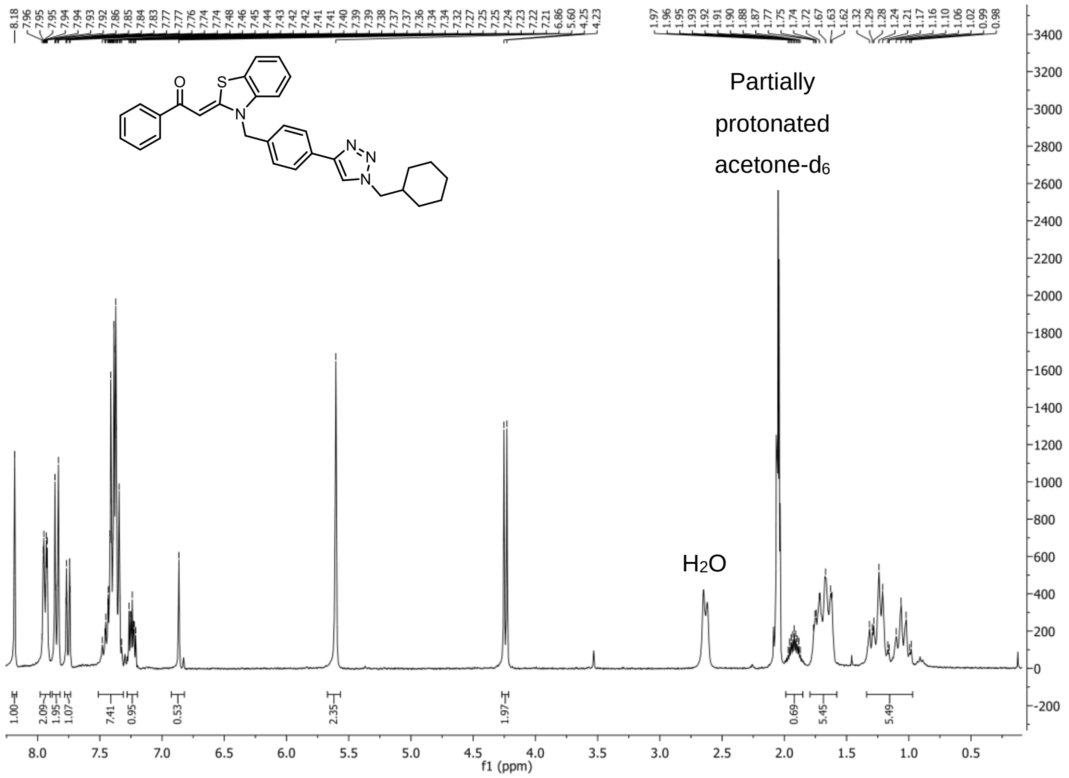

Figure S63. ^{1}H NMR spectrum (*Z*)-2-(3-(4-(1-(cyclohexylmethyl)-1*H*-1,2,3-triazol-4-yl)benzyl)benzo[*d*]thiazol-2(3*H*)-ylidene)-1-phenylethan-1-one (**6c**) (acetone-d_6/CS_2 5:1, 300 MHz, 298 K).

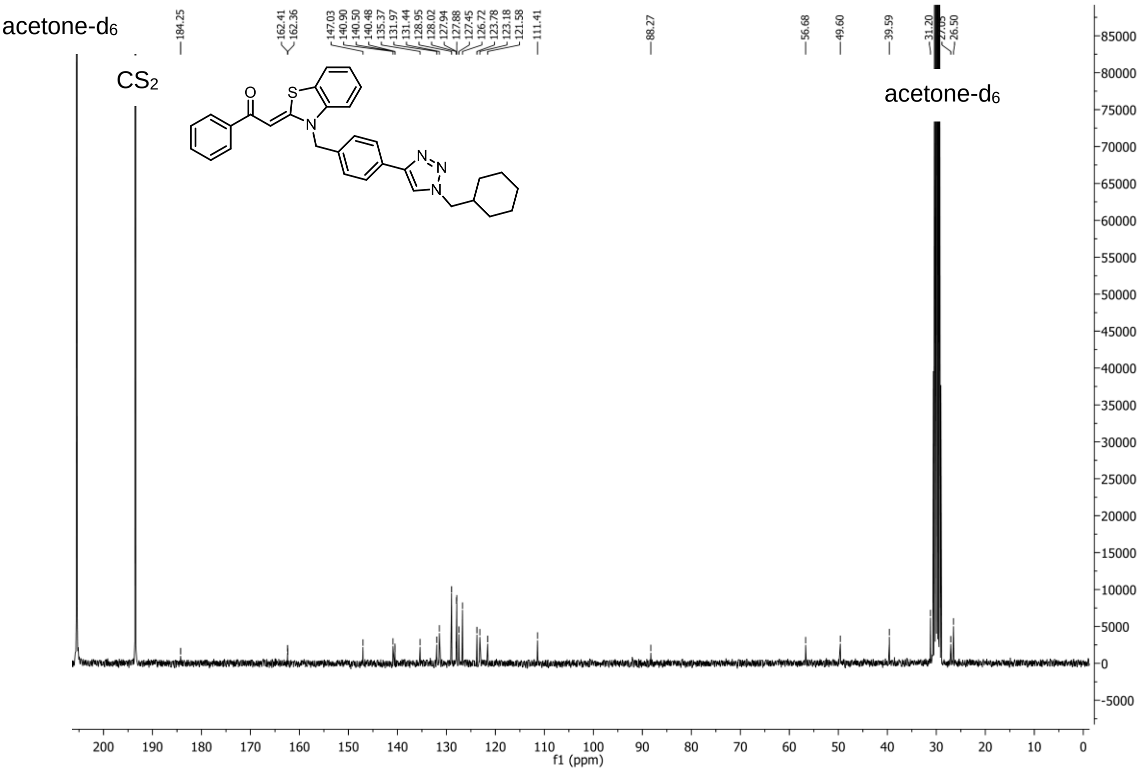

Figure S64. ^{13}C NMR spectrum (*Z*)-2-(3-(4-(1-(cyclohexylmethyl)-1*H*-1,2,3-triazol-4-yl)benzyl)benzo[*d*]thiazol-2(3*H*)-ylidene)-1-phenylethan-1-one (**6c**) (acetone-d_6/CS_2 5:1, 75 MHz, 298 K).

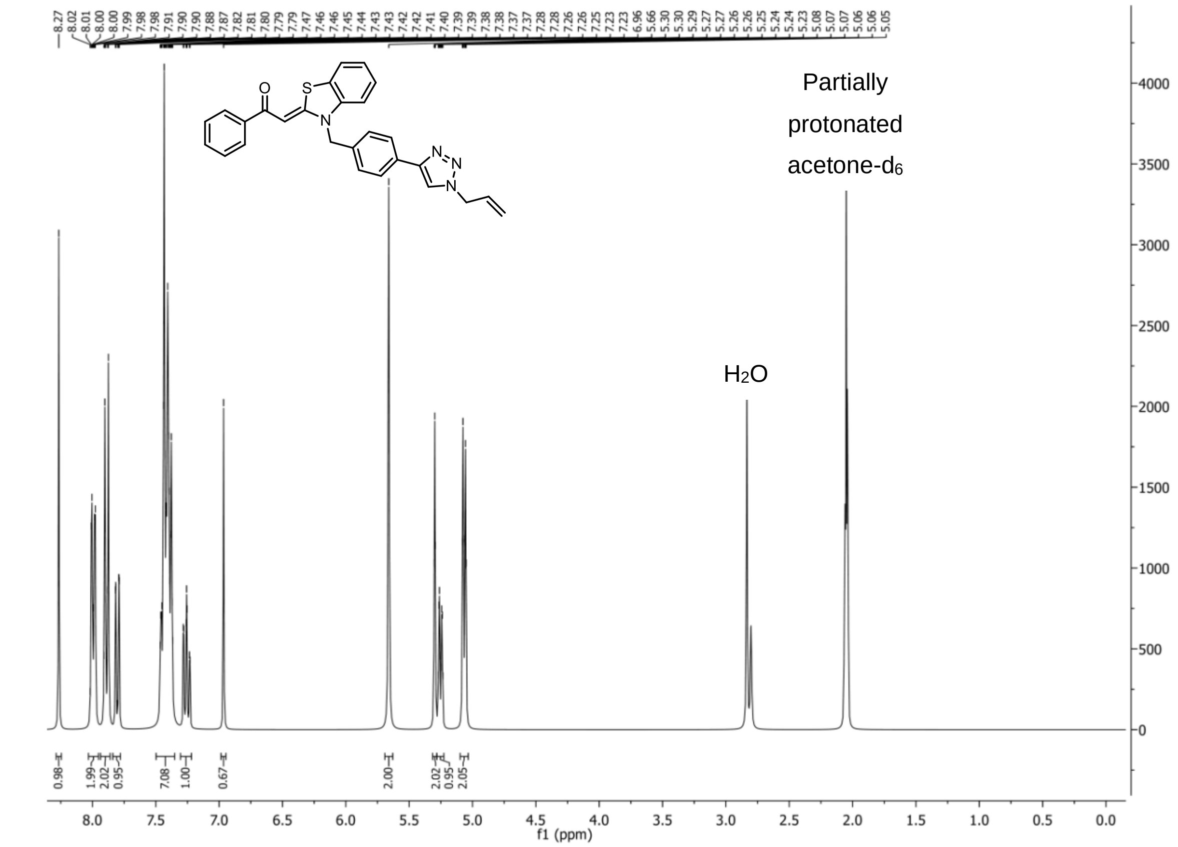

Figure S65. ^{1}H NMR spectrum (*Z*)-2-(3-(4-(1-allyl-1*H*-1,2,3-triazol-4-yl)benzyl)benzo[*d*]thiazol-2(3*H*)-ylidene)-1-phenylethan-1-one (**6d**) (acetone-d$_6$, 300 MHz, 298 K).

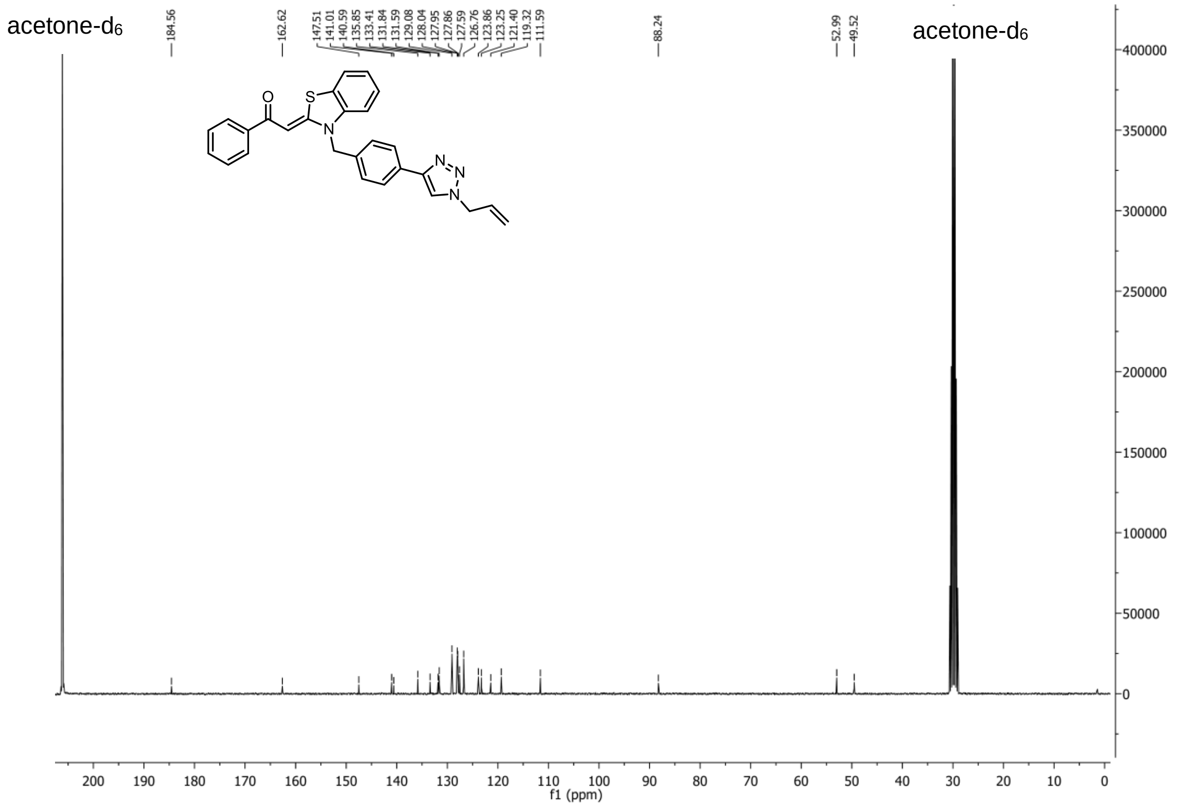

Figure S66. ^{13}C NMR spectrum (*Z*)-2-(3-(4-(1-allyl-1*H*-1,2,3-triazol-4-yl)benzyl)benzo[*d*]thiazol-2(3*H*)-ylidene)-1-phenylethan-1-one (**6d**) (acetone-d$_6$, 75 MHz, 298 K).

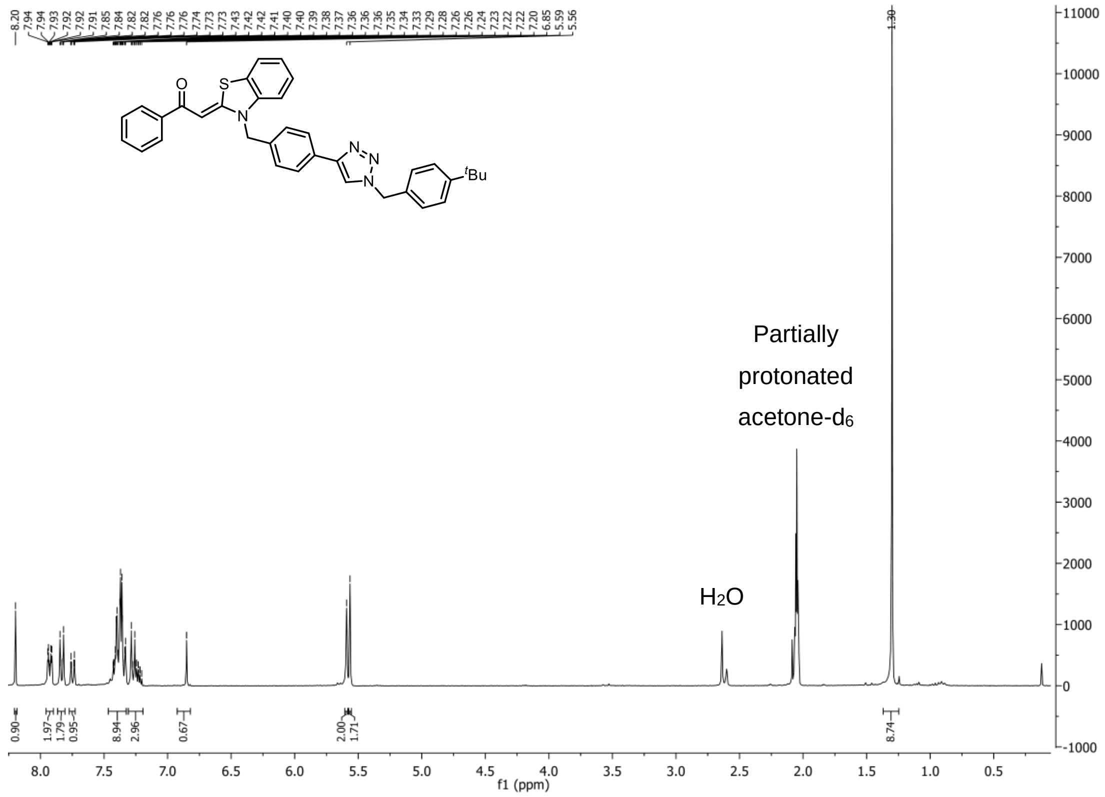

Figure S67. ^{1}H NMR spectrum (*Z*)-2-(3-(4-(1-(4-(*tert*-butyl)benzyl)-1*H*-1,2,3-triazol-4-yl)benzyl)benzo[*d*]thiazol-2(3*H*)-ylidene)-1-phenylethan-1-one (**6e**) (acetone-d$_6$/CS_2 5:1, 300 MHz, 298 K).

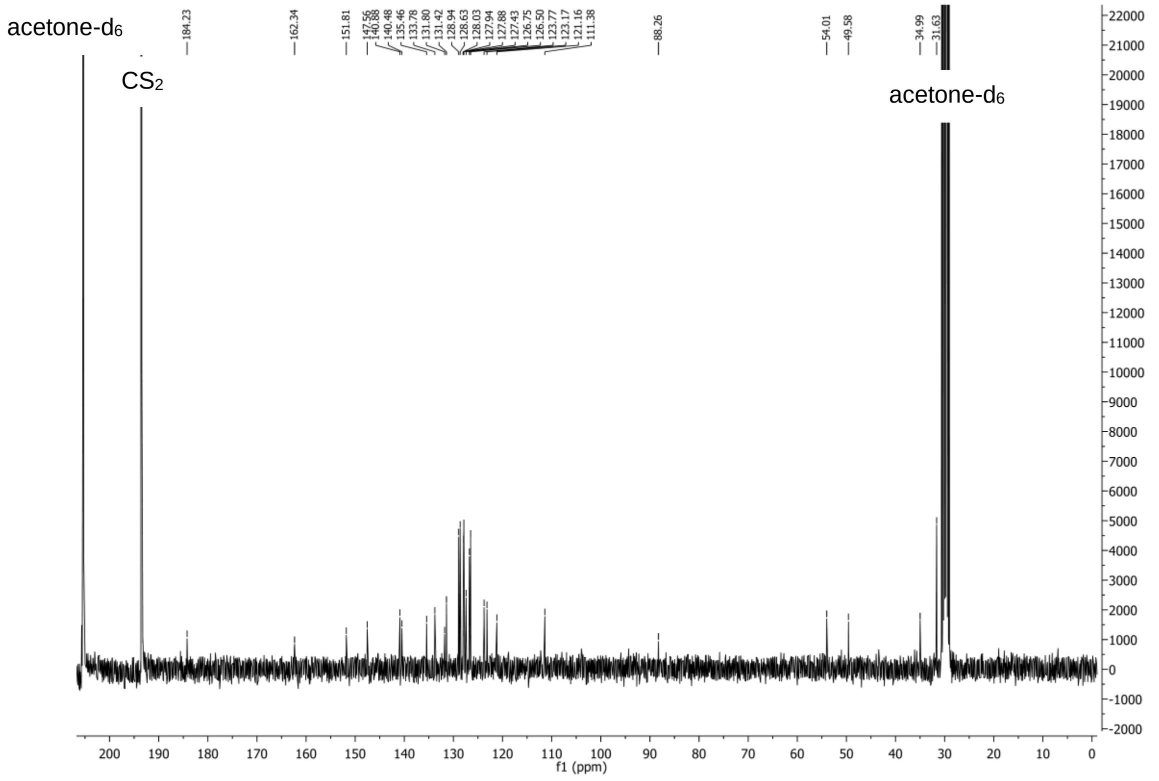

Figure S68. ^{13}C NMR spectrum (*Z*)-2-(3-(4-(1-(4-(*tert*-butyl)benzyl)-1*H*-1,2,3-triazol-4-yl)benzyl)benzo[*d*]thiazol-2(3*H*)-ylidene)-1-phenylethan-1-one (**6e**) (acetone-d$_6$/CS_2 5:1, 75 MHz, 298 K).

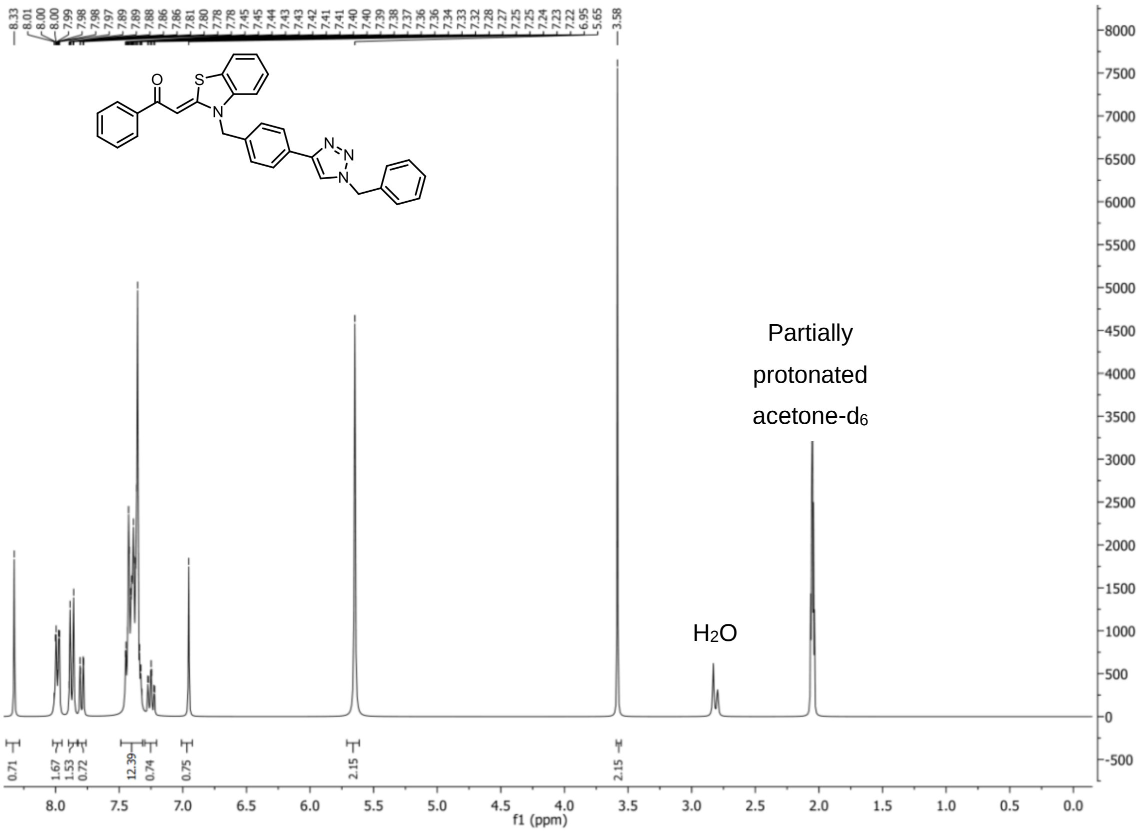

Figure S69. ^{1}H NMR spectrum (*Z*)-2-(3-(4-(1-benzyl-1*H*-1,2,3-triazol-4-yl)benzyl)benzo[*d*]thiazol-2(3*H*)-ylidene)-1-phenylethan-1-one (**6f**) (acetone-d$_6$, 300 MHz, 298 K).

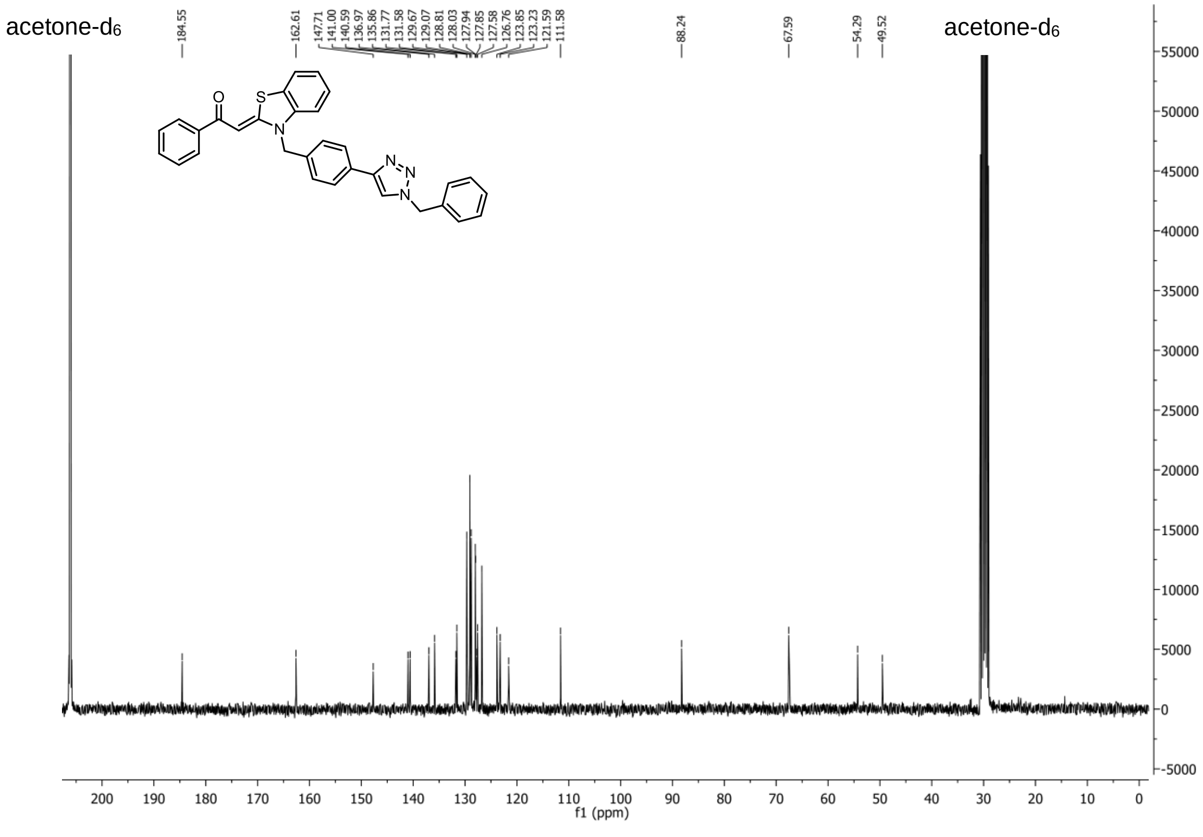

Figure S70. ^{13}C NMR spectrum (*Z*)-2-(3-(4-(1-benzyl-1*H*-1,2,3-triazol-4-yl)benzyl)benzo[*d*]thiazol-2(3*H*)-ylidene)-1-phenylethan-1-one (**6f**) (acetone-d$_6$, 75 MHz, 298 K).

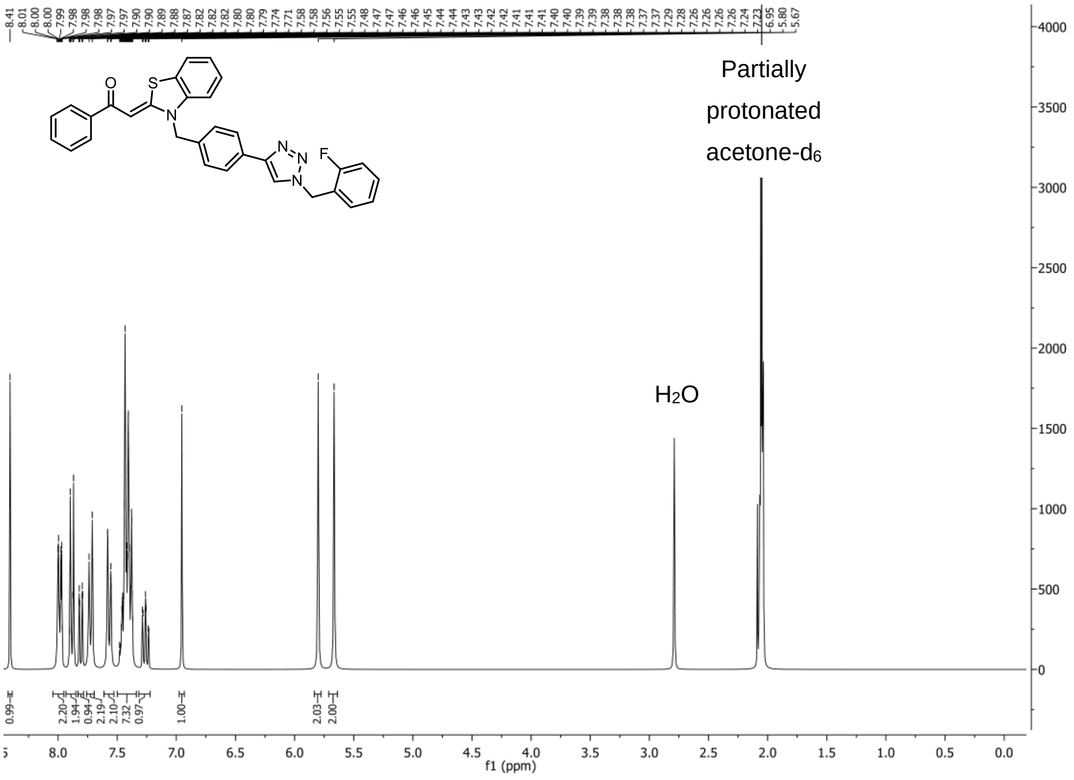

Figure S71. ^{1}H NMR spectrum (*Z*)-2-(3-(4-(1-(2-fluorobenzyl)-1*H*-1,2,3-triazol-4-yl)benzyl)benzo[*d*]thiazol-2(3*H*)-ylidene)-1-phenylethan-1-one (**6g**) (acetone-d$_6$, 300 MHz, 298 K).

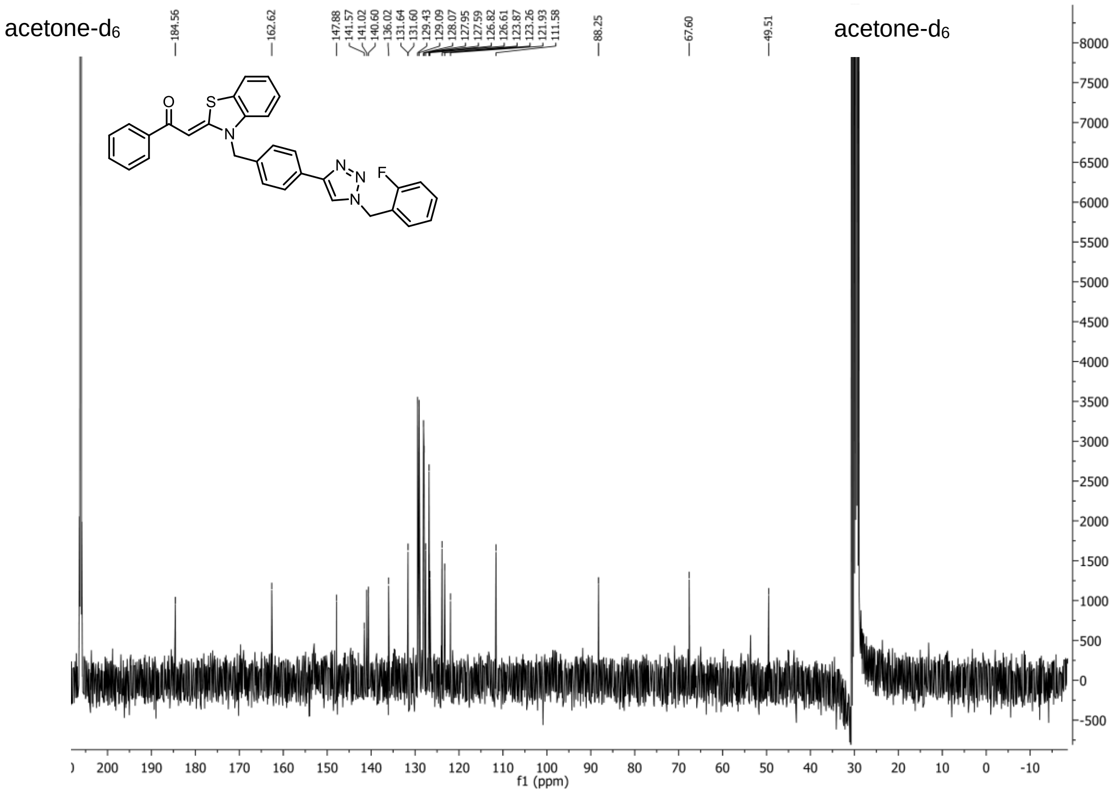

Figure S72. ^{13}C NMR spectrum (*Z*)-2-(3-(4-(1-(2-fluorobenzyl)-1*H*-1,2,3-triazol-4-yl)benzyl)benzo[*d*]thiazol-2(3*H*)-ylidene)-1-phenylethan-1-one (**6g**) (acetone-d$_6$, 75 MHz, 298 K).

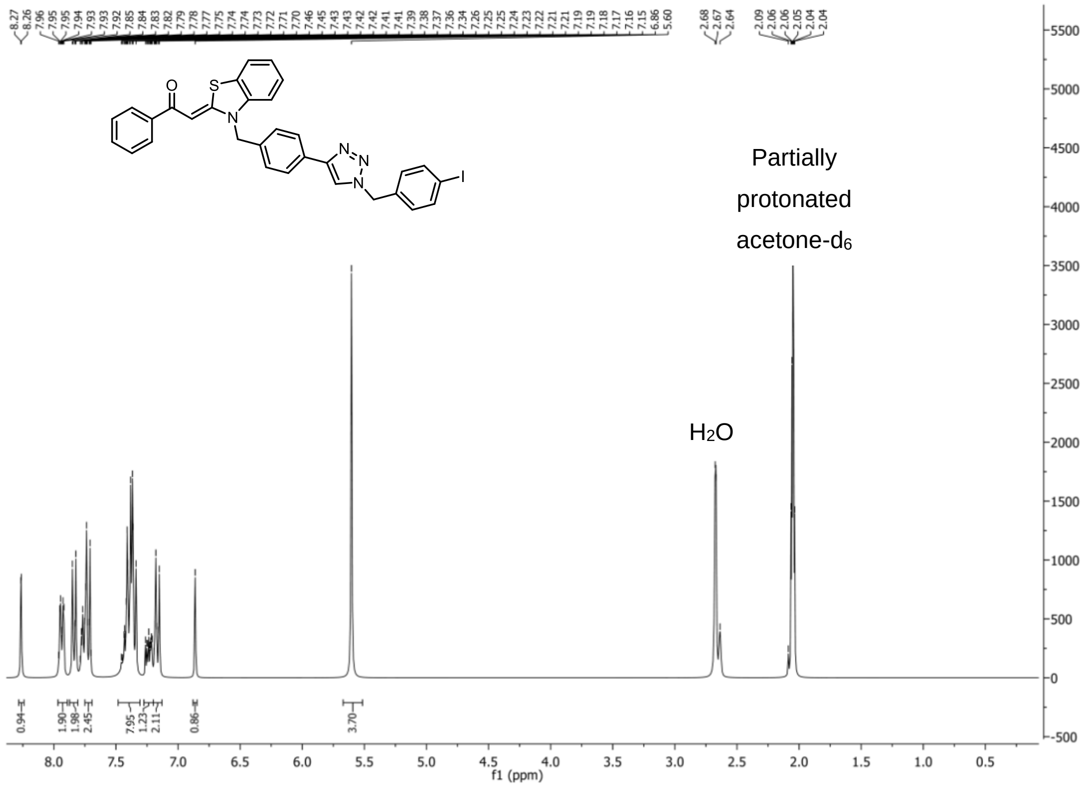

Figure S73. ^{1}H NMR spectrum (*Z*)-2-(3-(4-(1-(4-iodobenzyl)-1*H*-1,2,3-triazol-4-yl)benzyl)benzo[*d*]thiazol-2(3*H*)-ylidene)-1-phenylethan-1-one (**6h**) (acetone-d$_6$/CS_2 5:1, 300 MHz, 298 K).

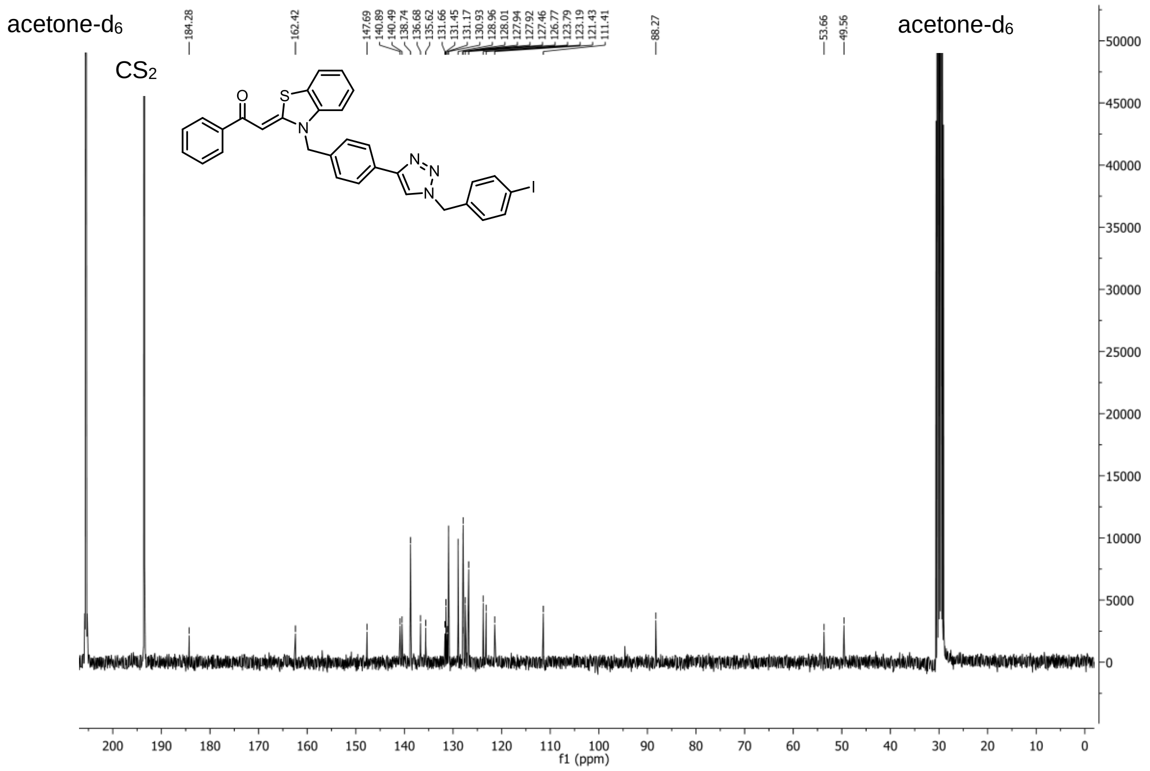

Figure S74. ^{13}C NMR spectrum (*Z*)-2-(3-(4-(1-(4-iodobenzyl)-1*H*-1,2,3-triazol-4-yl)benzyl)benzo[*d*]thiazol-2(3*H*)-ylidene)-1-phenylethan-1-one (**6h**) (acetone-d$_6$/CS_2 5:1, 75 MHz, 298 K).

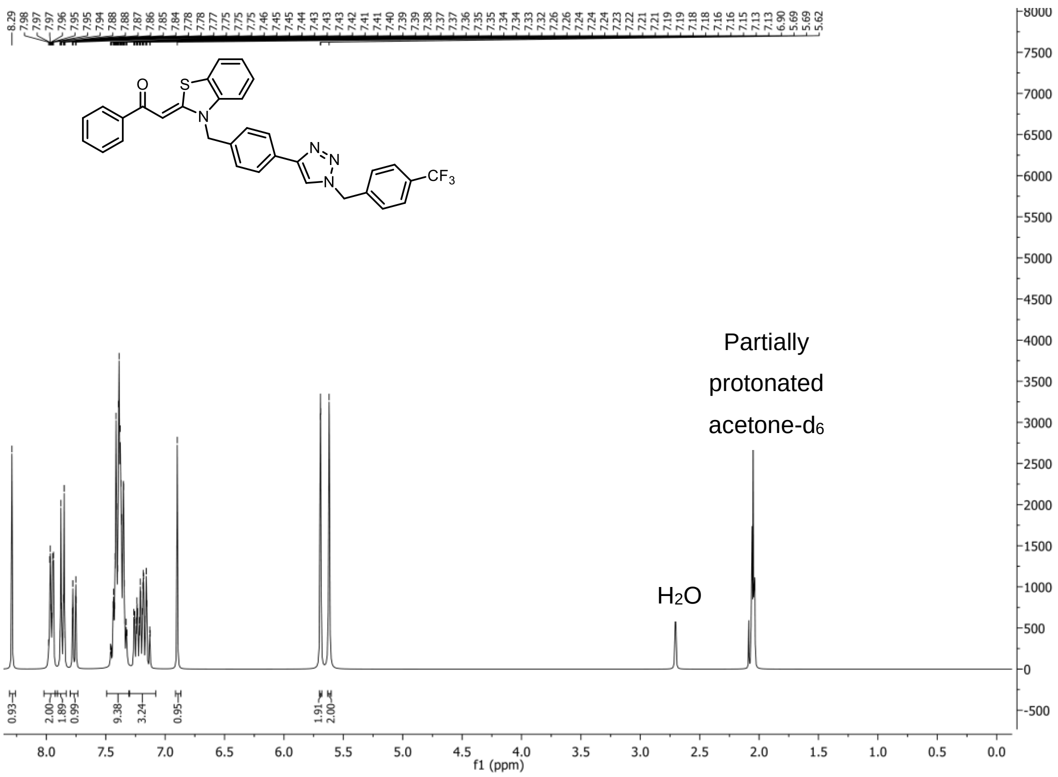

Figure S75. ^{1}H NMR spectrum (*Z*)-2-(3-(4-(1-(4-trifluoromethylbenzyl)-1*H*-1,2,3-triazol-4-yl)benzyl)benzo[*d*]thiazol-2(3*H*)-ylidene)-1-phenylethan-1-one (**6i**) (acetone-d_6/CS_2 5:1, 300 MHz, 298 K).

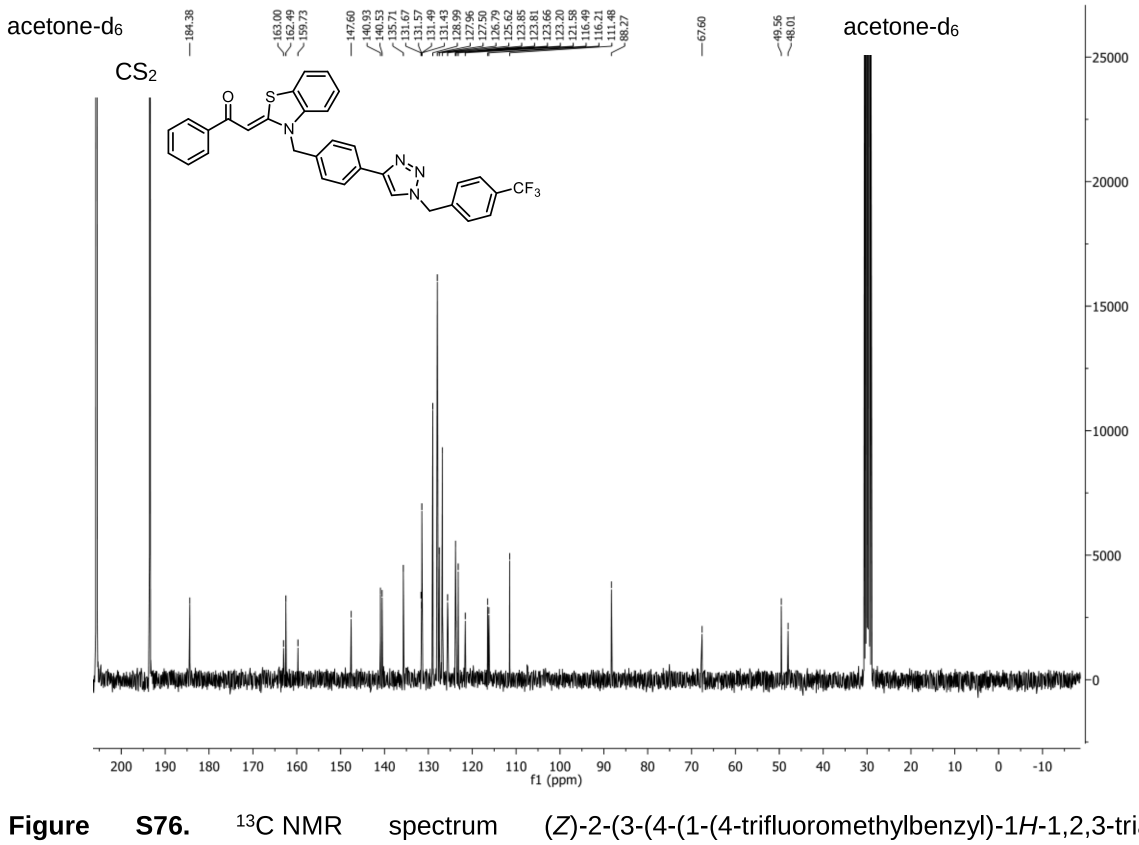

Figure S76. ^{13}C NMR spectrum (*Z*)-2-(3-(4-(1-(4-trifluoromethylbenzyl)-1*H*-1,2,3-triazol-4-yl)benzyl)benzo[*d*]thiazol-2(3*H*)-ylidene)-1-phenylethan-1-one (**6i**) (acetone-d_6/CS_2 5:1, 75 MHz, 298 K).

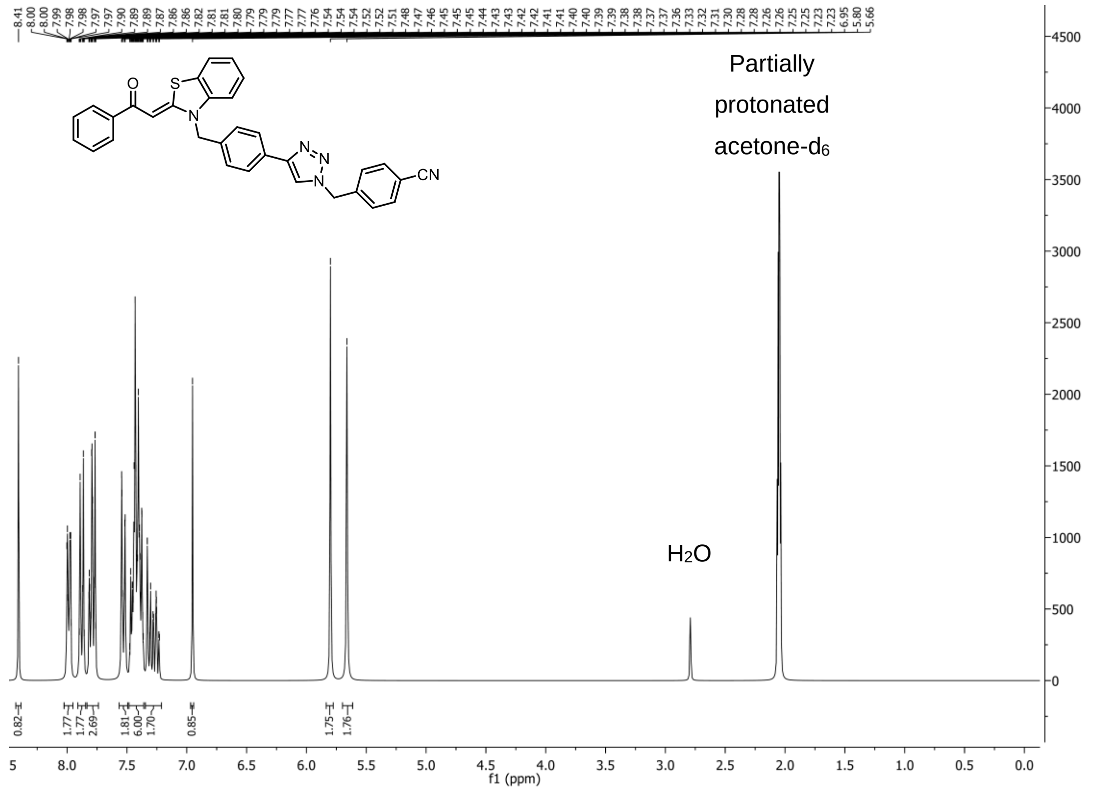

Figure S77. ^{1}H NMR spectrum (*Z*)-4-((4-(4-((2-(2-oxo-2-phenylethylidene)benzo[*d*]thiazol-3(2*H*)-yl)methyl)phenyl)-1*H*-1,2,3-triazol-1-yl)methyl)benzonitrile (**6j**) (acetone-d$_6$, 300 MHz, 298 K).

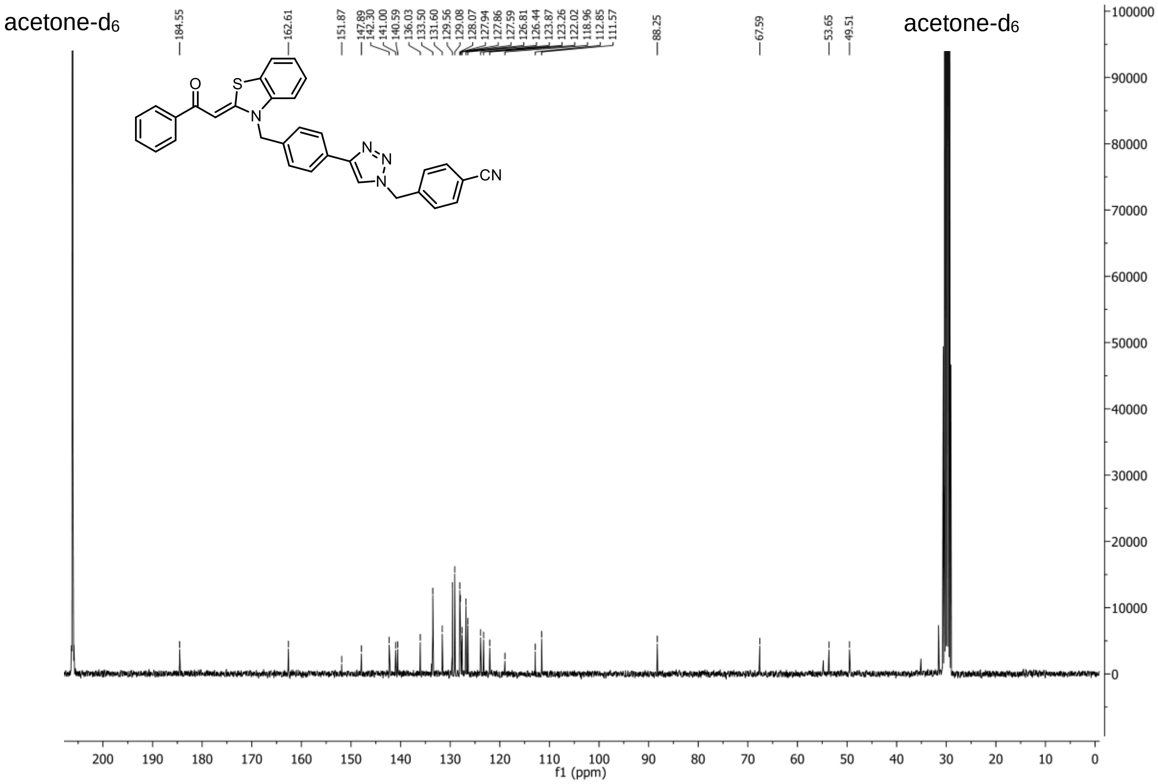

Figure S78. ^{13}C NMR spectrum (*Z*)-4-((4-(4-((2-(2-oxo-2-phenylethylidene)benzo[*d*]thiazol-3(2*H*)-yl)methyl)phenyl)-1*H*-1,2,3-triazol-1-yl)methyl)benzonitrile (**6j**) (acetone-d$_6$, 75 MHz, 298 K).

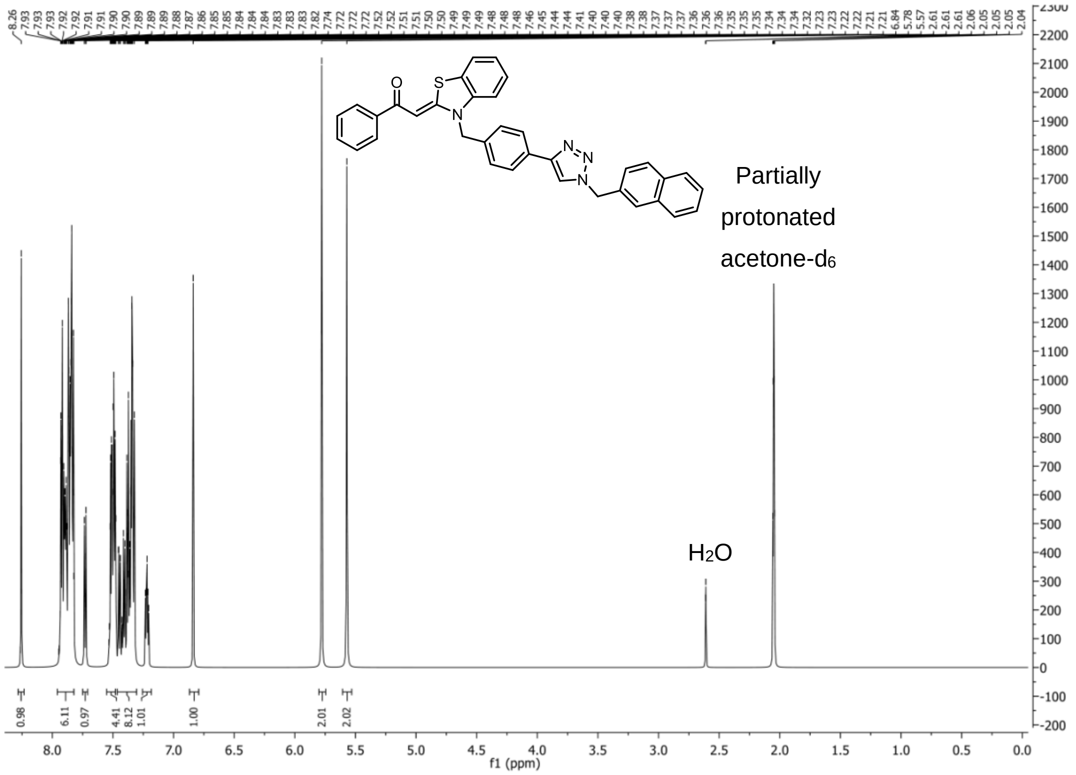

Figure S79. ^{1}H NMR spectrum (*Z*)-2-(3-(4-(1-(naphthalene-2-ylmethyl)-1*H*-1,2,3-triazol-4-yl)benzyl)benzo[*d*]thiazol-2(3*H*)-ylidene)-1-phenylethan-1-one (**6k**) (acetone-d6/CS_2 5:1, 600 MHz, 298 K).

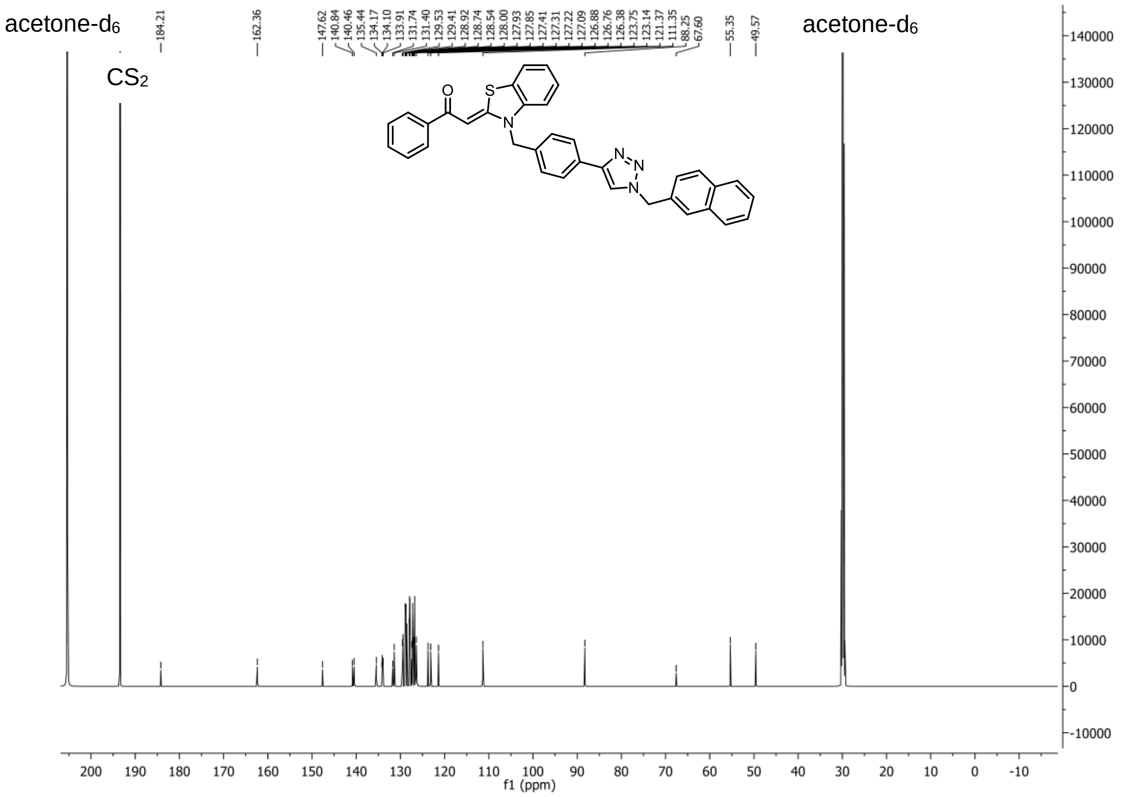

Figure S80. ^{13}C NMR spectrum (*Z*)-2-(3-(4-(1-(naphthalene-2-ylmethyl)-1*H*-1,2,3-triazol-4-yl)benzyl)benzo[*d*]thiazol-2(3*H*)-ylidene)-1-phenylethan-1-one (**6k**) (acetone-d6/CS_2 5:1, 150 MHz, 298 K).

7 Overview of photophysical properties of alkynylated aroyl-*S,N*-ketene acetals 5 and 6

All solution spectra were recorded in ethanol or in ethanol/water mixtures at $T = 298$ K, the excitation wavelengths λ_{exc} for the AIE-titration studies and the emission spectra in solution were determined from the absorption maxima λ_{max} of this compound, the excitation wavelength for the solid-state emission spectra was determined from solid-state excitation spectra. The dye concentration of the solution for absorption measurements was $c = 10^{-5}$ M and the dye concentration of the ethanol/water mixtures for AIE measurements was $c = 10^{-7}$ M. All alkynylated derivatives **3** and **4** fluoresce in ethanol blue ($\lambda_{max(em.)}$ = 447 - 454 nm) with mostly very small quantum yields ($\Phi_f < 0.01$).

Table S10. Photophysical properties of alkynylated aroyl-*S,N*-ketene acetal **3** and **4**.

entry	compound	$\lambda_{max(abs.)}$[a] (ε [L·mol^{-1}·cm^{-1}])	$\lambda_{max(em.)}$[b] solid state [nm] (Φ_f)	solid state picture[c]
1	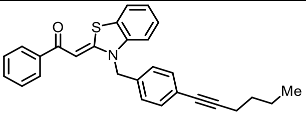 **3a**	256 (50800), 382 (37000)	510 (0.07)	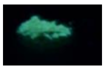
2	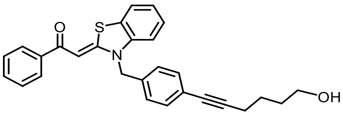 **3b**	256 (37500), 382 (35300)	525 (0.13)	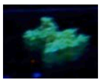
3	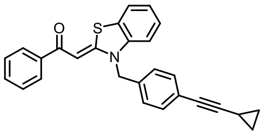 **3c**	257 (51100), 382 (37400)	500 (0.07)	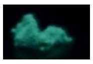
4	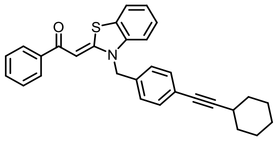 **3d**	258 (47700), 382 (35500)	500 (0.07)	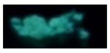
5	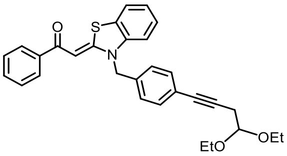 **3e**	382 (38000)	-	-

Table S10 continued.

entry	compound	$\lambda_{max(abs.)}$[a] (ε [$L \cdot mol^{-1} \cdot cm^{-1}$])	$\lambda_{max(em.)}$[b] solid state [nm] (Φ_f)	solid state picture[c]
6	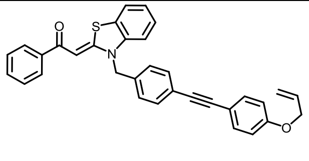 **3f**	293 (31000),<br>312 (25300)<br>382 (38900)	550<br>(0.05)	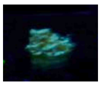
7	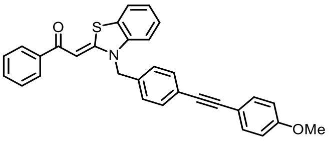 **3g**	255 (54800),<br>292 (48000),<br>311 (31300),<br>382 (50000)	500<br>(0.07)	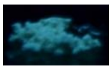
8	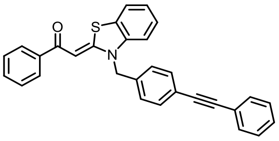 **3h**	269 (55100),<br>283 (47200),<br>302 (30100),<br>382 (45700)	498<br>(0.05)	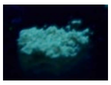
9	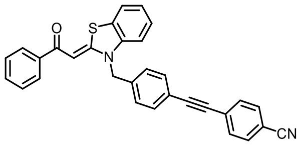 **3i**	258 (55500),<br>271 (47500<br>298 (30000)<br>316 (31100),<br>382 (34100)	520<br>(0.05)	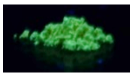
10	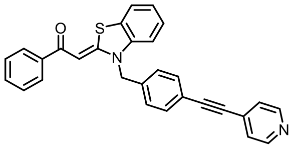 **3j**	286 (41900),<br>383 (32800)	470<br>(0.02)	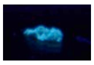
11	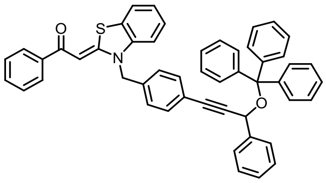 **3k**	383 (26100)	496<br>(0.03)	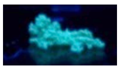
12	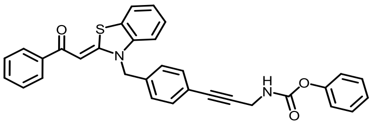 **3l**	256 (34000),<br>382 (29900)	500<br>(0.15)	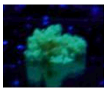

Table S10 continued.

entry	compound	$\lambda_{max(abs.)}$[a] (ε [L·mol^{-1}·cm^{-1}])	$\lambda_{max(em.)}$[b] solid state [nm] (Φ_f)	solid state picture[c]
13	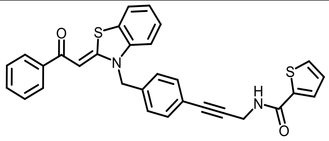 **3m**	382 (33400)	492 (0.19)	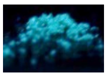
14	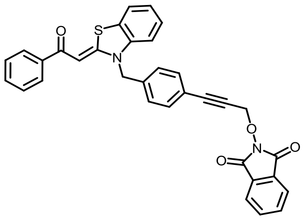 **3n**	382 (15600)	499 (0.04)	-
15	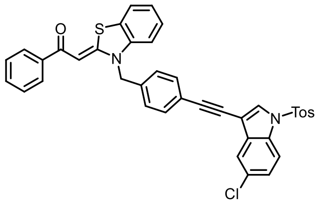 **3o**	308 (19800), 382 (15000)	546 (0.09)	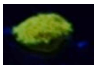
16	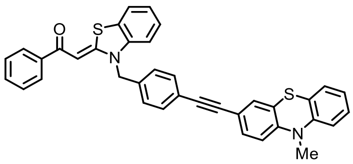 **3p**	272 (56100), 379 (40400)	524 (0.01)	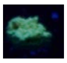
17	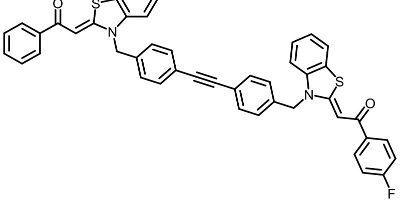 **3q**	288 (39000), 307 (30500), 382 (61500)	460 (0.24)	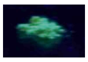
18	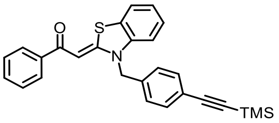 **3r**	263 (37700), 381 (35500)	465 (0.09)	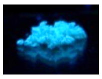
19	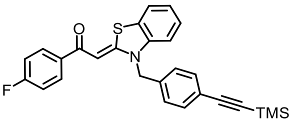 **3s**	253 (61400), 264 (65500), 381 (35100)	518 (0.09)	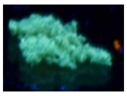

Table S10 continued.

entry	compound	$\lambda_{max(abs.)}$[a] (ε [L·mol^{-1}·cm^{-1}])	$\lambda_{max(em.)}$[b] solid state [nm] (Φ_f)	solid state picture[c]
20	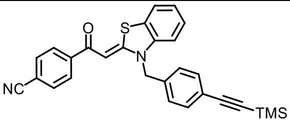 **3t**	262 (50300), 400 (38400)	500 (0.04)	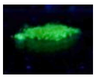
21	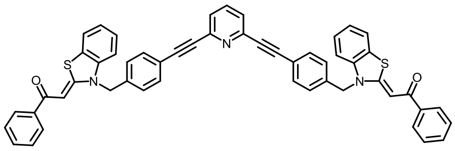 **3u**	271 (33200), 321 (21600), 381 (31500)	-	-
22	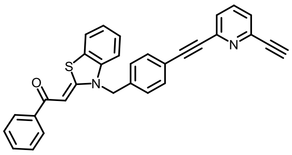 **3v**	284 (21600), 310 (17500), 382 (15700)	-	-
23	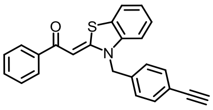 **4a**	251 (40400), 382 (41300)	508 (0.10)	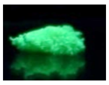
24	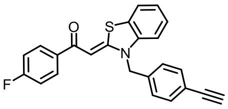 **4b**	263 (74800), 386 (76200)	520 (0.09)	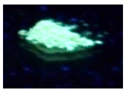
25	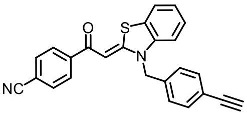 **4c**	401 (28300)	547 (0.08)	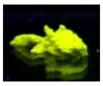

[a]: measured in ethanol, T = 298 K, c = 10^{-5} M, [b]: T = 298 K, λ_{exc} = $\lambda_{abs,max}$, [c]: pictures taken under UV-light (λ_{exc} = 365 nm).

8 Absorption and emission spectra

All solution spectra were recorded in ethanol or in ethanol/water mixtures at T = 298 K, the excitation wavelengths λ_{exc} for the AIE-titration studies and the emission spectra in solution were determined from the absorption maxima λ_{max} of this compound, the excitation wavelength for the solid-state emission spectra was determined from solid-state excitation spectra. The dye concentration of the solution for absorption measurements was *c*(**3**,**4**) = 10^{-5} M and the dye concentration of the ethanol/water mixtures for AIE measurements was *c*(**3**,**4**) = 10^{-7} M.

8.1 Absorption and emission spectra of alkynylated aroyl-*S,N*-ketene acetals 3 and 4

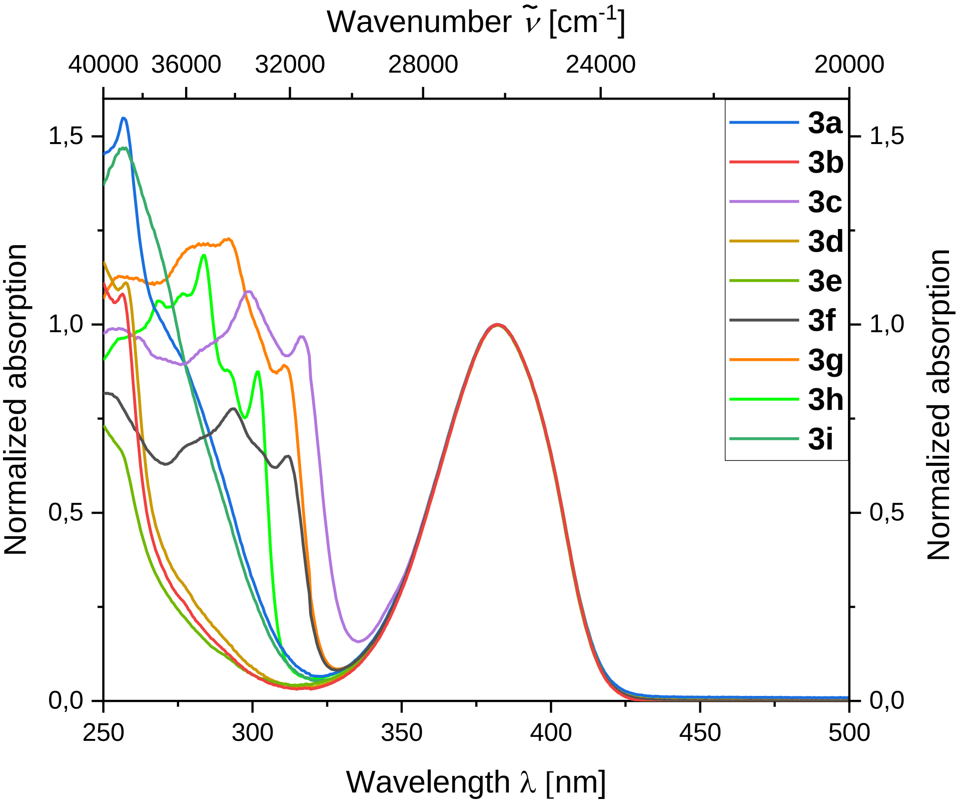

Figure S81. Selected, normalized UV/Vis absorption bands of alkynylated aroyl-*S,N*-ketene acetals **3**.

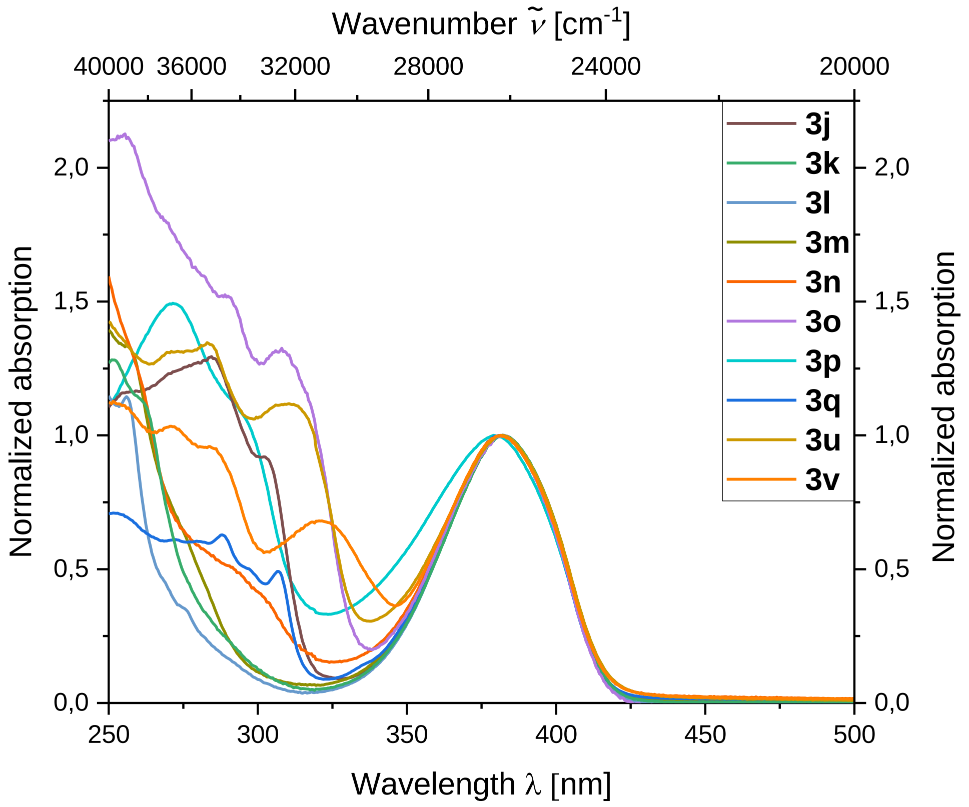

Figure S82. Selected, normalized UV/Vis absorption bands of alkynylated aroyl-*S,N*-ketene acetals **3**.

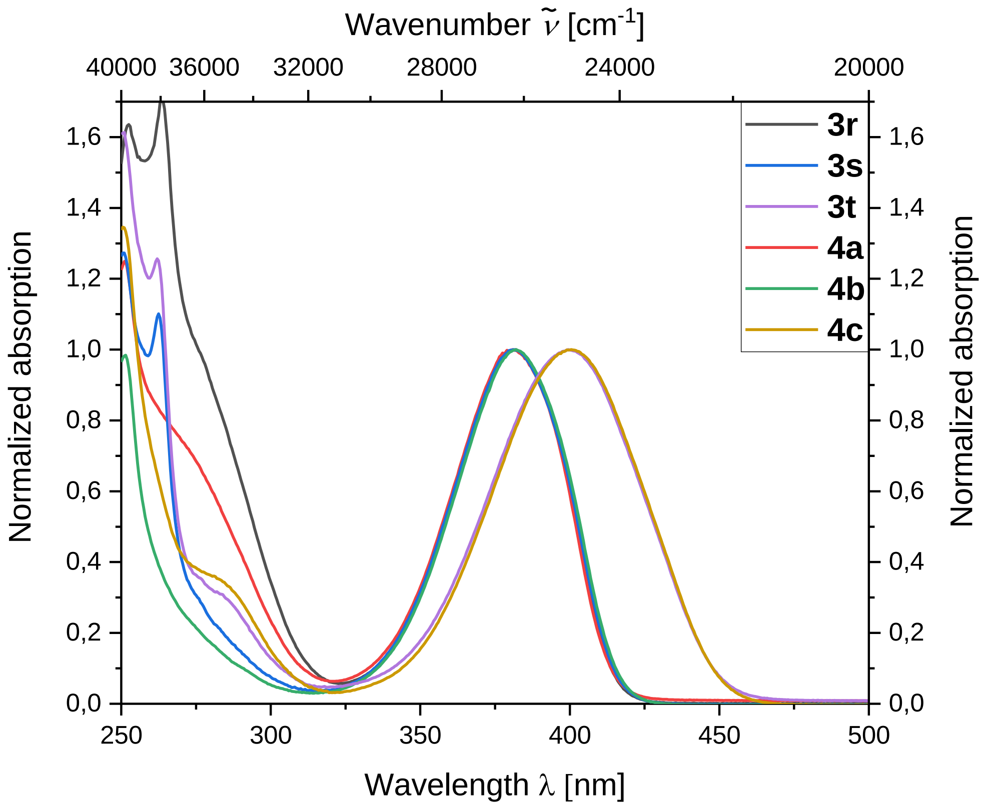

Figure S83. Selected, normalized UV/Vis absorption bands of alkynylated aroyl-*S,N*-ketene acetals **3** and **4**.

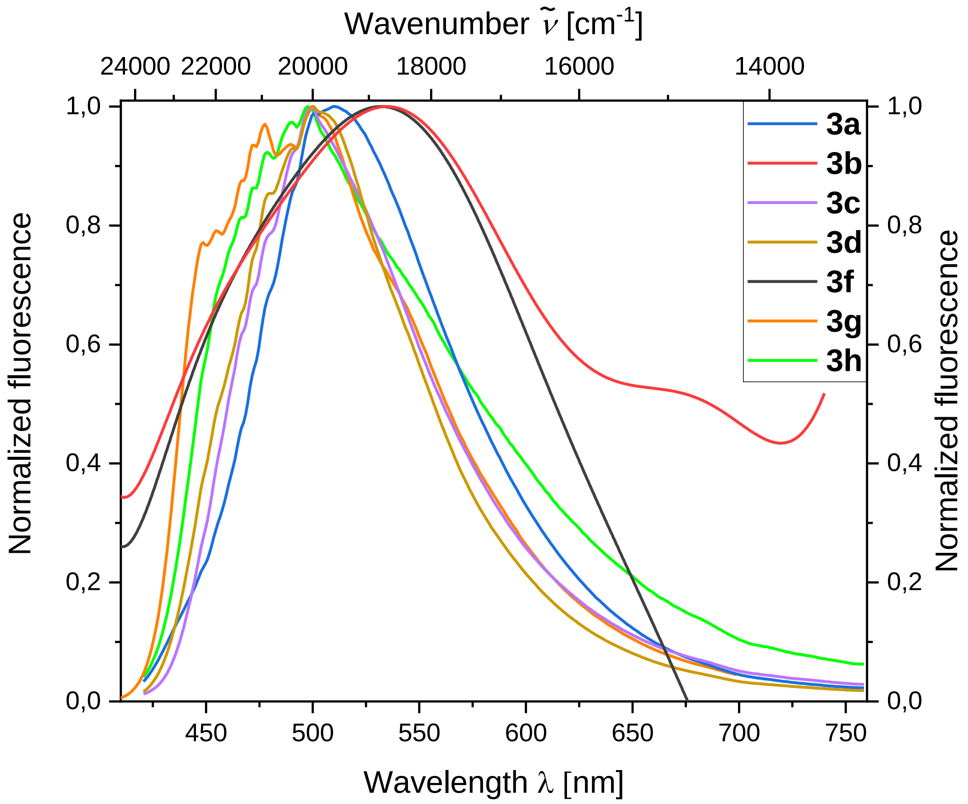

Figure S84. Normalized solid-state emission bands of alkynylated aroyl-*S,N*-ketene acetals **3**.

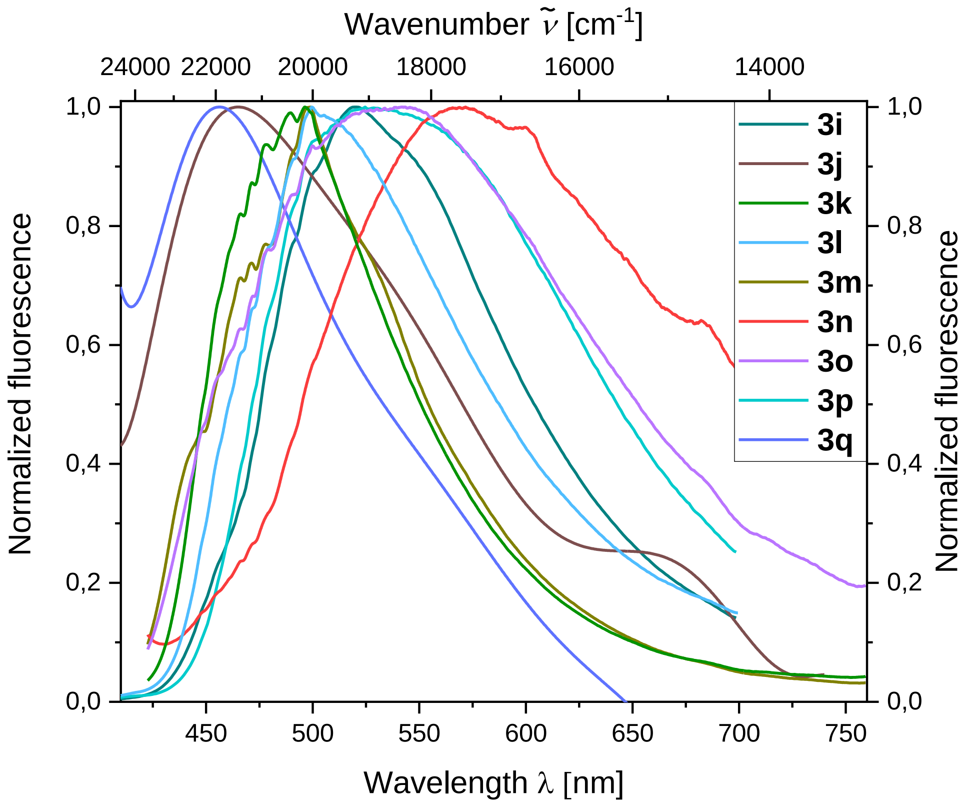

Figure S85. Normalized solid-state emission bands of alkynylated aroyl-*S,N*-ketene acetals **3**.

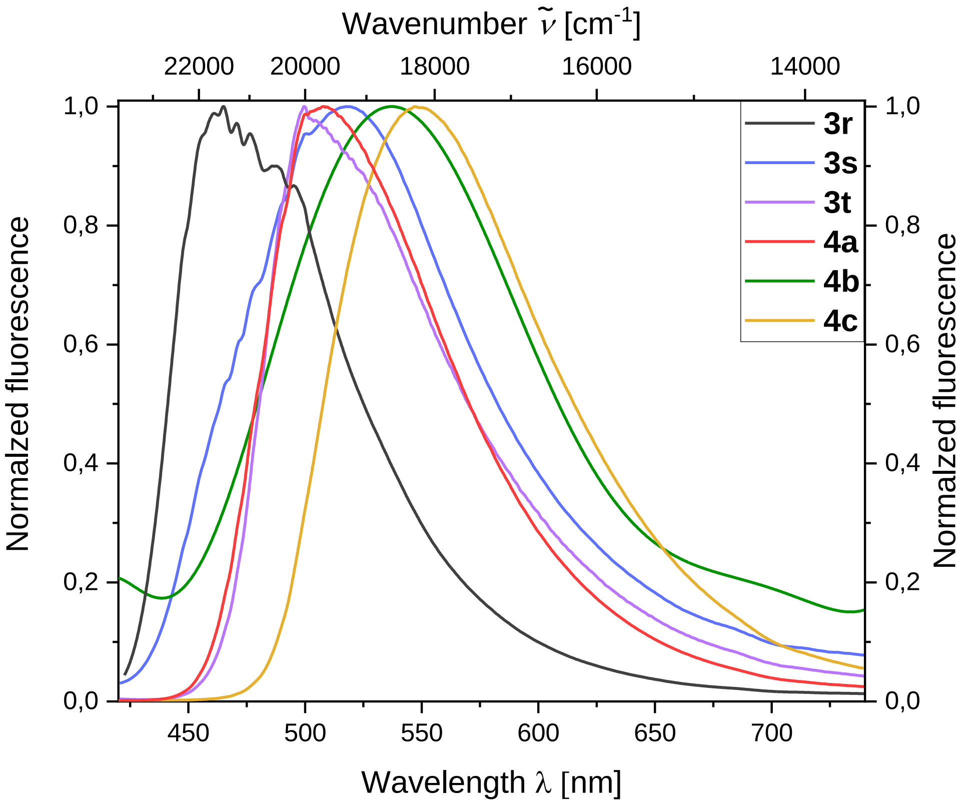

Figure S86. Normalized solid-state emission bands of alkynylated aroyl-*S,N*-ketene acetals **3** and **4**.

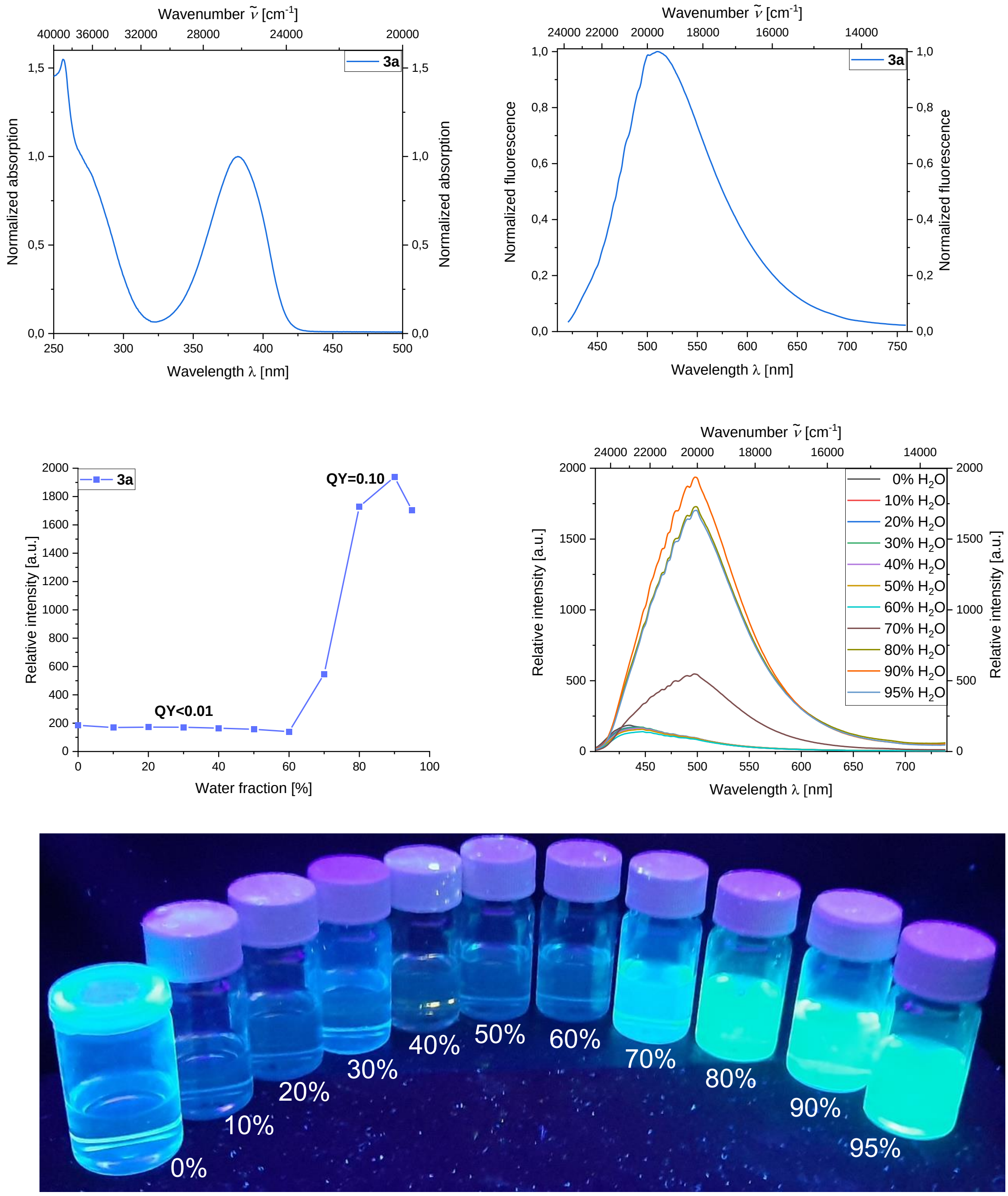

Figure S87. Absorption spectrum of **3a** in ethanol (top, left), solid-state emission spectrum (top, right), and AIE-induced changes in emission (center, left), AIE-related emission spectra of compound **3a** (center, right) and photographs of solutions of dye **3a** in ethanol/water mixtures of increasing water content (bottom). The latter spectra were measured in ethanol/water mixtures of varying water content.

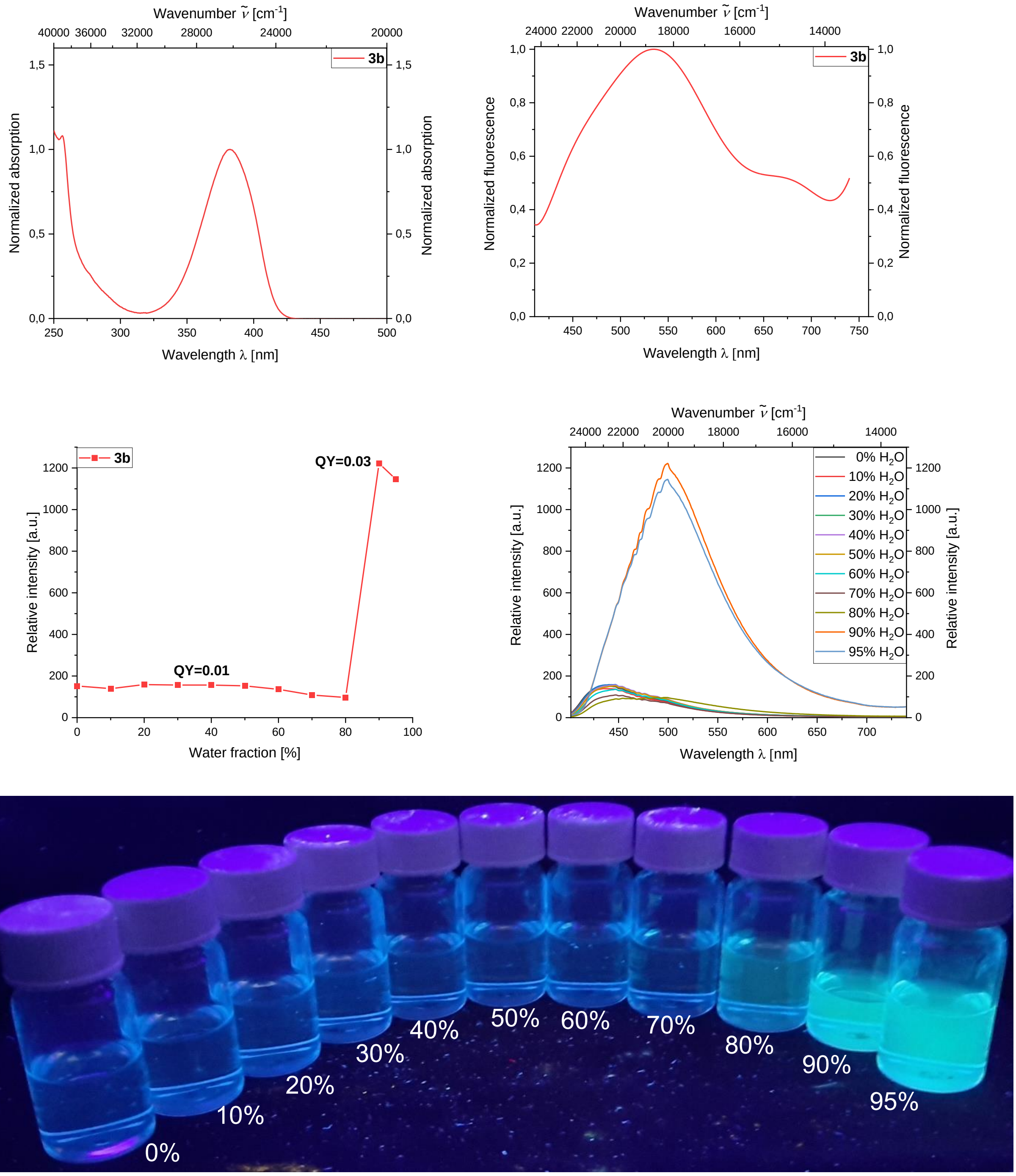

Figure S88. Absorption spectrum of **3b** in ethanol (top, left), solid-state emission spectrum (top, right), and AIE-induced changes in emission (center, left), AIE-related emission spectra of compound **3b** (center, right) and photographs of solutions of dye **3b** in ethanol/water mixtures of increasing water content (bottom). The latter spectra were measured in ethanol/water mixtures of varying water content.

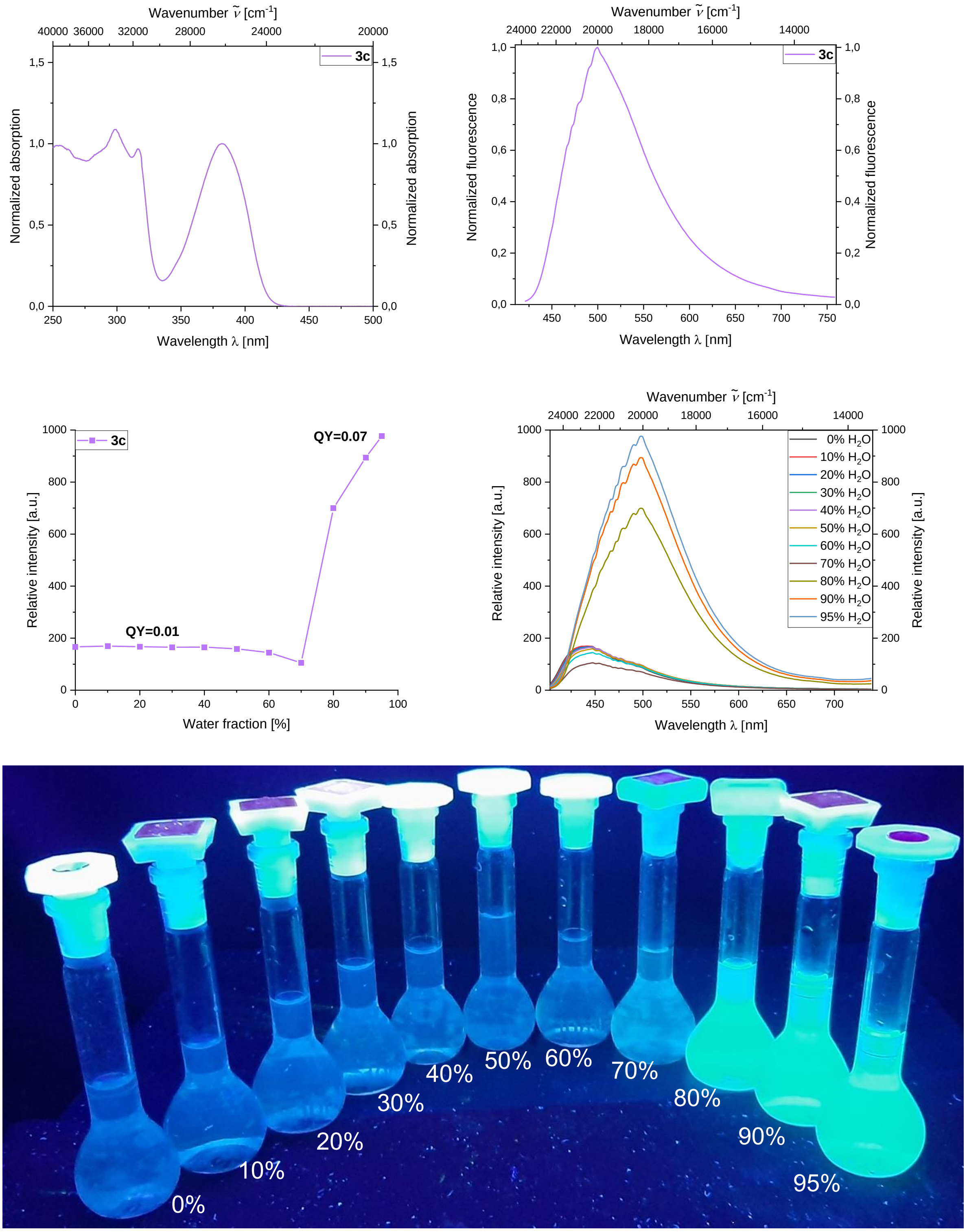

Figure S89. Absorption spectrum of **3c** in ethanol (top, left), solid-state emission spectrum (top, right), and AIE-induced changes in emission (center, left), AIE-related emission spectra of compound **3c** (center, right) and photographs of solutions of dye **3c** in ethanol/water mixtures of increasing water content (bottom). The latter spectra were measured in ethanol/water mixtures of varying water content.

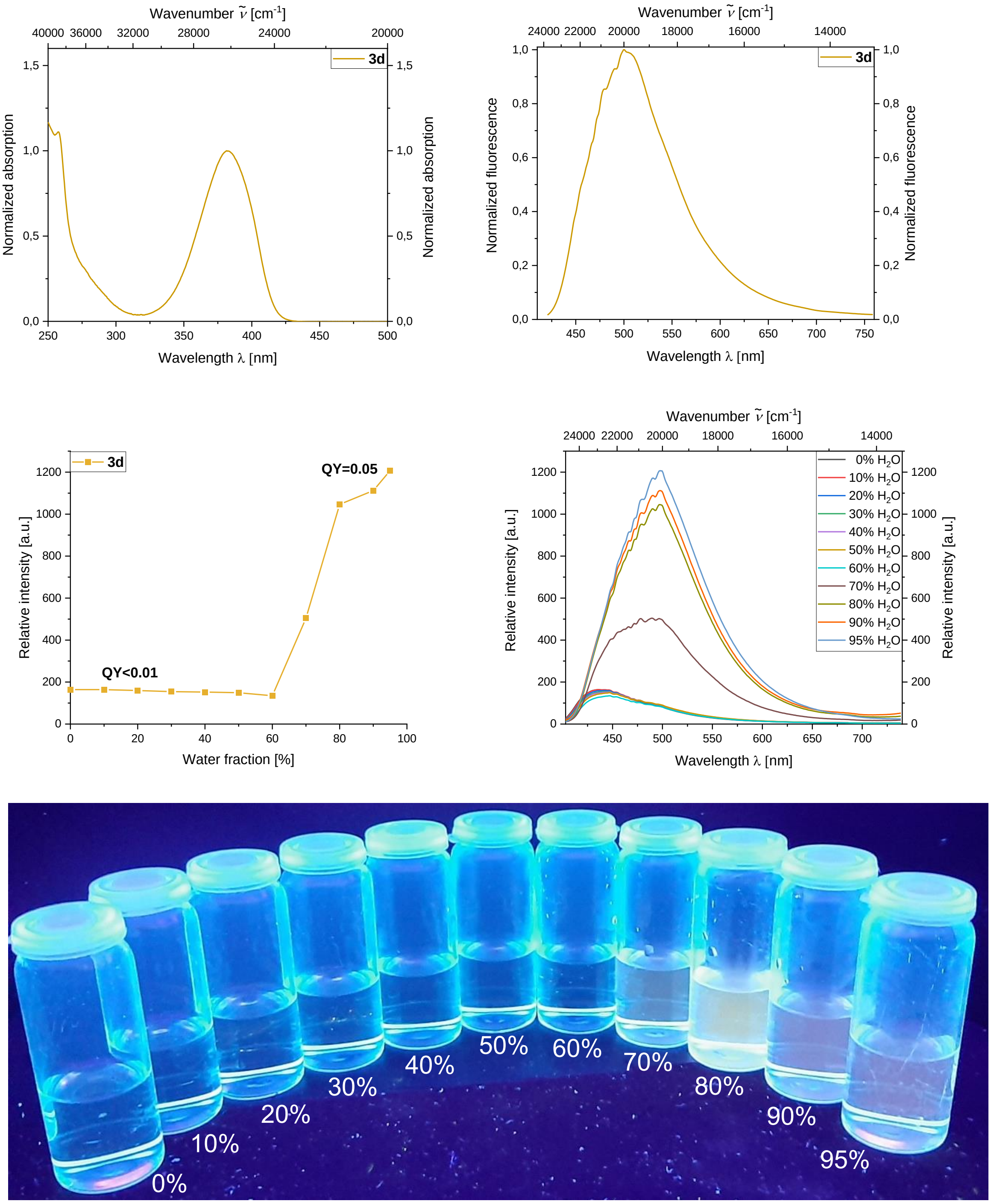

Figure S90. Absorption spectrum of **3d** in ethanol (top, left), solid-state emission spectrum (top, right), and AIE-induced changes in emission (center, left), AIE-related emission spectra of compound **3d** (center, right) and photographs of solutions of dye **3d** in ethanol/water mixtures of increasing water content (bottom). The latter spectra were measured in ethanol/water mixtures of varying water content.

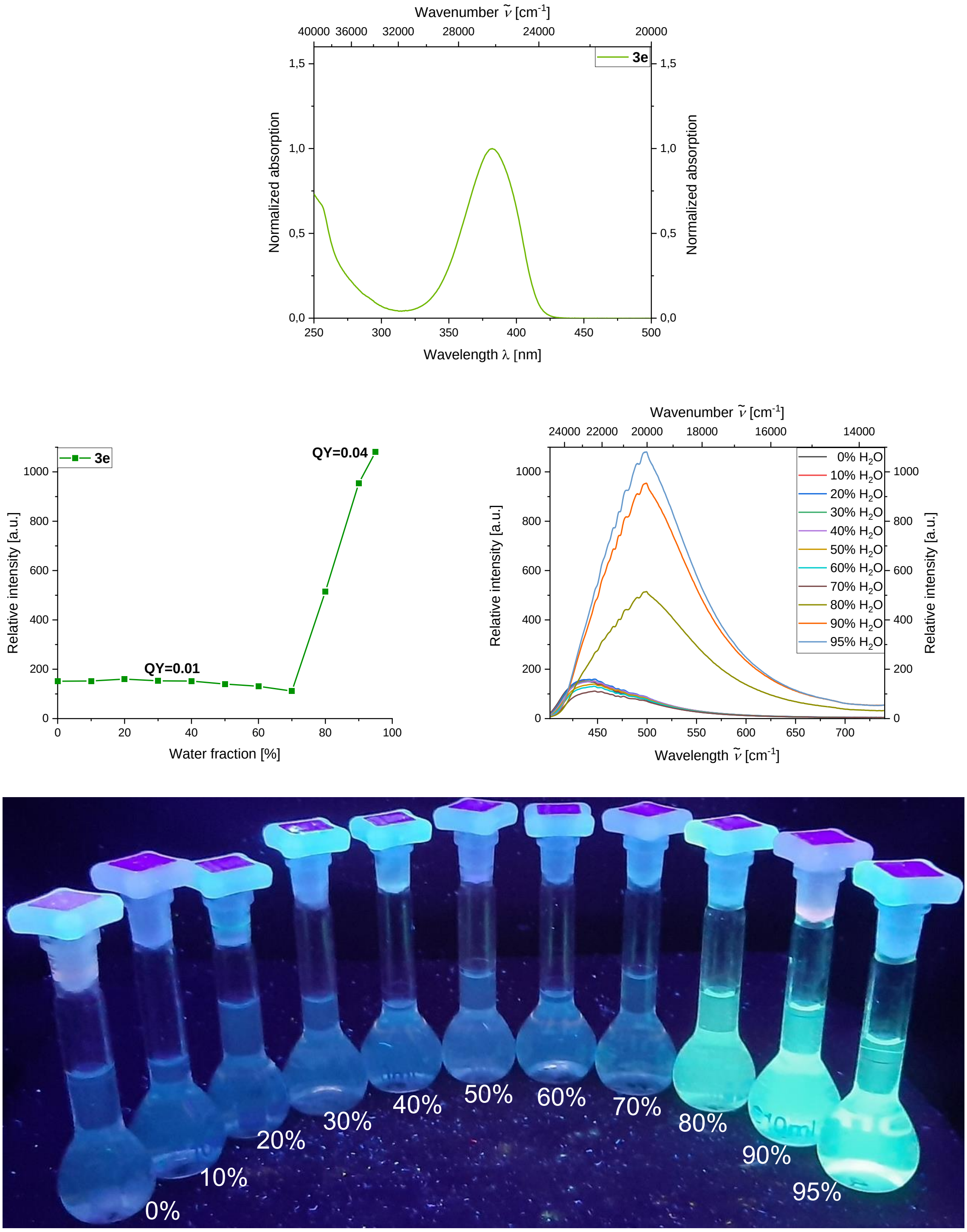

Figure S91. Absorption spectrum of **3e** in ethanol (top), and AIE-induced changes in emission (center, left), AIE-related emission spectra of compound **3e** (center, right) and photographs of solutions of dye **3e** in ethanol/water mixtures of increasing water content (bottom). The latter spectra were measured in ethanol/water mixtures of varying water content.

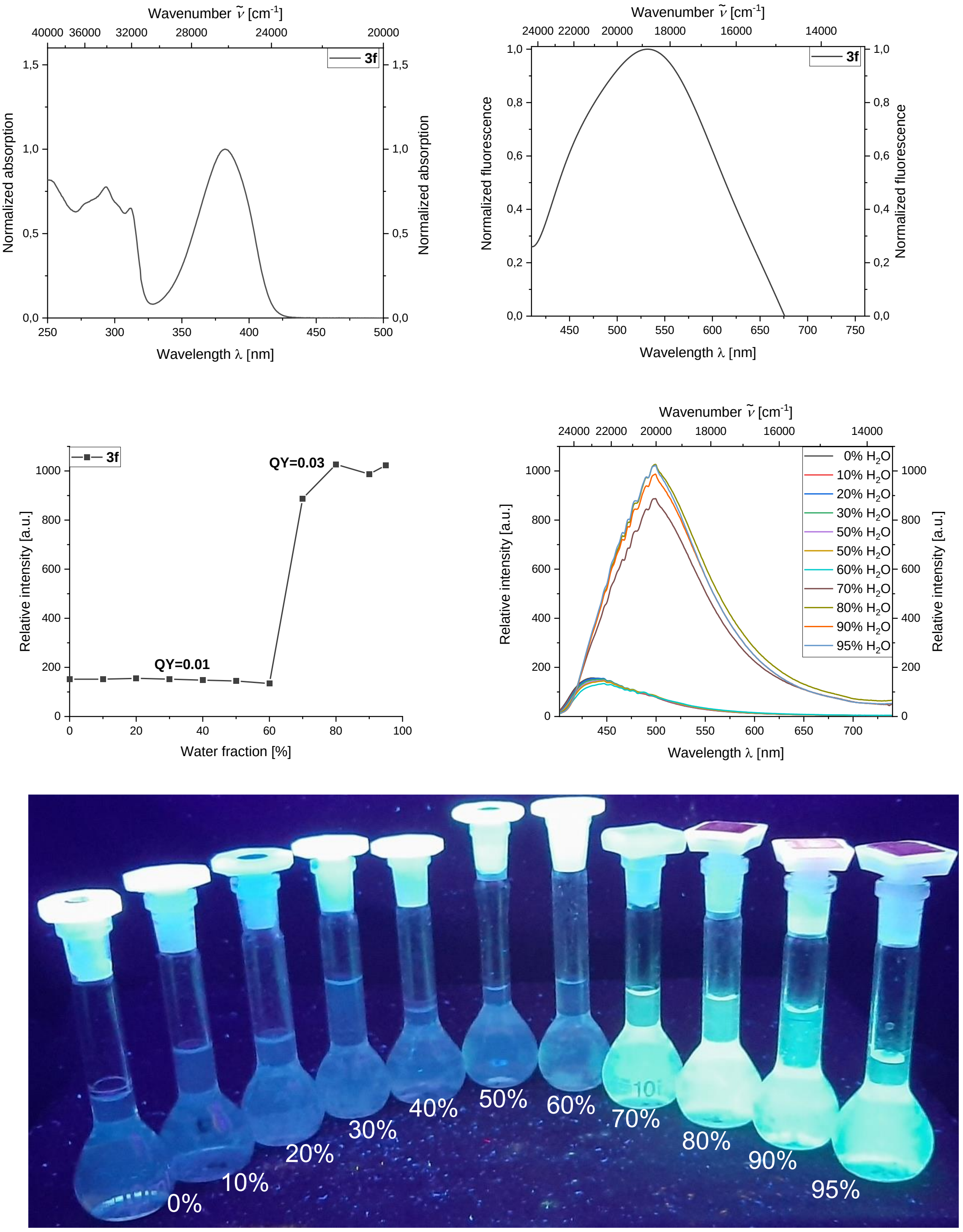

Figure S92. Absorption spectrum of **3f** in ethanol (top, left), solid-state emission spectrum (top, right), and AIE-induced changes in emission (center, left), AIE-related emission spectra of compound **3f** (center, right) and photographs of solutions of dye **3f** in ethanol/water mixtures of increasing water content (bottom). The latter spectra were measured in ethanol/water mixtures of varying water content.

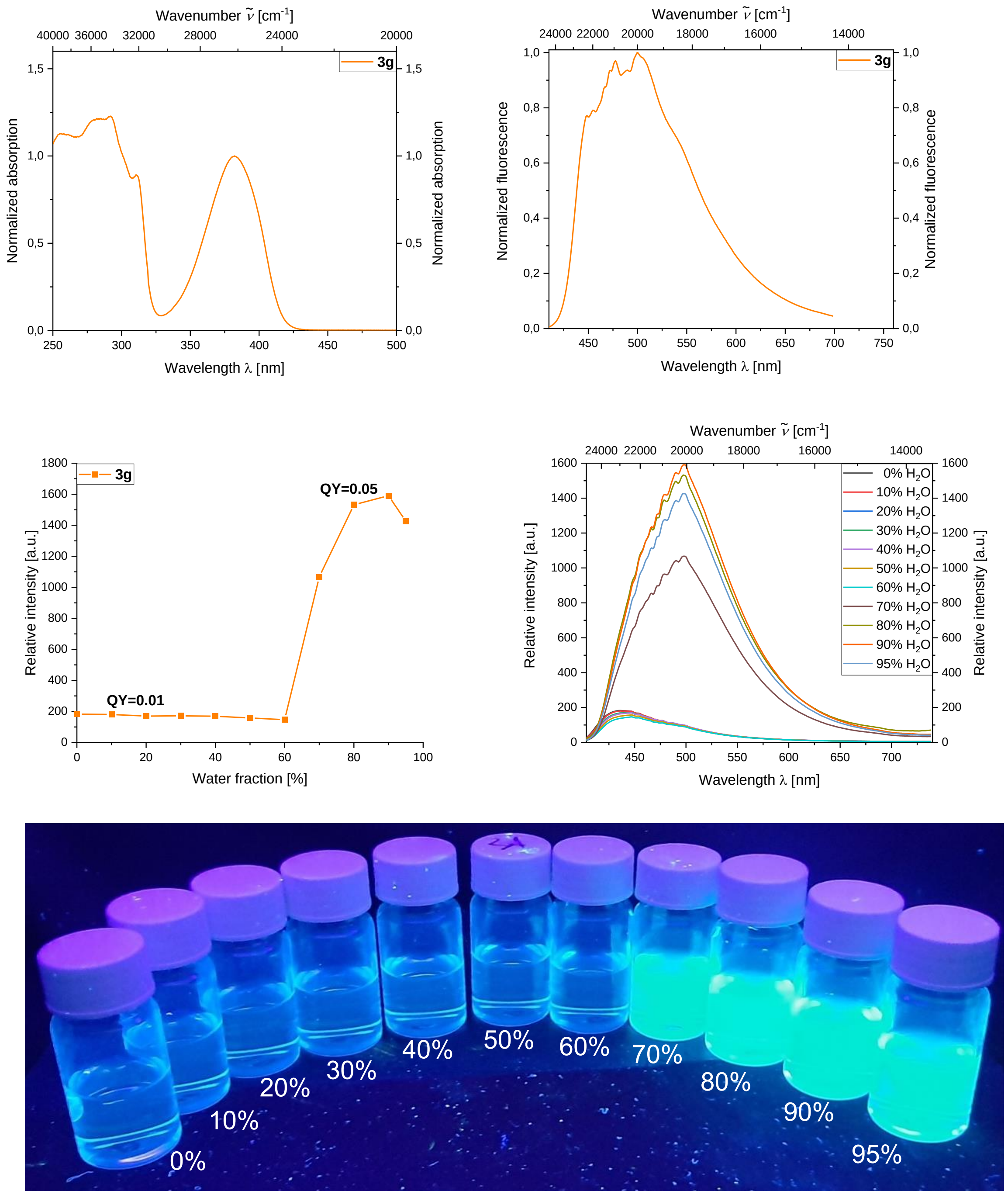

Figure S93. Absorption spectrum of **3g** in ethanol (top, left), solid-state emission spectrum (top, right), and AIE-induced changes in emission (center, left), AIE-related emission spectra of compound **3g** (center, right) and photographs of solutions of dye **3g** in ethanol/water mixtures of increasing water content (bottom). The latter spectra were measured in ethanol/water mixtures of varying water content.

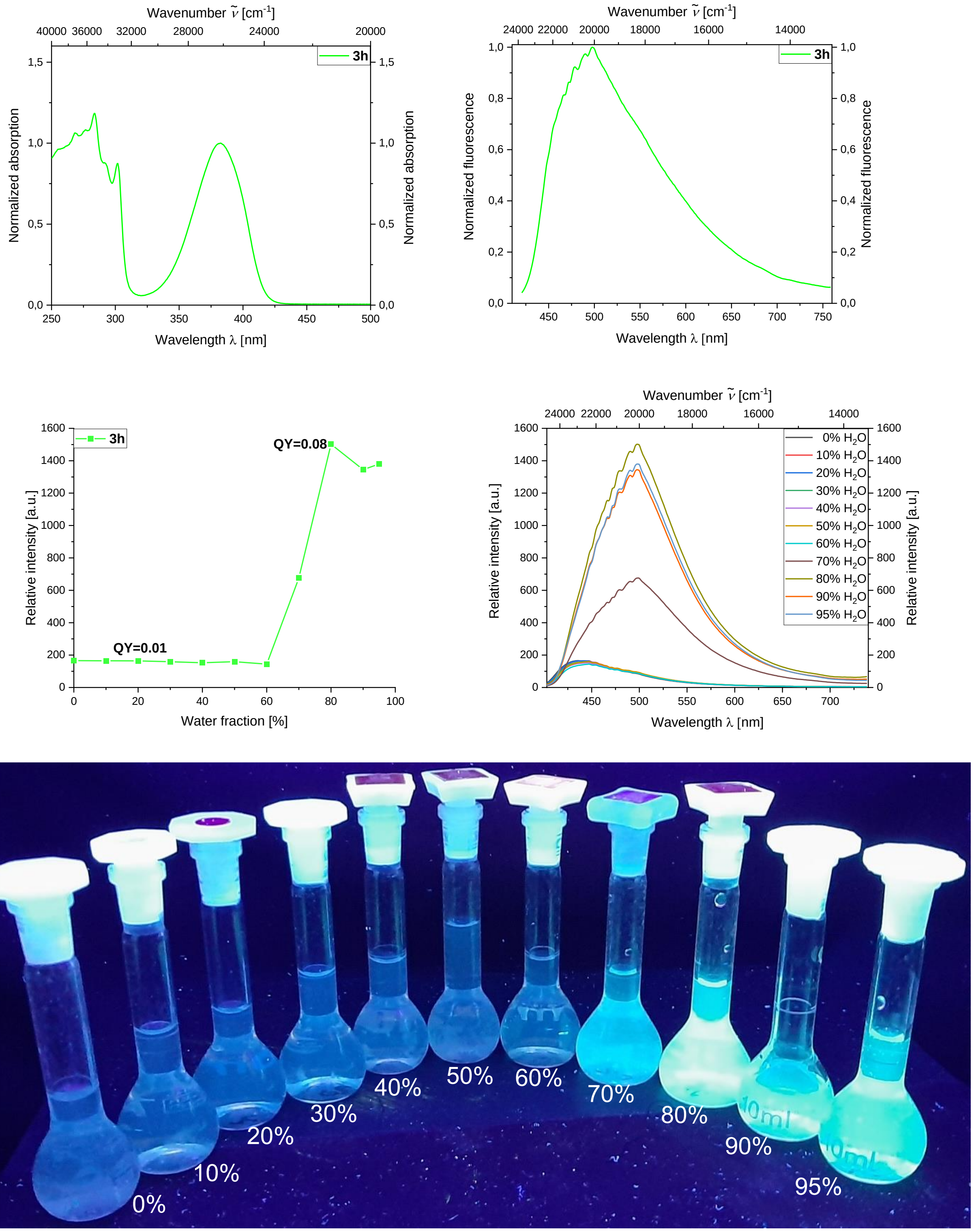

Figure S94. Absorption spectrum of **3h** in ethanol (top, left), solid-state emission spectrum (top, right), and AIE-induced changes in emission (center, left), AIE-related emission spectra of compound **3h** (center, right) and photographs of solutions of dye **3h** in ethanol/water mixtures of increasing water content (bottom). The latter spectra were measured in ethanol/water mixtures of varying water content.

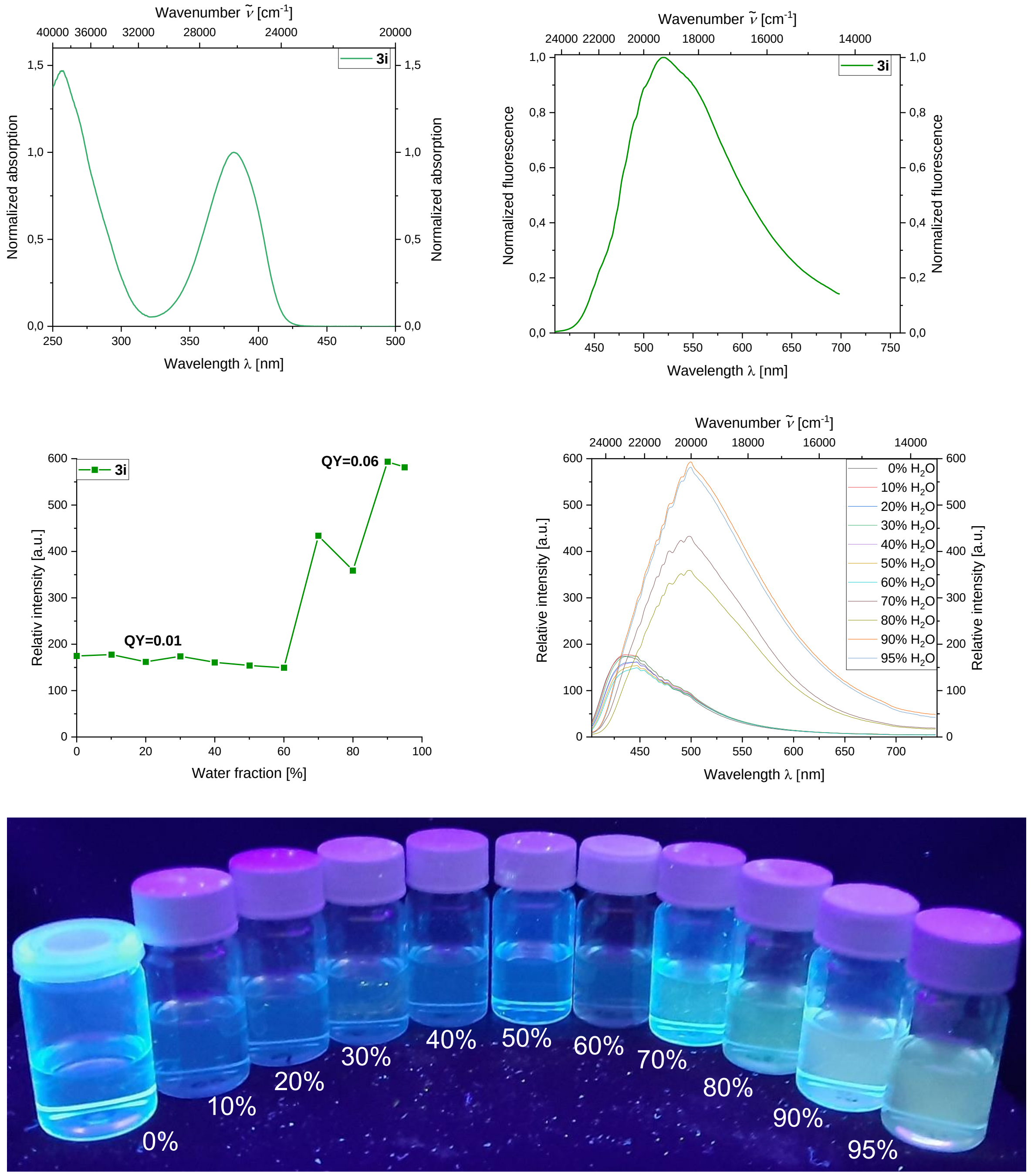

Figure S95. Absorption spectrum of **3i** in ethanol (top, left), solid-state emission spectrum (top, right), and AIE-induced changes in emission (center, left), AIE-related emission spectra of compound **3i** (center, right) and photographs of solutions of dye **3i** in ethanol/water mixtures of increasing water content (bottom). The latter spectra were measured in ethanol/water mixtures of varying water content.

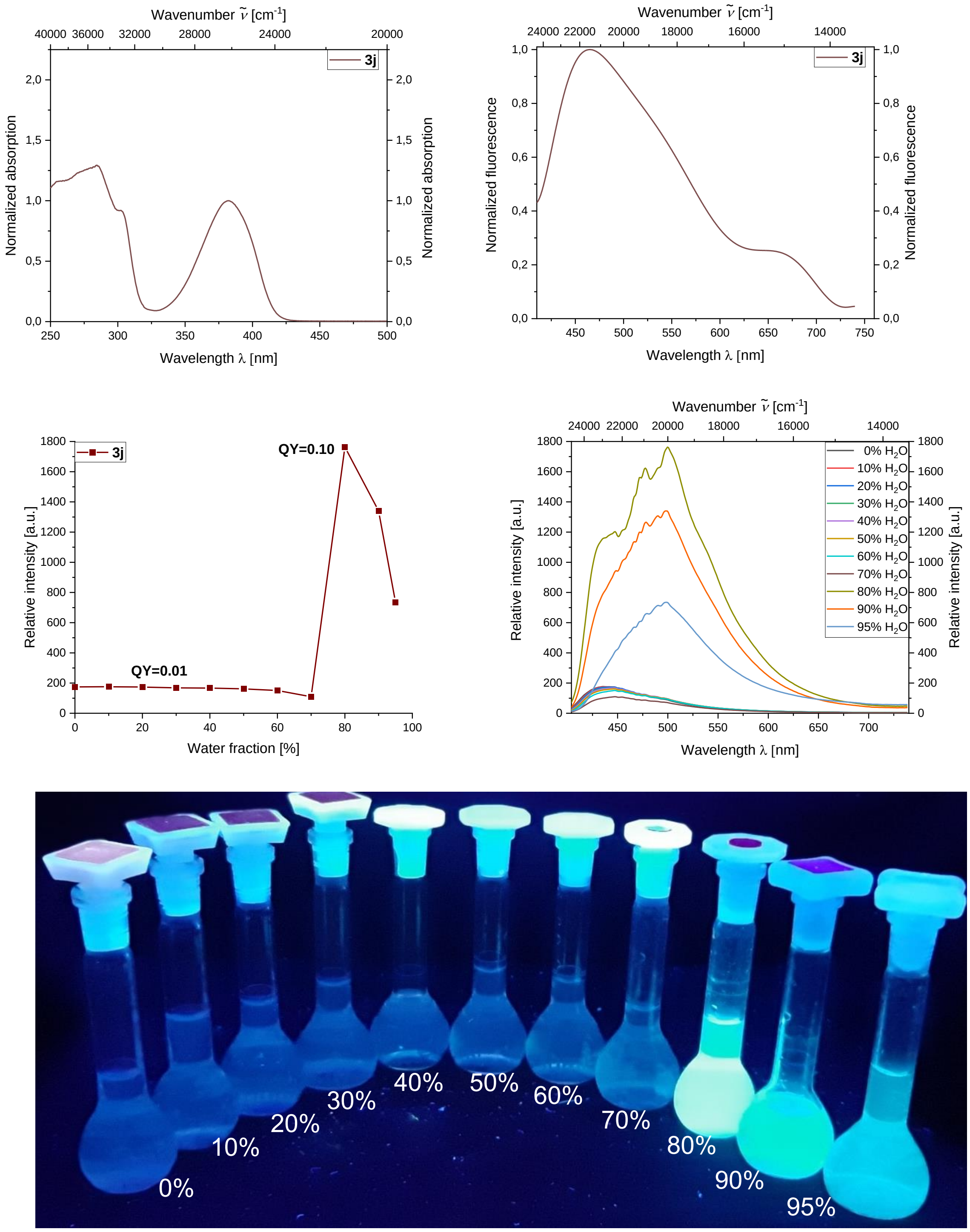

Figure S96. Absorption spectrum of **3j** in ethanol (top, left), solid-state emission spectrum (top, right), and AIE-induced changes in emission (center, left), AIE-related emission spectra of compound **3j** (center, right) and photographs of solutions of dye **3j** in ethanol/water mixtures of increasing water content (bottom). The latter spectra were measured in ethanol/water mixtures of varying water content.

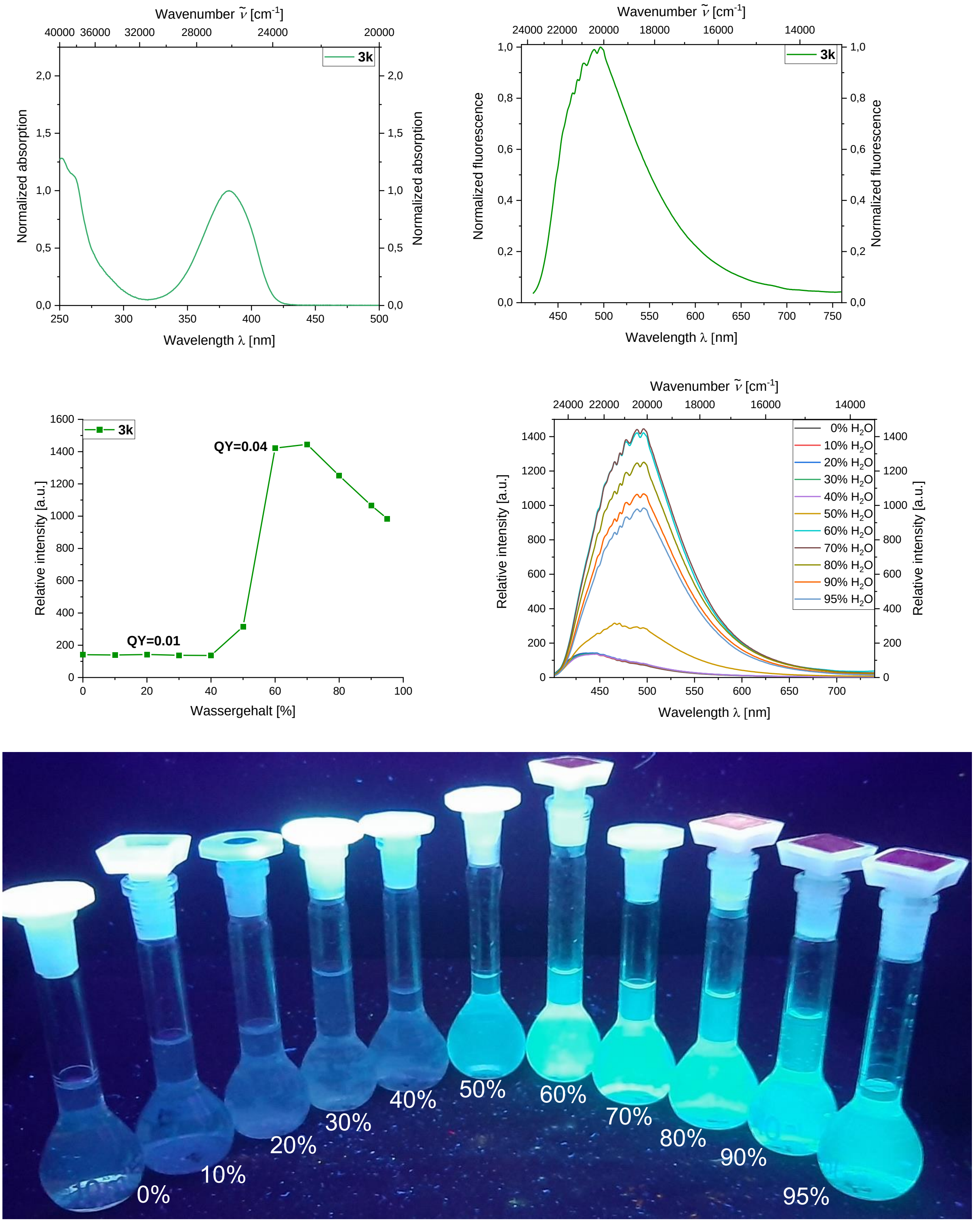

Figure S97. Absorption spectrum of **3k** in ethanol (top, left), solid-state emission spectrum (top, right), and AIE-induced changes in emission (center, left), AIE-related emission spectra of compound **3k** (center, right) and photographs of solutions of dye **3k** in ethanol/water mixtures of increasing water content (bottom). The latter spectra were measured in ethanol/water mixtures of varying water content.

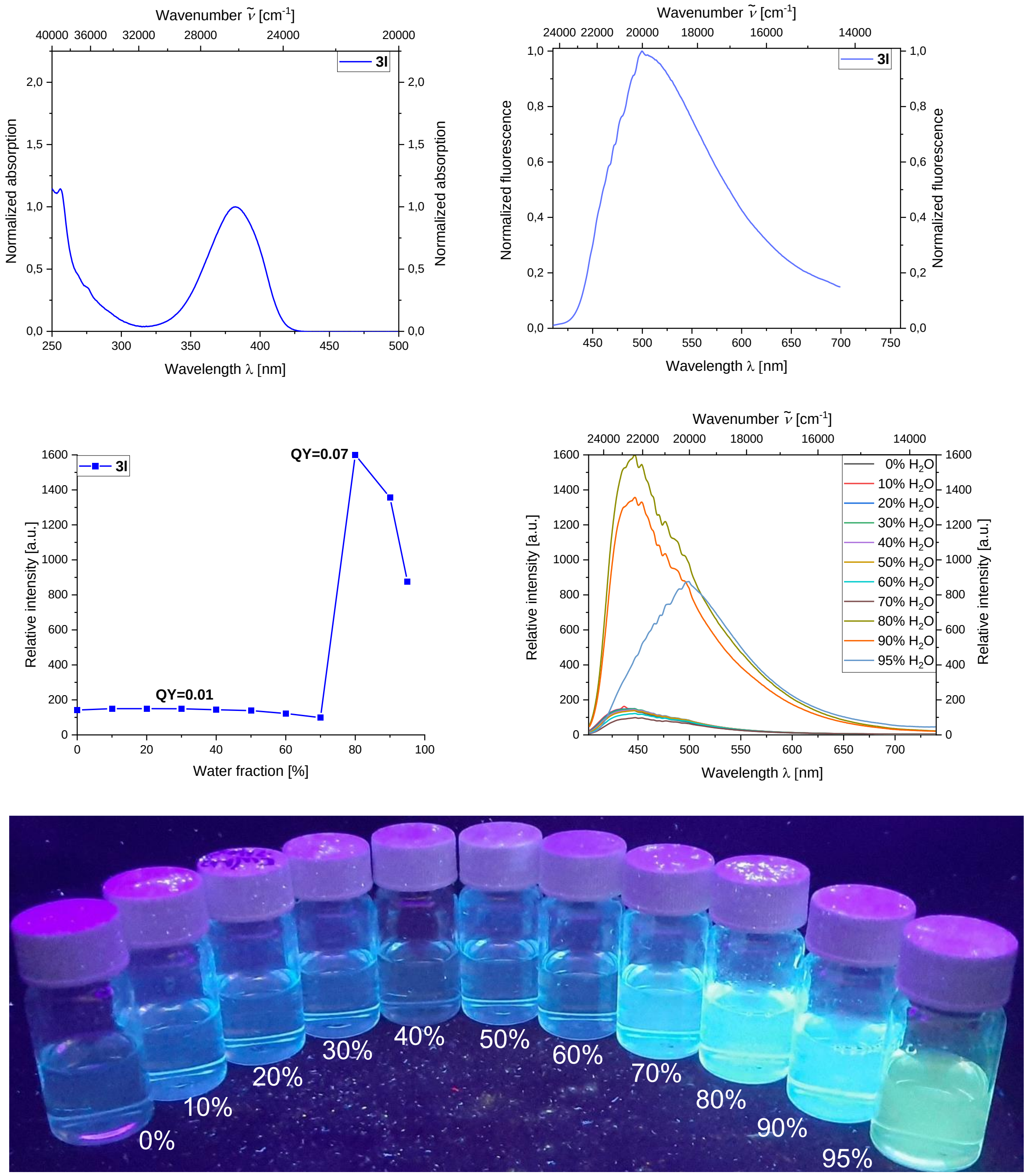

Figure S98. Absorption spectrum of **3l** in ethanol (top, left), solid-state emission spectrum (top, right), and AIE-induced changes in emission (center, left), AIE-related emission spectra of compound **3l** (center, right) and photographs of solutions of dye **3l** in ethanol/water mixtures of increasing water content (bottom). The latter spectra were measured in ethanol/water mixtures of varying water content.

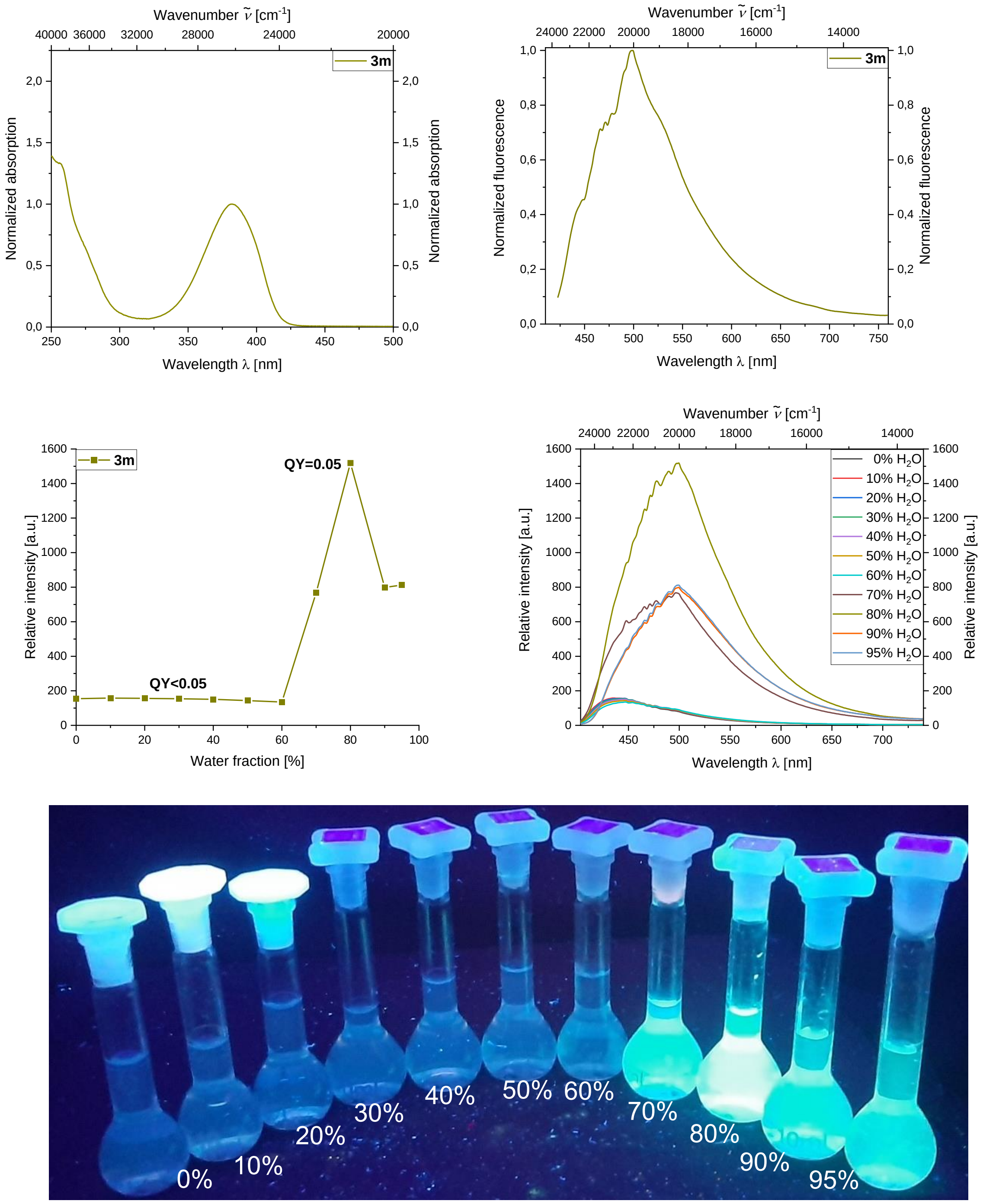

Figure S99. Absorption spectrum of **3m** in ethanol (top, left), solid-state emission spectrum (top, right), and AIE-induced changes in emission (center, left), AIE-related emission spectra of compound **3m** (center, right) and photographs of solutions of dye **3m** in ethanol/water mixtures of increasing water content (bottom). The latter spectra were measured in ethanol/water mixtures of varying water content.

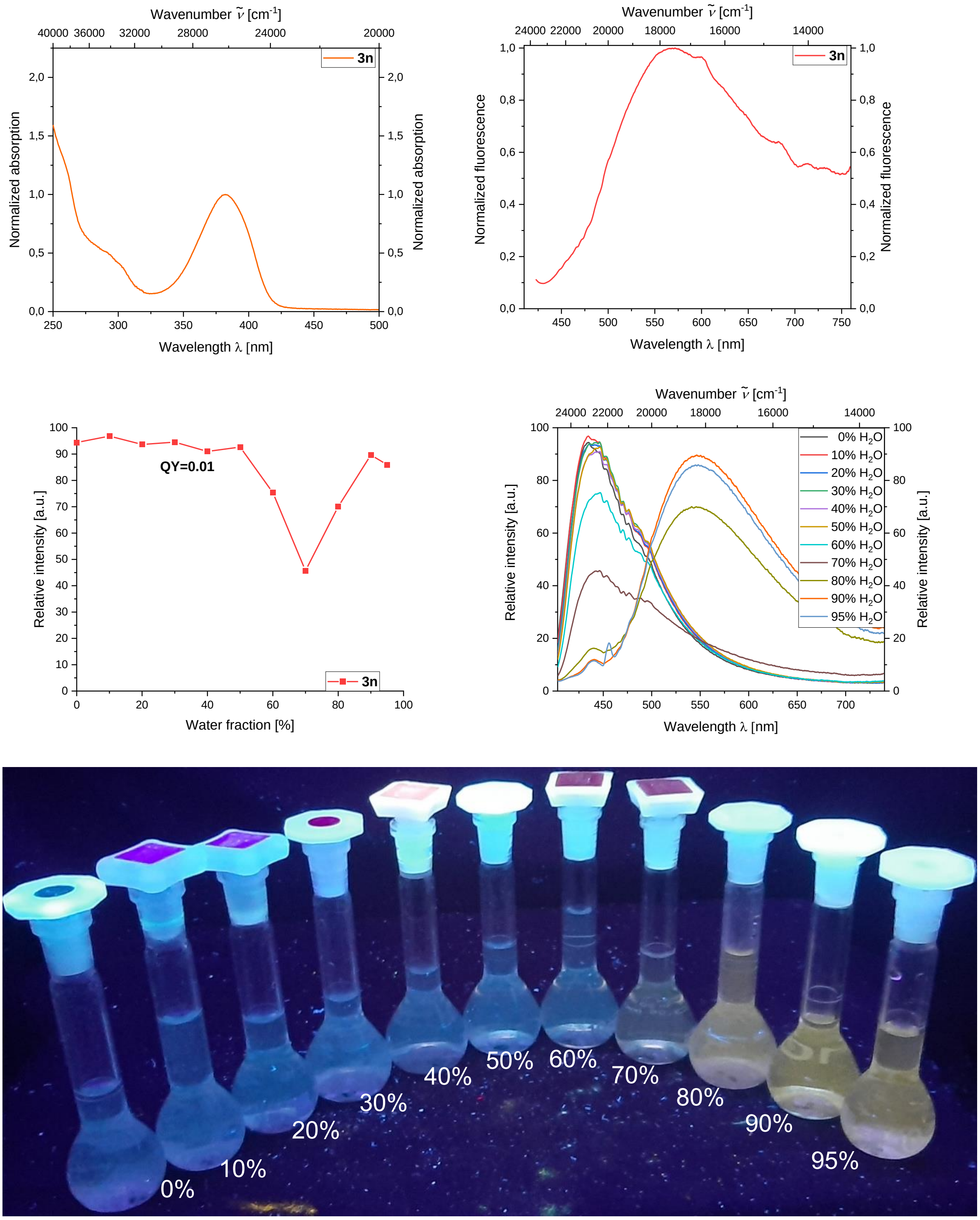

Figure S100. Absorption spectrum of **3n** in ethanol (top, left), solid-state emission spectrum (top, right), and AIE-induced changes in emission (center, left), AIE-related emission spectra of compound **3n** (center, right) and photographs of solutions of dye **3n** in ethanol/water mixtures of increasing water content (bottom). The latter spectra were measured in ethanol/water mixtures of varying water content.

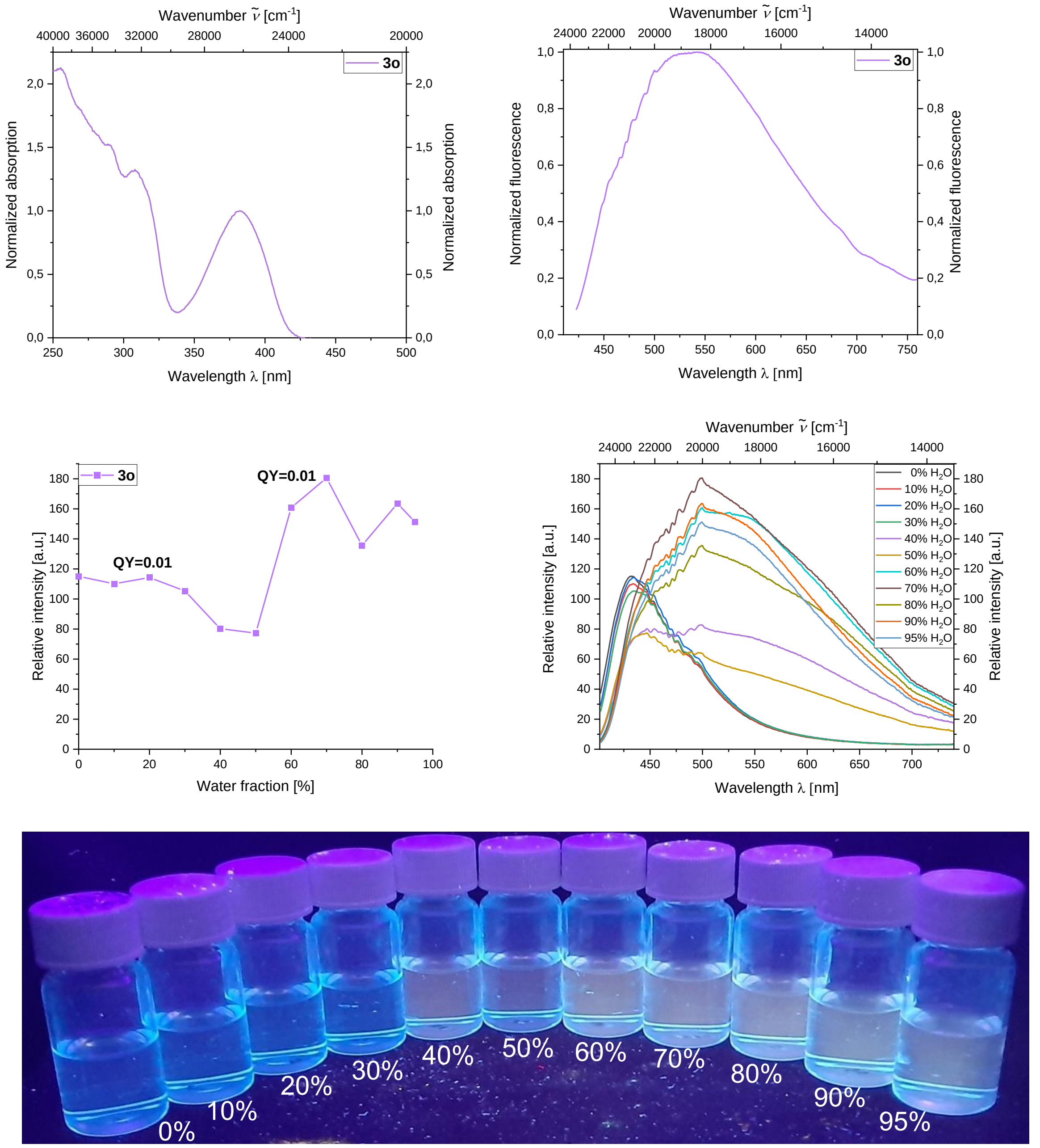

Figure S101. Absorption spectrum of **3o** in ethanol (top, left), solid-state emission spectrum (top, right), and AIE-induced changes in emission (center, left), AIE-related emission spectra of compound **3o** (center, right) and photographs of solutions of dye **3o** in ethanol/water mixtures of increasing water content (bottom). The latter spectra were measured in ethanol/water mixtures of varying water content.

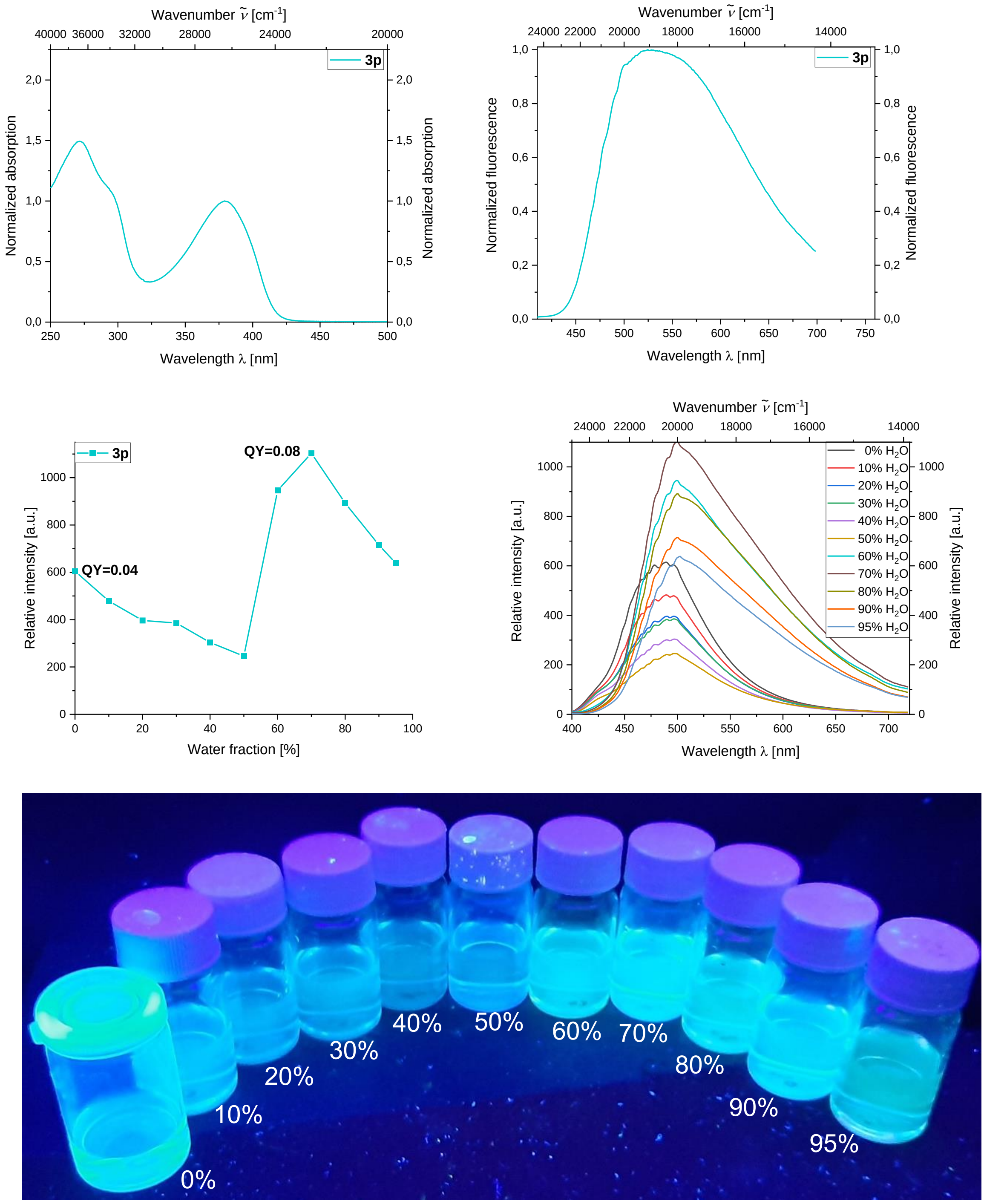

Figure S102. Absorption spectrum of **3p** in ethanol (top, left), solid-state emission spectrum (top, right), and AIE-induced changes in emission (center, left), AIE-related emission spectra of compound **3p** (center, right) and photographs of solutions of dye **3p** in ethanol/water mixtures of increasing water content (bottom). The latter spectra were measured in ethanol/water mixtures of varying water content.

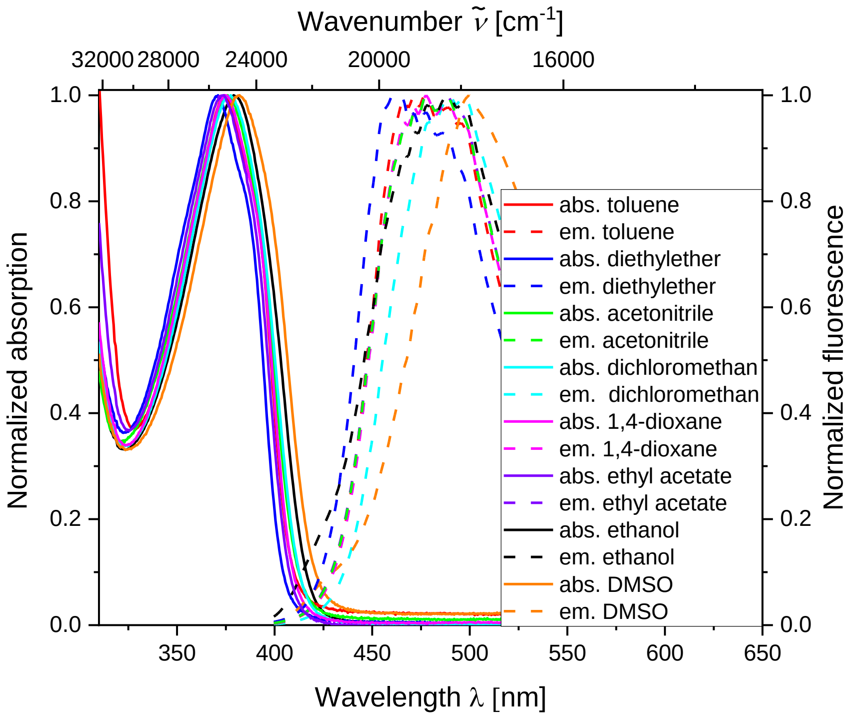

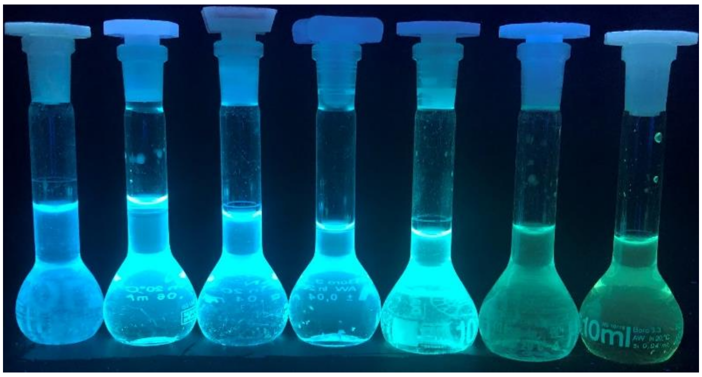

Figure S103. Comparison of absorption and emission maxima of compound **3p** in various solvents (*T* = 298 K, *c(abs.)* = 10^{-5} M, *c(em.)* = 10^{-7} M, $\lambda_{exc} = \lambda_{max(abs.)}$) and visual impression of emission solvatochromism of compound **3p** (UV-lamp, λ_{exc} = 365 nm, *T* = 298 K) in diethylether, 1,4-dioxane, ethyl acetate, toluene, dichloromethan, acetonitrile and DMSO (from left to right).

Table S11. Solvatochromism study of compound **3p**.

solvent	$\lambda_{max(abs.)}$[a] [nm]	$\lambda_{max(em.)}$[b] [nm]	Stokes shift $\Delta\tilde{\nu}$ [cm^{-1}][c]
DMSO	382	500	6180
Acetonitrile	376	500	6600
Dichloromethan	378	496	6290
Diethylether	371	471	5720
Toluene	375	477	5700
Ethyl acetate	375	478	5750
1,4-Dioxane	375	478	5750

[a]: T = 298 K, $c = 10^{-5}$ M, [b]: $c = 10^{-7}$ M, T = 298 K, $\lambda_{exc} = \lambda_{max(Abs.)}$; [c]: $\tilde{\nu} = \tilde{\nu}_{max(Abs.)} - \tilde{\nu}_{max(Em.)}$.

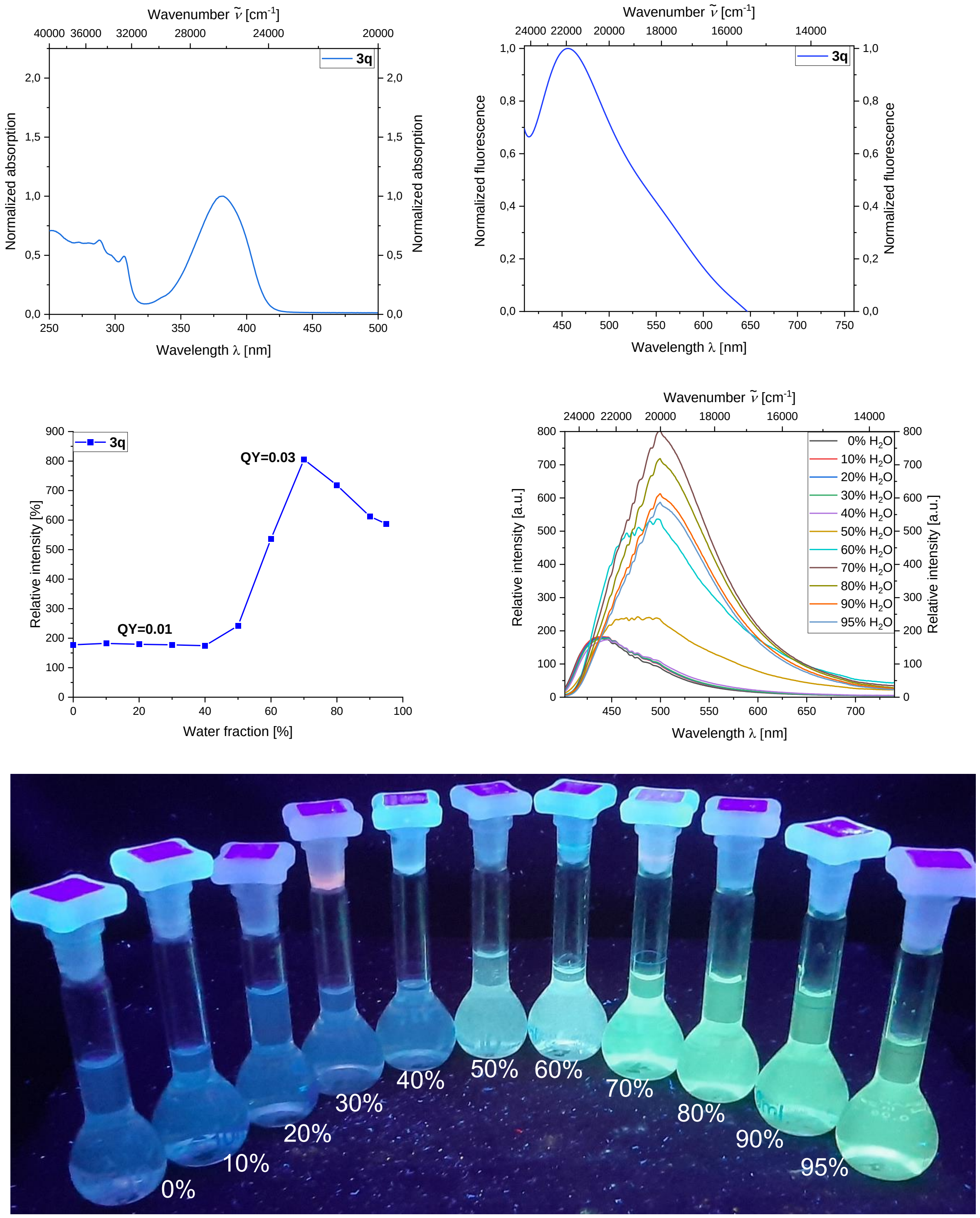

Figure S104. Absorption spectrum of **3q** in ethanol (top, left), solid-state emission spectrum (top, right), and AIE-induced changes in emission (center, left), AIE-related emission spectra of compound **3q** (center, right) and photographs of solutions of dye **3q** in ethanol/water mixtures of increasing water content (bottom). The latter spectra were measured in ethanol/water mixtures of varying water content.

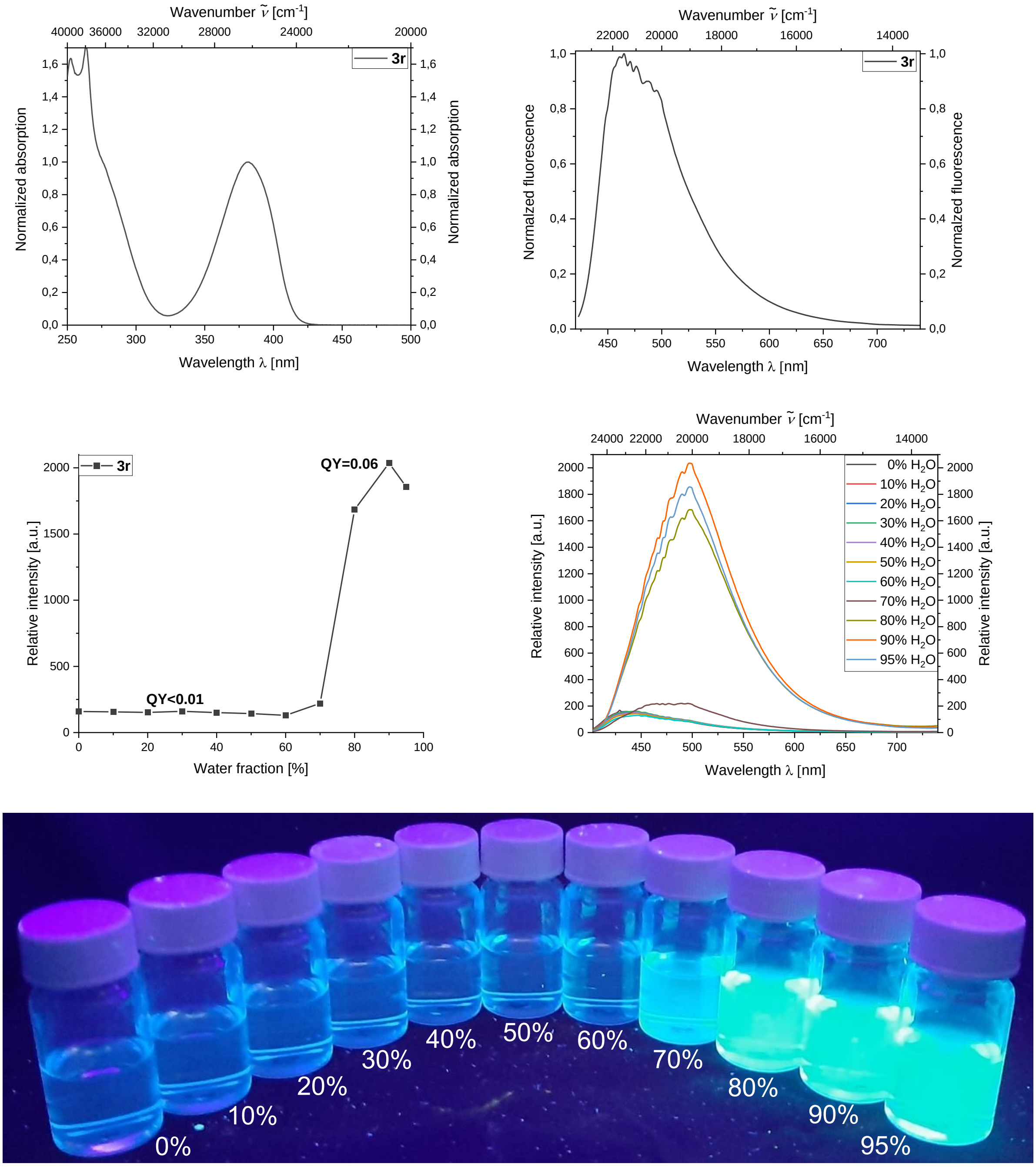

Figure S105. Absorption spectrum of **3r** in ethanol (top, left), solid-state emission spectrum (top, right), and AIE-induced changes in emission (center, left), AIE-related emission spectra of compound **3r** (center, right) and photographs of solutions of dye **3r** in ethanol/water mixtures of increasing water content (bottom). The latter spectra were measured in ethanol/water mixtures of varying water content.

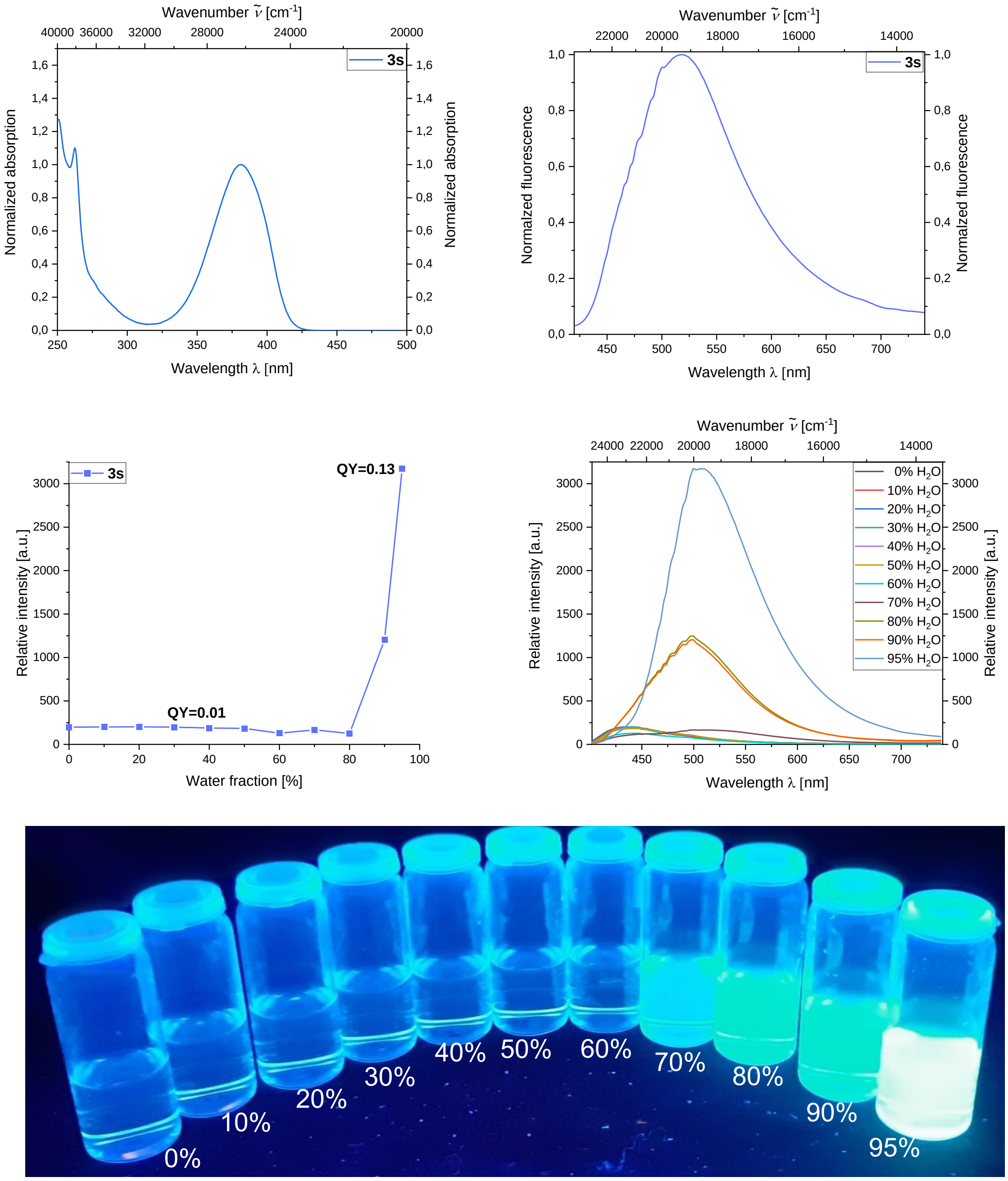

Figure S106. Absorption spectrum of **3s** in ethanol (top, left), solid-state emission spectrum (top, right), and AIE-induced changes in emission (center, left), AIE-related emission spectra of compound **3s** (center, right) and photographs of solutions of dye **3s** in ethanol/water mixtures of increasing water content (bottom). The latter spectra were measured in ethanol/water mixtures of varying water content.

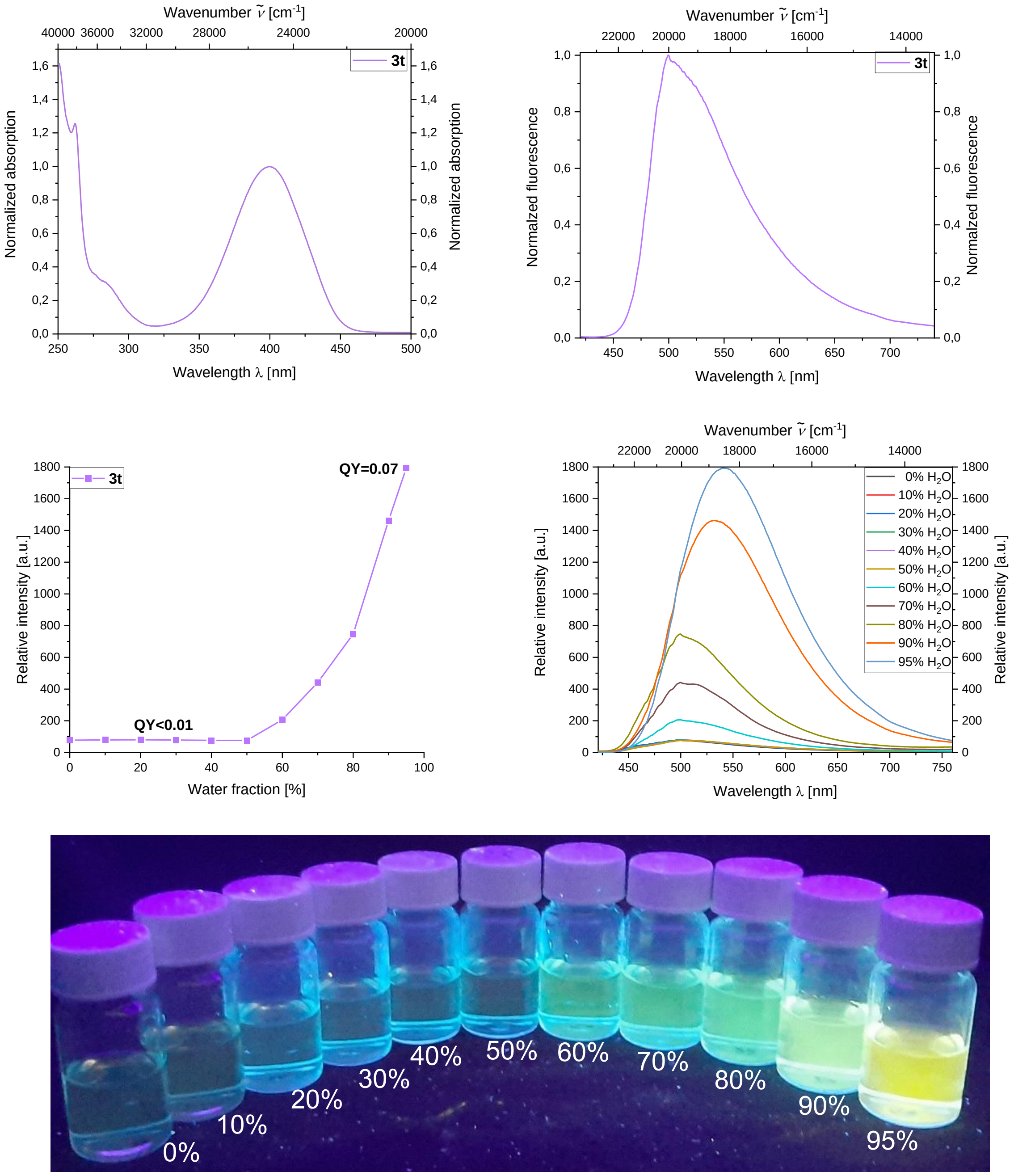

Figure S107. Absorption spectrum of **3t** in ethanol (top, left), solid-state emission spectrum (top, right), and AIE-induced changes in emission (center, left), AIE-related emission spectra of compound **3t** (center, right) and photographs of solutions of dye **3t** in ethanol/water mixtures of increasing water content (bottom). The latter spectra were measured in ethanol/water mixtures of varying water content.

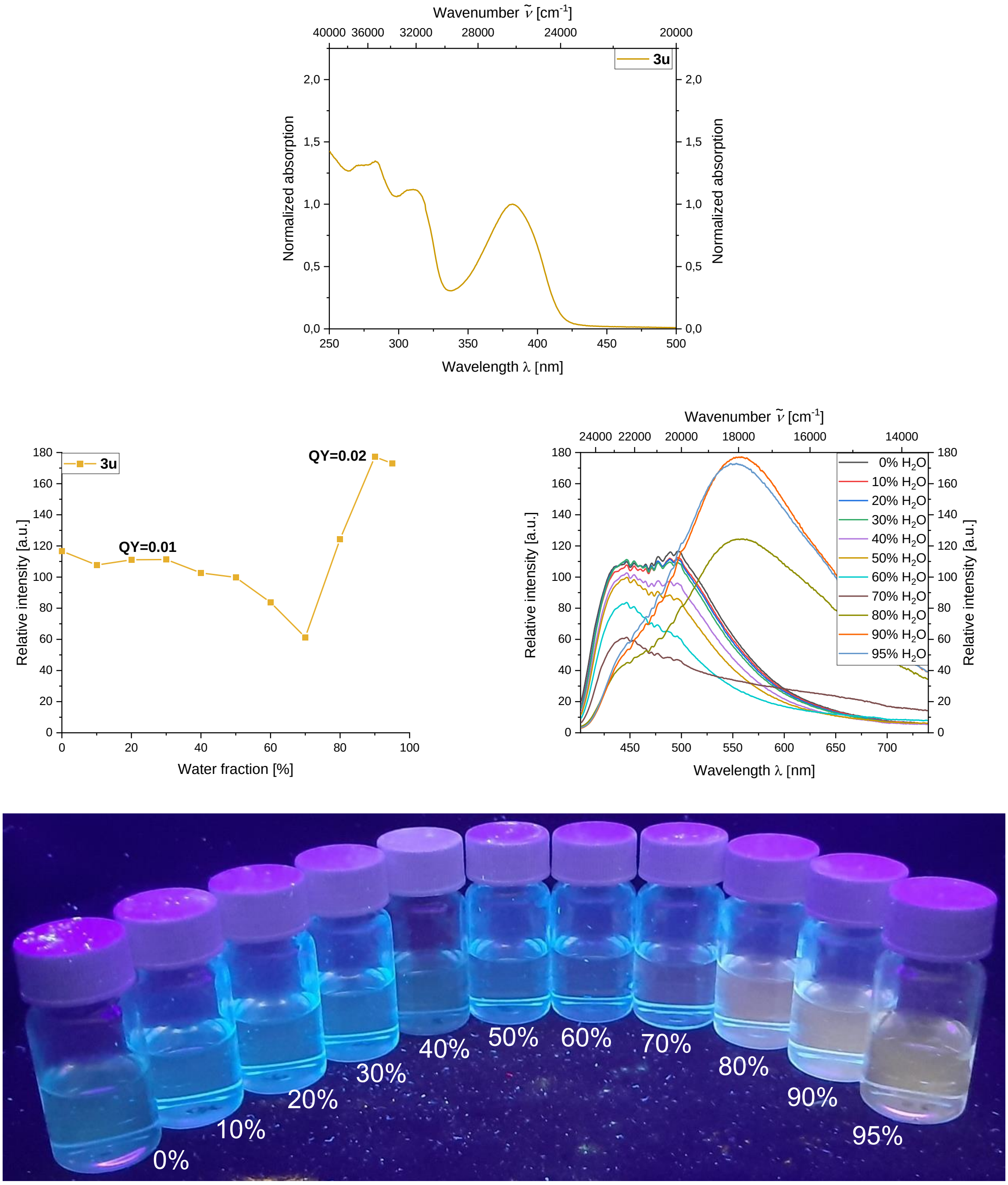

Figure S108. Absorption spectrum of **3u** in ethanol (top), and AIE-induced changes in emission (center, left), AIE-related emission spectra of compound **3u** (center, right) and photographs of solutions of dye **3u** in ethanol/water mixtures of increasing water content (bottom). The latter spectra were measured in ethanol/water mixtures of varying water content.

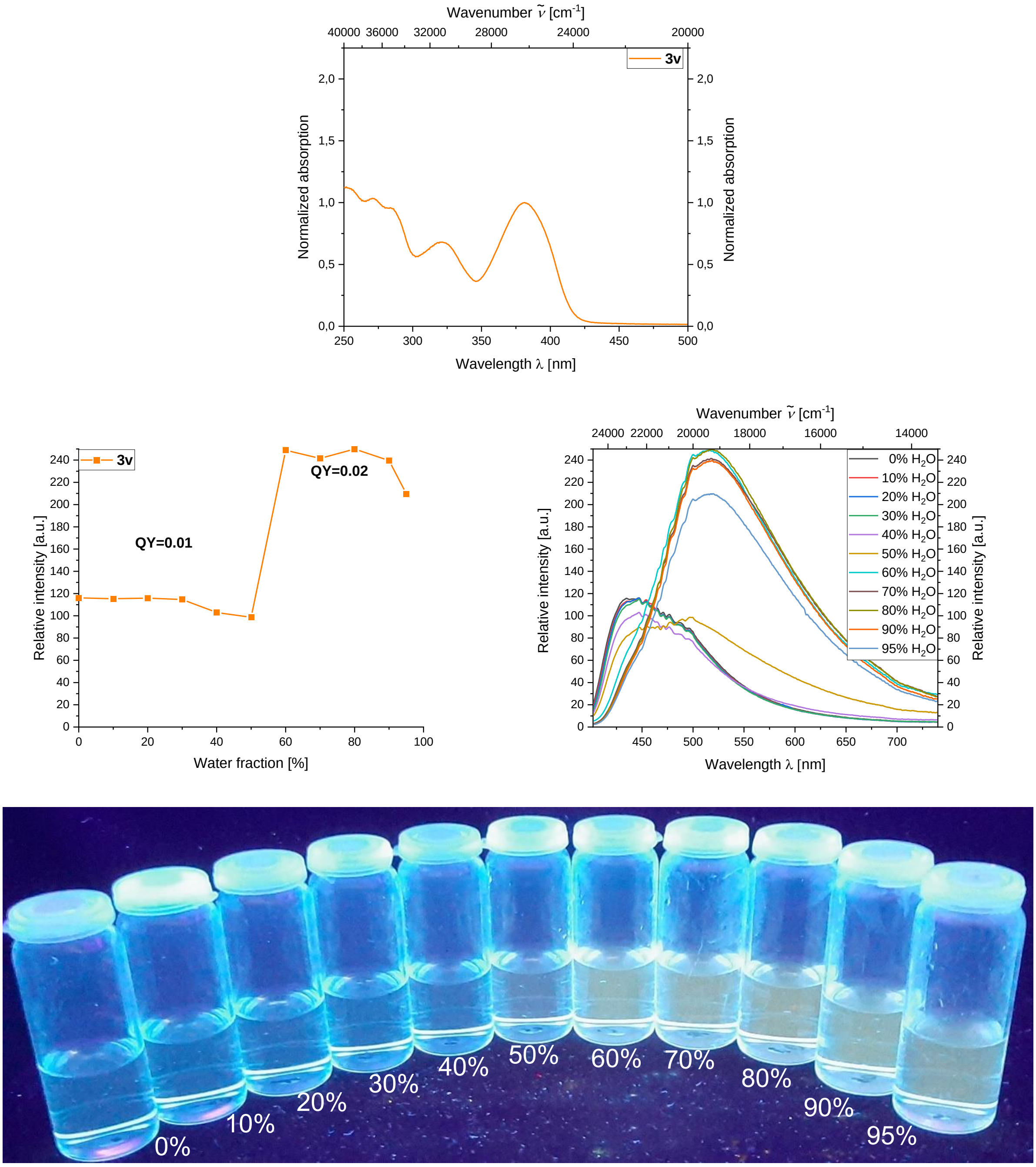

Figure S109. Absorption spectrum of **3v** in ethanol (top), and AIE-induced changes in emission (center, left), AIE-related emission spectra of compound **3v** (center, right) and photographs of solutions of dye **3v** in ethanol/water mixtures of increasing water content (bottom). The latter spectra were measured in ethanol/water mixtures of varying water content.

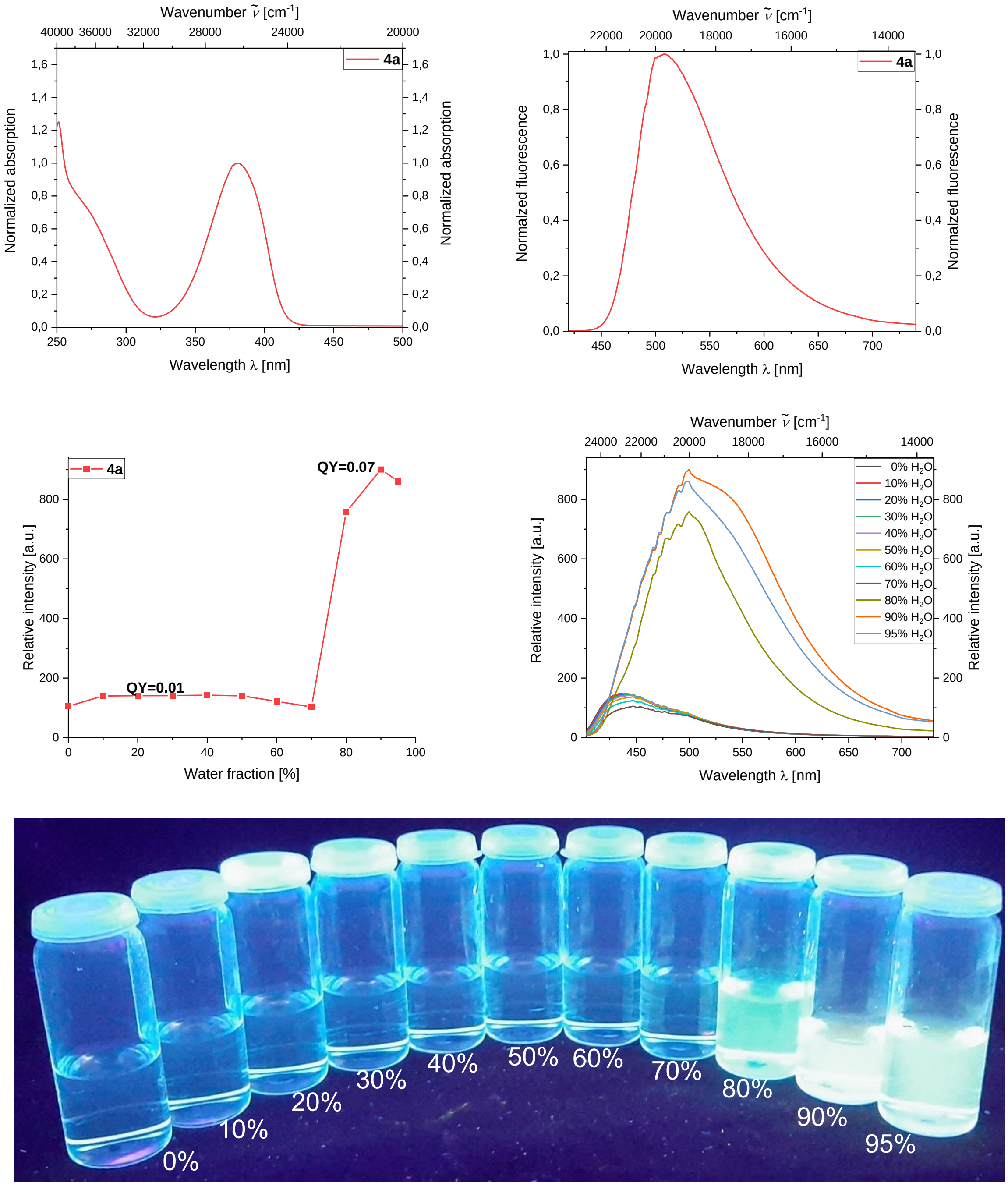

Figure S110. Absorption spectrum of **4a** in ethanol (top, left), solid-state emission spectrum (top, right), and AIE-induced changes in emission (center, left), AIE-related emission spectra of compound **4a** (center, right) and photographs of solutions of dye **4a** in ethanol/water mixtures of increasing water content (bottom). The latter spectra were measured in ethanol/water mixtures of varying water content.

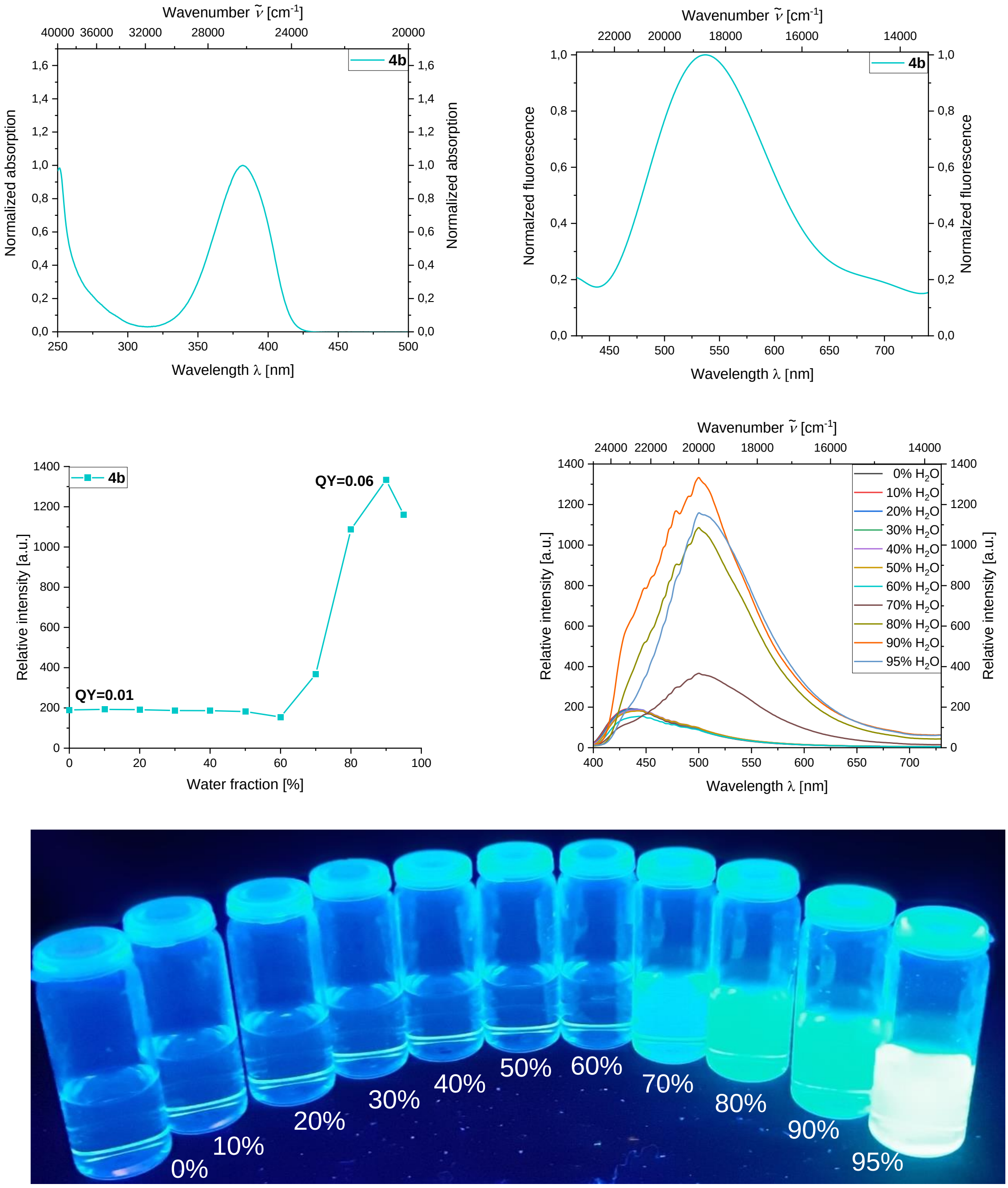

Figure S111. Absorption spectrum of **4b** in ethanol (top, left), solid-state emission spectrum (top, right), and AIE-induced changes in emission (center, left), AIE-related emission spectra of compound **4b** (center, right) and photographs of solutions of dye **4b** in ethanol/water mixtures of increasing water content (bottom). The latter spectra were measured in ethanol/water mixtures of varying water content.

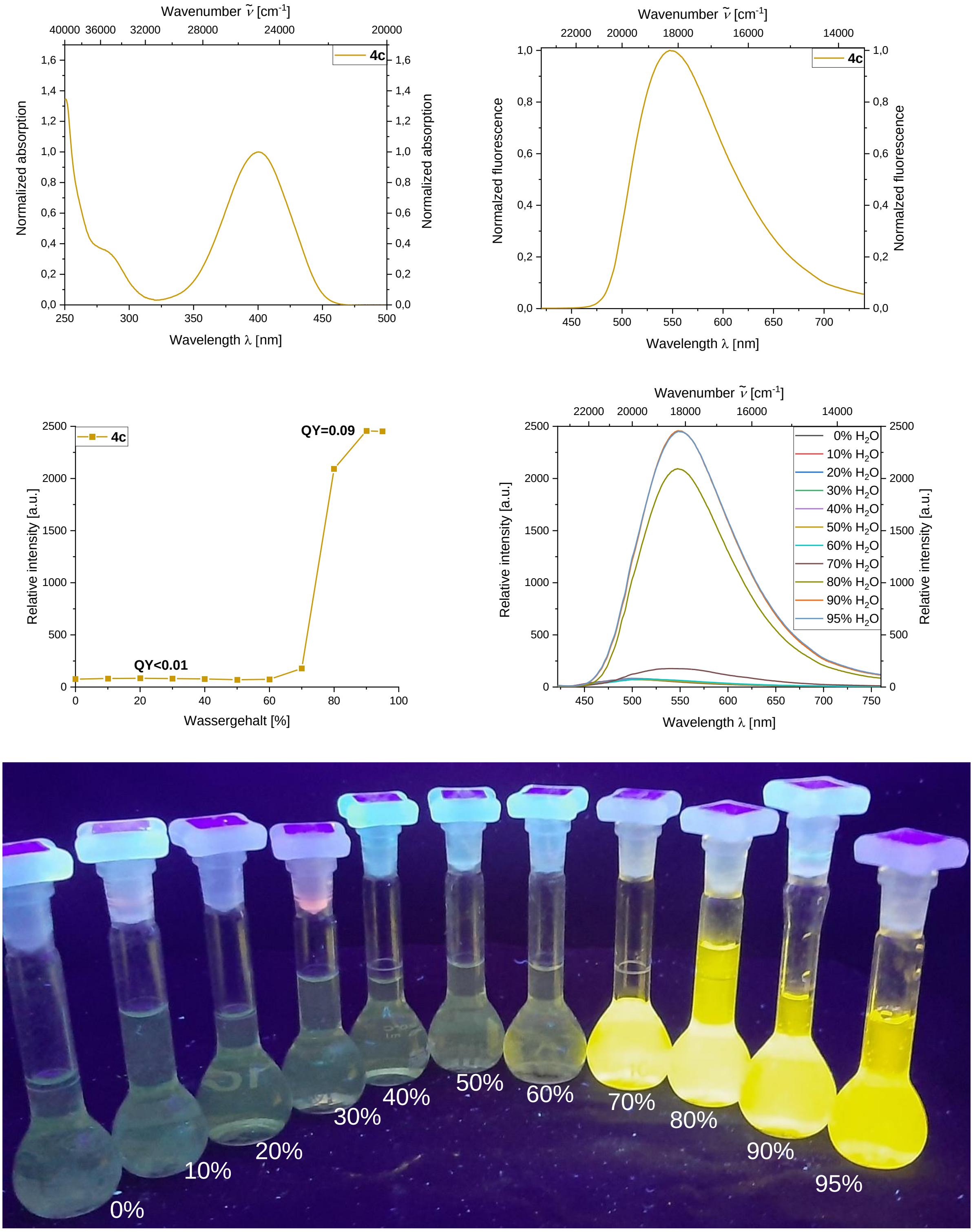

Figure S112. Absorption spectrum of **4c** in ethanol (top, left), solid-state emission spectrum (top, right), and AIE-induced changes in emission (center, left), AIE-related emission spectra of compound **4c** (center, right) and photographs of solutions of dye **4c** in ethanol/water mixtures of increasing water content (bottom). The latter spectra were measured in ethanol/water mixtures of varying water content.

9 Overview of photophysical properties of triazole aroyl-*S,N*-ketene acetals 6

All solution spectra were recorded in ethanol or in ethanol/water mixtures at T = 298 K, the excitation wavelengths λ_{exc} for the AIE-titration studies and the emission spectra in solution were determined from the absorption maxima λ_{max} of this compound, the excitation wavelength for the solid-state emission spectra was determined from solid-state excitation spectra. The dye concentration of the solution for absorption measurements was $c = 10^{-5}$ M and the dye concentration of the ethanol/water mixtures for AIE measurements was $c = 10^{-7}$ M.
The fluorescence quantum yields Φ_f in solution was below the detection limit of 0.01.

Table S12. Photophysical properties of triazole aroyl-*S,N*-ketene acetal **6**.

entry	compound	$\lambda_{max(abs.)}$[a] (ε [L·mol^{-1}·cm^{-1}])	$\lambda_{max(em.)}$ solution [nm][b]	Stokes Shift[c] $\Delta\tilde{\nu}$ [cm^{-1}]	$\lambda_{max(em.)}$[d] solid state [nm] (Φ_f)	solid state picture[e]
1	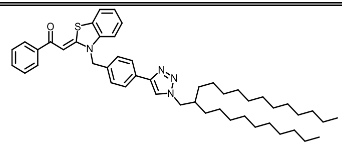 **6a**	383 (29100)	435	3120	-	-
2	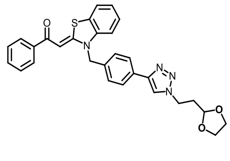 **6b**	381 (24400)	435	3260	460 (0.73)	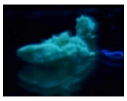
3	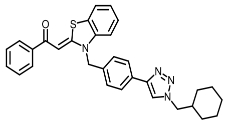 **6c**	382 (37800)	435	3190	470, 505 (0.22)	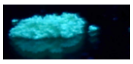
4	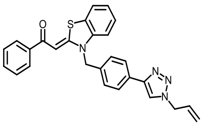 **6d**	382 (33900)	435	3190	500 (0.16)	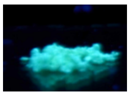
5	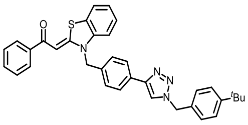 **6e**	383 (37400)	434	3070	456, 499 (0.14)	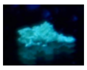

Table S12 continued.

entry	compound	$\lambda_{max(abs.)}$[a] (ε [L·mol^{-1}·cm^{-1}])	$\lambda_{max(em.)}$ solution [nm][b]	Stokes Shift[c] $\Delta\tilde{\nu}$ [cm^{-1}]	$\lambda_{max(em.)}$[d] solid state [nm] (Φ_f)	solid state picture[e]
6	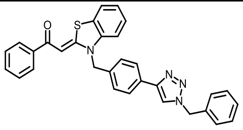 **6f**	383 (26700)	435	3120	471, 533 (0.14)	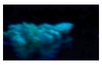
7	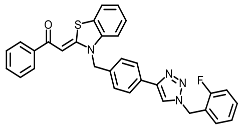 **6g**	382 (32300)	435	3190	470 (0.24)	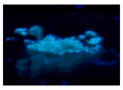
8	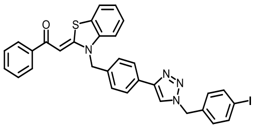 **6h**	382 (31200)	436	3240	465 (0.21)	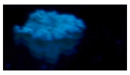
9	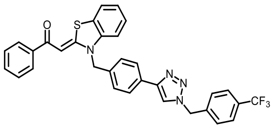 **6i**	382 (39400)	435	3190	466, 499 (0.15)	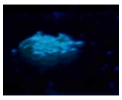
10	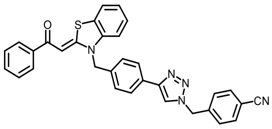 **6j**	382 (28100)	435	3190	454, 530 (0.20)	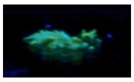
11	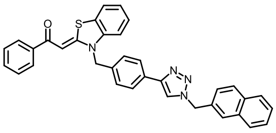 **6k**	382 (25300)	435	3190	496 (0.46)	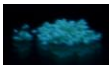

[a]: measured in ethanol, T = 298 K, c = 10^{-5} M, [b]: c = 10^{-7} M, T = 298 K, λ_{exc} = $\lambda_{max(abs.)}$; [c]: $\tilde{\nu} = \tilde{\nu}_{max(abs.)} - \tilde{\nu}_{max(em.)}$, [d]: T = 298 K, λ_{exc} = $\lambda_{max(abs.)}$, [e]: pictures taken under UV-light (λ_{exc} = 365 nm).

10 Absorption and emission spectra

All solution spectra were recorded in ethanol or in ethanol/water mixtures at T = 298 K, the excitation wavelengths λ_{exc} for the AIE-titration studies and the emission spectra in solution were determined from the absorption maxima λ_{max} of this compound, the excitation wavelength for the solid-state emission spectra was determined from solid-state excitation spectra. The dye concentration of the solution for absorption measurements was c(**6**) = 10^{-5} M and the dye concentration of the ethanol/water mixtures for AIE measurements was c(**6**) = 10^{-7} M.

10.1 Absorption and emission spectra of triazole aroyl-*S,N*-ketene acetals 6

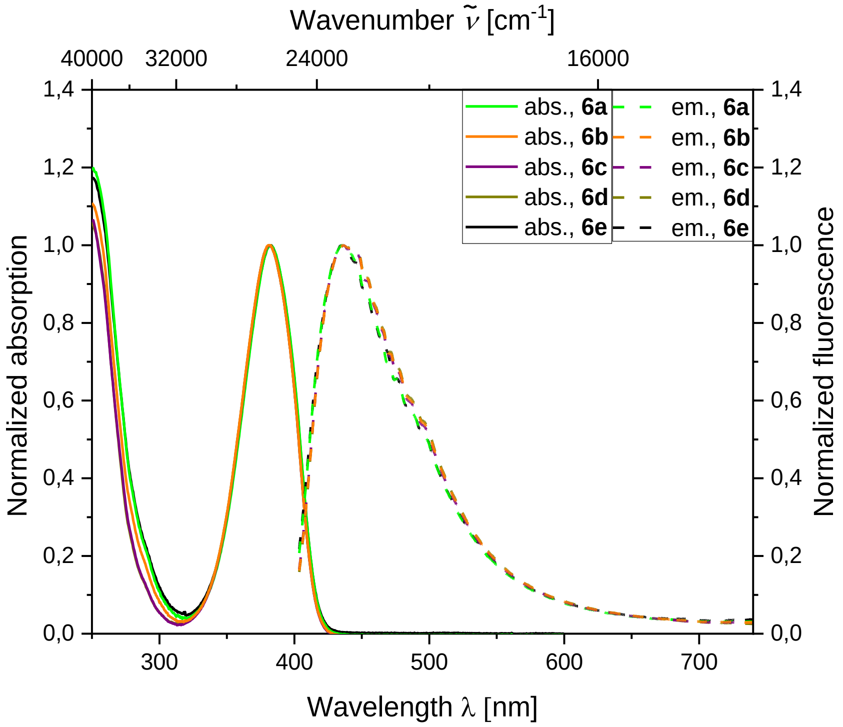

Figure S113. Selected, normalized UV/Vis absorption and emission bands of selected triazole aroyl-*S,N*-ketene acetals **6**.

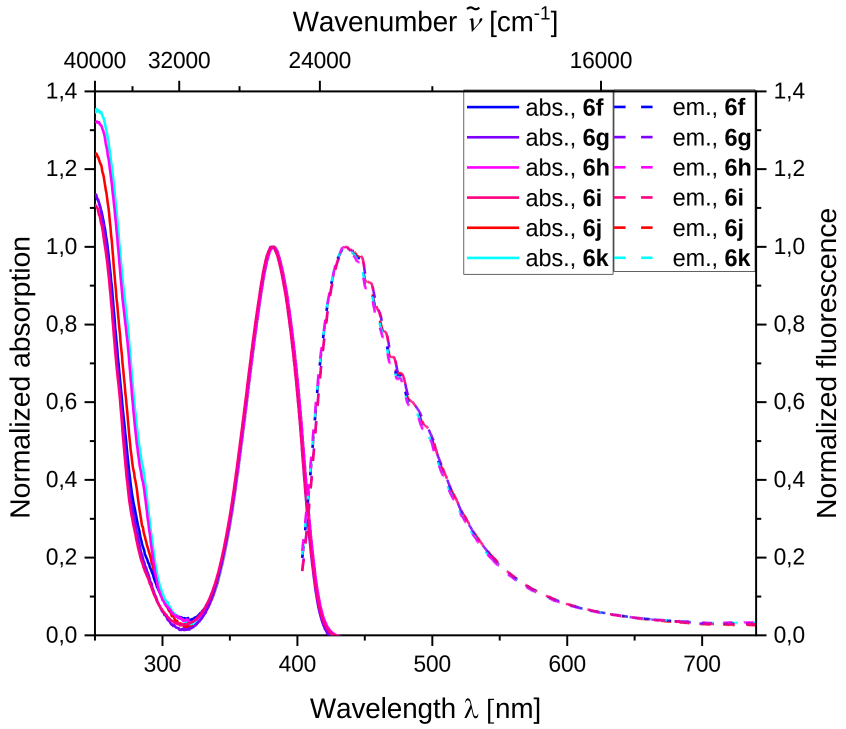

Figure S114. Selected, normalized UV/Vis absorption and emission bands of selected triazole aroyl-*S,N*-ketene acetals **6**.

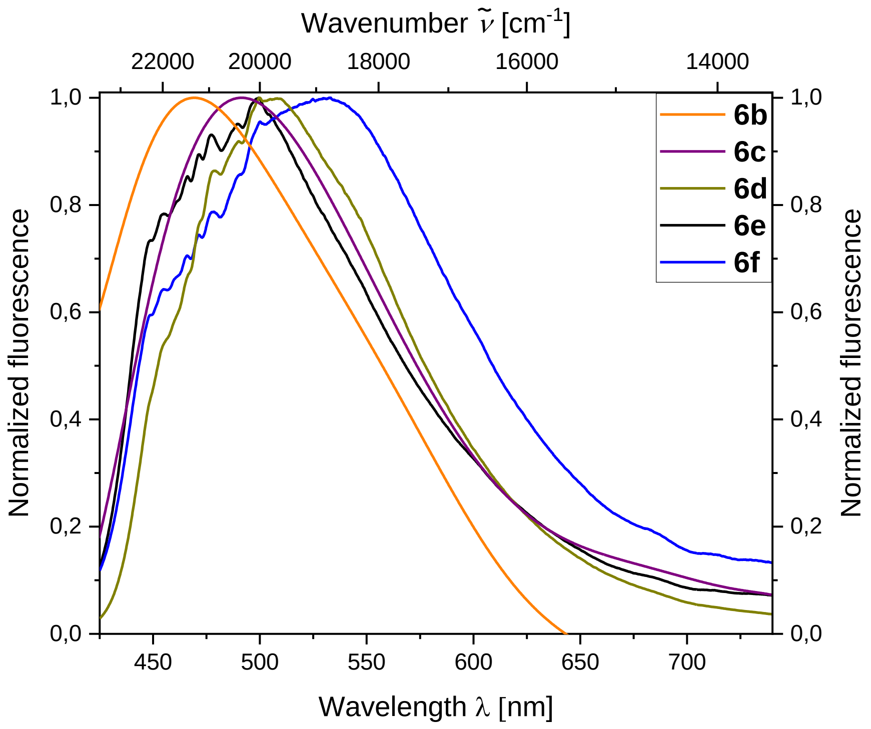

Figure S115. Normalized solid-state emission bands of selected triazole aroyl-*S,N*-ketene acetals **6**.

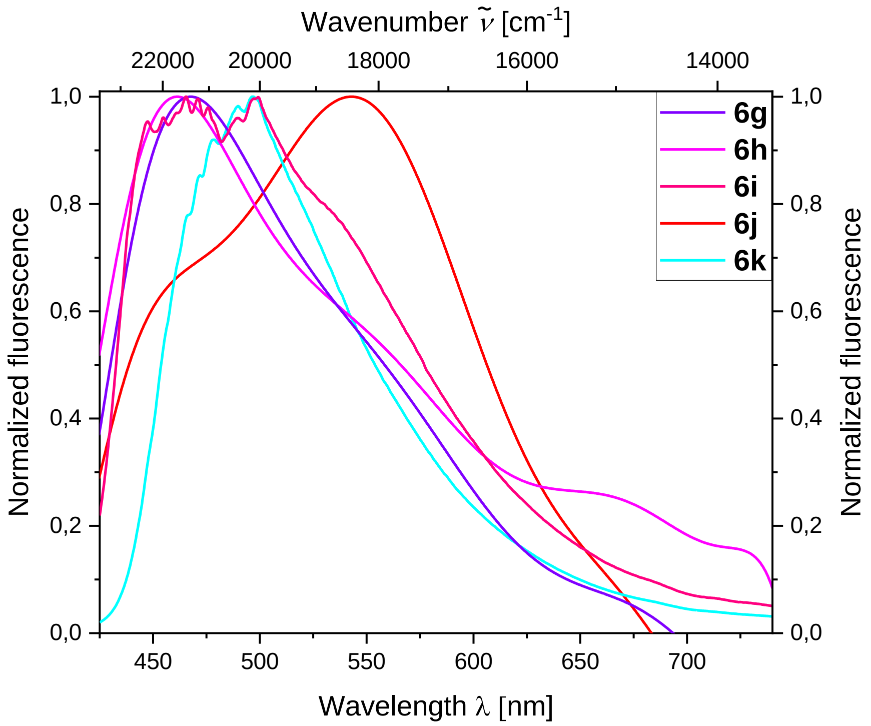

Figure S116. Normalized solid-state emission bands of selected triazole aroyl-*S*,*N*-ketene acetals **6**.

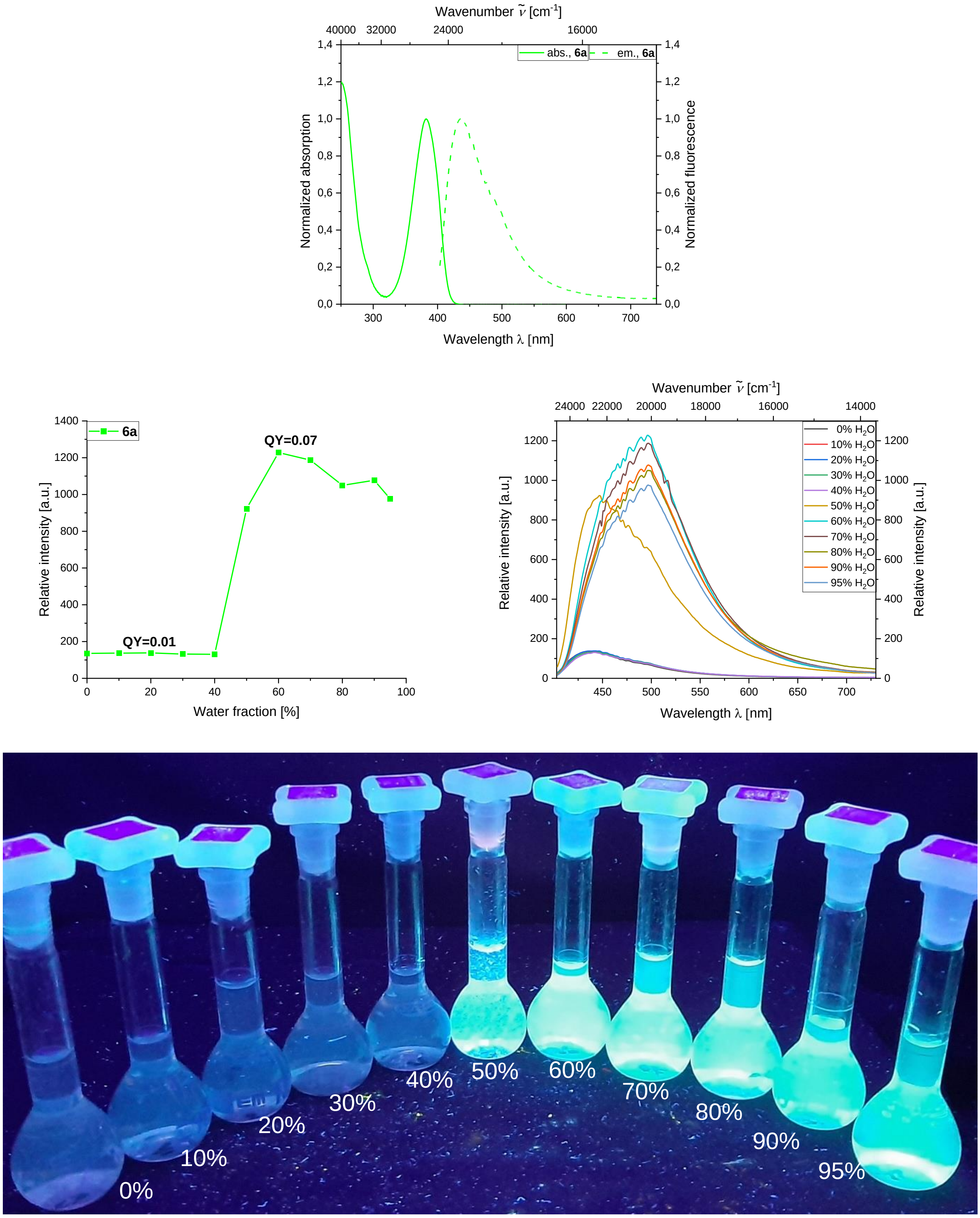

Figure S117. Absorption spectrum of **6a** in ethanol (top), and AIE-induced changes in emission (center, left), AIE-related emission spectra of compound **6a** (center, right) and photographs of solutions of dye **6a** in ethanol/water mixtures of increasing water content (bottom). The latter spectra were measured in ethanol/water mixtures of varying water content.

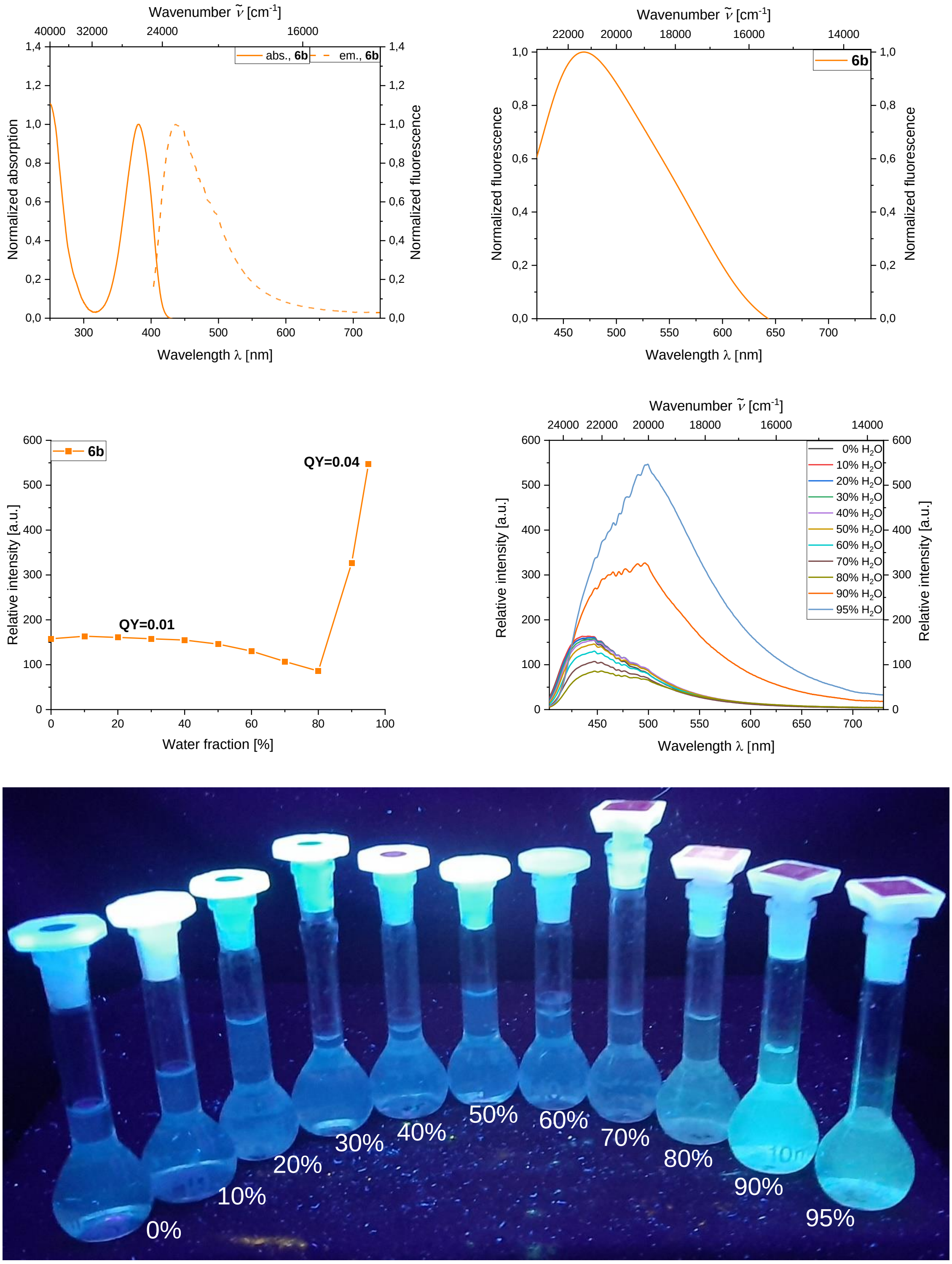

Figure S118. Absorption spectrum of **6b** in ethanol (top, left), solid-state emission spectrum (top, right), and AIE-induced changes in emission (center, left), AIE-related emission spectra of compound **6b** (center, right) and photographs of solutions of dye **6b** in ethanol/water mixtures of increasing water content (bottom). The latter spectra were measured in ethanol/water mixtures of varying water content.

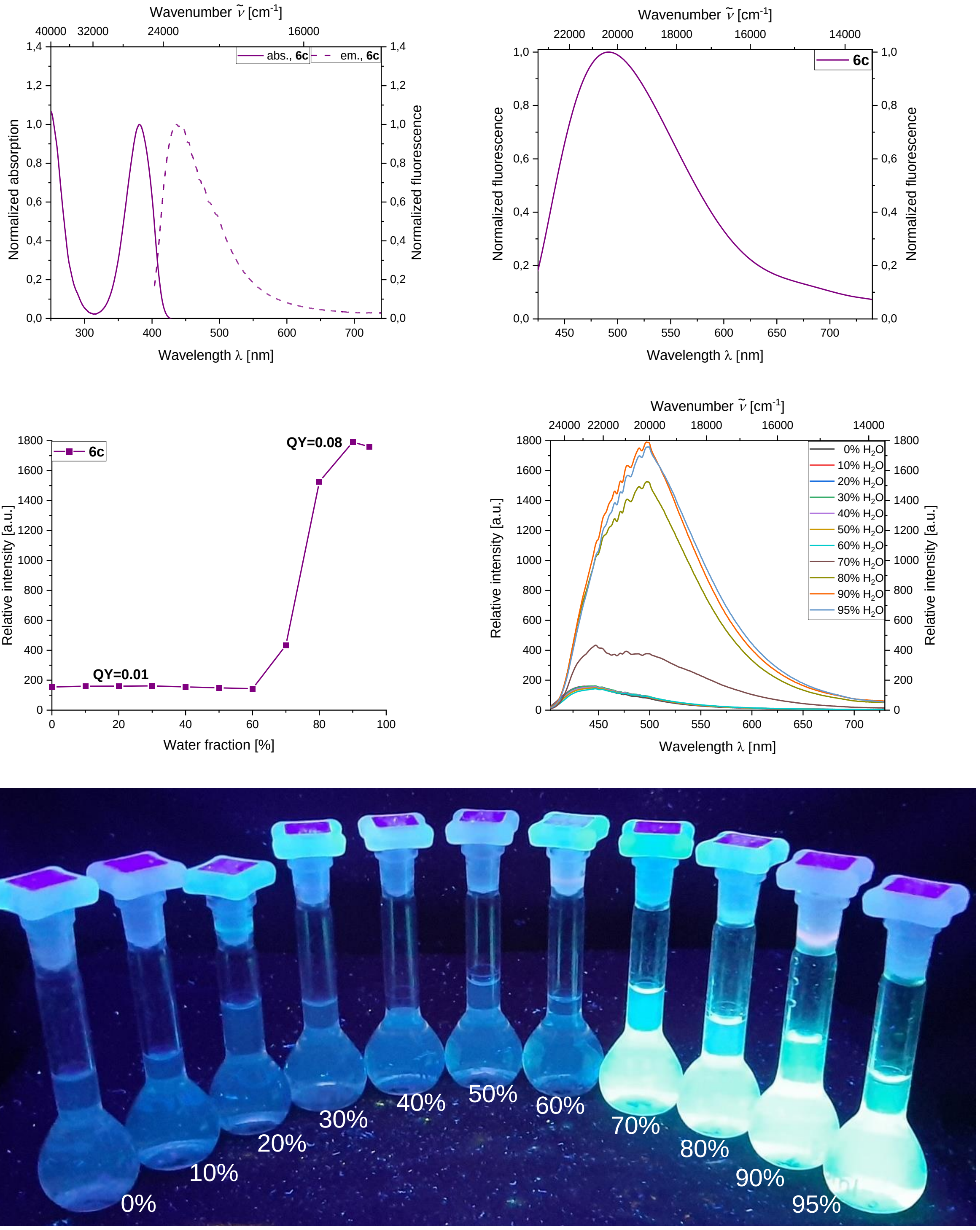

Figure S119. Absorption spectrum of **6c** in ethanol (top, left), solid-state emission spectrum (top, right), and AIE-induced changes in emission (center, left), AIE-related emission spectra of compound **6c** (center, right) and photographs of solutions of dye **6c** in ethanol/water mixtures of increasing water content (bottom). The latter spectra were measured in ethanol/water mixtures of varying water content.

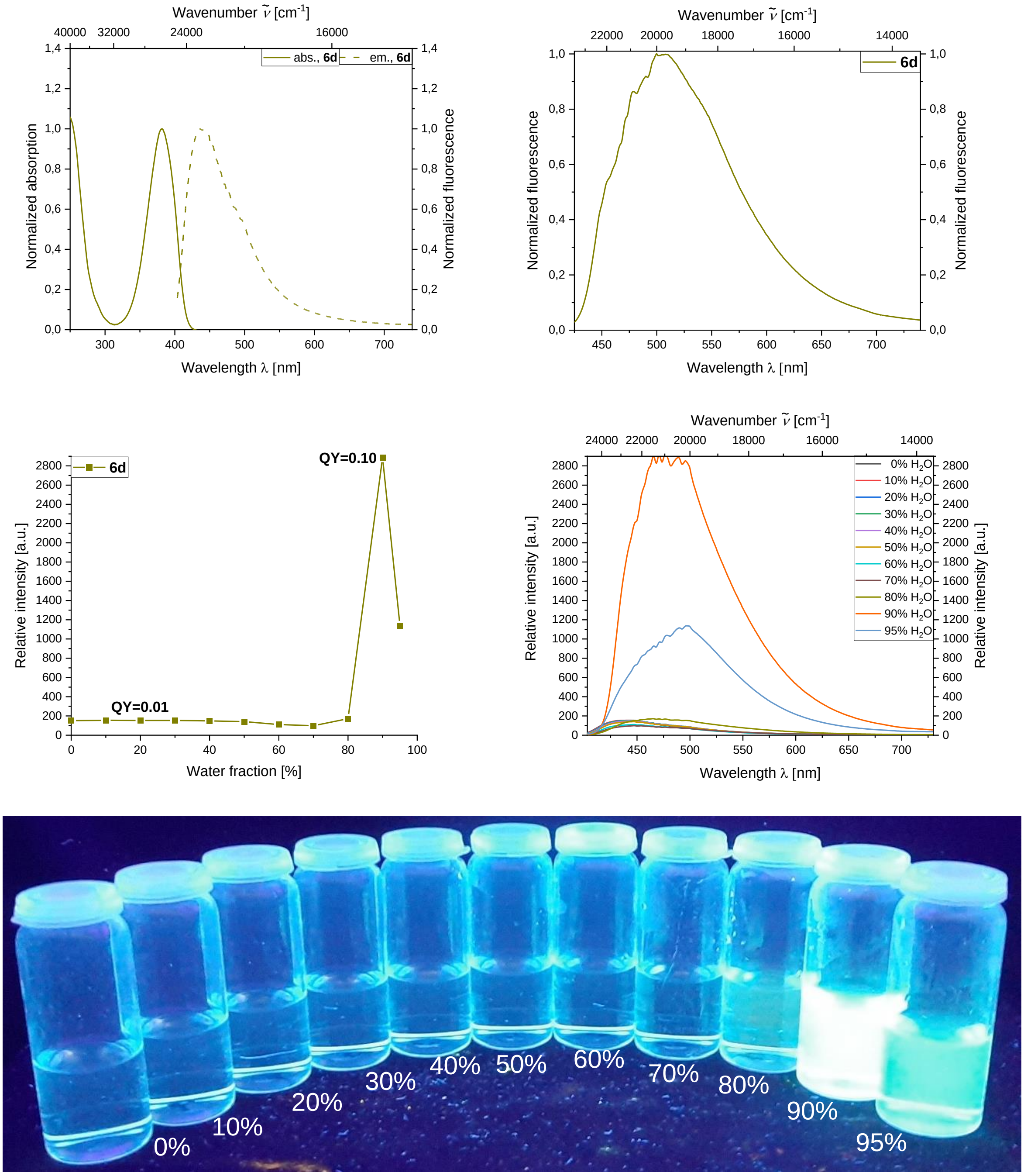

Figure S120. Absorption spectrum of **6d** in ethanol (top, left), solid-state emission spectrum (top, right), and AIE-induced changes in emission (center, left), AIE-related emission spectra of compound **6d** (center, right) and photographs of solutions of dye **6d** in ethanol/water mixtures of increasing water content (bottom). The latter spectra were measured in ethanol/water mixtures of varying water content.

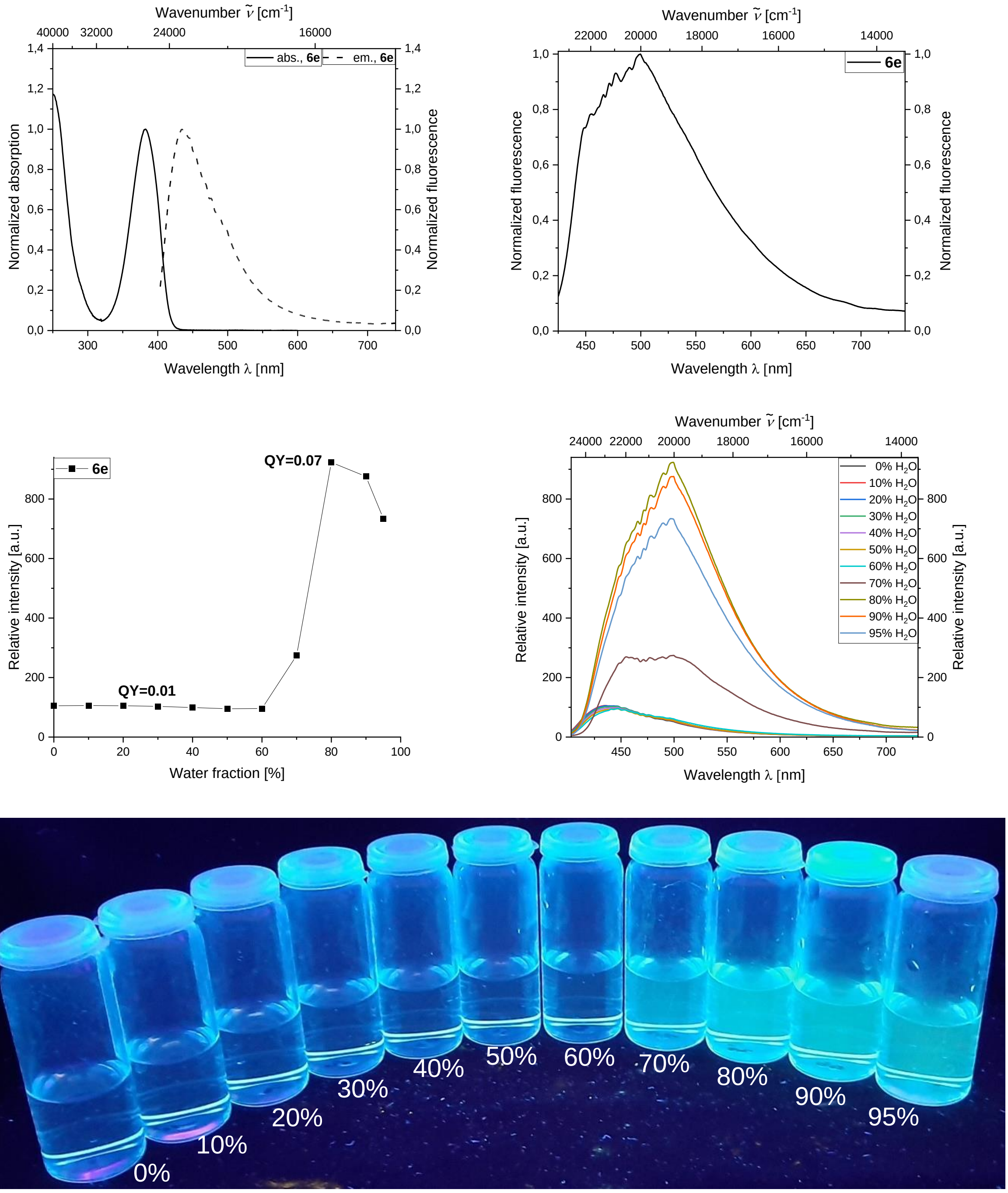

Figure S121. Absorption spectrum of **6e** in ethanol (top, left), solid-state emission spectrum (top, right), and AIE-induced changes in emission (center, left), AIE-related emission spectra of compound **6e** (center, right) and photographs of solutions of dye **6e** in ethanol/water mixtures of increasing water content (bottom). The latter spectra were measured in ethanol/water mixtures of varying water content.

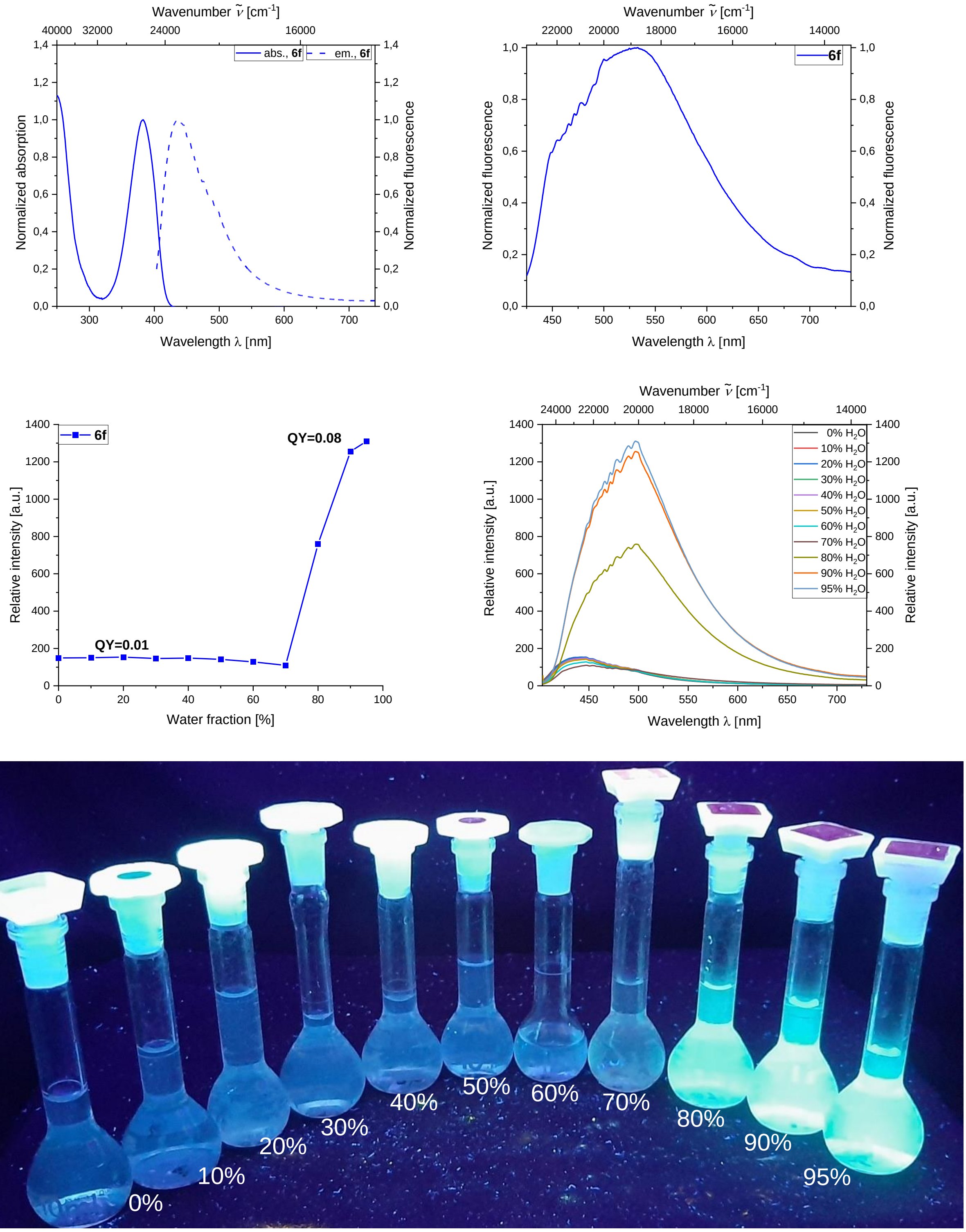

Figure S122. Absorption spectrum of **6f** in ethanol (top, left), solid-state emission spectrum (top, right), and AIE-induced changes in emission (center, left), AIE-related emission spectra of compound **6f** (center, right) and photographs of solutions of dye **6f** in ethanol/water mixtures of increasing water content (bottom). The latter spectra were measured in ethanol/water mixtures of varying water content.

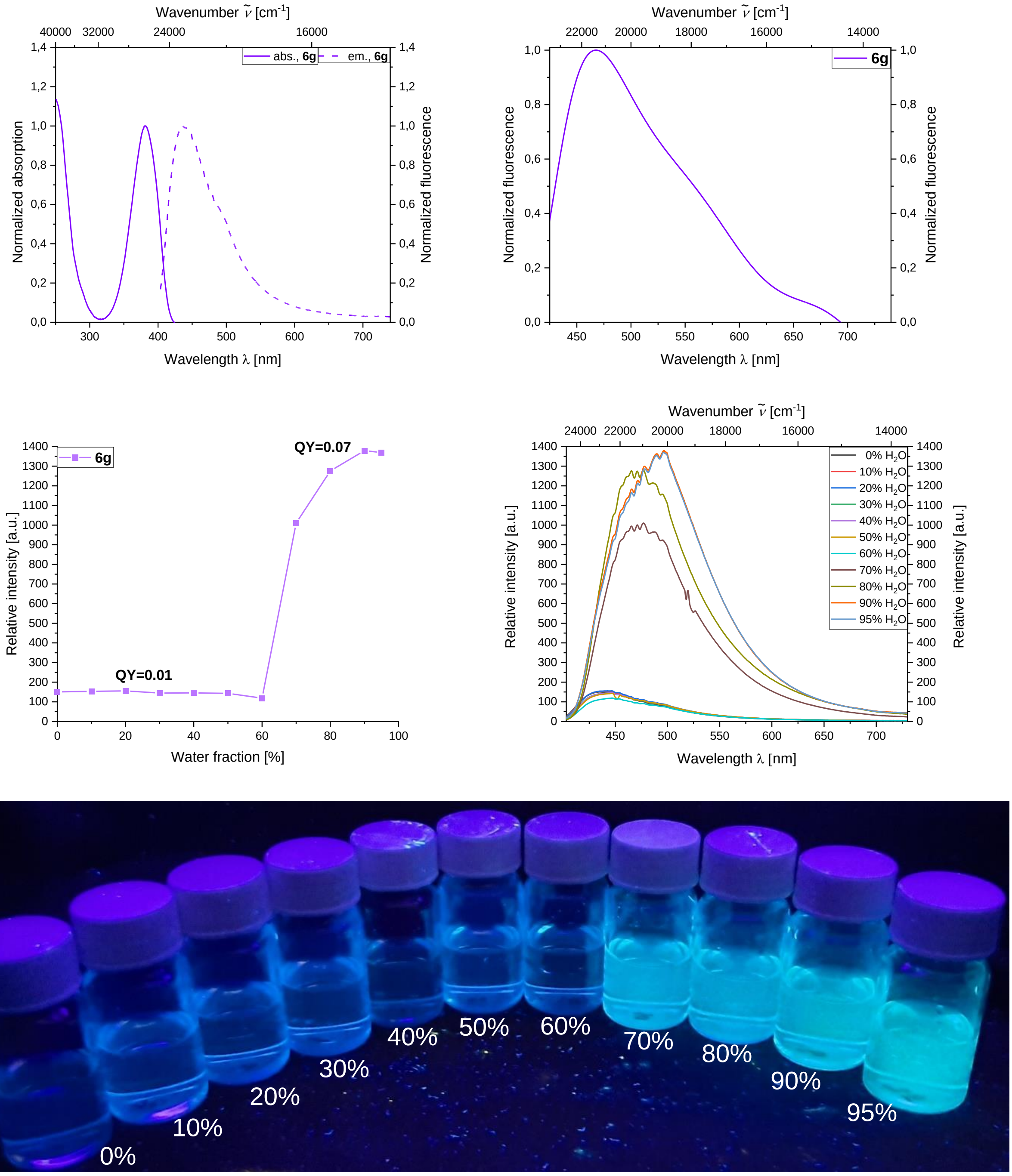

Figure S123. Absorption spectrum of **6g** in ethanol (top, left), solid-state emission spectrum (top, right), and AIE-induced changes in emission (center, left), AIE-related emission spectra of compound **6g** (center, right) and photographs of solutions of dye **6g** in ethanol/water mixtures of increasing water content (bottom). The latter spectra were measured in ethanol/water mixtures of varying water content.

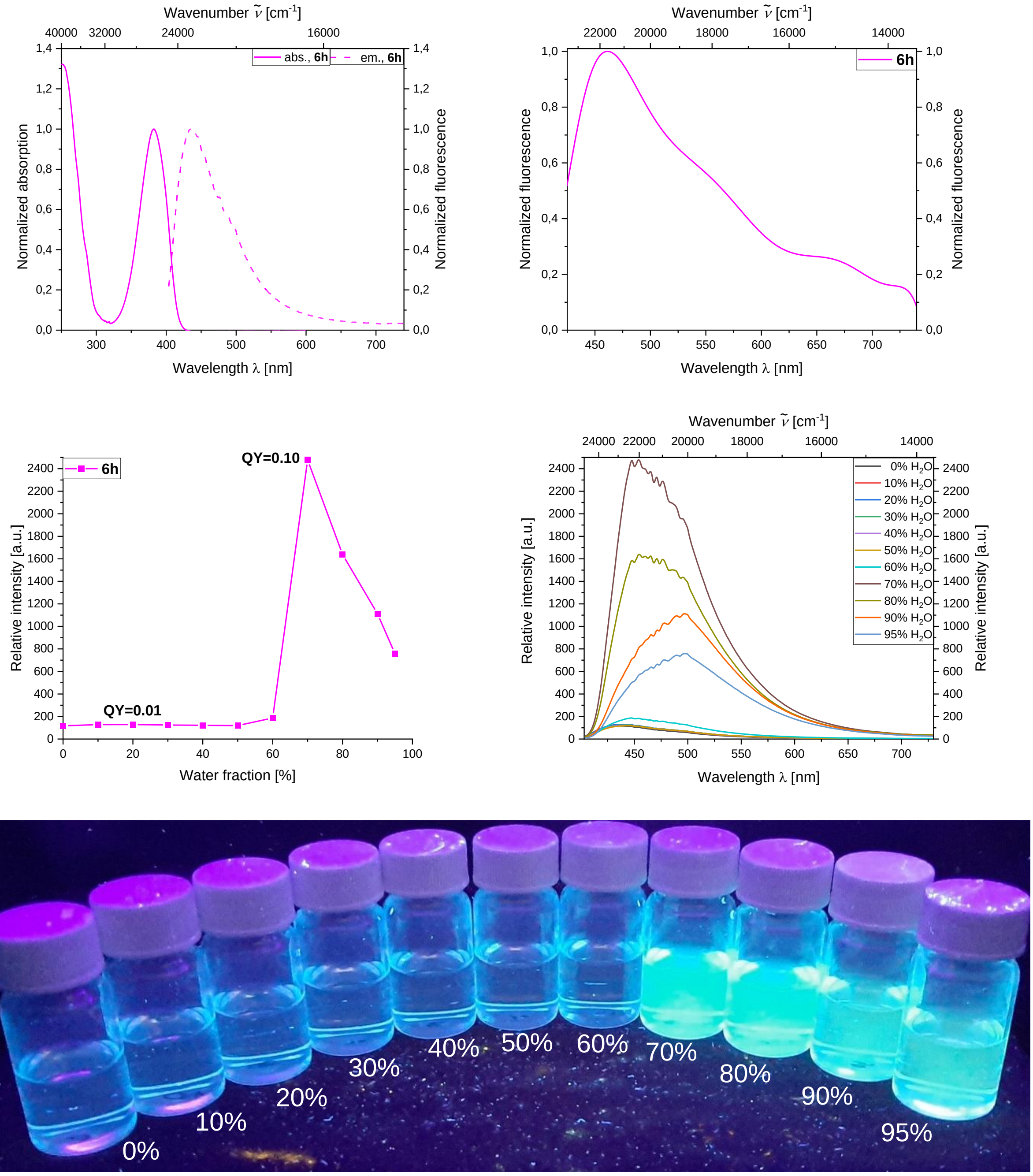

Figure S124. Absorption spectrum of **6h** in ethanol (top, left), solid-state emission spectrum (top, right), and AIE-induced changes in emission (center, left), AIE-related emission spectra of compound **6h** (center, right) and photographs of solutions of dye **6h** in ethanol/water mixtures of increasing water content (bottom). The latter spectra were measured in ethanol/water mixtures of varying water content.

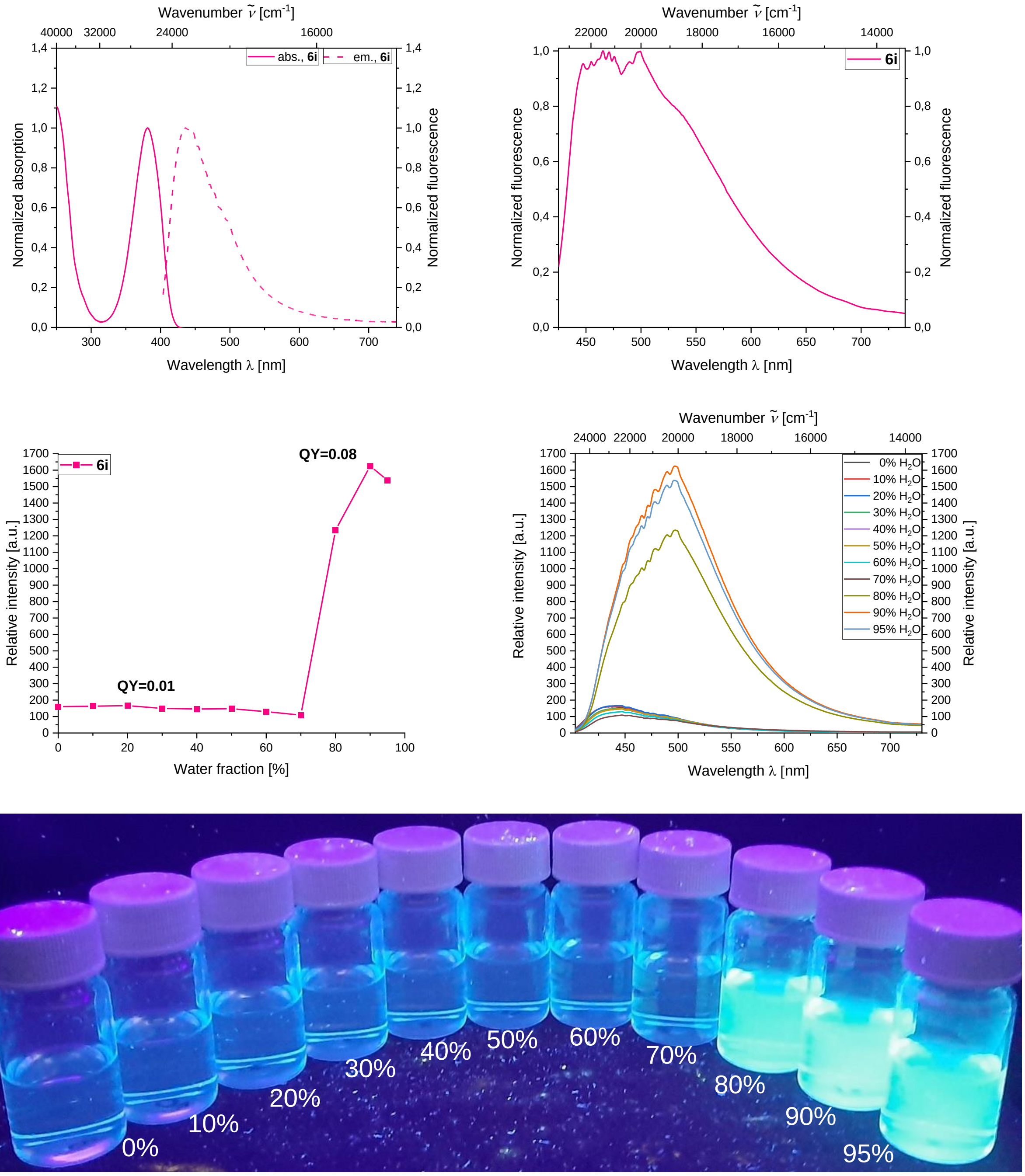

Figure S125. Absorption spectrum of **6i** in ethanol (top, left), solid-state emission spectrum (top, right), and AIE-induced changes in emission (center, left), AIE-related emission spectra of compound **6i** (center, right) and photographs of solutions of dye **6i** in ethanol/water mixtures of increasing water content (bottom). The latter spectra were measured in ethanol/water mixtures of varying water content.

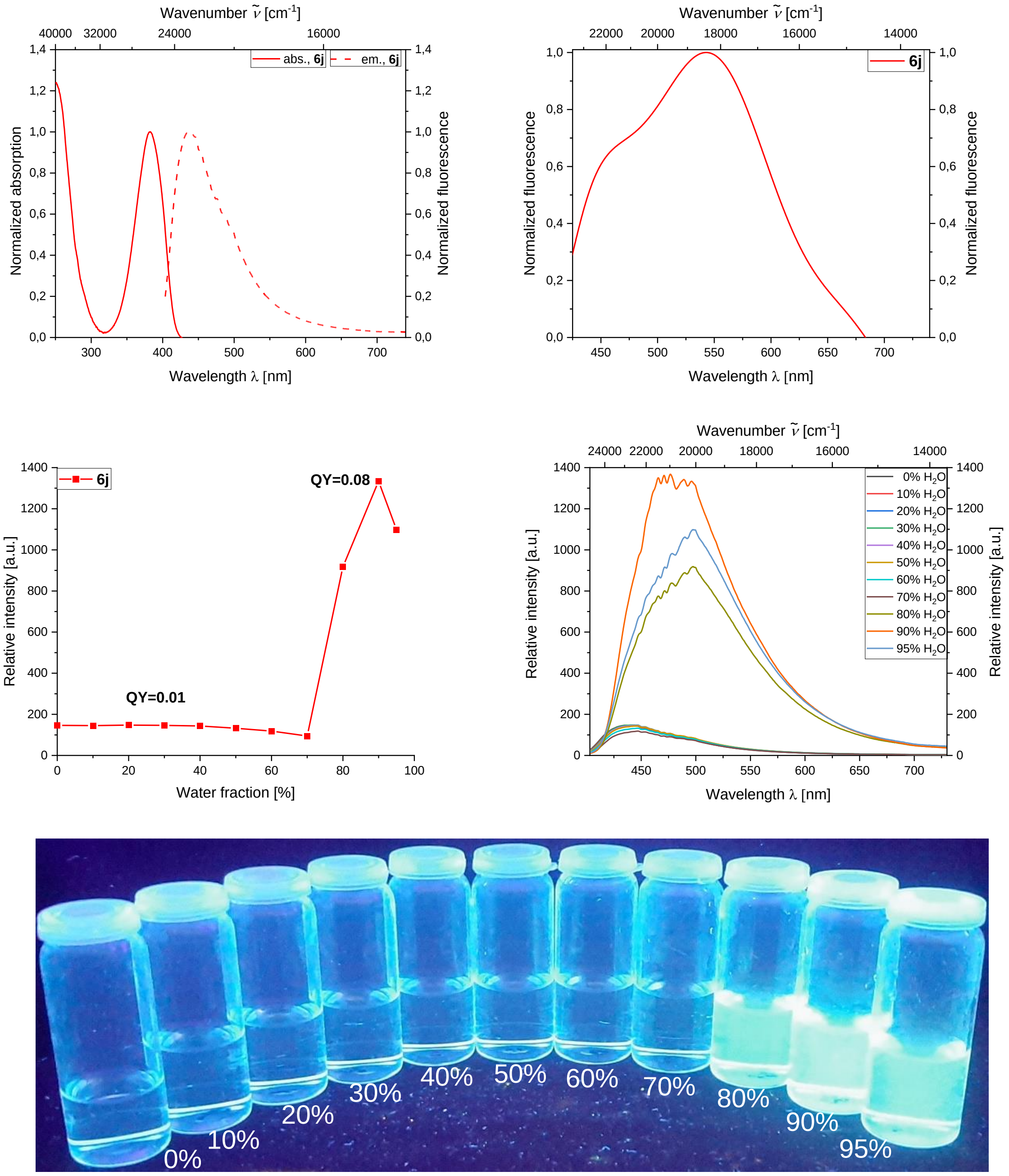

Figure S126. Absorption spectrum of **6j** in ethanol (top, left), solid-state emission spectrum (top, right), and AIE-induced changes in emission (center, left), AIE-related emission spectra of compound **6j** (center, right) and photographs of solutions of dye **6j** in ethanol/water mixtures of increasing water content (bottom). The latter spectra were measured in ethanol/water mixtures of varying water content.

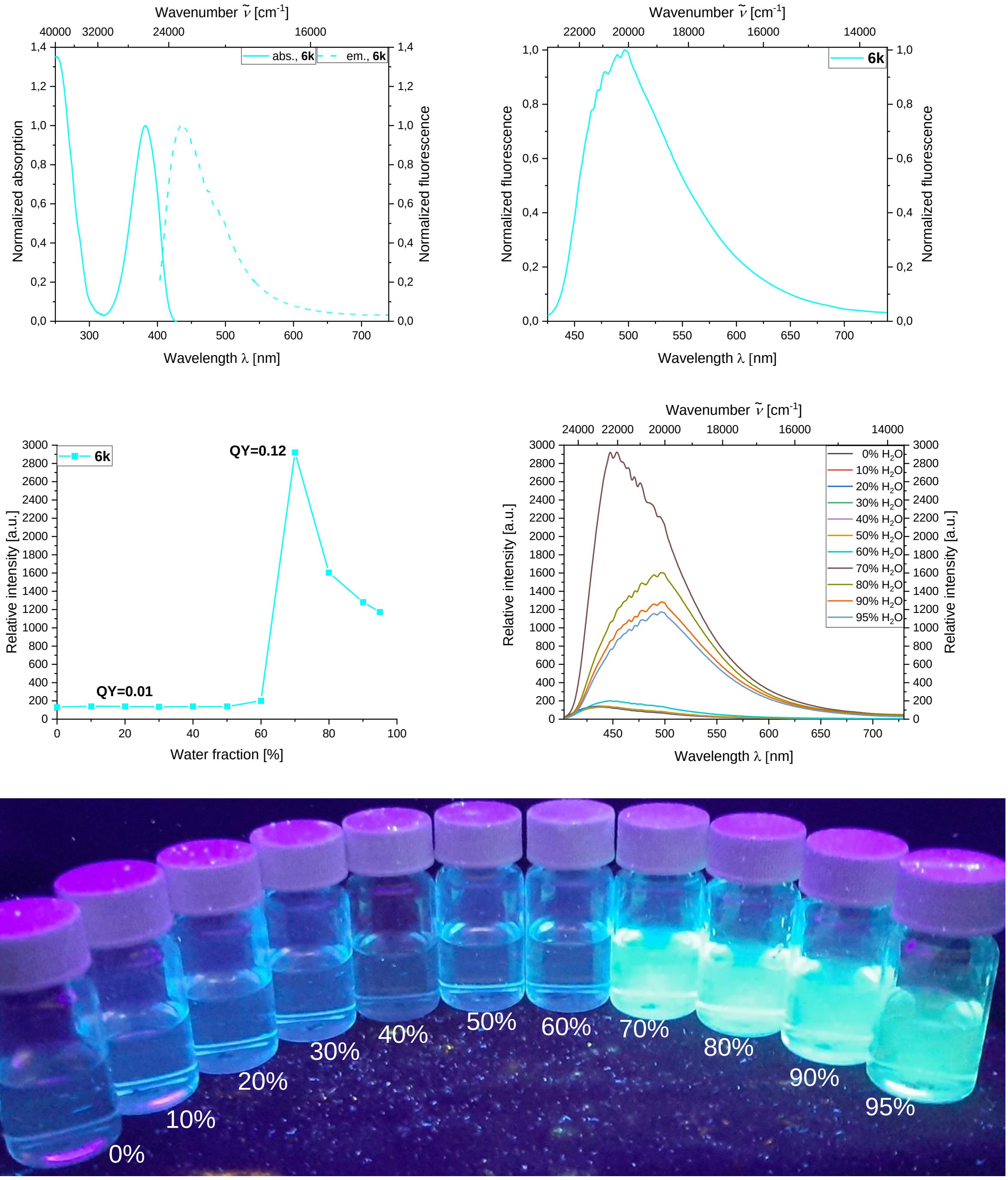

Figure S127. Absorption spectrum of **6k** in ethanol (top, left), solid-state emission spectrum (top, right), and AIE-induced changes in emission (center, left), AIE-related emission spectra of compound **6k** (center, right) and photographs of solutions of dye **6k** in ethanol/water mixtures of increasing water content (bottom). The latter spectra were measured in ethanol/water mixtures of varying water content.

11 Literature

[1] M. Kuhnert-Brandstatter, *Sci. Pharm.* **1966**, *34*, 147-166.

[2] M. Hesse, H. Meier, B. Zeeh, *Spektroskopische Methoden in der organischen Chemie*, Georg Thieme Verlag, Stuttgart, **2005**.

[3] S. K. Yen, L. L. Koh, F. E. Hahn, H. V. Huynh, T. A. Hor, *Organometallics* **2006**, *25*, 5105-5112.

[4] L. Biesen, N. Nirmalananthan-Budau, K. Hoffmann, U. Resch-Genger, T. J. J. Müller, *Angew. Chem. Int. Ed*. **2020**, *59*, 10037-10041.
